# Rapid Screening of Polyol Polyketides from Marine Dinoflagellates

**DOI:** 10.1021/acs.analchem.2c02185

**Published:** 2022-10-03

**Authors:** Adrián Morales-Amador, María L. Souto, Christian Hertweck, José J. Fernández, María García-Altares

**Affiliations:** † Departamento de Química Orgánica, Instituto Universitario de Bio-Orgánica Antonio González (IUBO AG), Universidad de La Laguna (ULL), Avenida Astrofísico Francisco Sánchez 2, 38206 La Laguna, Tenerife, Spain; ‡ Department of Biomolecular Chemistry, Leibniz Institute for Natural Products Research and Infection Biology, Hans Knöll Institute (HKI), Adolf-Reichwein-Straße 23, 07745 Jena, Germany; § Faculty of Biological Sciences, Friedrich Schiller University Jena, 07743 Jena, Germany; ∥ Department of Electronic Engineering, Rovira i Virgili University, 43007 Tarragona, Spain

## Abstract

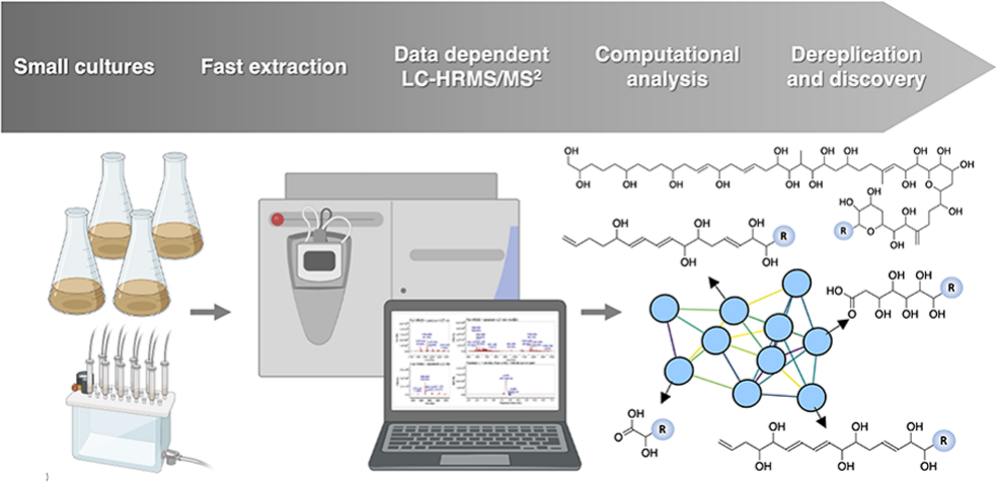

Dinoflagellate-derived polyketides are typically large molecules (>1000 Da) with complex structures, potent bioactivities, and high toxicities. Their discovery suffers three major bottlenecks: insufficient bioavailability, low-yield cultivation of producer organisms, and production of multiple highly related analogues by a single strain. Consequently, the biotechnological production of therapeutics or toxicological standards of dinoflagellate-derived polyketides is also hampered. Strategies based on sensitive and selective techniques for chemical prospection of dinoflagellate extracts could aid in overcoming these limitations, as it allows selecting the most interesting candidates for discovery and exploitation programs according to the biosynthetic potential. In this work, we assess the combination of data-dependent liquid chromatography coupled with high-resolution tandem mass spectrometry (LC–HRMS^2^) and molecular networking to screen polyol polyketides. To demonstrate the power of this approach, we selected dinoflagellate *Amphidinium carterae* since it is commonly used as a biotechnological model and produces amphidinols, a family of polyol-polyene compounds with antifungal and antimycoplasmal activity. First, we screened families of compounds with multiple hydroxyl groups by examining MS^2^ profiles that contain sequential neutral losses of water. Then, we clustered MS^2^ spectra by molecular networking to facilitate the dereplication and discovery of amphidinols. Finally, we used the MS^2^ fragmentation behavior of well-characterized luteophanol D as a model to propose a structural hypothesis of nine novel amphidinols. We envision that this strategy is a valuable approach to rapidly monitoring toxin production of known and unknown polyol polyketides in dinoflagellates, even in small culture volumes, and distinguishing strains according to their toxin profiles.

## Introduction

Dinoflagellates are marine microalgae that manufacture unusually long and complex polyketides, whose biogenetic origin is still poorly understood.^1^ Certain genera are responsible for harmful algal blooms that negatively impact the economy, environment, and public health. Those microalgae able to produce biotoxins can cause a wide range of severe symptoms like the polyether polyketide maitotoxin from *Gambierdiscus*, the longest and most toxic polyketide known hitherto.^2^ On the other hand, biotoxins show potent dose-dependent bioactivities that make them promising therapeutics and biomedical tools.^3,4^

However, several reasons limit the discovery of dinoflagellates polyketides through traditional pipelines. First, dinoflagellates are delicate under laboratory conditions: strains of the same species may have radically different culture behaviors, grow very slowly, yield poor cell harvests, and produce minute quantities of polyketide products. Second, biotoxin production is strain-specific, and each strain can biosynthesize multiple analogues with almost identical structures (e.g., positional isomerism of a single hydroxyl group).^3−7^ Due to their complex structures, their industrial synthesis is not feasible; thus, biotechnology is still the most promising approach for their commercial production.^4^ Since marine biotoxins have attracted interest from many sectors, from food safety to drug development, there have been a few recent attempts to develop bioprocesses for dinoflagellates,^8,9^ but part of their success relies on selecting suitable dinoflagellates strains.

The field of natural product discovery is rejuvenating thanks to the emergence of novel dereplication strategies. These aim to avoid rediscovering and isolating valueless compounds by unveiling the presence of chemical entities or molecular families in extracts of environmental samples or small cultures.^10−12^ In this budding field, new methods often rely on spectroscopy, spectrometry, and omics, supported by computational tools for data interpretation. Among them, nontargeted liquid chromatography coupled to high-resolution mass spectrometry (LC–HRMS) and tandem fragment analysis (MS*^n^*) stands out^13−15^ and is considered one of the most suitable techniques for analyzing dinoflagellate biotoxins.^15−20^ In fact, the selectivity and sensitivity of current mass spectrometers can overcome the limited chromatographic resolution between biotoxin analogues and the need to work with large volumes of culture.

Against all odds, the study of dinoflagellates by dereplication using LC-HRMS has not attempted except for a few recent studies,^20,21^ although it could ignite compound discovery programs by facilitating the selection of the best candidate strains to undergo biotechnological processes. The current main bottleneck of LC-HRMS*^n^* dereplication approaches is the lack of experimental reference data. Fortunately, there are several initiatives to expand repositories of experimental and in silico MS^2^ data^22−24^ and to combine LC-HRMS*^n^* data with other techniques that provide richer structural information, like NMR.

In this work, we propose a profiling and dereplication strategy based on LC-HRMS to investigate dinoflagellate *Amphidinium carterae* and its production of polyol polyketides. These types of compounds, together with polyether, constitute the main classes of “super-carbon-chain” biotoxins of dinoflagellates and include karlotoxins, palytoxins, symbiodinolides, etc.^1,2,25^*A. carterae* produces amphidinols (AMs), a family of lineal polyol-polyene compounds with potent activities against pathogens like *Candida* and *Mycoplasma* by direct interaction with cell membranes.^20,25−27^ Amphidinols’ amphipathic structure has a central core delimitated by two tetrahydropyran rings linked by a C6 alkyl chain. This system connects polyolic and polyene branches whose variations result in the corresponding AM congeners. Its mode of action consists of inserting the polyene moiety in the hydrophobic bulk of the phospholipid bilayers of cells.^28−32^ Therefore, slight modifications such as one more hydroxyl groups in the polyene or one sulfate at any position decrease their bioactivity and toxicity,^28,33,34^ contrary to what happens in other toxins.^35^

**Figure 1:**
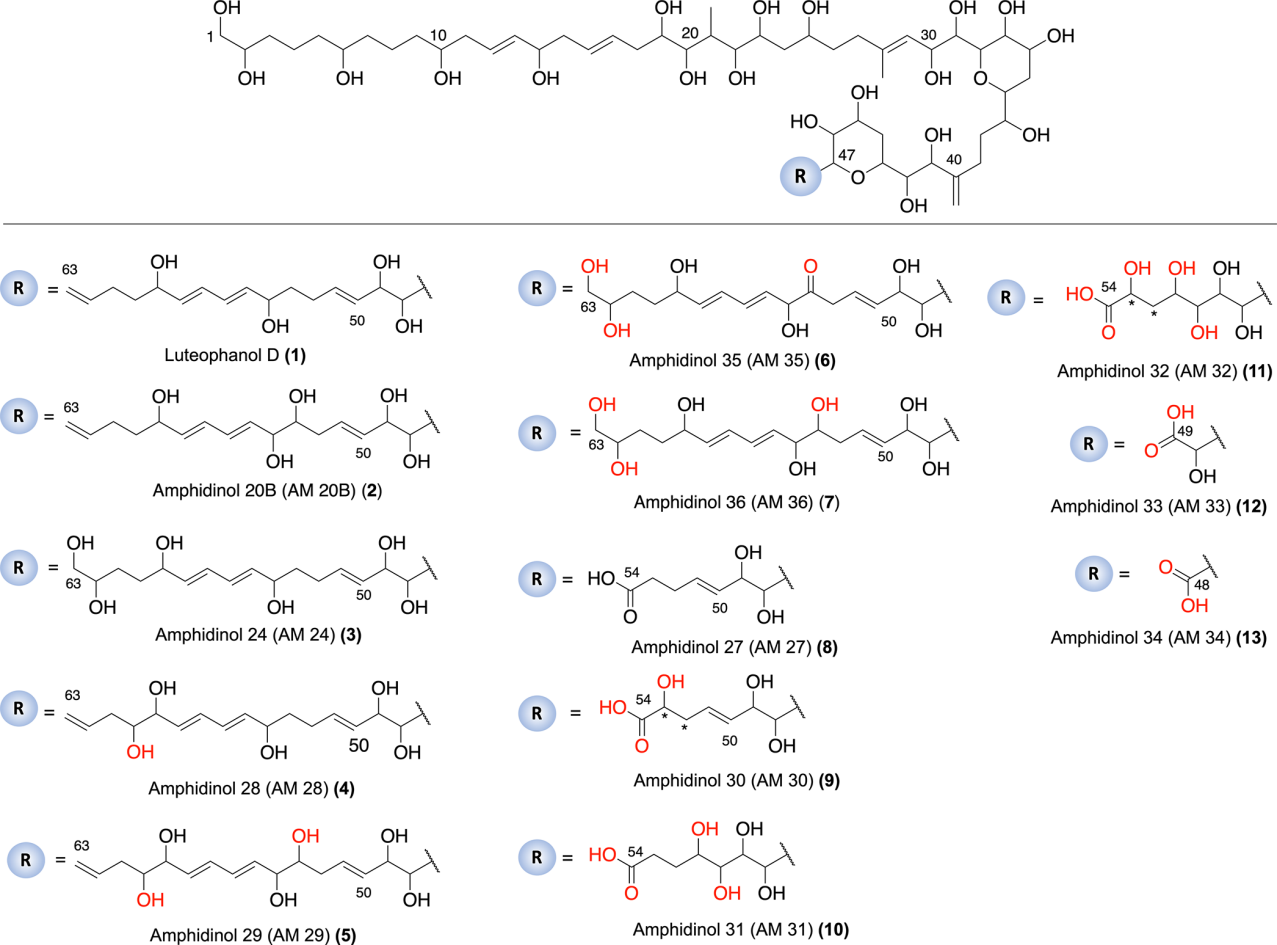
Structures of luteophanol D, AM 20B, AM 24, and AM 27–AM 36. The positions of functional groups in red are in hypothetical positions (not confirmed experimentally). (*) The position of the hydroxyl group in AM 30 and AM 32 can be located at position C52 or C53. Radical (R) starts at C47.

The potential of *A. carterae* in terms of metabolite production and culturing feasibility has put it in the spotlight to develop dinoflagellate-based bioprocesses for producing AMs from photobioreactors.^8,9,28^ We studied the metabolic production of four strains from different worldwide locations: Brazil coast (ACBR01), Reunion Island (ACRN02 and ACRN03), and Mauritius Island (ACMK03). Our profiling and dereplication strategy for AMs uses small amounts of extracts from low-volume cultures, and it combines data-dependent tandem mass spectrometry (dd-MS^2^) experiments and molecular networking (MN) analyses from the Global Natural Products Social Media (GNPS) platform.^36^ Molecular networking is a bioinformatic pipeline for MS^2^ fragmentation comparison that classifies similar MS^2^ spectra into molecular families organized by structural relationships. This strategy rapidly distinguished *Amphidinium* strains according to their AM production. Moreover, we confirmed the presence of known AMs in the extracts (like amphidinols 20B and 27) and isolated luteophanol D.^28,37^ We used the MS^2^ fragmentation behavior of this well-characterized amphidinol as a model to propose structural hypothesis of nine novel amphidinols (AMs 28–36), as shown in Figure 1.

## Materials and Methods

A full description of the Materials and Methods Section can be found in the Supporting Information.

### Microalgae Culture

*A. carterae* strains were isolated from the Brazilian coast (ACBR01), Réunion Island (ACRN02 and ACRN03), and Mauritius Island (ACMK03). They were kept in maintenance conditions as cultures of 125 mL stored in flasks of 250 mL, containing sterile modified Guillard K medium. Cultures were kept for 50 days to induce nutrient depletion stress.

### Cell-Free Medium and Biomass Extraction

*A. carterae* cultures were centrifuged, and supernatants were filtered with borosilicate filters. Pelleted cells were frozen, lyophilized, and extracted with three portions of 10 mL of methanol assisted by ultrasonication. The extraction of the organic content in cell-free media was done by solid-phase extraction (SPE, C18), desorbed using methanol, and dried by rotatory evaporation.

### Isolation and Purification of Luteophanol D

Luteophanol D was isolated from the cell-free medium of ACRN03 batch culture to be used as a self-made analytical reference, as described in Scheme S1. Luteophanol D was structurally characterized by NMR analysis (Table S1and Figures S1–S5) and electrospray ionization-HRMS (ESI-HRMS) as a monoisotopic peak at *m*/*z* 1329.7506 [M + Na]^+^ (theoretical *m*/*z* 1329.7546 for C_66_H_114_O_25_Na^+^).^8,30^

### Liquid Chromatography–High-Resolution Mass Spectrometry Experiments

Luteophanol D was analyzed by LC–ESI-HRMS–higher-energy C-trap dissociation-MS^2^ (LC–ESI-HRMS–HCD-MS^2^) in a Q-Exactive Orbitrap mass spectrometer to set the chromatographic and spectrometric parameters and to characterize and annotate its fragmentation pattern. ESI full HRMS spectra were acquired for the range of *m*/*z* 500–2000. Luteophanol D and the eight extracts of *A. carterae* were analyzed under data-dependent (dd) acquisition scan mode (LC–ESI-full HRMS/dd-MS^2^) with a top 5 set up. All of the extracts were solved with methanol (MeOH) to obtain 100 μL aliquots at 2 mg mL^–1^ and luteophanol D sample at 1 mg mL^–1^.

### Water Loss Analysis

The occurrence of dehydration events observed in positive ionization mode was counted by inspecting MS^2^ spectra and considering all signals with Δ*m*/*z* 18. For this purpose, a script was developed in R language (**RSript S1**).

### Molecular Networks on *A. carterae*

Raw files were exported to the universal readable mzXML format. The Global Natural Product Social Media (GNPS) platform was used to analyze mzXML files. Molecular networking was performed using its online workflow. MS^2^ spectra were filtered by choosing the top 6 peaks in the ±50 Da window throughout the spectrum. Data were clustered with MS-Cluster with a parent mass tolerance of 0.1 Da and a MS^2^ fragment ion tolerance of 0.1 Da to create a consensus spectrum. A network was created, and the edges were filtered to have a cosine score above 0.7 and more than six matched peaks.

## Results and Discussion

### Characterization of Luteophanol D

Isolated luteophanol D served to study the chromatographic and MS^2^ fragmentation behavior of AMs. Luteophanol D was detected in LC–HRMS positive and negative electrospray ionization (ESI) modes as the ions shown in Table S2and Reports S1 and S2.

**Figure 2:**
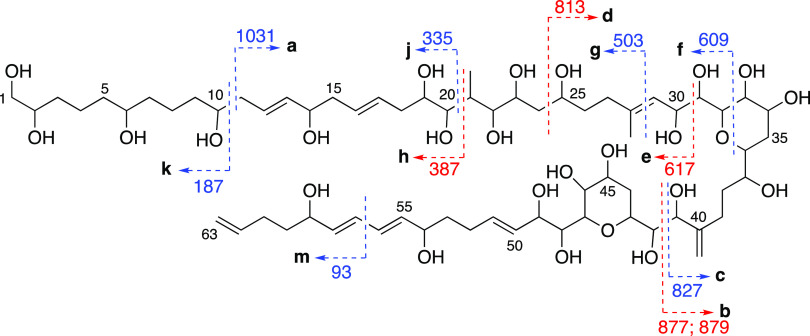
Fragmentation patterns observed in luteophanol D. Ions from positive mode are represented in blue, and ions observed in negative mode are represented in red. (Note: no fragment was named “i” nor “l” to avoid confusion with “j”).

[M + H]^+^ and [M – H]^−^ ions were chosen as precursors for MS^2^ experiments to set ionization and higher-energy C-trap dissociation (HCD) parameters to get the most descriptive fragmentation for further AM analysis (Reports S3 and S4 and Tables S3 and S4). MS^2^ spectra included abundant ion fragments produced by typical ESI charge-remote and charge-migration fragmentation for both ionization modes (Figure S6). Both tandem fragmentations were consistent with the luteophanol D structure (Figure 2), fully characterized by NMR (Table S1 and Figures S1–S5). Positive ESI ionization mode (ESI+) provided richer information, so it was prioritized to perform chemo-prospection and structural analysis, although fragments from both scan modes were analyzed.

The most intense peaks in positive ion mode MS^2^ spectra belonged to the precursor ion and the fragment resulting from cleavage **a** (C10/C11) and their series of conjugated polyenes derived from sequential dehydration. Other major ion fragments resulting from cleavages **f** (C32/C33, C36/O) and **c** (C41/C42) also define conserved region C1–C41, found along several AM subfamilies. Fragments from cleavages **k** (C10/C11), **g** (C28/C29), and **j** (C20/C21) were also recurrent and descriptive but showed weaker intensity (Figure 2, Tables S3 and S4, and Report S4).

In the negative scan mode, the molecular ion [M – H]^−^ stands out, followed by two fragments from cleavage **b** (C40/C41) to obtain ions of fragment C1–C41 at *m*/*z* 877 (C_44_H_77_O_17_^–^) and 879 (C_44_H_79_O_17_^–^) and cleavage **d** (C24/C25) that produces fragment C25–C63, found in all MS^2^ spectra of luteophanol D (Figure 2, Tables S3 and S4, and Report S3). The fragmentation pattern of the [M – H]^−^ ion of luteophanol D is highly consistent with the one reported by Wellkamp et al.^20^

While the C1–C41 segment is conserved along AM subfamilies like luteophanols or lingshuiols, the polyene region seems to be the most variable according to our previous studies on ACRN03 strain.^8,28^ In fact, tetrahydropyrans and the C6 chain in between are conserved in all AMs (Table S5) and even karlotoxins. Thus, ubiquitous fragments describing preserved regions, such as those from **c**, **b**, **f**, or **k** cleavages, can be considered like “hooks for fishing” AM analogues (i.e., finding new potential AM analogues). On the other hand, fragments from cleavages found in all MS^2^ spectra, like **a** or **d**, could inform about the variations described from smaller fragments of the polyene branch.

Another characteristic aspect of tandem MS for luteophanol D in ESI+ mode is the occurrence of massive dehydration events, giving a recognizable profile to MS^2^ spectra (Reports S4 and S5). It is observed as a series of conjugated polyenes derived from each fragment due to sequential losses of water. This is a typical effect in polyols when protonated adducts predominate, which has been explored as a tool for the theoretical elucidation of hydroxyls positions in other marine biotoxins.^38^ Thus, besides the usage of diagnostic fragments to screen AM analogues from MS^2^ data, we explored the potential of this ionization behavior for their detection.

### Dereplication of Luteophanol D and Identification of Analogues in *A. carterae* Cell-Free Medium Extracts

To screen known AMs in our extracts, all reported AMs hitherto (Table S5) were screened along the LC-HRMS spectra considering the *m*/*z* of expected adducts (+H^+^, +Na^+^, +K^+^, −H^–^, +HCOO^–^), with mass errors below 5.0 ppm. Luteophanol D and its analogues AM 20B (**2**), AM 24 (**3**), and AM 27 (**8**)^8,28^ were found as [M + H]^+^ and/or [M + HCOO]^−^ adducts in strains from Reunion Island (ACRN02 and ACRN03), as summarized in Table 1. No known AMs were dereplicated from ACBR01 and ACMK03 strains.

**Table 1:** Dereplicated AMs (Underlined) and New Proposed Analogues^a^

						[M + H]^+^ ion				[M + HCOO]^−^ ion				
compound	exact mass	Δlut D	equiv.	formula	RDB	*m*/*z* theo.	*m*/*z* exp.	Δppm	RT	*m*/*z* theo.	*m*/*z* exp.	Δppm	RT	strain
luteophanol D	1306.7649			C_66_H_114_O_25_	10.0	1307.7722	1307.7731	0.67	4.90	1351.7631	1351.7664	2.44	4.91	ACRN02
							1307.7703	–1.48	4.89					ACRN03
AM 28	1322.7598	+16	+O	C_66_H_114_O_26_	10.0	1323.7671	1323.7683	0.91	4.53	1367.7580	1367.7589	0.65	4.54	ACRN02
							1323.7655	–1.12	4.52		1367.7588	0.58	4.53	ACRN03
AM 20B	1322.7598	+16	+O	C_66_H_114_O_26_	10.0	1323.7671	1323.7673	0.17	5.15	1367.7580	1367.7612	2.34	5.07	ACRN02
														ACRN03
AM 29	1338.7547	+32	+O_2_	C_66_H_114_O_27_	10.0	1339.7620	1339.7631	0.77	4.70	1383.7529	1383.7557	2.02	4.70	ACRN02
							1339.7600	–1.51	4.69		1383.7544	1.54	4.72	ACRN03
AM 24	1340.7704	+34	+2OH	C_66_H_116_O_27_	9.0					1385.7686	1385.7694	0.57	4.45	ACRN02
											1385.7661	1.80	4.25	ACRN03
AM 27	1184.6554	–122	–C_9_H_14_	C_57_H_100_O_25_	8.0	1185.6627	1185.6602	–2.10	4.27					ACRN02
							1185.6580	–3.87	4.26					ACRN03
AM 30	1200.6503	–106	–C_9_H_14_; +O	C_57_H_100_O_26_	8.0	1201.6576	1201.6545	–1.80	4.21					ACRN02
							1201.6528	–3.94	4.23					ACRN03
AM 31	1218.6608	–88	–C_9_H_12_; +O_2_	C_57_H_102_O_27_	7.0	1219.6681	1219.6648	–2.73	4.15					ACRN02
							1219.6644	–3.03	4.17					ACRN03
AM 32	1234.6558	–72	–C_9_H_12_; +3O	C_57_H_102_O_28_	7.0	1235.6630	1235.6648	1.42	4.21					ACRN02
														ACRN03
AM 33	1100.5979	–206	–C_14_H_22_O	C_52_H_92_O_24_	7.0	1101.6051	1101.6027	–2.24	4.17	-				ACRN02
														ACRN03
AM 34	1070.5873	–236	–C_15_H_24_O_2_	C_51_H_90_O_23_	7.0	1071.5946	1071.5917	–2.70	4.23					ACRN02
														ACRN03
AM 35	1354.7497	+48	+3O	C_66_H_114_O_28_	10.0	1355.7569								ACRN02
							1355.7548	–1.61	4.68					ACRN03
AM 36	1356.7653	+50	+2OH; +O	C_66_H_116_O_28_	9.0	1357.7726				1401.7635				ACRN02
							1357.7732	0.45	4.21		1401.7622	–0.93	4.24	ACRN03

a Δlut D = mass difference with luteophanol D in Da; RDB = rings and double bond equivalents; Δppm = error between experimental and theoretical mass in ppm; RT = retention time (min).

This first dereplication attempt showed that AM production by the Reunion Island strains (ACRN02 and ACRN03) was dominated by luteophanol D and its derivatives. Therefore, we screened the presence of AM diagnostic fragments through full data-dependent MS^2^ experiments to identify new analogues in the samples. The diagnostic fragments were chosen according to their intensity, ubiquity, and ability to describe conserved regions of luteophanol D. In positive ionization mode, we used fragments from cleavages **k** (fragment C1–C10) and **f** (fragment C1–C32), and in negative ionization mode, we selected cleavage **b** (fragment C1–C41) at *m*/*z* 877 (Table S6).

The MS^2^ spectra of the potential precursors of AMs with at least one diagnostic fragment were investigated, and 13 precursors and 12 precursors in strains ACRN02 and ACRN03, respectively, were finally identified as AMs structurally related to luteophanol D (Table S6). The fragment from cleavage **f** was the most appropriate to pinpoint AMs: it could detect all potential AM precursors from ACRN02 and ACRN03, which in most cases were present in both strains and included luteophanol D (**1**), AM 20B (**2**), and AM 27 (**8**). Strain ACBR01 was found to be a poor producer of AMs with only two potential analogues (*m*/*z* 1277.7102 and 1145.6682) that produced the fragment from cleavage **f**, while strain ACMK03 did not produce luteophanol D or any analogue. Besides, no MS^2^ spectra from cell extracts of any strain contained diagnostic fragments of AMs, which is consistent with our observations that AMs are mostly excreted.^8,28^

### Sequential Neutral Losses of Water to Screen Polyols

**Figure 3:**
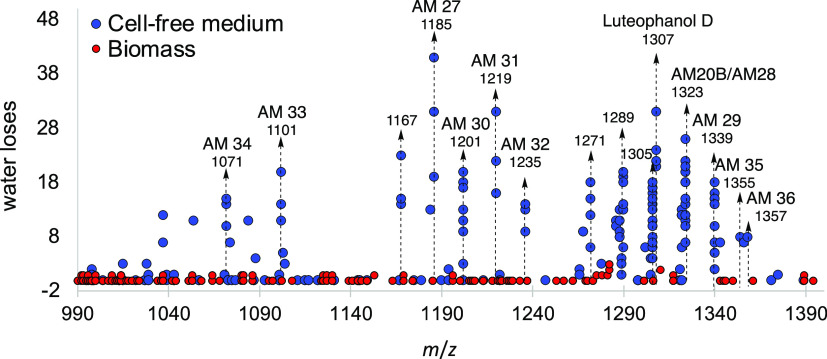
Dehydration events observed in MS^2^ spectra in *A. carterae* from Reunion Island (ACRN02 extracts) obtained from cell-free medium (blue dots) and biomass (red dots). Dashed lines indicate those precursor ions for AMs identified in this study. Each point represents a single data-dependent MS^2^ spectrum.

The total number of water losses for every fragmented parental ion was monitored (**RScript S1**) to investigate whether a large number of water losses was common in MS^2^ spectra of AMs (Figure S7). Using the extracts from strain ACRN02 as an example, it was observed that the precursor ions with many dehydration events in the mass range of *m*/*z* 800–1600 in Figure 3 were monocharged adducts of AMs, only found in the cell-free medium extract. Those precursors identified as AMs in this study are highlighted with dashed lines. This fragmentation behavior was common in MS^2^ spectra from all cell-free medium extract, except for the ACMK03 strain (Mauritius Island), which showed few dehydration events.

Nevertheless, we found that the number of dehydration events for each individual fragment in every averaged MS^2^ spectrum was very dependent on the parental ion intensity (Reports S5–S25). This fact is also shown in Figure 3 since different MS^2^ scan events for a given precursor have different numbers of water losses. Thus, we concluded that this parameter does not necessarily represent the amount of hydroxyl groups in the structure, according to our results for luteophanol D and other known analogues. However, they can inform about the polyol nature of the compound.

### Molecular Networking on *A. carterae* Extracts

**Figure 4:**
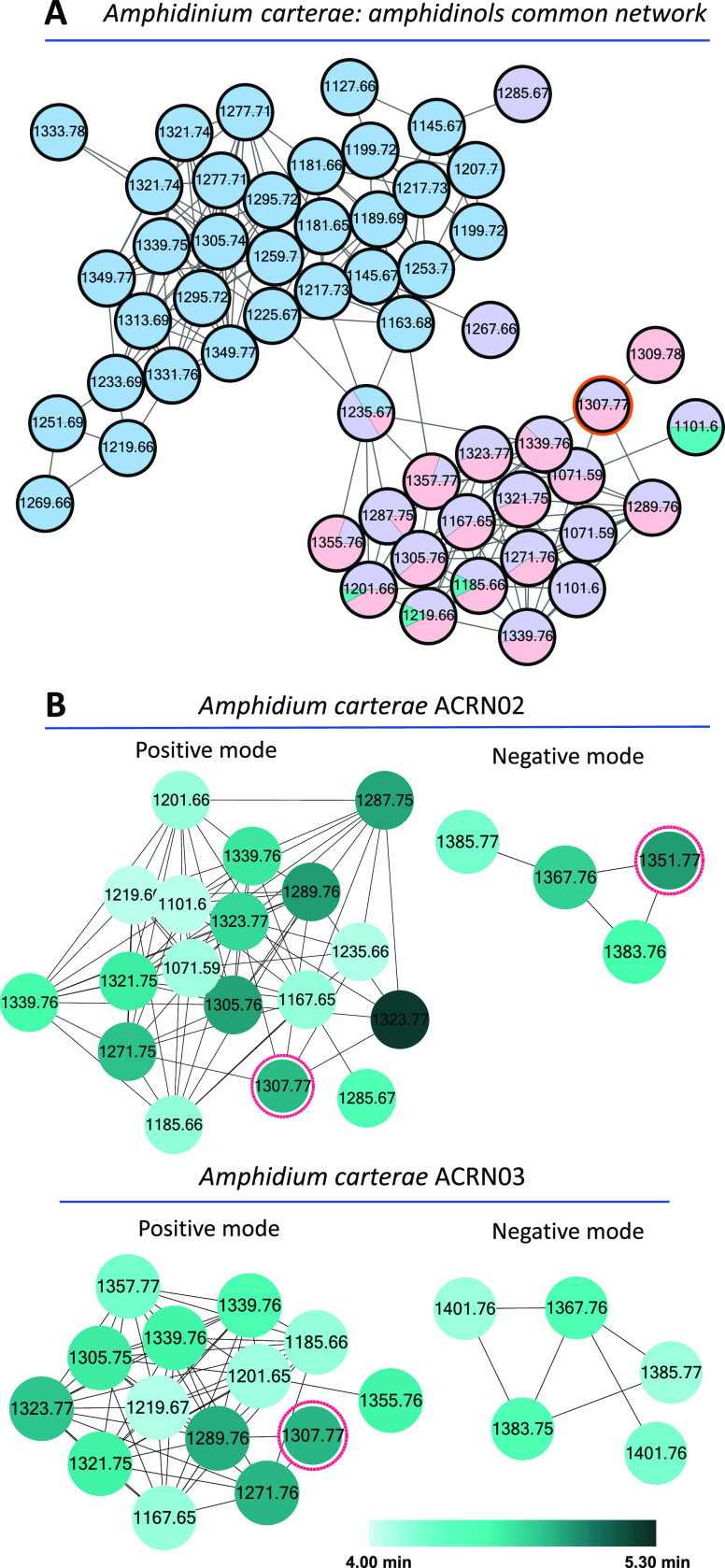
(A) Molecular network of cell-free medium for all *A. carterae* strains in positive ion mode (only the AM family is shown). Nodes are colored as a pie chart; the colored proportion represents the proportion of spectra coming from each strain. The node of luteophanol D is highlighted in red and serves as a seed to propagate the annotation of fragments to other nodes. (B) Family of AMs in individual molecular networks from strains ACRN02 and ACRN03 for both ionization modes. The nodes are colored by retention time.

Molecular networking on *A. carterae* cell-free medium extracts revealed the presence of one family of AMs, as it contained a node attributed to luteophanol D (Figure 4) and several nodes precursor ions that suffered neutral losses of water (Figures S7, S21, and S22). In this family, the Brazilian strain (ACBR01) contributed 33 nodes (in blue), connected to the nodes shared by the strains from Reunion Island (ACRN02 and ACRN03, pink and purple nodes, respectively) through node 1235.67. Strain ACMK03 shows a few shared nodes (in green) with Reunion Island ones (nodes 1101.60; 1185.66, 1201.66, and 1219.66) and only contributed one MS^2^ spectrum to the respective nodes, while the metabolomes of both strains from Reunion Island share 17 nodes including luteophanol D, AM 20B, and AM 27 as [M + H]^+^ adducts. These results suggest that polyketide production of strains from Reunion Island is highly similar, while compounds produced by strain ACBR01 (Brazil) are structurally divergent from luteophanol D. This reveals the surprising biosynthetic potential of the ACBR01 strain, but the lack of dereplicated compounds in its network prevented the structural description of its potential AMs.

To describe the AM-like compounds in detail, a molecular network analysis was performed for each strain individually. AMs were not found in strain ACMK03 (Mauritius Island) despite its shared nodes in the common molecular network, which means that AMs were probably very minoritarian compounds in the extract.

The AM families of ACRN02 and ACRN03 strains (Reunion Island) comprised 18 and 14 nodes in positive ion mode, respectively (Figure 4). As expected, both individual families shared several nodes, including luteophanol D (**1**, node 1307.77) and AM 27 (**8**, node 1185.66). To confirm duplicated nodes as isobaric compounds with *m*/*z* 1323.77 in ACRN02, we obtained the extracted ion chromatogram (EIC) with a mass tolerance of 5.0 ppm of theoretical *m*/*z* 1323.7671, corresponding to the [M + H]^+^ for AM 20B, which showed two peaks at RT 4.53 and 5.15 min, thus revealing two isomers. The first analogue was named AM 28 (**4**, Report S6), while the second was identified as AM 20B (**2**, Report S7) by further MS^2^ fragment analysis. The AM family in ACRN03 just showed one node 1323.77 at RT 4.52 min. Node 1339 also appeared twice in both families, but the EICs just showed one peak. In positive ionization mode, node 1101.60 was exclusive to ACRN02, while nodes 1355.76 and 1357.77 were exclusive to ACRN03.

A limitation of molecular networks is that the final nodes need to be curated, as some of them may result from in-source water losses. These in-source dehydration products can be identified as such because they have the same retention time and MS^2^ fragmentation (Figures S25 and S26). In samples ACRN02 and ACRN03, we found four in-source dehydration series from four precursor compounds: luteophanol D (*m*/*z* 1307.77, *m*/*z* 1289.76, and *m*/*z* 1271.75), AM 29 (*m*/*z* 1339.76 and *m*/z 1321.75), AM 20B and AM 28 (*m*/*z* 1323.77, *m*/*z* 1305.76, and *m*/*z* 1287.75), and AM 27 (*m*/*z* 1185.66 and *m*/*z* 1167.65).

ACRN02 and ACRN03 strains also contained one small AM family in the molecular network computed for negative ionization as [M + HCOO]^−^ adducts, comprising four and five nodes, respectively, that included AM 20B (**2**), AM 24 (**3**), and AM 27 (**8**) in both strains and luteophanol D in ACRN02 (Table 1 and Figure 4). Strain ACRN03 had an exclusive node in negative ionization mode at *m*/*z* 1401.76.

### Description of Nine New Amphidinol Analogues

We used the MS^2^ fragmentation behavior of luteophanol D as a model to describe those novel AMs selected due to the presence of diagnostic fragments and dehydration events in their MS^2^ spectra and the relationships between their nodes in the molecular networks. This description was based on positive ionization mode MS^2^ fragmentation since it was the most informative, while the negative ion fragments were used to verify the observations. Thus, luteophanol D served as a “seed” from which to propagate the structural information throughout the rest connecting nodes by analyzing shared and own fragments of each one. In addition, the presence of known compounds along networks was used as supportive links to ensure the coherence of the proposed structures (Scheme S2). These “anchor” compounds used were AM 27 (**8**, present in positive ionization mode for both strains), AM 20B (**2**, only in ACRN02 in both ionization modes), and AM 24 (**3**, in both strains but only in negative ionization mode).

We characterized nine new analogues from ACRN02 and ACRN03 strains using this approach, which were named AMs 28–36 and ranged from *m*/*z* 1071.59 to 1357.77 (Figure 1 and Table 1). Analogues AMs 28–31 emerged for both strains, while AMs 32–34 appeared in just ACRN02 and AM 35 and AM 36 appeared in only ACRN03. AM 28 (**4**), AM 29 (**5**), and AM 36 (**7**) were detected as both [M + H]^+^ and [M + HCOO]^−^ adducts, while the rest (AMs 30–35) were only detected in positive ionization mode. Table 1 shows the information related to these compounds, including the producer strain and ionization mode in which they were identified.

All analogues reported in this study conserve the region from C1 to C48 identical to luteophanol D based on the following arguments:

(i) Fragments resulting from cleavages **b**, **c**, and **f** suggest that region C1–C41 is conserved for most analogues, except for AM 35 and AM 36 that lack signals for these respective fragments in their MS^2^ (Figures 1 and 2).
(ii) Fragments from cleavage **s** or **w** and the crossing with fragments **r**, **u**, and **v** for AM 28, AM 29, and AM 35 suggest that structural changes are located in the lower branch (Table S11).
(iii) Cleavages **a** and **d**, together with other fragments (Reports S5–S34), ensure that the second tetrahydropyran ring is the only possibility to the calculated molecular formula for those analogues that do not accomplish argument (ii).
(iv) The structural region from C31 to C48 that includes both tetrahydropyran rings is conserved among AMs, in accordance with the previous literature (Table S5).

Therefore, the new analogues AMs 28–36 would deviate from luteophanol D at the region of molecule C48–C63 that includes hydroxylation and C–C oxidative cleavages (Scheme S2). These occur at four potential oxidation carbon points of luteophanol D, located at carbons C53 and C60, and at the double bond between C62 and C63. The hydroxylation of these positions in luteophanol D produces the second generation of precursors: AM 24 is oxidized at C62 and C63, isobaric compounds AM 20B and AM 28 are oxidized at positions C53 and C60, respectively, and AM 29 is oxidized at C60 and C53. The rest of analogues derived from further oxidations on these precursors.

#### (a) Amphidinol 24 and Derivates

AM 24 was only detected as [M + HCOO]^−^ adduct (*m*/*z* 1385.7694, C_67_H_117_O_29_^–^) in both strains of Reunion Island. The calculated molecular formula and the MS^2^ fragmentation fitted with its structure, which was fully characterized by NMR in our group.^28^ It had two additional hydroxyl groups with respect to luteophanol D, forming a 1,2-dihydroxyl system that replaces the terminal double bond at C62/C63. AM 35 and AM 36 were found to be derivatives of AM 24, with one and two more oxidation grades, respectively. AM 36 was observed as *m*/*z* 1357.7732 ([M + H]^+^, C_66_H_117_O_28_^+^) and *m*/*z* 1401.7622 ([M + HCOO]^−^, C_67_H_117_O_30_^–^) and was expected to have an additional hydroxyl group on an sp^3^ carbon between C52 and C63; however, fragmentation data did not allow to locate it more precisely. AM 35 appeared at *m*/*z* 1355.7548 ([M + H]^+^, [C_66_H_115_O_28_]^+^) and displayed one additional instauration compared with AM 36 in region C42–C63, according to the elemental formula of fragment from cleavage **s** (Table S11). Cleavages **u** and **v** suggested that there was a ketone on C52 or C53, although C53 is more likely since it is a hydroxylated center in AM 20B.^8^

#### (b) Amphidinols 20B and 28

The EICs of *m*/*z* 1323.7671 and *m*/*z* 1367.7569 ([M + H]^+^, C_66_H_115_O_26_^+^ and [M + HCOO]^−^, C_67_H_115_O_28_^–^) showed the presence of two isobaric compounds referred to as AM 28 (RT 4.53 min) and AM 20B (RT 5.12 min), respectively, represented as two nodes in the molecular network of ACRN02 strain. According to the elemental formulae of precursor ions, they displayed one oxygen atom and one oxidative grade more than luteophanol D; so, they are subjected to a hydroxylation on an sp^3^ carbon (Scheme S2). An interpretation of internal fragments relevant to the C42–C63 segment provided discerning structural information about AM 28: cleavage **s** located the extra oxygen beyond C43, and the combination of cleavages **s** and **p** ensured the presence of alyl-vic-diol between C48 and C51. An insightful cross of **o** and **p** cleavages resulted in a C1–C54 superimposable segment compared to that in luteophanol D, which limited the possible locations of the extra hydroxyl group to C61, which was supported by fragment **n** (Table S11). The fragmentation pattern of AM 20B could not provide enough insight to locate the extra hydroxyl group, but the previous studies based on NMR unequivocally located it at carbon C53.^8^

#### (c) Amphidinol 29

AM 29 was identified as the monoisotopic ions *m*/*z* 1339.7631 ([M + H]^+^, C_66_H_115_O_27_^+^) and *m*/*z* 1383.7557 ([M + HCOO]^−^, C_67_H_115_O_29_^–^) in ACRN02 and ACRN03. In accordance with its elemental formula, this compound had two more oxygen atoms than luteophanol D. In this case, fragments from cleavage **t** allowed to set modifications beyond C50, and **s** established the presence of two hydroxyl groups on sp^3^ carbons (Table S11). Although any carbon beyond C51 was suitable to bear these extra hydroxyl groups, C53 and C60 positions were considered the most probable ones because these are hydroxylated in AM 20B and AM 28. Therefore, AM 29 could result from double hydroxylation of luteophanol D or from one oxidative process from AM 20B or AM 28 (Scheme S2).

#### (d) Analogues Resulting from Oxidative C–C Cleavages

AM 27, AM 33, and AM 34 were predicted to be oxidative truncated versions of luteophanol D at C54, C49, and C48, respectively. AM 27 was found at *m*/*z* 1185.6602 ([M + H]^+^, C_57_H_101_O_25_^+^) as a truncated analogue that differs in C_9_H_14_ with respect to luteophanol D, indicating an oxidative cleavage at C54. The fragment resulting from cleavage **s** described the C42–C54 moiety as AM 27 (Reports S10 and S21), which was fully characterized by NMR in our laboratory previously^28^ from the ACRN03 strain. AM 33 was identified as a monoisotopic peak at *m*/*z* 1101.6027 [M + H]^+^ (C_52_H_93_O_24_^+^). The molecular formula predicted for the precursor ion and the fragment ion from cleavage **a** (*m*/*z* 861.4466, C_42_H_69_O_18_^+^) revealed a shorter structure than luteophanol D. Therefore, cleavage **c** (*m*/*z* 809.5004, C_44_H_72_O_13_^+^) together with a difference of C_14_H_22_O versus luteophanol D might suggest an oxidative cleavage on the allyl alcohol at carbon C49 with the conversion of the hydroxyl group into a carboxylic group (Report S15), as in AM 27. AM 34 was identified as monoisotopic peak *m*/*z* 1071.5917 ([M + H]^+^, C_51_H_91_O_23_^+^) and its diagnostic ion fragment **a** (*m*/*z* 831.4365, C_41_H_67_O_17_^+^) manifested the same oxidative cleavage on C48 that bears a carboxylate group (Scheme S2 and Report S16). AM 30 (*m*/*z* 1201.6545 [M + H]^+^, C_57_H_101_O_26_^+^) had one oxygen more than AM 27, as supported by fragments from cleavages **s** and **w** that established the position of the hydroxyl group at carbon C52 or C53 (Table S11). AM 31 (*m*/*z* 1219.6648 [M + H]^+^, C_57_H_103_O_27_^+^) had two hydroxyl groups more and one unsaturation less than AM 27, as supported also by cleavage **a** (*m*/*z* 961.4975, C_47_H_77_O_20_^+^), produced probably by hydroxylation of the double bound at C50 and C51 (Reports S13 and S23). A comparison of their respective fragments from cleavage **a** showed that AM 32 (*m*/*z* 1235.6648 [M + H]^+^, (C_57_H_103_O_28_^+^)) possessed one additional hydroxyl group with respect to AM 31, comparing their fragments from cleavage **a** (*m*/*z* 995.5057, C_47_H_81_O_23_^+^).

#### (e) Structural Relationships among AMs

We propose that analogue AM 27 and those from AM 30 to AM 34 are the result of oxidative cleavages at C54 in luteophanol D. The structure of these analogues can be explained by a plausible oxidative cascade, as shown in Scheme S2. Our proposal is based on the well-established structure of metabolites AM 20B, AM 24, AM 27, and the new AM 28. According to this proposal, the oxidative cleavage at C54 in luteophanol D structure would generate truncated analogues with terminal carboxylic acids. The cascade would start with an analogue with a terminal aldehyde, compound AM 26, which was not detected in the current study. However, AM 26 was isolated from the same strains and characterized by NMR in a previous study.^28^ AM 26 would transform into AM 27 as its carboxylic version. Then, AM 31 would be explained as a branch of AM 27 by a dihydroxylation at the C50–C51 double bond. In addition, from these intermediates, it is possible to originate one of the isobaric structures for AM 30 and AM 32. A sequence of oxidative cleavages would produce AM 33 and the shortest compound AM 34.

## Conclusions

In this work, we propose a strategy based on data-dependent MS^2^ comparisons by molecular networks to screen polyol polyketides from dinoflagellates using extracts from small culture volumes. Our strategy can distinguish between producers of structurally related and nonrelated analogues and help describe unknown compounds.

Sequential water losses are a distinctive feature of MS^2^ fragmentation in the positive mode of polyols. Molecular networks can easily group spectra that contain this feature, revealing families of compounds with a polyol nature, such as AMs or other polyketides with many hydroxyls in their structure. Water losses have also been proposed as a tool for elucidating the number and position of hydroxyl groups in other polyketides like ovatoxins,^38^ but we concur with previous studies^39^ that recognized that water losses are highly dependent on precursor intensity. On the other hand, one of the limitations of our approach is the low chromatography resolution between highly similar analogues of polyols, which influences the quality of MS^2^ data. Besides, molecular networks require a curating step as they cannot discriminate between genuine precursor and in-source fragments that may occur (such as expected in-source water losses). Finally, the structural characterization of new analogues depends on the presence of known and well-characterized compounds (“seeds”) to propagate their structural information throughout the network.

We consider that our strategy can be applied to the study of other compounds produced by microalgae, especially those of polyol nature (palytoxins, karlotoxins, gibbosols, prorocentroic acid, etc.) and will contribute to speed up the discovery of new analogues of marine polyketides directly from small volumes of cultures or even from concentrated field samples.

## Supporting Information Available

Isolation and characterization by NMR of luteophanol D; molecular networks, node refinement, and dereplication of known compounds; structural analysis from LC–full HRMS/dd-MS^2^ data analysis of known and new compounds identified in this study; results of the screening of amphidinols by searching diagnostic fragments and monitoring water loses in MS^2^ spectra; and chromatographic and spectrometric descriptions of all studied compounds (PDF)
