# Supplementary material for: Rapid Screening of Polyol Polyketides from Marine Dinoflagellates
Source: Anal Chem. 2022 Oct 3;94(41):14205–13. doi: 10.1021/acs.analchem.2c02185 (PMC9583072; doi:10.1021/acs.analchem.2c02185)
Supplement: Supplementary file 1 — ac2c02185_si_001.pdf [file ac2c02185_si_001.pdf]

SUPPORTING INFORMATION

A Rapid Screen for Polyol Polyketides from Marine Dinoflagellates

Adrián Morales-Amador,*[1,2] María L. Souto,[1] Christian Hertweck,[2,3] José J. Fernández,[1] María García-Altares*[2,4]

[1] Instituto Universitario de Bio-orgánica Antonio González (IUBO AG), Departamento de Química Orgánica, Universidad de La Laguna (ULL), Avenida Astrofísico Francisco Sánchez 2, 38206, La Laguna, Tenerife, Spain

[2] Department of Biomolecular Chemistry, Leibniz Institute for Natural Products Research and Infection Biology - Hans Knöll Institute (HKI), Adolf-Reichwein-Straße 23, 07745, Jena, Germany

[3] Faculty of Biological Sciences, Friedrich Schiller University Jena, 07743 Jena, Germany

[4] Department of Electronic Engineering, Rovira i Virgili University, 43007, Tarragona, Spain

Corresponding authors: amoralea@ull.edu.es; maria.garcia-altares@urv.cat

Table of Contents

S1. Full description of Materials and Methods 5

S2. Characterization of luteophanol D by NMR and LC-HRMS-MS^2 analysis 8

Scheme S1. Isolation of luteophanol D from ACRN03 culture. 8

Table S1. ^{1}H and ^{13}C NMR data for luteophanol D in CD_3OD. 9

Figure S1. ^{1}H NMR spectrum (600 MHz, 298 K, CD_3OD) for luteophanol D. 10

Figure S2. COSY spectrum (600 MHz, 298 K, CD_3OD) for luteophanol D**.** 11

Figure S3. HSQC spectrum (600 MHz, 298 K, CD_3OD) for luteophanol D. 12

Figure S4. HSQC-TOCSY spectrum (600 MHz, 298 K, CD_3OD) for luteophanol D. 13

Figure S5. H2BC spectrum (600 MHz, 298 K, CD_3OD) for luteophanol D. 14

Report S1 Identification of luteophanol D in ESI+ full HRMS. 15

Report S2. Identification of luteophanol D in ESI- full HRMS 15

Report S3. Annotation of MS^2 fragments of luteophanol D in ESI- mode at different percentages of HCD energy. 16

Report S4. Annotation of MS^2 fragments of luteophanol D in ESI+ mode at different percentages of HCD energy. 17

Table S2. Main precursor ions for luteophanol D in both ion scan modes. 18

Table S3. MS^2 fragments of luteophanol D in ESI- mode. 18

Table S4. MS^2 fragments of luteophanol D in ESI+ mode. 18

Figure S6. Alternative mechanisms of fragmentation observed in luteophanol D and analogs. 19

S3. Search for amphidinols in non-targeted LC-HRMS-MS^2 experiments 20

Table S5. Amphidinol-related analogs published hitherto. 20

RScript S1. R Script used for monitoring fragments and total water losses along MS^2 spectra on mzXML files. 22

Table S6. Precursor ions containing diagnostic MS^2 fragments in cell-free medium extracts. 25

Figure S7. Water loss count in MS^2 spectra. 26

Figure S8. Structures of luteophanol D and amphidinols 20B, 24 and 27-36. 27

Table S7. Description of the amphidinol analogs reported in this study. 28

Scheme S2. Structural relationships between the amphidinol analogs. 29

S4. Molecular networking (MN) analysis of non-targeted LC-MS^2 experiments. 30

Figure S9. MN of cell-free medium extract of **ACBR01** in ESI+ mode. 30

Figure S10. MN of cell-free medium extract of **ACBR01** in ESI- mode. 31

Figure S11. MN of biomass extract of **ACBR01** in ESI+ mode. 32

Figure S12. MN of cell-free medium extract of **ACRN02** in ESI+ mode. 33

Figure S13. MN of cell-free medium extract of **ACRN02** in ESI- mode. 34

Figure S14. MN of biomass extract of **ACRN02** in ESI+ mode. 35

Figure S15. MN of cell-free medium extract of **ACRN03** in ESI+ mode. 36

Figure S16. MN of cell-free medium extract of ACRN03 in ESI- mode. 37

Figure S17. MN of biomass extract of **ACRN03** in ESI+ mode. 38
Figure S18. MN of cell-free medium extract of **ACMK03** in ESI+ mode. 39
Figure S19. MN of cell-free medium extract of **ACMK03** in ESI- mode. 40
Figure S20. MN of biomass extract of **ACMK03** in ESI+ mode. 41
Figure S21. MN of all cell-free medium extracts combined in ESI+ mode. 42
Figure S22. Common family of secreted amphidinols in ESI+ mode. 43
Scheme S3. General workflow for the identification and structural study of amphidinols in this study. 45
S5. Identification of amphidinol-analogs and structural study **46**
Positive ion mode (ESI+) as $[M+H]^+$ ions **46**
Figure S23. Family of amphidinols in cell-free medium extract of **ACRB01**. 46
Table S8. Dereplication of potential amphidinols in **ACBR01**. 47
Figure S24. Family of amphidinols of cell-free medium extract of **ACRN02**. 48
Table S9. Dereplication of potential amphidinols in **ACRN02**. 48
Figure S25. EIC comparison between potential dehydrated protonated ions. 49
Figure S26 (a-d). MS^2 comparison between potential dehydrated protonated ions. 50
Report S5. Characterization of **luteophanol D** (*m/z* 1307.77) in **ACRN02**. 53
Report S6. Characterization of **amphidinol 28** (*m/z* 1323.77; RT 4.53) in **ACRN02**. 55
Report S7. Characterization of **amphidinol 20B** (*m/z* 1323.77; RT 5.15) in **ACRN02**. 58
Report S8. Characterization of **amphidinol 29** (*m/z* 1339.76) in **ACRN02.** 61
Report S9. Characterization of ion *m/z* 1321.76 (amphidinol 29) in **ACRN02**. 63
Report S10. Characterization of **amphidinol 27** (*m/z* 1185.66) in **ACRN02**. 65
Report S11. Characterization of ion *m/z* 1167.65 (amphidinol 27) in **ACRN02**. 68
Report S12. Characterization of **amphidinol 30** (*m/z* 1201.67) in **ACRN02**. 71
Report S13. Characterization of **amphidinol 31** (*m/z* 1219.67) in **ACRN02**. 74
Report S14. Characterization of **amphidinol 32** (*m/z* 1235.66) in **ACRN02**. 76
Report S15. Characterization of **amphidinol 33** (*m/z* 1101.60) in **ACRN02**. 78
Report S16. Characterization of **amphidinol 34** (*m/z* 1071.59) in **ACRN02.** 80
Report S17. Characterization of ion *m/z* 1285.67 in **ACRN02.** 82
Figure S27. Family of AMs in cell-free medium extract of **ACRN03.** 83
Table S10. Dereplication of potential amphidinols in ACRN03. 83
Report S18. Identification of **luteophanol D** (*m/z* 1307.77) in **ACRN03**. 84
Report S19. Identification of **amphidinol 28** (*m/z* 1323.77) in **ACRN03**. 85
Report S20. Identification of **amphidinol 29** (*m/z* 1339.76) in **ACRN03**. 86
Report S21. Identification of **amphidinol 27** (*m/z* 1185.66) in **ACRN03**. 87
Report S22. Identification of **amphidinol 30** (*m/z* 1201.65) in **ACRN03**. 88
Report S23. Identification of **amphidinol 31** (*m/z* 1219.66) in **ACRN03**. 89
Report S24. Characterization of **amphidinol 35** (*m/z* 1355.76) in **ACRN03**. 90

Report S25. Characterization of **amphidinol 36** (*m/z* 1357.76) in **ACRN03**. 93
Table S11. Assignment of the internal fragments contained in MS2 spectra of AMs 28-35 to relevant cleavages (clv). 95
Negative ion mode (ESI–, HCD 22%) as [M+HCOO]⁻ ions **96**
Figure S28. Family of amphidinols in cell-free medium extract of **ACRN02** 96
Report S26. Characterization of **luteophanol D** (*m/z* 1351.77) in **ACRN02** 97
Report S27. Characterization of **amphidinol 28** (*m/z* 1367.76; RT 4.54) in **ACRN02** 99
Report S28. Characterization of **amphidinol 20B** (*m/z* 1367.76, RT 5.07) in **ACRN02** 101
Report S29. Characterization of **amphidinol 29** (*m/z* 1383.75) in **ACRN02** 103
Report S30. Characterization of **amphidinol 24** (*m/z* 1385.77) in **ACRN02.** 105
Figure S29. Family of amphidinols in cell-free medium extract of **ACRN03.** 107
Report S31. Identification of **amphidinol 28** (*m/z* 1367.76) in **ACRN03**. 108
Report S32. Identification of **amphidinol 29** (*m/z* 1383.76) in **ACRN03**. 109
Report S33. Identification of **amphidinol 24** (*m/z* 1385.77) in **ACRN03**. 110
Report S34. Characterization of **amphidinol 36** (*m/z* 1401.76) in **ACRN03**. 111
Figure S30. Common fragment ions found in luteophanol D, amphidinol 24 and amphidinols 27-36. 113
Figure S31. Fragment ions of lutophanol D. 114
Figure S32. Fragment ions of amphidinol 28. 114
Figure S33. Fragment ions of amphidinol 20B. 115
Figure S34. Fragment ions of amphidinol 29. 115
Figure S35. Fragment ions of amphidinol 27. 116
Figure S36. Fragment ions of amphidinol 30. 116
Figure S37. Fragment ions of amphidinol 31. 117
Figure S38. Fragment ions of amphidinol 32. 117
Figure S39. Fragment ions of amphidinol 33. 118
Figure S40. Fragment ions of amphidinol 34. 118
Figure S41. Fragment ions of amphidinol 35. 119

S1. Full description of Materials and Methods

General methods. Preparative HPLC was performed on Waters' system equipped with a Binary HPLC Pump 1525 and Photodiode Array Detector 2996. Reagents and solvents (CHROMASOLV®) were purchased in Sigma Aldrich. NMR experiments were carried out on a Bruker Avance 600 instrument (Karlsruhe, Germany) equipped with a 5-mm TCI (Triple Resonance CryoProbe) inverse detection cryo-probe. ^1H and ^{13}C NMR chemical shifts were referenced to the CD_3OD peak at 300 K (3.31 and 49.0 ppm). All experiments were performed in the phase-sensitive mode (States TPPI (Time Proportional Phase-Incrementation frequency discrimination) or echo-antiecho for quadrature detection in F1) and used gradient coherence selection. ESI-HRMS experiments were performed in a LCT Premier XE Micromass spectrometer (Waters, USA). LC-ESI-HRMS experiments were carried out in a Q-Exactive Orbitrap® mass spectrometer equipped with an Accela AS LC system (Thermo Fischer, USA) with an Accucore C18 column (2.6 μm, 100 x 2.1 mm; Thermo Fischer, USA). MS^2 averaged spectra of precursor ions were obtained by mMass v5.5.0 (01.07.2013) with a tolerance of 15.0 ppm. NMR, LC-MS data and MS^2 spectra were visualized and analyzed in MestReNova v14.2.0-26256 (25.09.2020). R analysis and representations were run on RStudio v1.3.1093 (28.11.2020), and all the packages updated (December 2020).

Microalgae culture. *A. carterae* strans were isolated from the Brazilian coast (ACBR01), Réunion Island (ACRN02 and ACRN03) and Mauritius Island (ACMK03). They were kept in maintenance conditions as cultures of 125 mL stored in flasks of 250 mL, containing sterile modified Guillard K medium (described below). Cultures were incubated at 21±2 °C with a light intensity of 60 $\mu E\cdot s^{-1}\cdot m^{-2}$ with a light:darkness cycle of 18:6 hours. For the study, 2 L cultures were prepared on 5 L sterile flasks with 250 mL of inoculum in exponential growing phase and 1.75 L of fresh supplemented water. Cultures were kept for 50 days to induce a nutrient depletion stress. Composition of modified Guillard K medium: $NaNO_3$ (882 μM), NH_4Cl (50 μM), NaH_2PO_4 (10 μM), TRIS (1 mM), $Na_2EDTA\cdot 2H_2O$ (90 μM), $FeEDTA\cdot 3H_2O$ (14.6 μM), $MnCl_2\cdot 4H_2O$ (0.9 μM), $ZnSO_4\cdot 7H_2O$ (0.08 μM), $CoCl_2\cdot 6H_2O$ (0.05 μM), $Na_2MoO_4\cdot 2H_2O$ (0.03 μM), H_2SeO_3 (0.01 μM), thiamine-HCl (0.3 μM), biotin (2.1 nM), and B12 (0.37 nM)] as described in Keller, M.D.; Selvin, R.C.; Claus, W.; Guillard, R.R.L. Media for the Culture of Oceanic Ultraphytoplankton. *J. Phycol.*, **1987**, *23*, 633-638.

Cell-free medium and biomass extraction. *A. carterae* cultures were centrifuged (6000 rpm, 12 min, SORVALL RC 6+, ThermoFischer) and supernatants filtered with borosilicate filters GMFA ø 0.47 mm (Scharlau) using Glassco Vacuum filtration system. Pelleted cells were frozen, lyophilized and extracted with 3 portions of 10 mL methanol assisted by bath ultrasonication for 10 min at room temperature. The extraction of the organic content in cell-free media was done by solid-phase extraction (SPE) using C18 prepacked cartridges (SVF D22 RP18 25-40 μm, 17g, Merck Chimie SAS) and an ISMATEC® Rotary Piston Pump (Cole Parmer GmbH). Cartridges were washed with water to remove salts, and compounds were desorbed using methanol. Solvents were removed by rotary evaporation.

Isolation and purification of luteophanol D. Luteophanol D was isolated from the cell-free medium of ACRN03 batch culture to be used as a self-made analytical reference as described in **Scheme S1**. Luteophanol D was structurally characterized by NMR analysis (**Table S1**, **Figures S1**-**S5**), and ESI-HRMS as a monoisotopic peak at *m/z* 1329.7506 $[M+Na]^+$ (theoretical *m/z* 1329.7546 for $C_{66}H_{114}O_{25}Na^+$) [8,30].

Liquid chromatography-high resolution mass spectrometry experiments. Luteophanol D was analyzed by LC-ESI-HRMS-HCD-MS^2 in the Q-Exactive Orbitrap® mass spectrometer to set the chromatographic and spectrometric parameters, as well as to characterize and annotate its fragmentation pattern. ESI full HRMS spectra were acquired for the range of *m/z* 500-2000. For all samples, over 8000 full HRMS spectra were generated. After evaluating the full HRMS for both ionization modes, $[M+H]^+$ and $[M–H]^-$ ions were selected to be fragmented by applying 10, 15, 22, and 30 % of higher-energy collisional dissociation (HCD) for ESI+ mode and 10, 15, 22, 30, 35 and 42 % HCD for ESI– mode to get the most informative fragmentation, that were set up in 12 and 22 % HCD, for positive and negative mode respectively. Afterward, luteophanol D and the eight extracts of *A. carterae* were analyzed under data-dependent (dd) acquisition scan mode (LC-ESI-Full HRMS/dd-MS^2) with a Top 5 set up, so for every 2 scan events the 1st scan acquired the Full HRMS experiment whereas the 2nd scan event performed HCD-HRMS^2 acquisition, fragmenting the 5 most intense ions observed. All the extracts were solved with methanol (MeOH) to obtain 100 μL aliquots at 2 $mg\cdot mL^{-1}$, and luteophanol D sample at 1 $mg\cdot mL^{-1}$. The spectrometer was coupled to an Accela AS LC system (Thermo Fischer, USA) with an Accucore C18 column (2.6 μm, 100 x 2.1 mm; Thermo Fischer, USA). Flow rate was established at 0.2 $mL\cdot min^{-1}$ with water (eluent A) and acetonitrile (eluent B) both containing 0.1% of formic acid. Gradient elution used was: 5-98% B over 10 min, hold 12 min, 98 to 5% of B in 0.1 min, and hold 6.9 min, and injection volume was 10 μL and the oven temperature was 30 ^oC. Source settings of the Full HRMS experiments for both ionization modes were: spray voltage 3.5 V; capillary temperature 250 ^oC; capillary voltage 50 V; sheath glass flow 49.0

and auxiliary gas flow 5 (arbitrary units); tube lens voltage 130 V; and resolving power as set at 70,000 (FWHM at *m/z* 400).

Water losses analysis. The occurrence of dehydration events observed in positive ionization mode counted by inspecting MS^2 spectra and considering all signals with Δ*m/z* 18. For this purpose, a script was developed in R language (**RSript S1**).

Molecular networks on *Amphidinium carterae*. Raw files were exported to universal readable mzXML format, using MSConvert from Proteowizard https://proteowizard.sourceforge.net. Global Natural Product Social Media (GNPS) platform (https://gnps.ucsd.edu) was used to analyze mzXML files. Molecular networking (MN) was performed using its online workflow. For every analysis, data were filtered by removing all MS^2 peaks within ±17 Da of the precursor *m/z*. MS^2 spectra were filtered by choosing the Top 6 peaks in the ±50 Da window throughout the spectrum. Data were clustered with MS-Cluster with a parent mass tolerance of 0.1 Da and a MS^2 fragment ion tolerance of 0.1 Da, to create a consensus spectrum. Further, just consensus spectrum formed from at least 2 MS^2 spectra were kept. A network was created, and the edges were filtered to have a cosine score above 0.7 and more than 6 matched peaks. The edges between two nodes were kept only if they appeared in their Top10 most similar nodes.

Table 1. Description of MS[2] fragments of luteophanol D for both ESI modes. RDB = Rings and Double Bond Equivalents; Δppm = error between experimental and theoretical mass in ppm.

Cleavage	Ion/radical	Formula	*m/z* theo.	*m/z* exp.	RDB	Δppm
a	$[X+H\text{-}4H_2O]^+$	$C_{56}H_{87}O_{17}^+$	1031.5938	1031.5977	13.5	3.83
b	$[X–H]^–$	$C_{44}H_{77}O_{17}^–$	877.5166	877.5182	6.5	1.82
c	$[X+H–3H_2O]^+$	$C_{44}H_{75}O_{14}^+$	827.5151	827.5181	7.5	3.59
d	$[X–H]^–$	$C_{41}H_{65}O_{16}^–$	813.4273	813.4291	9.5	1.60
e	$[X–H]^–$	$C_{32}H_{57}O_{11}^–$	617.3906	617.3912	4.5	0.97
f	$[X+H–4H_2O]^+$	$C_{34}H_{57}O_9^+$	609.3997	609.4022	6.5	4.14
g	$[X+H–4H_2O]^+$	$C_{30}H_{47}O_6^+$	503.3367	503.3388	7.5	4.23
h	$[X–H]^–$	$C_{20}H_{35}O_7^–$	387.2388	387.2396	3.5	2.07
j	$[X+H–3H_2O]^+$	$C_{20}H_{31}O_4^+$	335.2222	335.2228	5.5	1.79
k	$[X+H–H_2O]^+$	$C_{10}H_{19}O_3^+$	187.1329	187.1336	1.5	3.81
m	$[X–H_2O]^·$	$C_7H_8^·$	93.0704	93.0708	4.0	4.29

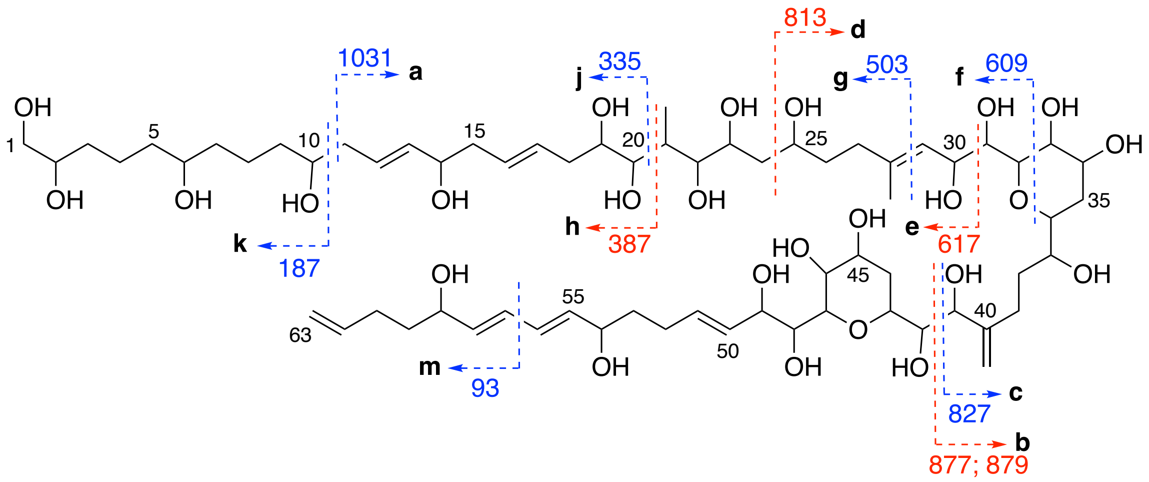

S2. Characterization of luteophanol D by NMR and LC-HRMS-MS² analysis

Scheme S1. Isolation of luteophanol D from ACRN03 culture.

Luteophanol D (LTD) was isolated from ACRN03 strain to be used as self-made analytical reference to set up chromatographic, ionization and fragmentation parameters. The compound was purified from the cell-free medium extract of a batch culture of ACRN03. The extract was subjected to size-exclusion filtration on Sephadex LH-20® (27.5 x 6.5 cm) eluted with methanol (flow rate of 12 mL·min^{-1}). The fraction containing luteophanol D was chromatographed in reverse phase by medium pressure liquid chromatography using Lobar LiChroprep RP18 column, and a gradient of water:acetonitrile:methanol from 7:2:1 to 0:1:0 (flow rate of 3 mL·min^{-1}, 52 min). The final purification was performed on HPLC with μ-Bondapack® C18 (10 μm, 19 x 190 mm. Waters, USA) column and a methanol:water gradient from 1:1 to 8:2 (flow rate of 1 mL·min^{-1}, 40 min) to obtain luteophanol D (4.6 mg, RT 16.0 min) as a pale-yellow amorphous solid.

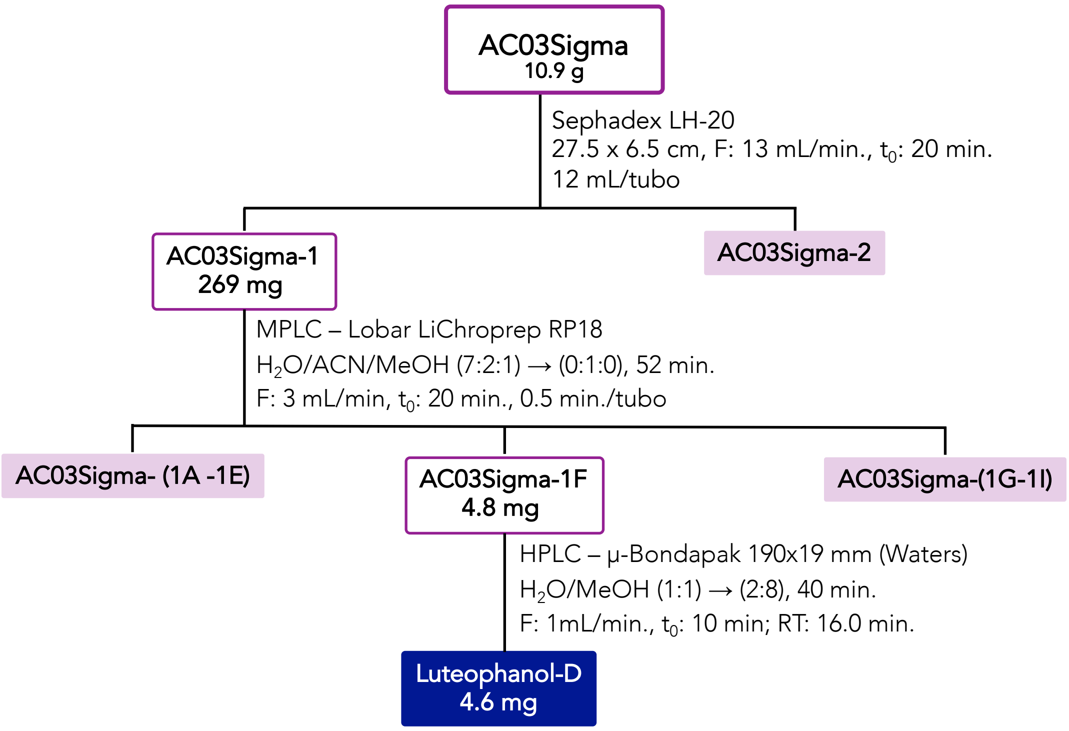

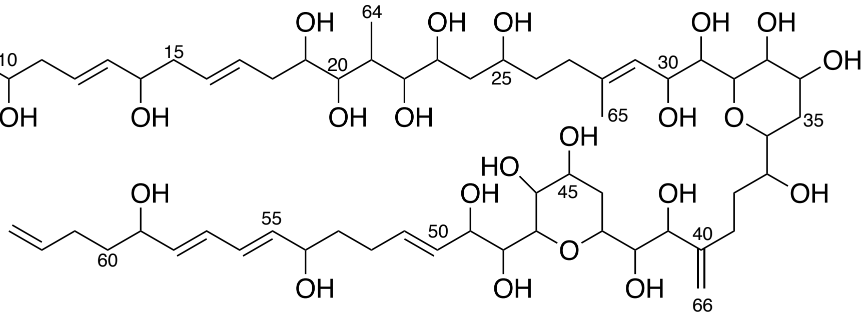

Luteophanol D

Chemical Formula: $C_{66}H_{114}O_{25}$
Exact Mass: 1306.7649

Table S1. ^{1}H and ^{13}C NMR data for luteophanol D in CD_3OD.

N°	C-type	δ_C (ppm)	δ_H (ppm)		N°	C-type	δ_C (ppm)	δ_H (ppm)	
**1**	CH_2	67.1	3.43		**31**	CH	72.2	3.68	
			3.48		**32**	CH	78.9	3.96	
**2**	CH	72.9	3.52		**33**	CH	68.5	4.05	
**3**	CH_2	34.2	1.38		**34**	CH	68.3	4.04	
			1.54		**35**	CH_2	30.0	1.79	(2H)
**4**	CH_2	22.7	1.39		**36**	CH	75.3	3.49	
			1.62		**37**	CH	74.2	3.60	
**5**	CH_2	38.1	1.41		**38**	CH_2	32.2	1.57	
			1.51					1.97	
**6**	CH	72.0	3.55		**39**	CH_2	27.7	2.11	
**7**	CH_2	38.1	1.41					2.42	
			1.51		**40**	C	151.2	-	
**8**	CH_2	22.7	1.39		**41**	CH	76.3	4.19	
			1.62		**42**	CH	74.9	3.36	
**9**	CH_2	37.5	1.40		**43**	CH	70.1	4.04	
			1.51		**44**	CH_2	31.3	1.57	
**10**	CH	72.3	3.59					2.10	
**11**	CH_2	41.1	2.20	(2H)	**45**	CH	67.1	4.05	
**12**	CH	128.6	5.68		**46**	CH	68.5	4.05	
**13**	CH	135.9	5.53		**47**	CH	80.1	3.74	
**14**	CH	73.2	4.05		**48**	CH	71.6	3.97	
**15**	CH_2	41.7	2.25	(2H)	**49**	CH	73.8	4.37	
**16**	CH	129.6	5.54		**50**	CH	128.6	5.65	
**17**	CH	130.0	5.61		**51**	CH	134.9	5.80	
**18**	CH_2	37.7	2.09		**52**	CH_2	29.5	2.16	(2H)
			2.48		**53**	CH_2	33.3	2.20	(2H)
**19**	CH	72.2	3.52		**54**	CH	72.2	4.10	
**20**	CH	78.9	3.52		**55**	CH	136.6	5.68	
**21**	CH	35.0	2.31		**56**	CH	130.6	6.23	
**22**	CH	79.5	3.52		**57**	CH	130.6	6.23	
**23**	CH	71.9	3.71		**58**	CH	136.6	5.68	
**24**	CH_2	40.8	1.54		**59**	CH	72.2	4.10	
			1.91		**60**	CH_2	37.5	1.62	(2H)
**25**	CH	71.1	3.86		**61**	CH_2	30.7	2.11	(2H)
**26**	CH_2	36.3	1.59		**62**	CH	139.1	5.84	
			1.68		**63**	CH_2	114.7	4.95	
**27**	CH_2	36.5	2.12					5.02	
			2.22		**64**	CH_3	6.6	0.97	
**28**	C	139.1	-		**65**	CH_3	17.1	1.75	
**29**	CH	125.9	5.48		**66**	CH_2	112.8	4.99	
**30**	CH	67.5	4.56					5.08	

Figure S1. ^{1}H NMR spectrum (600 MHz, 298 K, CD_3OD) for luteophanol D.

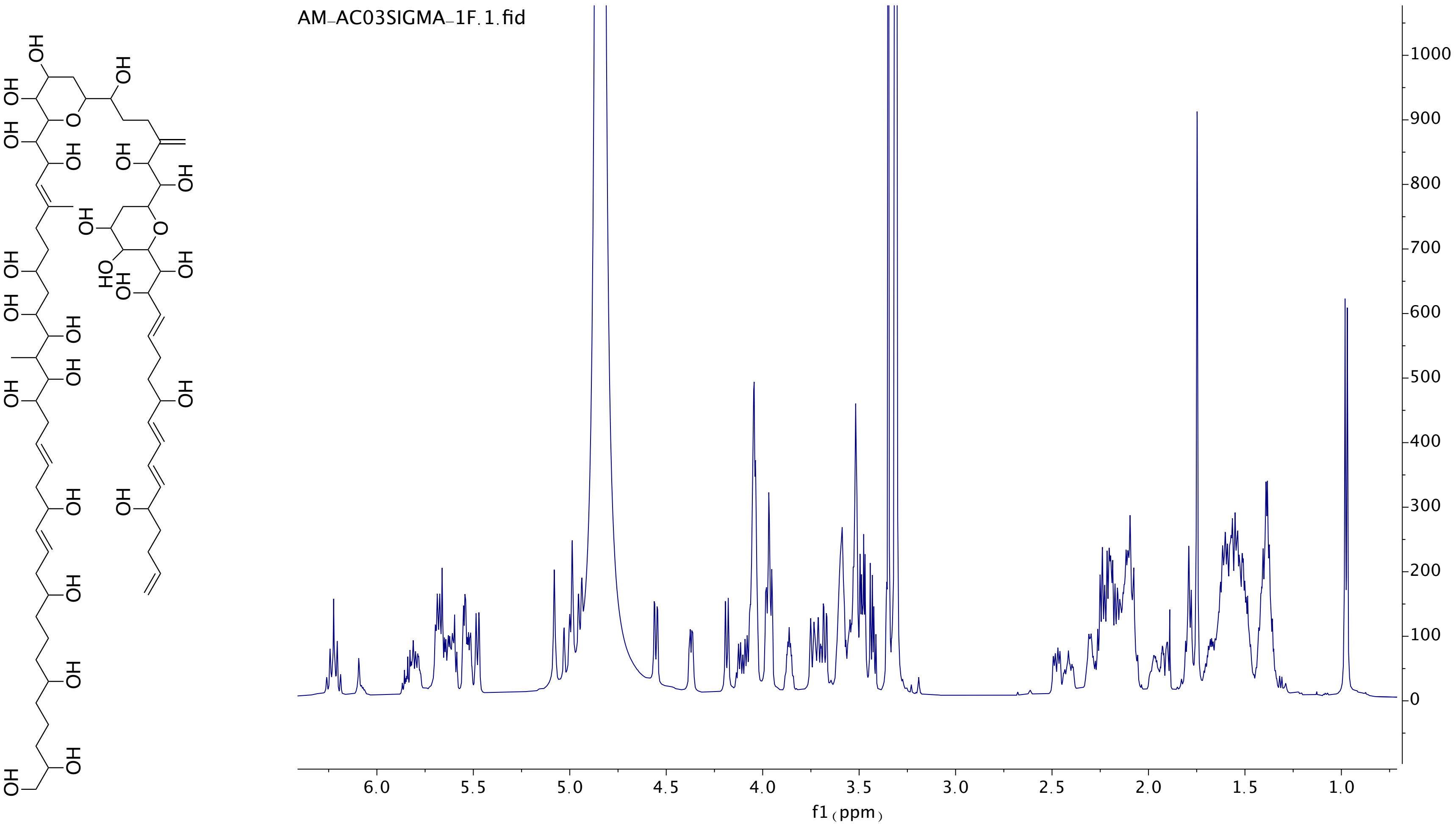

Figure S2. COSY spectrum (600 MHz, 298 K, CD_3OD) for luteophanol D**.**

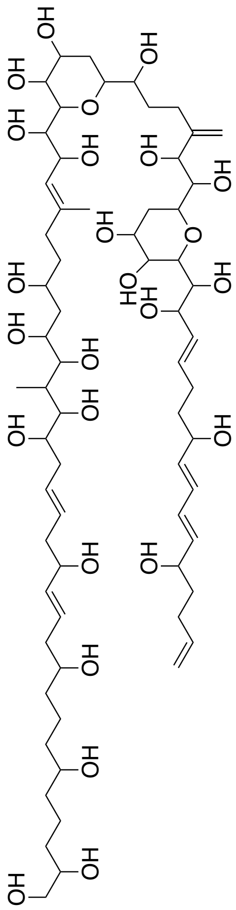

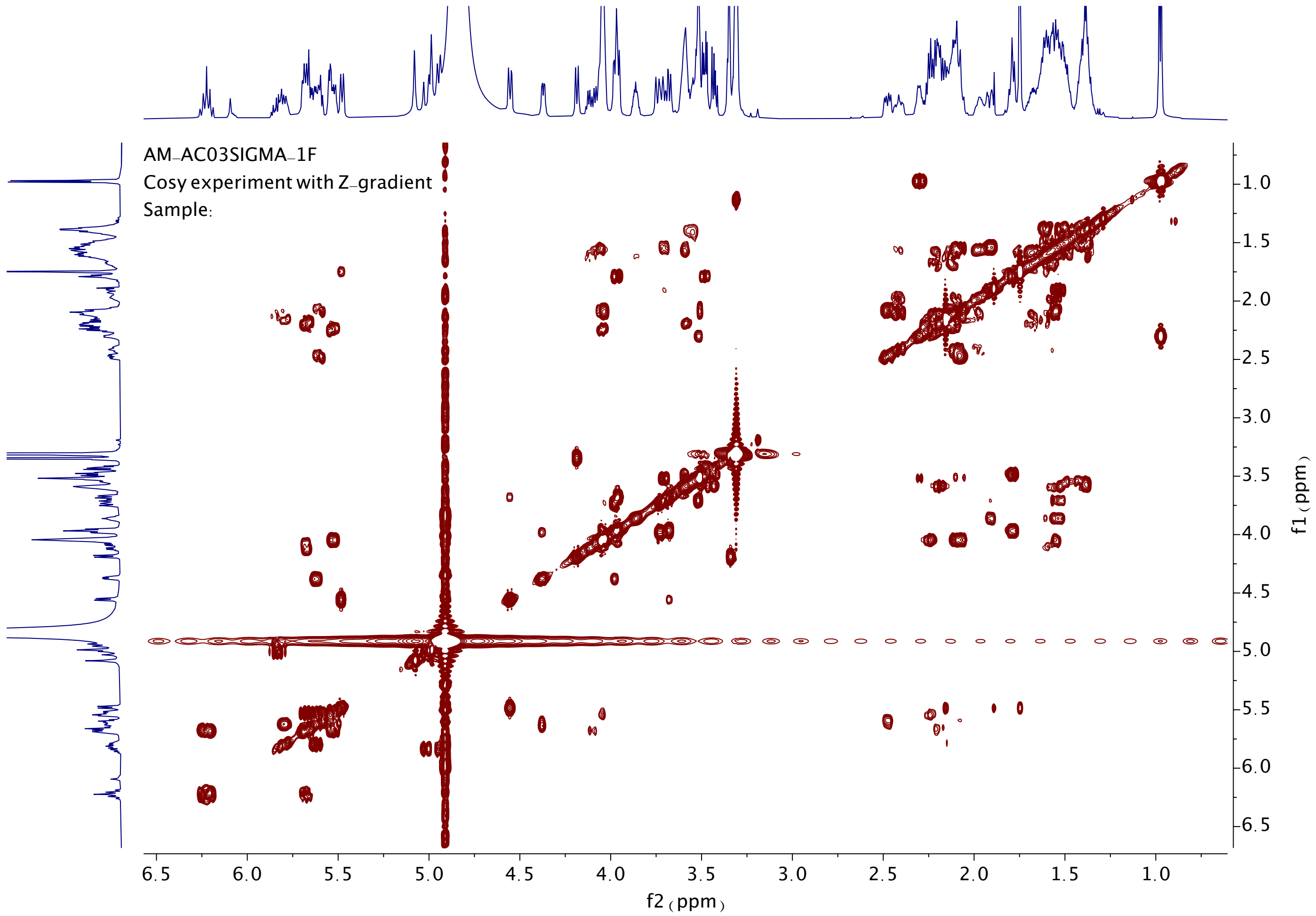

Figure S3. HSQC spectrum (600 MHz, 298 K, CD_3OD) for luteophanol D.

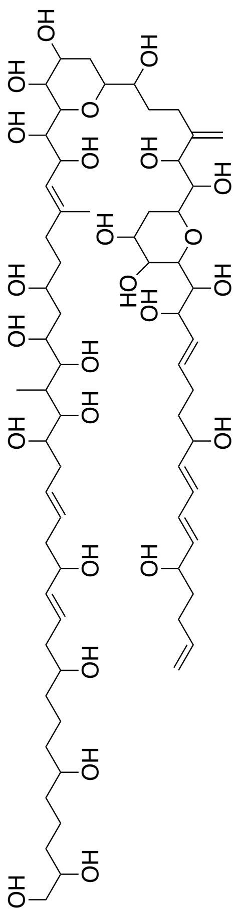

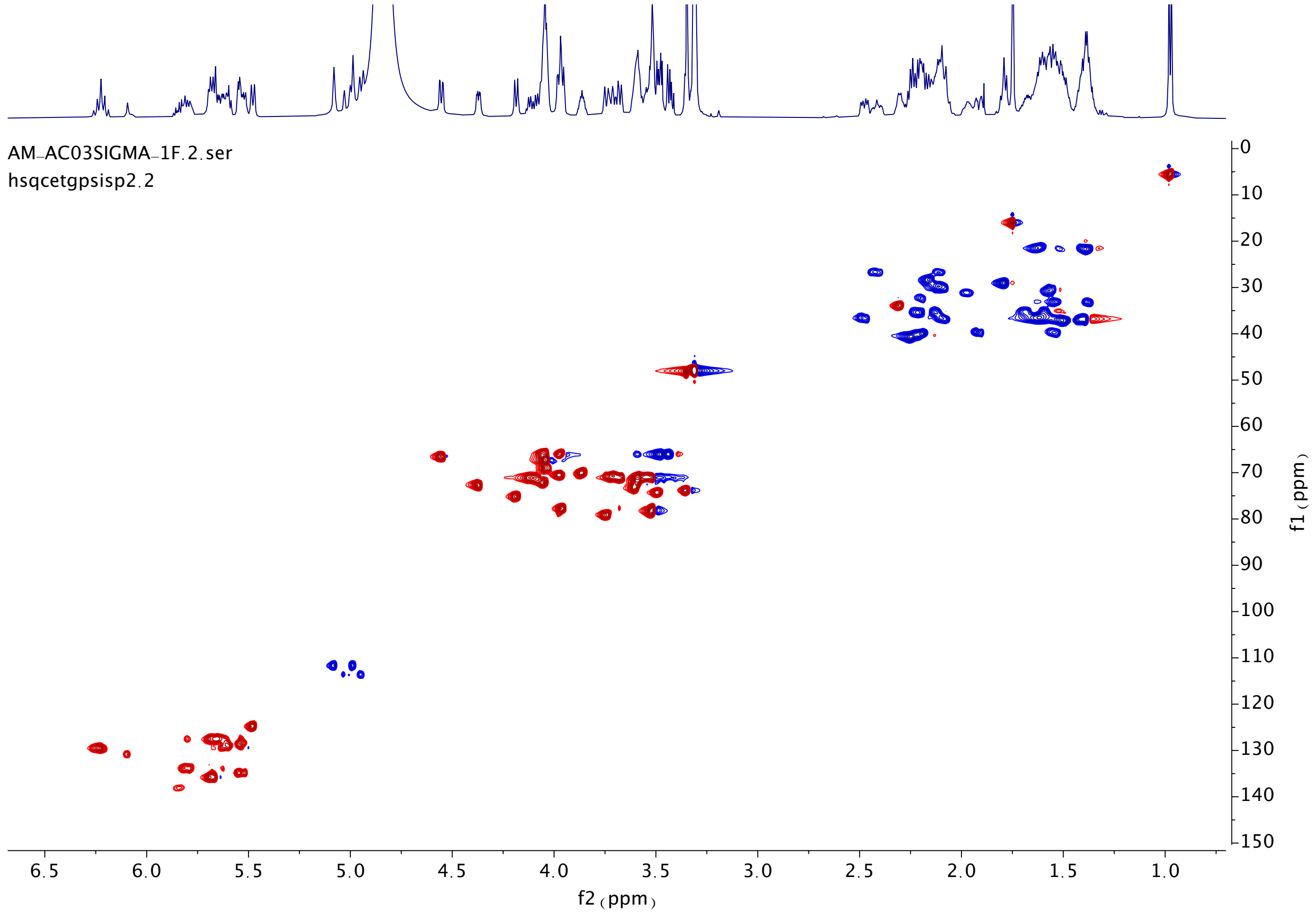

Figure S4. HSQC-TOCSY spectrum (600 MHz, 298 K, CD_3OD) for luteophanol D.

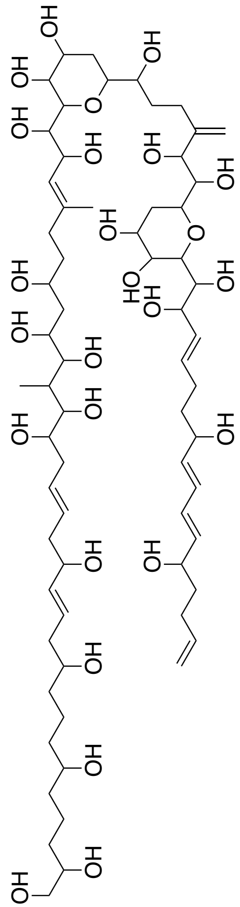

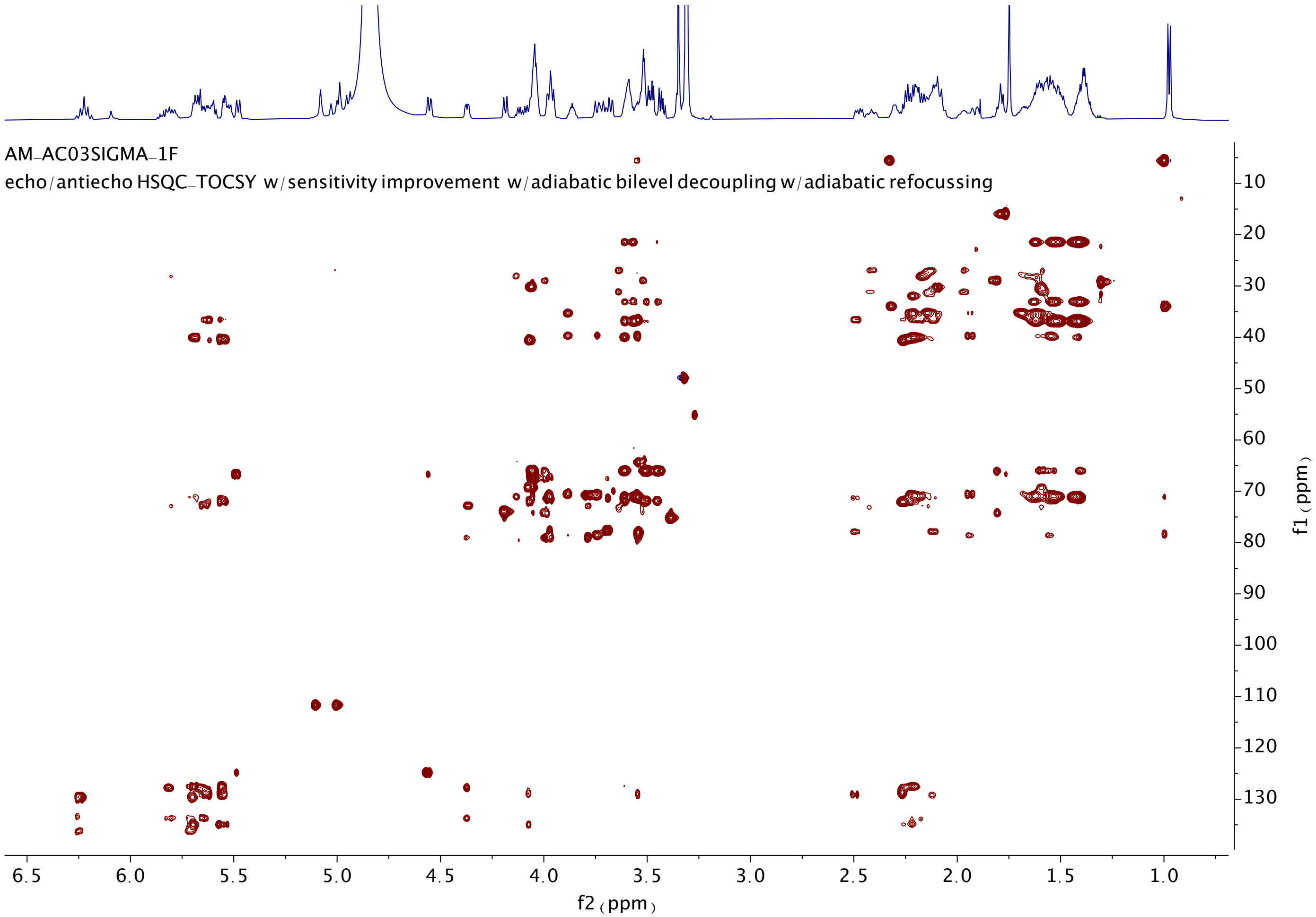

Figure S5. H2BC spectrum (600 MHz, 298 K, CD_3OD) for luteophanol D.

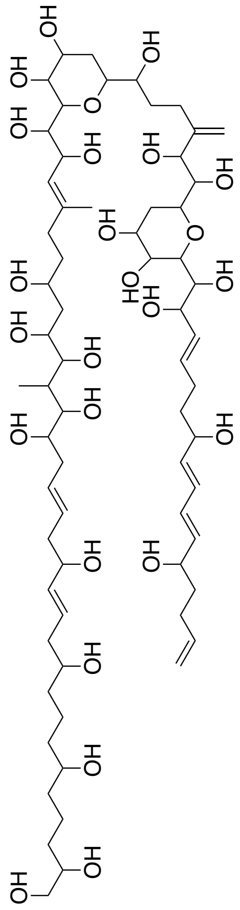

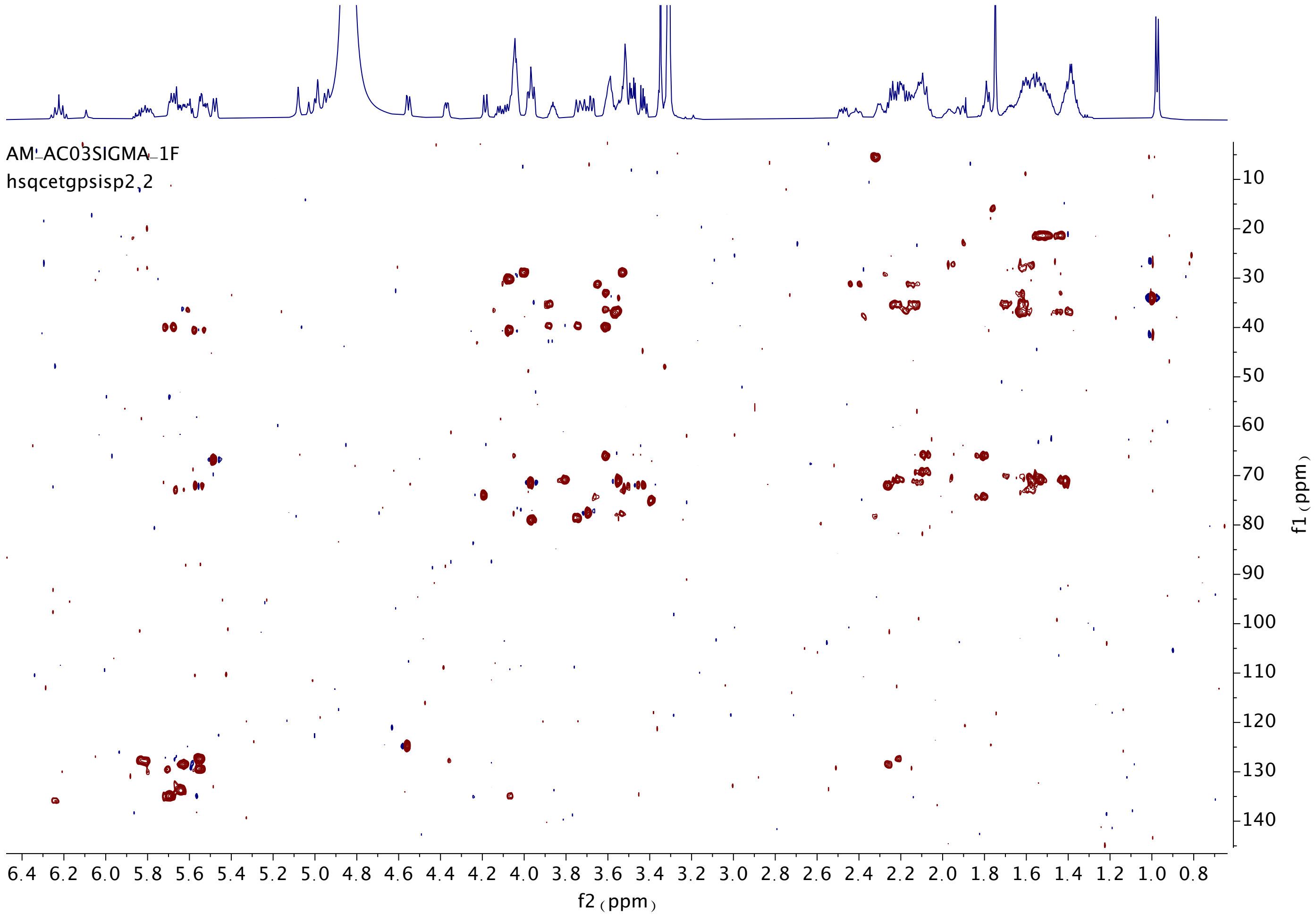

Report S1 Identification of luteophanol D in ESI+ full HRMS.

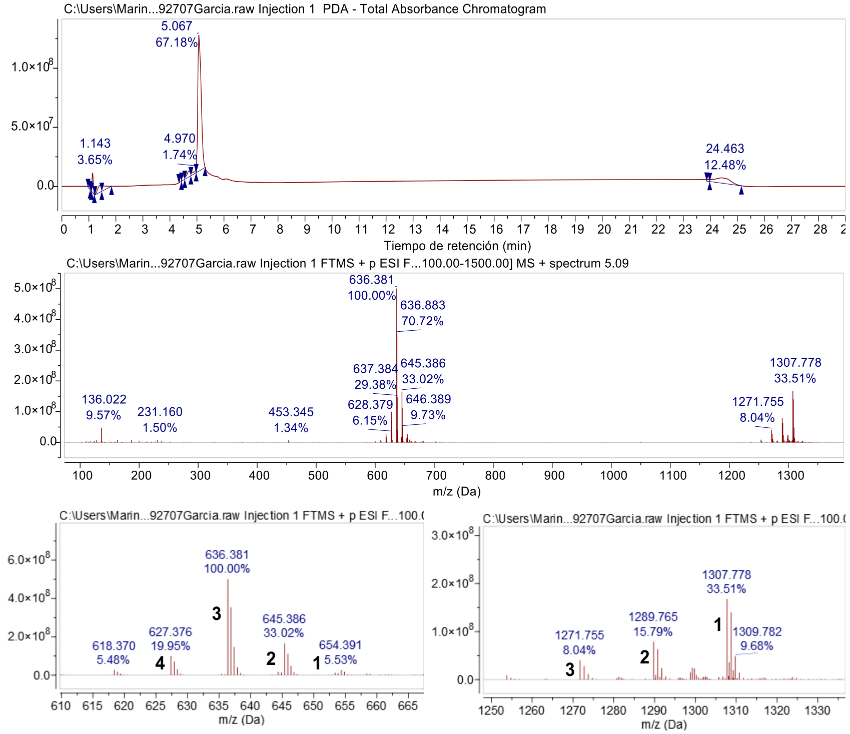

	Ion	Formula	*m/z* theo.	*m/z* exp.	RDB	Δ Da	Δ ppm
1	$[M+H]^+$	$C_{66}H_{115}O_{25}^+$	1307.7722	1307.7775	9.5	5.33	4.07
2	$[M+H–H_2O]^+$	$C_{66}H_{113}O_{24}^+$	1289.7616	1289.7655	10.5	3.84	2.98
3	$[M+H–2H_2O]^+$	$C_{66}H_{111}O_{23}^+$	1271.7511	1271.7550	11.5	3.97	3.12

***z* = 2**

	Ion	Formula	*m/z* theo.	*m/z* exp.	RDB	Δ Da	Δ ppm
1	$[M+2H]^{2+}$	$C_{66}H_{116}O_{25}^{2+}$	654.3898	654.3908	9.0	1.11	1.69
2	$[M+2H–H_2O]^{2+}$	$C_{66}H_{114}O_{24}^{2+}$	645.3844	645.3859	10.0	1.45	2.26
3	$[M+2H–2H_2O]^{2+}$	$C_{66}H_{112}O_{23}^{2+}$	636.3792	636.3813	11.0	2.14	3.36
4	$[M+2H–3H_2O]^{2+}$	$C_{66}H_{110}O_{22}^{2+}$	627.3739	627.3757	12.0	1.79	2.86

Report S2. Identification of luteophanol D in ESI- full HRMS

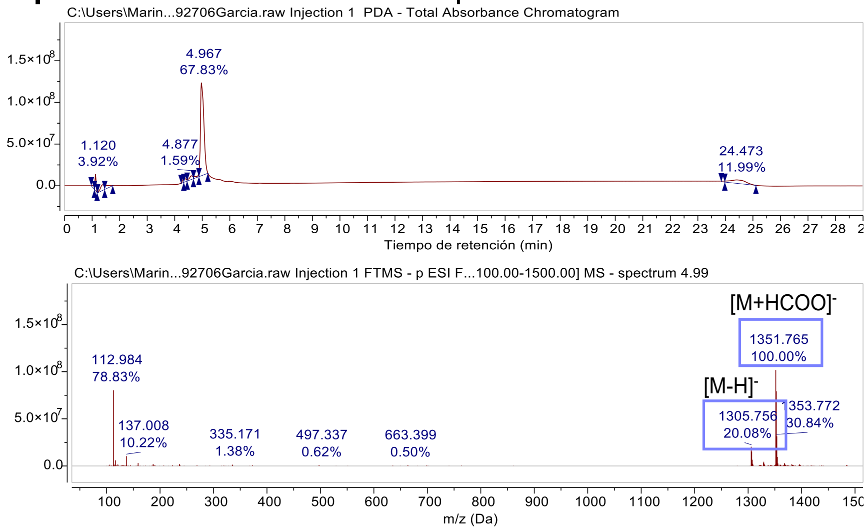

	Ion	Formula	*m/z* theo.	*m/z* exp.	RDB	Δ Da	Δ ppm
1	$[M+HCOO]^–$	$C_{67}H_{115}O_{27}^–$	1351.7626	1351.7642	10.5	1.60	1.63
2	$[M–H]^–$	$C_{66}H_{113}O_{25}^–$	1305.7571	1305.7563	10.5	–0.83	–0.63

Report S3. Annotation of MS^2 fragments of luteophanol D in ESI- mode at different percentages of HCD energy.

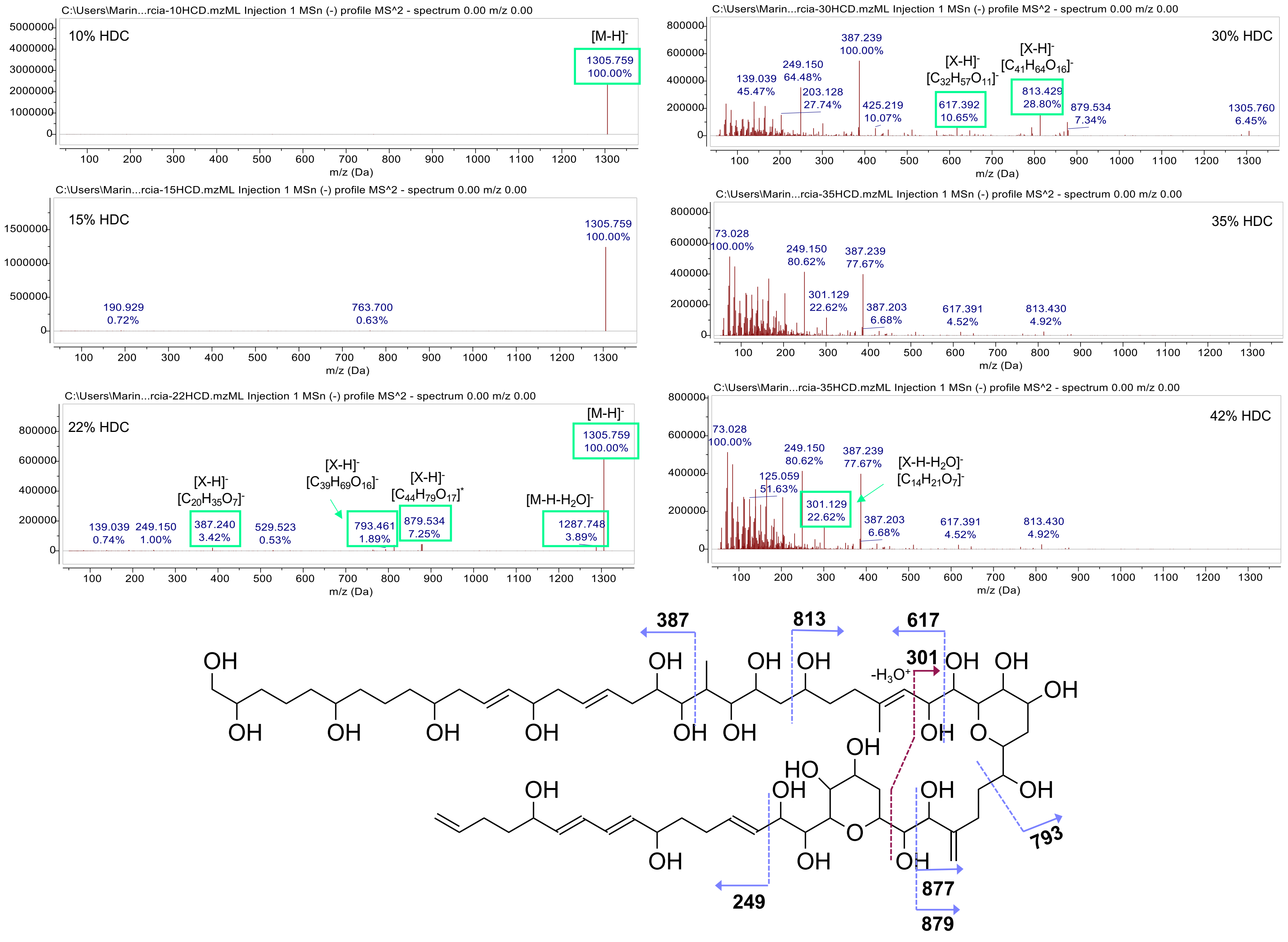

Report S4. Annotation of MS² fragments of luteophanol D in ESI+ mode at different percentages of HCD energy.

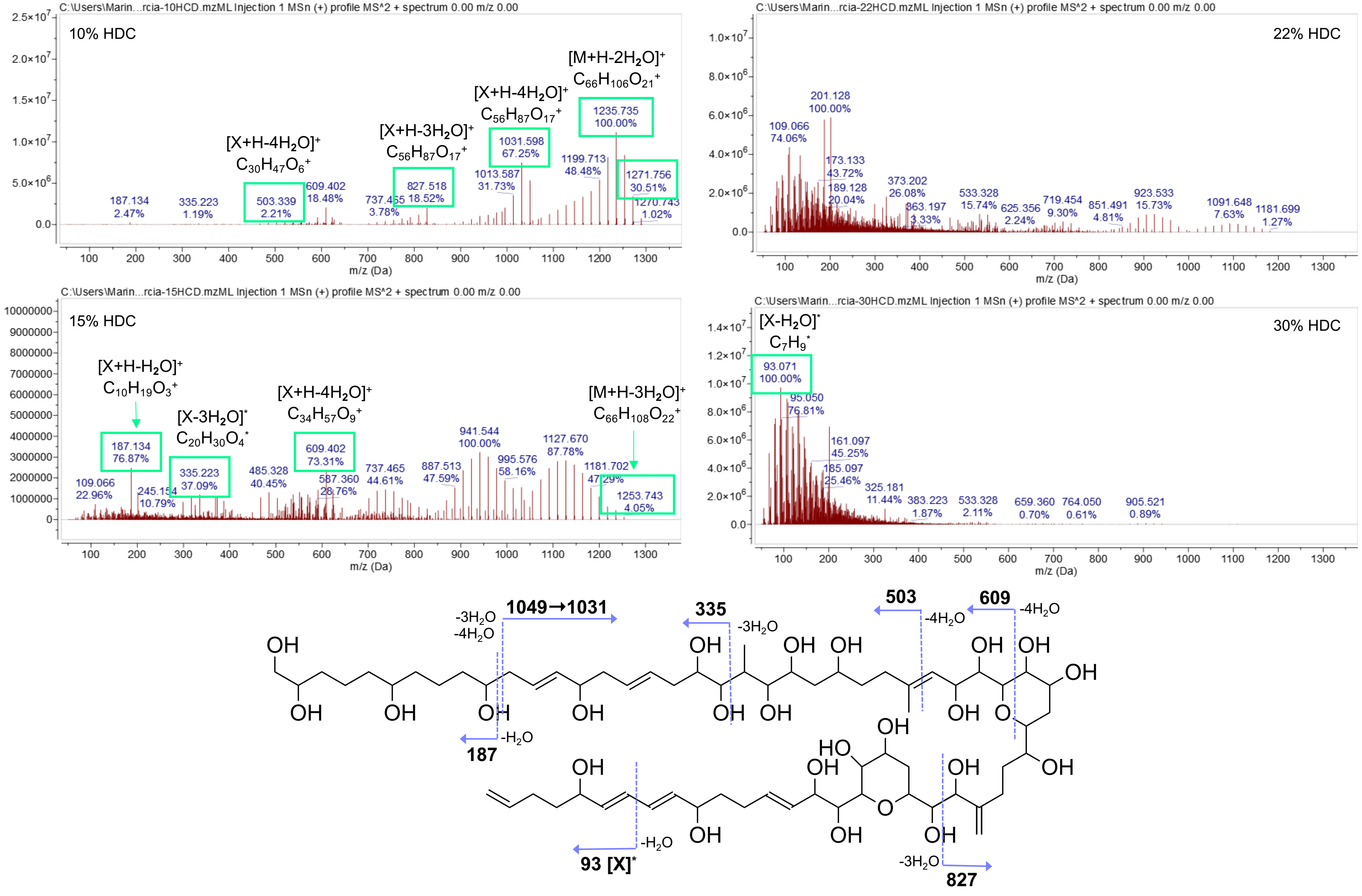

Table S2. Main precursor ions for luteophanol D in both ion scan modes.

Ion	Formula	*m/z* theo.	*m/z* exp.	RDB	Δppm
$[M+H]^+$	$C_{66}H_{115}O_{25}^+$	1307.7722	1307.7775	10.0	4.07
$[M+H–H2O]^+$	$C_{66}H_{113}O_{24}^+$	1289.7616	1289.7655	11.0	2.98
$[M–H]^–$	$C_{66}H_{113}O_{25}^–$	1305.7571	1305.7563	10.5	–0.63
$[M+HCOO]^–$	$C_{67}H_{114}O_{27}^–$	1351.7626	1351.7642	10.5	1.63

Table S3. MS[2] fragments of luteophanol D in ESI- mode.

	Ion	Formula	*m/z* theo.	*m/z* exp.	RDB	Δ ppm
1	$[M-H]^–$	$C_{66}H_{113}O_{25}^–$	1305.7571	1305.7588	10.5	1.32
2	$[M-H-H_2O]^–$	$C_{66}H_{111}O_{24}^–$	1287.7465	1287.7478	11.5	0.97
3	$[X-H]^–$	$C_{44}H_{79}O_{17}^–$	879.5317	879.5339	5.5	2.47
4	$[X-H]^–$	$C_{44}H_{77}O_{17}^–$	877.5166	877.5182	6.5	1.82
5	$[X-H]^–$	$C_{41}H_{65}O_{16}^–$	813.4273	813.4291	9.5	1.60
6	$[X-H]^–$	$C_{39}H_{69}O_{16}^–$	793.4591	793.4610	5.5	2.39
7	$[X-H]^–$	$C_{32}H_{57}O_{11}^–$	617.3906	617.3912	4.5	0.97
8	$[X-H]^–$	$C_{20}H_{35}O_{7}^–$	387.2388	387.2396	3.5	2.07

Table S4. MS[2] fragments of luteophanol D in ESI+ mode.

	Ion/radical	Formula	*m/z* theo.	*m/z* exp.	RDB	Δ ppm	Water losses
1	$[M+H-2H_2O]^+$	$C_{66}H_{111}O_{23}^+$	1271.7511	1271.7559	11.5	3.79	15
2	$[M+H-4H_2O]^+$	$C_{66}H_{107}O_{21}^+$	1235.7299	1235.7346	13.5	3.78	13
3	$[X+H-4H_2O]^+$	$C_{56}H_{87}O_{17}^+$	1031.5938	1031.5977	13.5	3.83	10
4	$[X+H-3H_2O]^+$	$C_{44}H_{75}O_{14}^+$	827.5151	827.5181	7.5	3.59	8
5	$[X+H-4H_2O]^+$	$C_{34}H_{57}O_{9}^+$	609.3997	609.4022	6.5	4.14	6
6	$[X+H-4H_2O]^+$	$C_{30}H_{49}O_{6}^+$	503.3367	503.3388	7.5	4.23	3
7	$[X+H-5H_2O]^+$	$C_{24}H_{35}O_{4}^+$	387.2529	387.2545	7.5	3.98	1
8	$[X+H-3H_2O]^+$	$C_{20}H_{31}O_{4}^+$	335.2222	335.2228	5.5	1.79	3
9	$[X+H-H_2O]^+$	$C_{10}H_{19}O_{3}^+$	187.1329	187.1336	1.5	3.81	1
10	$[X]^•$	$C_7H_9^•$	93.0704	93.0708	3.5	4.29	-

Figure S6. Alternative mechanisms of fragmentation observed in luteophanol D and analogs.

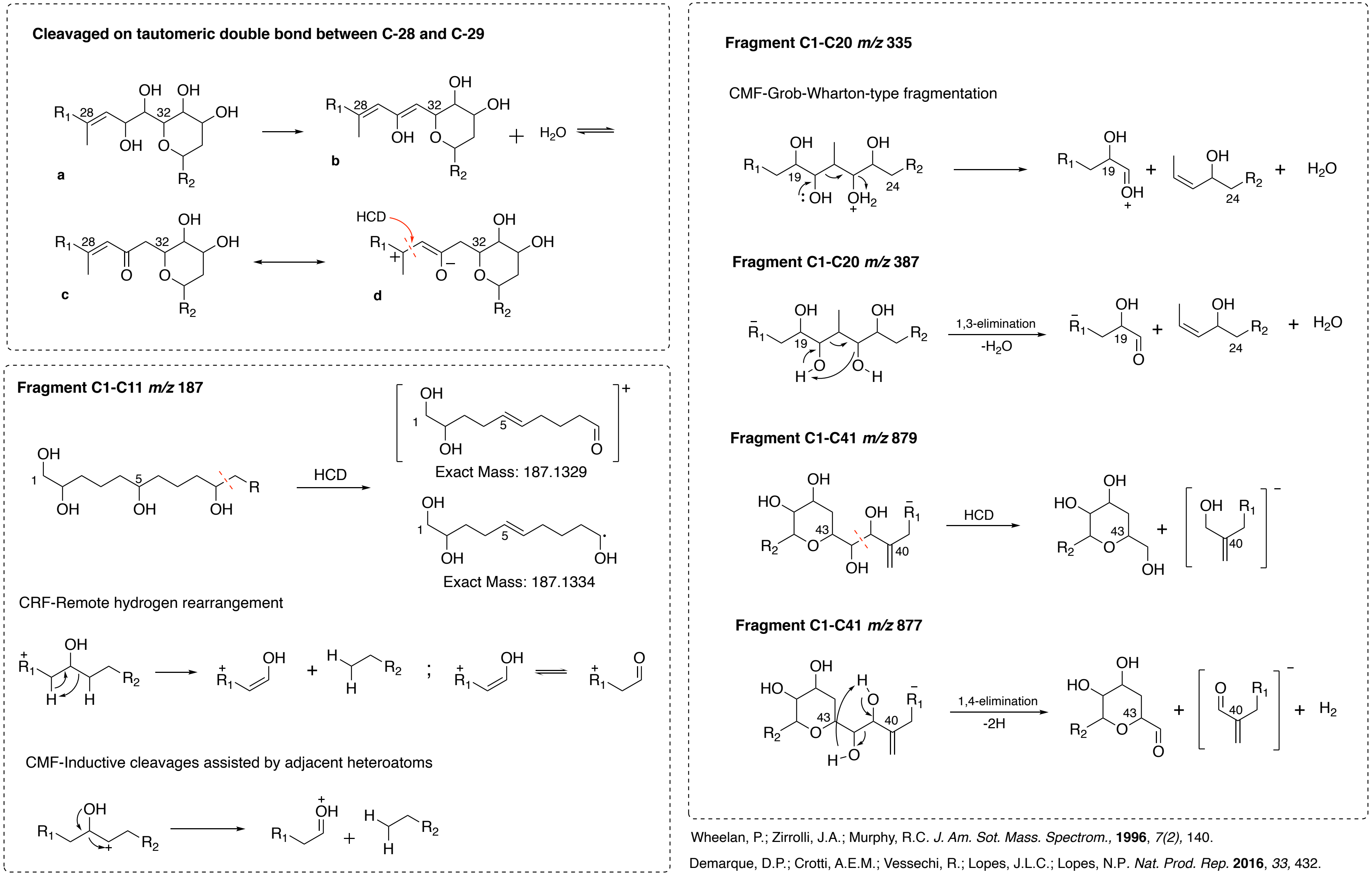

Wheelan, P.; Zirrolli, J.A.; Murphy, R.C. *J. Am. Sot. Mass. Spectrom.,* **1996**, *7(2),* 140.

Demarque, D.P.; Crotti, A.E.M.; Vessechi, R.; Lopes, J.L.C.; Lopes, N.P. *Nat. Prod. Rep.* **2016**, *33,* 432.

S3. Search for amphidinols in non-targeted LC-HRMS-MS^2 experiments

Table S5. Amphidinol-related analogs published hitherto.

Compound Name	Chemical Formula	Exact Mass	Organism	Original Reference
2-desulfo-Amphidinol 7	$C_{59}H_{100}O_{20}$	1128.6808	*Amphidinium klebsii*	*Bioorg. & Med. Chem.,* **2006**, 14(19), 6548.
Amphidinol 15	$C_{59}H_{102}O_{22}$	1162.6862	*A. klebsii*	*Bioorg. & Med. Chem.,* **2006**, 14(19), 6548.
Colopsinol C	$C_{62}H_{104}O_{20}S$	1200.6842	*Amphidinium* sp.	*J.Chem. Soc., Perkin Trans.* 1, **1999**, 3483
Amphidinol 7	$C_{59}H_{100}O_{23}S$	1208.6376	*A. klebsii*	*Tetrahedron,* **2005**, 61, 8606
Lingshuiol B	$C_{60}H_{100}O_{23}S$	1220.6376	*Amphidinium* sp.	*Tetrahedron Lett.*, **2004**, 45, 5501.
Symbiopolyol	$C_{60}H_{100}O_{23}S$	1220.6376	Simbiotic dinoflagellate	*J. Nat. Prod.*, **2010**, 73(7), 1318
Amphidinol 14	$C_{59}H_{102}O_{25}S$	1242.6431	*A. klebsii*	*Bioorg. & Med. Chem.,* **2006**, 14(19), 6548.
Colopsinol E	$C_{65}H_{110}O_{20}S$	1242.7311	*Amphidinium* sp.	*Chem. Pharm. Bull.,* **2000**, 48(10) 1447.
Amphezonol A	$C_{62}H_{114}O_{24}$	1242.7700	*Amphidinium* sp	*Tetrahedron Lett.,* **2006**, 47 4369
Luteophanol A	$C_{60}H_{102}O_{25}S$	1254.6431	*Amphidinium* sp.	*J. Org. Chem.,***1997**, 62(12), 3820
Amdigenol G	$C_{60}H_{102}O_{25}S$	1254.6431	*Amphidinium* sp.	*Tetrahedron Lett.,* **2014**, 55(46), 6319
Lingshuiol A	$C_{66}H_{112}O_{23}$	1272.7594	*Amphidinium* sp.	*Tetrahedron Lett.,* **2004**, 45, 5501.
Amphidinol 10	$C_{66}H_{112}O_{23}$	1272.7594	*Amphidinium* spp.	*Harmful Algae*, **2005**, 4, 383.
Amphidinol 17	$C_{63}H_{112}O_{24}S$	1282.7108	*Amphidinium carterae*	*J. Nat. Prod.*, **2010**, 73(3), 409
Amphidinol 4	$C_{68}H_{116}O_{23}$	1300.7907	*A. carterae* & *A. klebsii*	*Tetrahedron*, **2001**, 57(26), 5551
Luteophanol D	$C_{66}H_{114}O_{25}$	1306.7649	*A. carterae*	*Mar. Drugs*, **2005**, 3(4), 113
Luteophanol C	$C_{67}H_{116}O_{25}$	1320.7806	*Amphidinium* sp.	*Tetrahedron,* **1998**, 54(48), 14455.
Luteophanol B	$C_{67}H_{116}O_{25}$	1320.7806	*Amphidinium* sp.	*Tetrahedron,* **1998**, 54(48), 14455.
Amphidinol 20B	$C_{66}H_{114}O_{26}$	1322.7599	*A. carterae*	*Algal Res.,* **2018**, 31, 87
Amphidinol 3	$C_{70}H_{118}O_{23}$	1326.8064	*A. klebsii*	*J. Am. Chem. Soc.,* **1999**, 121, 870.
Amphidinol 9	$C_{70}H_{118}O_{23}$	1326.8064	*Amphidinium* spp.	*Harmful Algae,* 4, **2005**, 383
Amphidinol A	$C_{69}H_{126}O_{24}$	1338.8635	*A. carterae*	*Mar. Drugs,* **2017**, 15(6): 157.
Amphidinol 6	$C_{70}H_{120}O_{24}$	1344.8169	*A. klebsii*	*J. Mar. Biotechnol.*, **1997**, *5(2)*, 124.

NOTE: Amphidinol 20B (AM 20B) is named “B” to distinguish between two analogs that were published almost simultaneously with the same number.

Compound Name	Chemical Formula	Exact Mass	Organism	Original Reference
Lingshuiol	$C_{69}H_{122}O_{25}$	1350.8275	*Amphidinium* sp.	*Bioorg. & Med. Chem. Lett.,* **2004**, 14, 3117.
Amphidinol 18	$C_{71}H_{122}O_{24}$	1358.8326	*A. carterae*	*J. Nat. Prod.*, **2014**, 77(6), 1524
Colopsinol B	$C_{68}H_{114}O_{25}S$	1362.7370	*Amphidinium* sp.	*J. Chem. Soc.*, **1999**, 1, 3483
Amphidinol 5	$C_{72}H_{122}O_{24}$	1370.8326	*A. klebsii*	*J. Mar. Biotechnol.*, **1997**, 5(2), 124.
Amphidinol 2	$C_{71}H_{122}O_{25}$	1374.8275	*A. klebsii*	*Tetrahedron Lett.,* **1995**, 36(35), 6279
Amphidinol 12	$C_{68}H_{116}O_{26}S$	1380.7476	*Amphidinium* spp.	*Harmful Algae*, **2005**, 4, 383
Colopsinol D	$C_{71}H_{118}O_{24}S$	1386.7734	*Amphidinium* sp.	*Chem. Pharm. Bull.,* **2000**, 48 (10), 1447.
Carteraol E	$C_{74}H_{126}O_{24}$	1398.8639	*A.carterae*	*Tetrahedron Lett.*, **2009**, 50, 2512
Colopsinol A	$C_{71}H_{120}O_{25}S$	1404.7839	*Amphidinium* sp.	*J. Org. Chem.,* **1999***,* 64(5), 1478
Amphidinol 13	$C_{70}H_{118}O_{26}S$	1406.7632	*Amphidinium* spp.	*Harmful Algae,* **2005**, 4, 383
Amphidinol B	$C_{69}H_{126}O_{27}S$	1418.8207	*A. carterae*	*Mar. Drugs*, **2017**, 15(6), 157.
Amphidinol 19	$C_{71}H_{122}O_{27}S$	1438.7894	*A.carterae*	*J. Nat. Prod.*, **2014**, 77(6), 1524
Karatungiol B	$C_{73}H_{130}O_{27}$	1438. 8799	*Amphidinium* sp.	*Tetrahedron Lett.,* **2006**, 47, 2521
Amphidinol 11	$C_{71}H_{122}O_{28}S$	1454.7843	*Amphidinium* spp.	*Harmful Algae,* **2005**, 4, 383
Karatungiol A	$C_{73}H_{132}O_{28}$	1456.8905	*Amphidinium* sp.	*Tetrahedron Lett.,* **2006**, 47, 2521
Amphidinol	$C_{73}H_{126}O_{27}S$	1466.8207	*A. klebsii*	*J. Am. Chem. Soc.*, **1991**, 113, 9861.
Amphidinol 20	$C_{87}H_{152}O_{27}$	1629.0521	*A.carterae*	*J. Nat. Prod.*, **2017**, 80, 11, 2883
Amphidinol 22	$C_{84}H_{140}O_{31}$	1644.9379	*A.carterae*	*Mar. Drugs*, **2019**, *17*(7), 385
Amdigenol E	$C_{82}H_{138}O_{37}S$	1746.8637	*Amphidinium* sp.	*Tetrahedron Lett.***,** **2014**, 55(46)*,* 6319
Amphidinol 21	$C_{94}H_{166}O_{30}$	1775.1464	*A.carterae*	*J. Nat. Prod.,* **2017**, 80, 11, 2883
Amdigenol A	$C_{104}H_{178}O_{42}S$	2131.1513	*A.carterae*	*Tetrahedron Lett.,* **2012***,* 53, 239

RScript S1. R Script used for monitoring fragments and total water losses along MS^2 spectra on mzXML files.

````
---
title: "Support to mol nets"
author: "author"
date: "date"
output:
  html_document:
    df_print: paged
---

## Prepare list of precursors and their MS2 spectra
```{r echo = FALSE, message = FALSE, warning = FALSE}
library("MSnbase")
library(plotly)

rawdata <- readMSData("/FilePath", msLevel = 2, verbose = FALSE) #Here the path to your file

hd <- fData(rawdata)

prec <- rep(NA, length(hd$spectrum))

for( i in 1:length(hd$spectrum))
{
prec[i] <- rawdata[[i]]@precursorMz
}

hd$Precursor_mz <- prec

list_prec <- vector(mode = "list", length = length(prec))

names(list_prec) <- c(round(prec, digits = 2))

for( i in 1:length(hd$spectrum))
{
  list_prec[[c(i)]]   <- c(list_prec[[c(i)]], list("m/z"=NULL, "int"=NULL))
}

for( i in 1:length(hd$spectrum))
{
  list_prec[[c(i, 1)]]   <- c(list_prec[[c(i, 1)]], rawdata[[i]]@mz)
  list_prec[[c(i, 2)]]   <- c(list_prec[[c(i, 2)]], rawdata[[i]]@intensity)
}
```

## Look for a fragment in all spectra

```{r echo = FALSE, message = FALSE, warning = FALSE}

m1 <- 877.5166 #m/z of interest
ppm_error <- 5 #mass tolerance in ppm

df_min <- data.frame(prec_index = hd$spectrum,
  precursor_mz = hd$Precursor_mz,
                  min = rep(NA, length(prec)),
                   max = rep(NA, length(prec)),
````
````

````
                    mz_found =rep(NA, length(prec)),
                    mz_error = rep(NA, length(prec)))

for( i in 1:length(prec))
{
  df_min$min[i] <- min(list_prec[[c(i, 1)]])
  df_min$max[i] <- max(list_prec[[c(i, 1)]])
  df_min$mz_found[i] <- list_prec[[c(i, 1)]][which.min(abs(list_prec[[c(i, 1)]]-m1))]
  df_min$mz_error[i] <- round((abs((m1 - df_min$mz_found[i])/m1)*1E6), digits = 2)
}

df_selec <- df_min[df_min$mz_error<ppm_error,]

df_selec
```

## Plot all spectra that contain a selected fragment

```{r echo = FALSE, message = FALSE, warning = FALSE}

for (i in 1:length(df_selec$prec_index))
{
print(ggplotly(plot(rawdata[[df_selec$prec_index[i]]])+ geom_vline(xintercept = m1, linetype="dotted", color =
"blue", size=0.5)))
}

```



## Calculate how many water losses are in all spectra
```{r echo = FALSE, message = FALSE, warning = FALSE}

df_wloss_all <- data.frame(precursor_mz = hd$Precursor_mz,
                          amount_wloss = rep(NA, length(prec)))

for (i in 1:length(df_wloss_all$amount_wloss))
{
  df_wloss_all$amount_wloss[i] <- length(which((outer(list_prec[[c(i, 1)]],list_prec[[c(i, 1)]], '-') > 17.9) &
                                          (outer(list_prec[[c(i, 1)]],list_prec[[c(i, 1)]], '-') < 18.1)))
}
df_wloss_all_order <- df_wloss_all[order(-df_wloss_all$amount_wloss),]
df_wloss_all_order

ggplotly(ggplot(df_wloss_all_order , aes(x=precursor_mz, y=amount_wloss)) +
  geom_point(size=0.5, alpha = 0.8) + geom_vline(xintercept = 1307.77, linetype="dotted", color = "red",
size=0.5, alpha = 0.5))

```

## Look how many water losses there are in a spectra

```{r echo = FALSE, message = FALSE, warning = FALSE}

mz_wloss <- 1307.77                                                              #The precursor to
plot

spec_num <- hd[which.min(abs(hd$Precursor_mz-mz_wloss)),1]
````

```
df <- data.frame(x = list_prec[[c(spec_num, 1)]], y= list_prec[[c(spec_num, 1)]])
dst <- outer(df$x, df$y, '-')
mat_dist <- as.matrix(dst)
rownames(mat_dist) <- round(list_prec[[c(spec_num, 1)]], digits = 1)
colnames(mat_dist) <- round(list_prec[[c(spec_num, 1)]], digits = 1)
loss_water_val <- dst[(which(dst > 17.9 & dst < 18.1, arr.ind=TRUE))]
amount_w_loss <- length(loss_water_val)
loss_index <- which(mat_dist > 17.9 & dst < 18.1, arr.ind=TRUE)
loss_mz <- cbind(rownames(mat_dist)[loss_index[,"row"]], colnames(mat_dist)[loss_index[,"col"]])

print(ggplotly(plot(rawdata[[spec_num]])))
print(list_wloss <- list("Amount of H2O losses" = amount_w_loss, "mz H2O losses" = loss_mz))
```

Table S6. Precursor ions containing diagnostic MS^2 fragments in cell-free medium extracts. The *m/z* values shown for the precursor ions and fragment ions are averaged when more than one ion arose for the same compound.

**Fragment ion *m/z* 187** (*clv k*; *m/z* theo. 187.1329)			**Fragment ion *m/z* 609** (*clv f*; *m/z* theo. 609.3997)			**Fragment ion *m/z* 877** (*clv b*; *m/z* theo. 877.5166)		
**ACBR01**								
**Precursor ion (*m/z*)**	**Fragment ion (*m/z*)**	**Δppm**	**Precursor ion (*m/z*)**	**Fragment ion (*m/z*)**	**Δppm**	**Precursor ion (*m/z*)**	**Fragment ion (*m/z*)**	**Δppm**
-	-	-	1277.7102	609.4000	0.49	-	-	-
			1145.6682	609.4014	2.79			
**ACRN02**								
**Precursor ion (*m/z*)**	**Fragment ion (*m/z*)**	**Δppm**	**Precursor ion (*m/z*)**	**Fragment ion (*m/z*)**	**Δppm**	**Precursor ion (*m/z*)**	**Fragment ion (*m/z*)**	**Δppm**
1339.7594	187.1335	3.20	1323.7665	609.3993	-0.65	1415.7374	877.5172	0.68
1307.7731	187.1327	-1.07	1321.7526	609.3983	-2.29	1401.7554	877.5196	3.41
1305.7595	187.1337	4.27	1307.7724	609.3983	-2.29	1385.7693	877.5173	0.80
1289.7613	187.1332	1.60	1305.7574	609.4009	1.97	1383.7561	877.5187	2.39
1287.7455	187.1322	-3.74	1289.7666	609.3999	0.33	1367.7610	877.5181	1.71
1271.7574	187.1333	2.13	1271.7516	609.3988	-1.47	1351.7663	877.5179	1.48
1103.6031	187.1323	-3.21	1219.6641	609.3990	-1.15	1317.6765	877.5173	0.80
1071.5917	187.1324	-2.67	1185.6595	609.3994	-0.49	1301.6800	877.5156	-1.14
1036.5752	187.1334	2.67	1183.6467	609.3972	-4.10	1259.6710	877.5160	-0.68
1028.0717	187.1332	1.60	1167.6515	609.3985	-1.97	1245.6503	877.5189	2.62
			1101.6024	609.3982	-2.46	1233.6487	877.5297	4.67
			1083.5914	609.4017	3.28	1217.6540	877.5167	0.11
			1071.5906	609.3992	-0.82	1215.6406	877.5192	2.92
						1199.6445	877.5193	3.08
**ACRN03**								
**Precursor ion (*m/z*)**	**Fragment ion (*m/z*)**	**Δppm**	**Precursor ion (*m/z*)**	**Fragment ion (*m/z*)**	**Δppm**	**Precursor ion (*m/z*)**	**Fragment ion (*m/z*)**	**Δppm**
1339.7586	187.1334	2.71	1357.7811	609.4000	0.49	1499.8021	877.5138	-3.19
1309.7771	187.1325	2.10	1339.7602	609.3984	-2.13	1415.7385	877.5156	-1.14
1307.7729	187.1327	0.88	1323.7655	609.3985	-1.97	1401.7619	877.5171	0.60
			1321.7495	609.3984	-2.13	1399.7473	877.5146	-2.28
			1307.7722	609.3995	-0.33	1385.7650	877.5174	0.91
			1305.7547	609.3992	-0.82	1383.7534	877.5181	1.71
			1289.7607	609.3995	-0.33	1369.7494	877.5176	1.14
			1219.6657	609.3970	-4.43	1367.7591	877.5175	0.62
			1185.6600	609.3978	-3.12	1351.7667	877.5165	1.03
			1167.6477	609.3999	0.33	1321.7498	877.5148	-2.05
			1071.5922	609.3985	-1.97	1247.6625	877.5181	1.71
						1233.6482	877.5171	0.57
						1217.6518	877.5163	-0.34
						1215.6422	877.5154	-1.37
						1199.6456	877.5165	-0.11
						1185.6307	877.5184	2.05
						1183.6481	877.5140	-2.96
						1155.6186	877.5161	-0.57
**ACMK03**								
**Ion**	***m/z***	**Δppm**	**Ion**	***m/z***	**Δppm**	**Ion**	***m/z***	**Δppm**
-	-	-	-	-	-	-	-	-

Figure S7. Water loss count in MS^2 spectra.

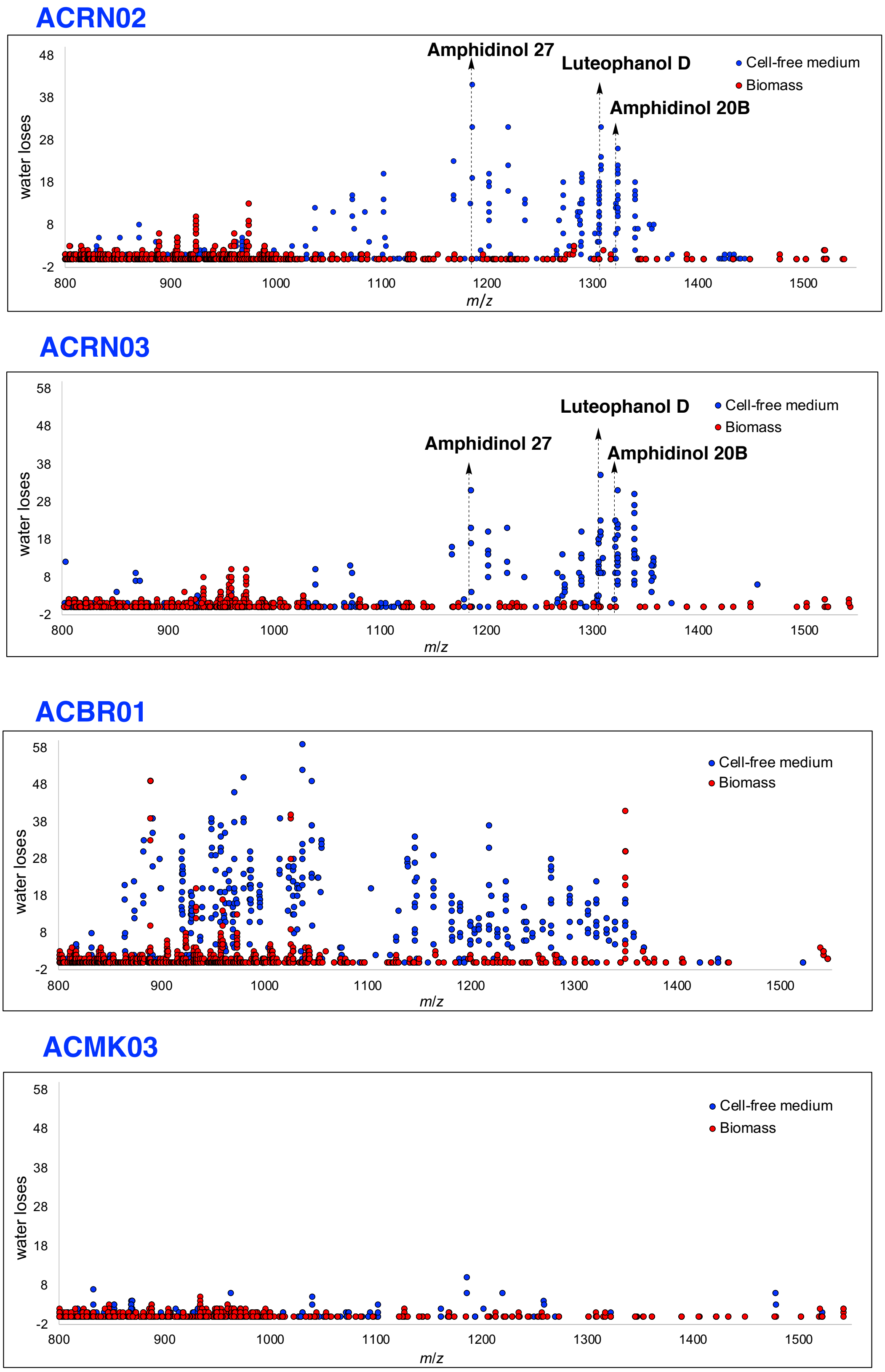

Figure S8. Structures of luteophanol D and amphidinols 20B, 24 and 27-36.

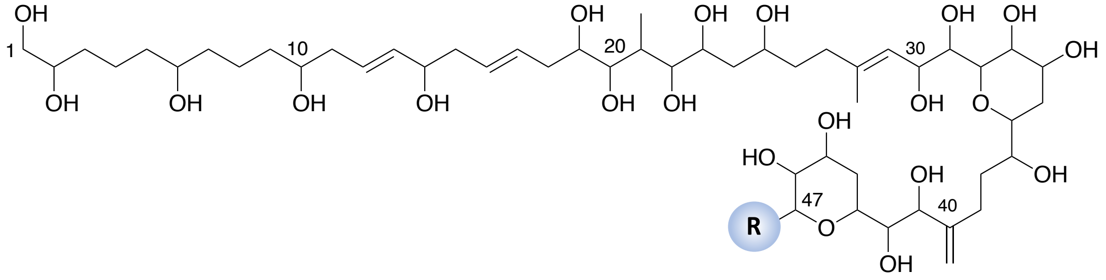

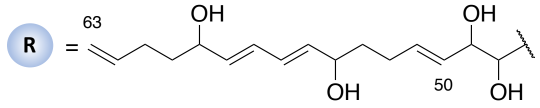

Luteophanol D **(1)**

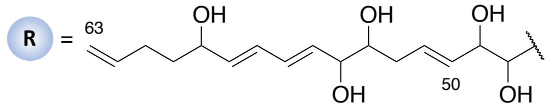

Amphidinol 20B (AM 20B) (**2**)

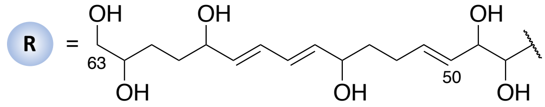

Amphidinol 24 (AM 24) **(3)**

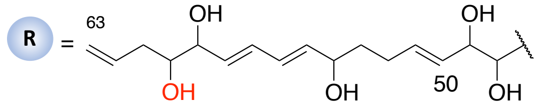

Amphidinol 28 (AM 28) **(4)**

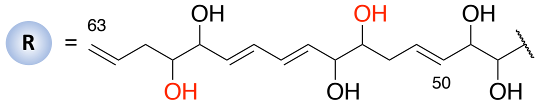

Amphidinol 29 (AM 29) **(5)**

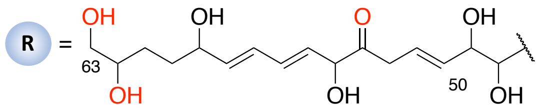

Amphidinol 35 (AM 35) **(6)**

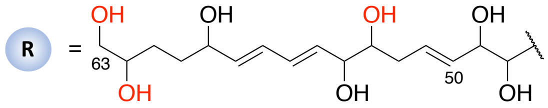

Amphidinol 36 (AM 36) (**7**)

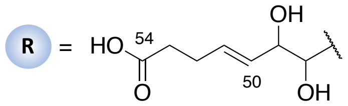

Amphidinol 27 (AM 27) **(8)**

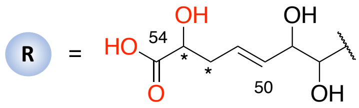

Amphidinol 30 (AM 30) **(9)**

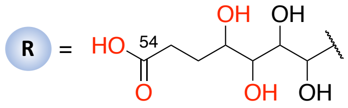

Amphidinol 31 (AM 31) **(10)**

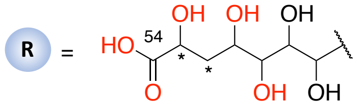

Amphidinol 32 (AM 32) **(11)**

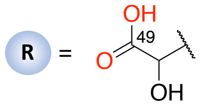

Amphidinol 33 (AM 33) **(12)**

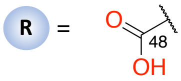

Amphidinol 34 (AM 34) **(13)**

Note: Hydroxyls in red are located according to MS² fragmentation. (*) The position of the hydroxyl group in amphidinols 30 and 32 can be located at position C-52 or C-53. Radical (R) starts at C-47.

Table S7. Description of the amphidinol analogs reported in this study.

Dereplicated are underlined.

Compound	Exact mass	ΔLutD	Equiv.	Formula	RDB	$[M+H]^+$ ion				$[M+HCOO]^-$ ion				Strain
						m/z theo.	*m/z* exp.	Δppm	RT	*m/z* theo.	*m/z* exp.	Δppm	RT	
Luteophanol D	1306.7649	-	-	$C_{66}H_{114}O_{25}$	10.0	1307.7722	1307.7731	0.67	4.90	1351.7631	1351.7664	2.44	4.91	ACRN02
							1307.7703	–1.48	4.89		-	-	-	ACRN03
AM 28	1322.7598	+16	+O	$C_{66}H_{114}O_{26}$	10.0	1323.7671	1323.7683	0.91	4.53	1367.7580	1367.7589	0.65	4.54	ACRN02
							1323.7655	–1.12	4.52		1367.7588	0.58	4.53	ACRN03
AM 20B	1322.7598	+16	+O	$C_{66}H_{114}O_{26}$	10.0	1323.7671	1323.7673	0.17	5.15	1367.7580	1367.7612	2.34	5.07	ACRN02
							-	-	-		-	-	-	ACRN03
AM 29	1338.7547	+32	$+O_2$	$C_{66}H_{114}O_{27}$	10.0	1339.7620	1339.7631	0.77	4.70	1383.7529	1383.7557	2.02	4.70	ACRN02
							1339.7600	–1.51	4.69		1383.7544	1.54	4.72	ACRN03
AM 24	1340.7704	+34	+2OH	$C_{66}H_{116}O_{27}$	9.0	-	-	-	-	1385.7686	1385.7694	0.57	4.45	ACRN02
							-	-	-		1385.7661	1.80	4.25	ACRN03
AM 27	1184.6554	–122	$–C_9H_{14}$	$C_{57}H_{100}O_{25}$	8.0	1185.6627	1185.6602	–2.10	4.27	-	-	-	-	ACRN02
							1185.6580	–3.87	4.26		-	-	-	ACRN03
AM 30	1200.6503	–106	$–C_9H_{14}$; +O	$C_{57}H_{100}O_{26}$	8.0	1201.6576	1201.6545	–1.80	4.21	-	-	-	-	ACRN02
							1201.6528	–3.94	4.23		-	-	-	ACRN03
AM 31	1218.6608	–88	$–C_9H_{12}$; $+O_2$	$C_{57}H_{102}O_{27}$	7.0	1219.6681	1219.6648	–2.73	4.15	-	-	-	-	ACRN02
							1219.6644	–3.03	4.17		-	-	-	ACRN03
AM 32	1234.6558	–72	$–C_9H_{12}$; +3O	$C_{57}H_{102}O_{28}$	7.0	1235.6630	1235.6648	1.42	4.21	-	-	-	-	ACRN02
							-	-	-		-	-	-	ACRN03
AM 33	1100.5979	–206	$–C_{14}H_{22}O$	$C_{52}H_{92}O_{24}$	7.0	1101.6051	1101.6027	–2.24	4.17	-	-	-	-	ACRN02
							-		-		-	-	-	ACRN03
AM 34	1070.5873	–236	$–C_{15}H_{24}O_2$	$C_{51}H_{90}O_{23}$	7.0	1071.5946	1071.5917	–2.70	4.23	-	-	-	-	ACRN02
							-	-	-		-	-	-	ACRN03
AM 35	1354.7497	+48	+3O	$C_{66}H_{114}O_{28}$	10.0	1355.7569	-	-	-	-	-	-	-	ACRN02
							1355.7548	–1.61	4.68	-	-	-	-	ACRN03
AM 36	1356.7653	+50	+2OH; +O	$C_{66}H_{116}O_{28}$	9.0	1357.7726	-	-	-	1401.7635	-	-	-	ACRN02
							1357.7732	0.45	4.21		1401.7622	–0.93	4.24	ACRN03

Scheme S2. Structural relationships between the amphidinol analogs.

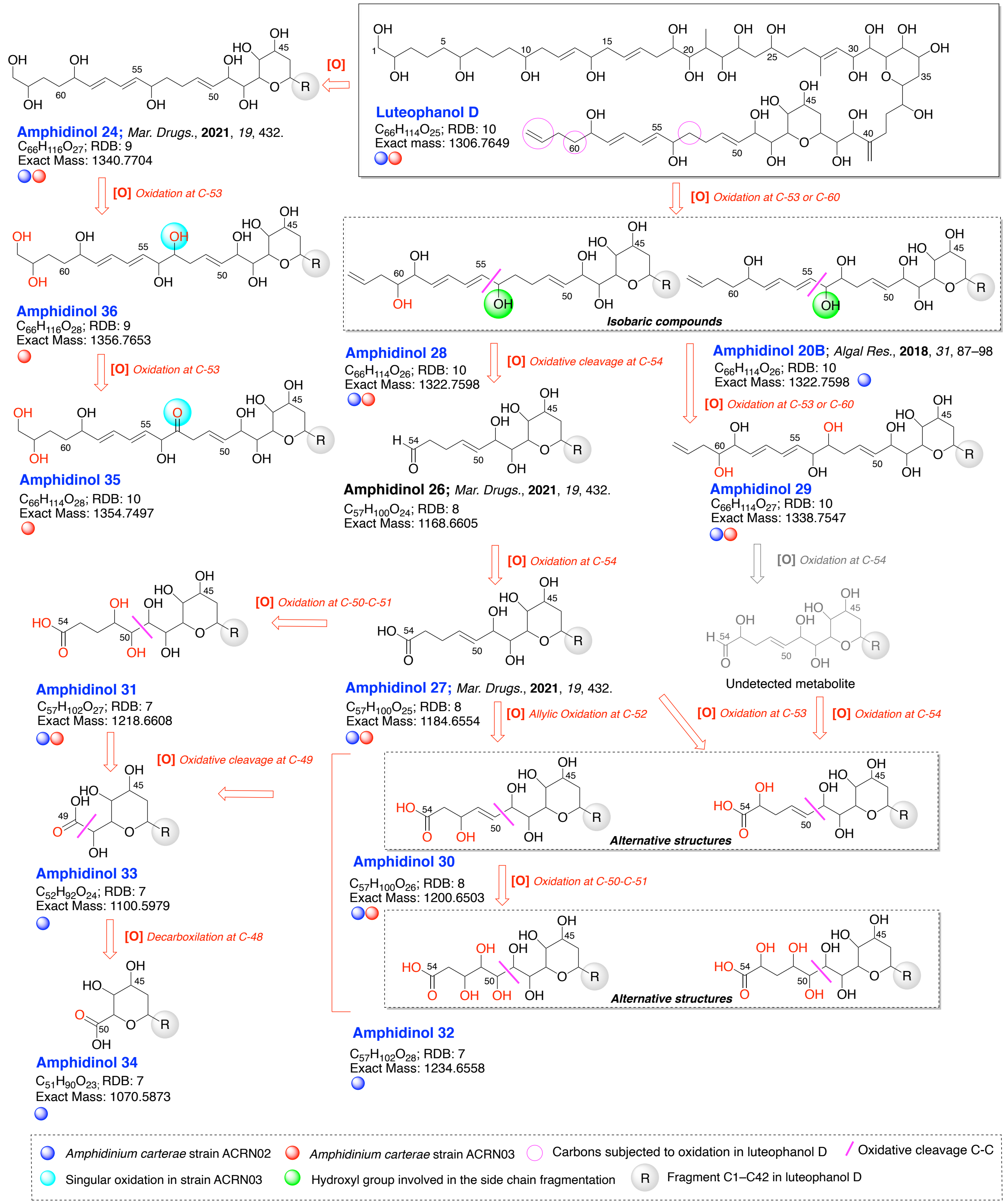

S4. Molecular networking (MN) analysis of non-targeted LC-MS² experiments.

Figure S9. MN of cell-free medium extract of **ACBR01** in ESI+ mode.
Colored by parental mass value

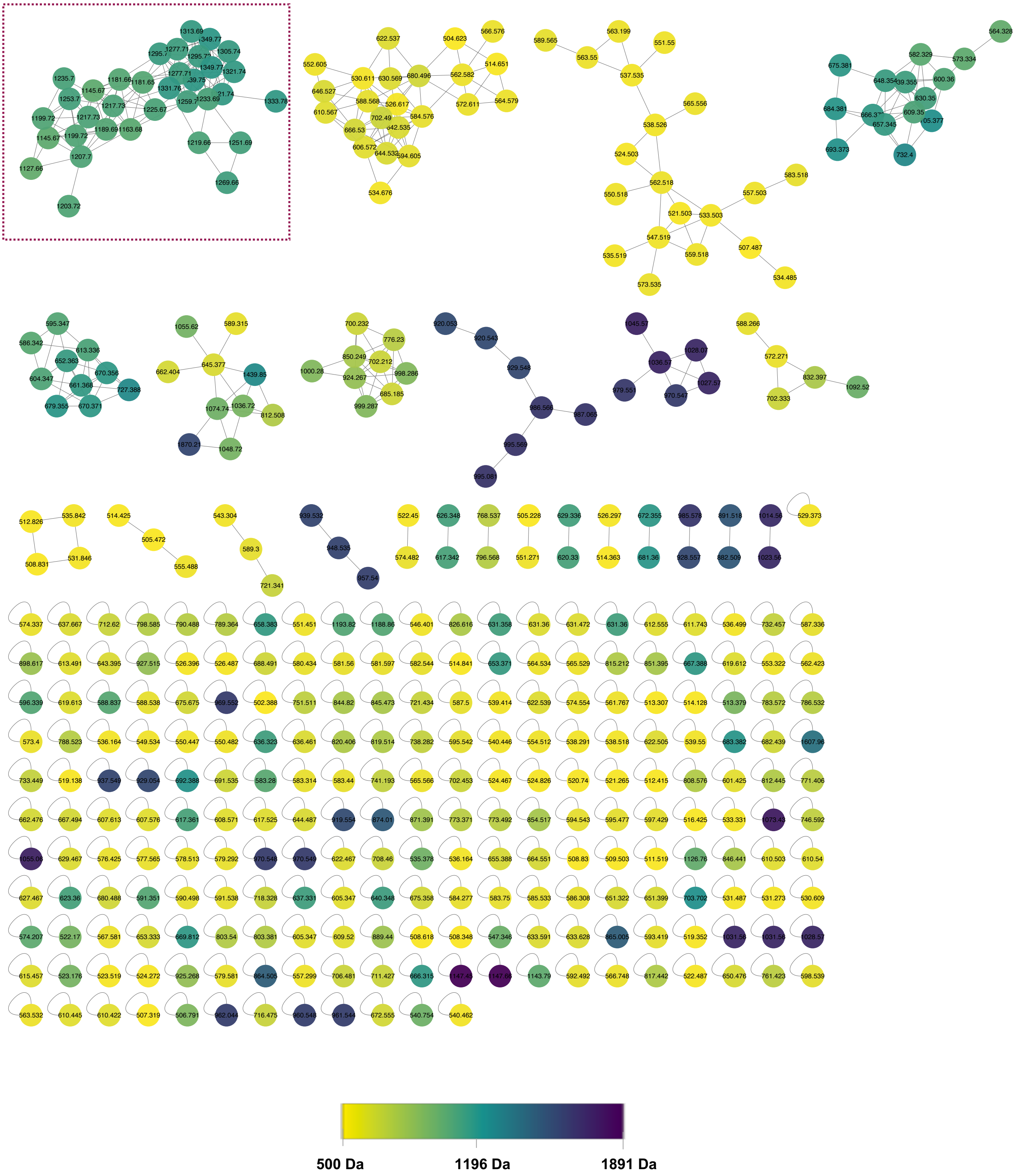

Figure S10. MN of cell-free medium extract of **ACBR01** in ESI- mode.
Colored by parental mass value.

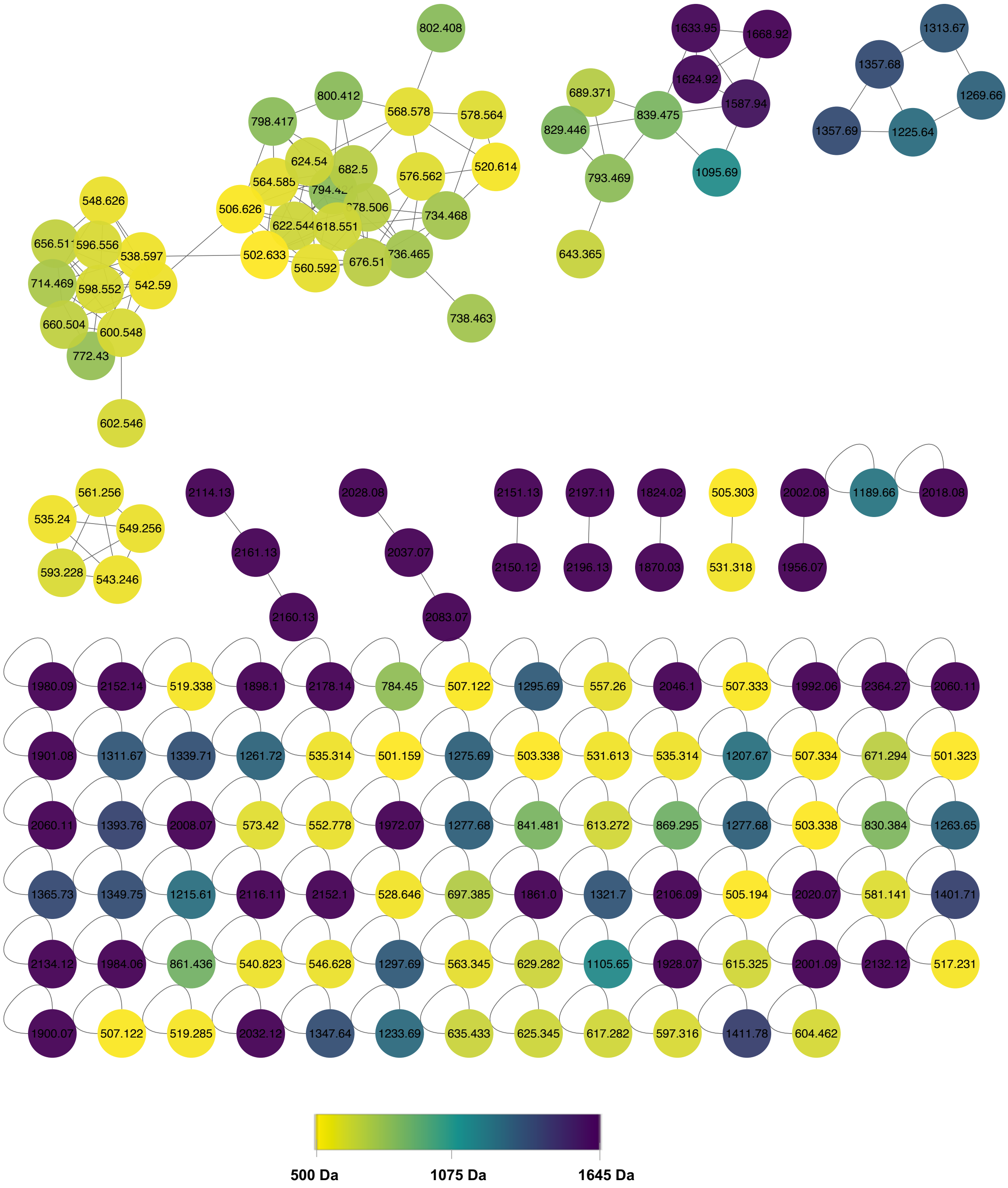

Figure S11. MN of biomass extract of **ACBR01** in ESI+ mode.
Colored by parental mass value.

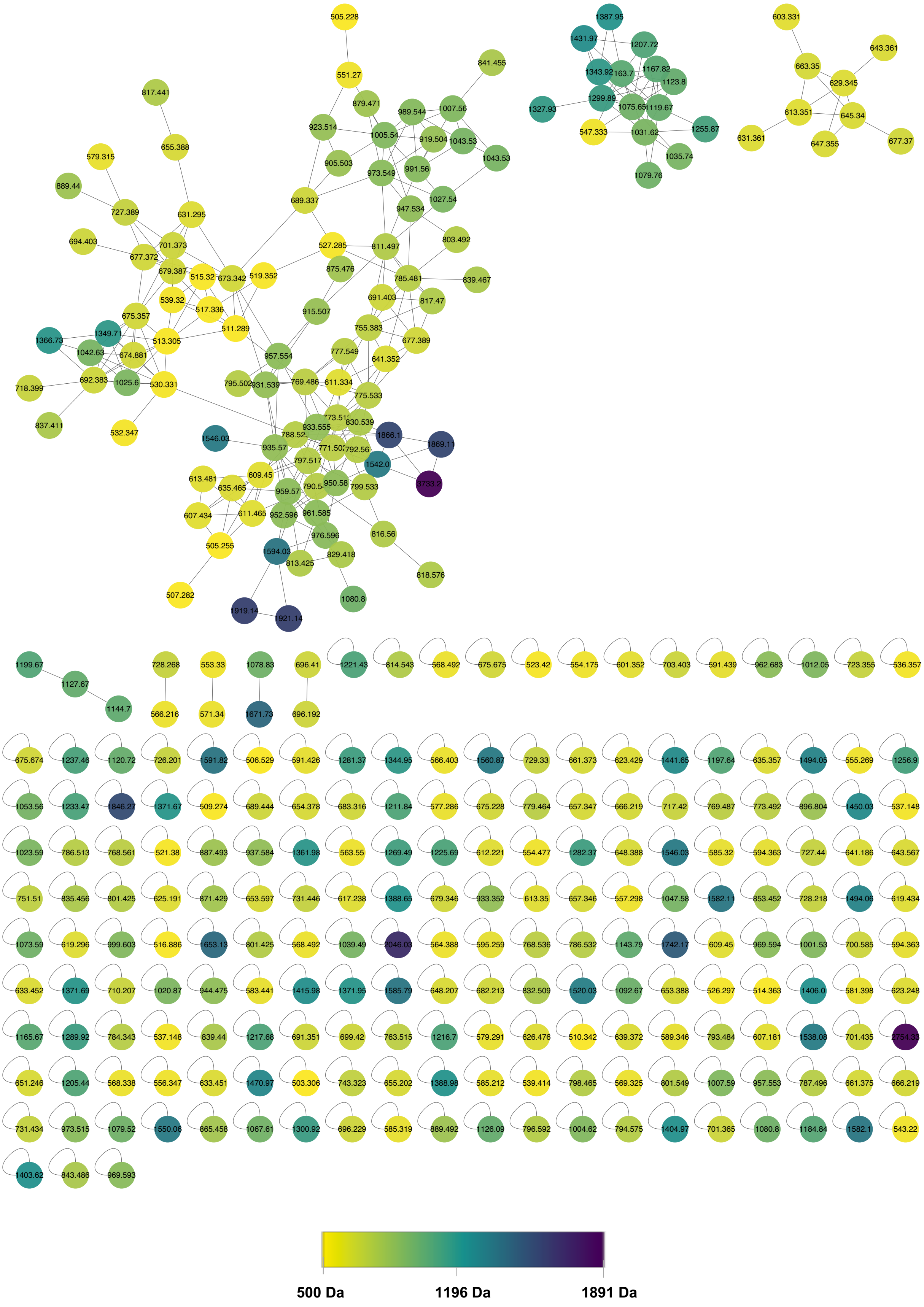

Figure S12. MN of cell-free medium extract of **ACRN02** in ESI+ mode.
Colored by parental mass value.

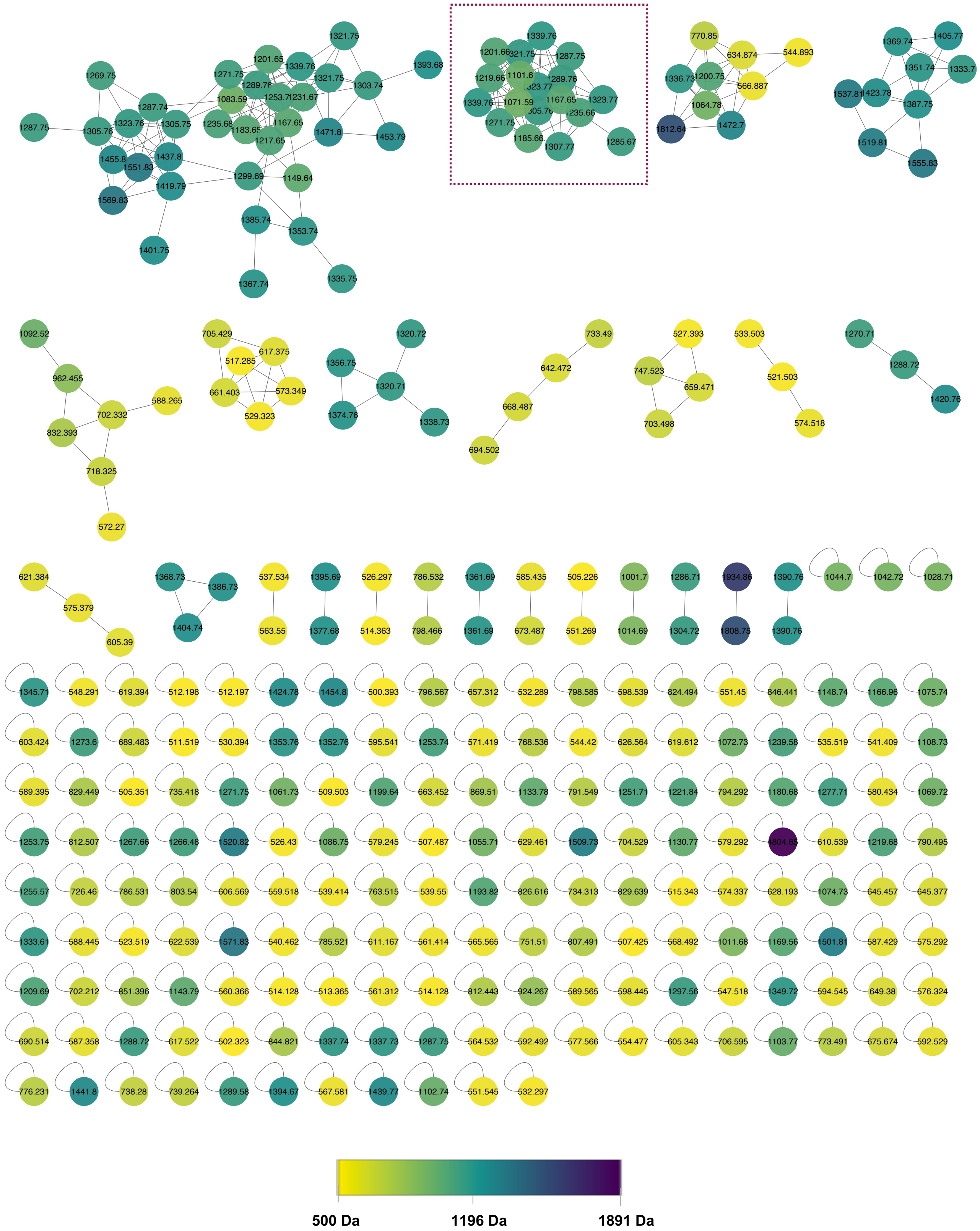

Figure S13. MN of cell-free medium extract of **ACRN02** in ESI- mode. Colored by parental mass value.

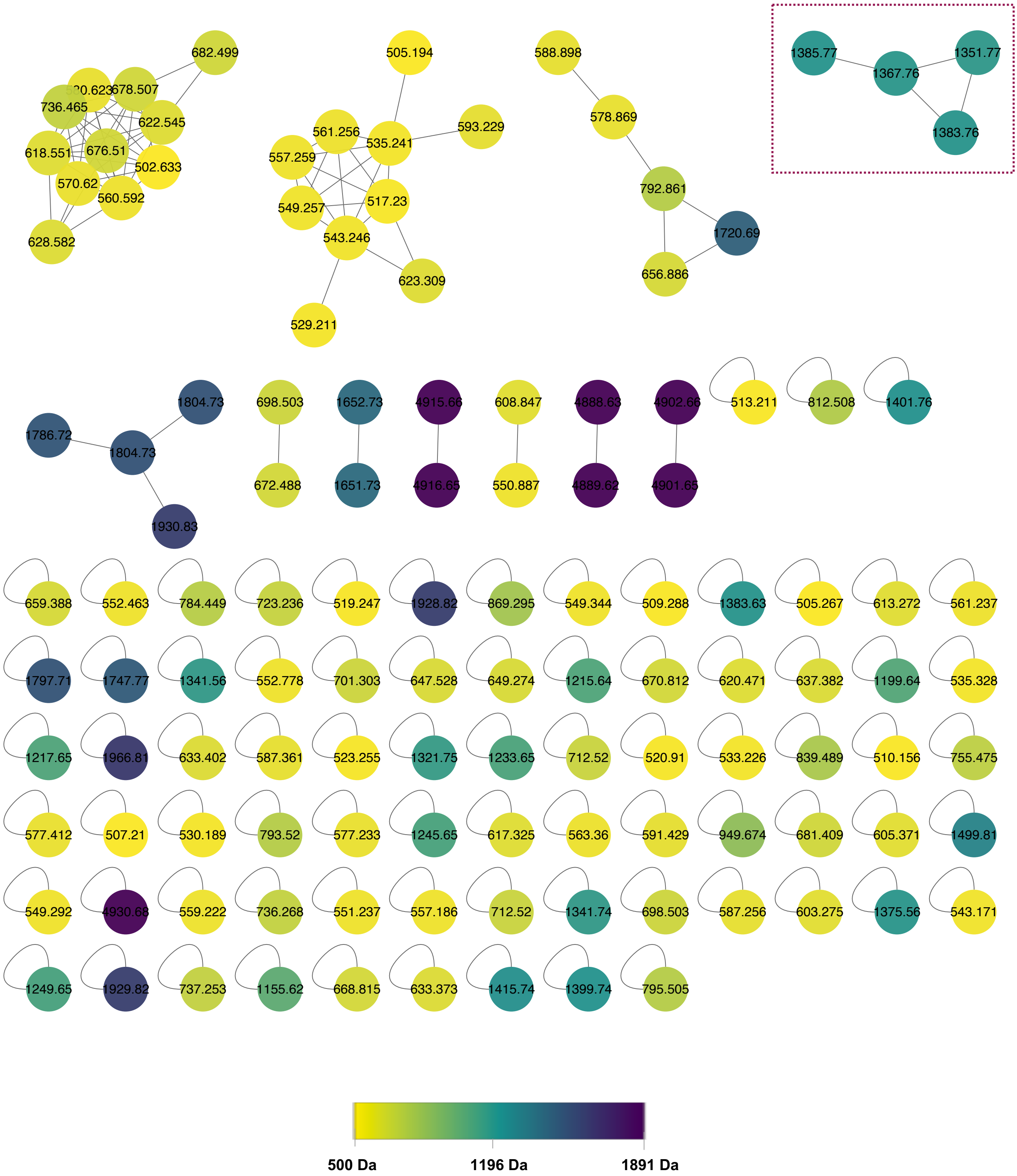

Figure S14. MN of biomass extract of **ACRN02** in ESI+ mode. Colored by parental mass value.

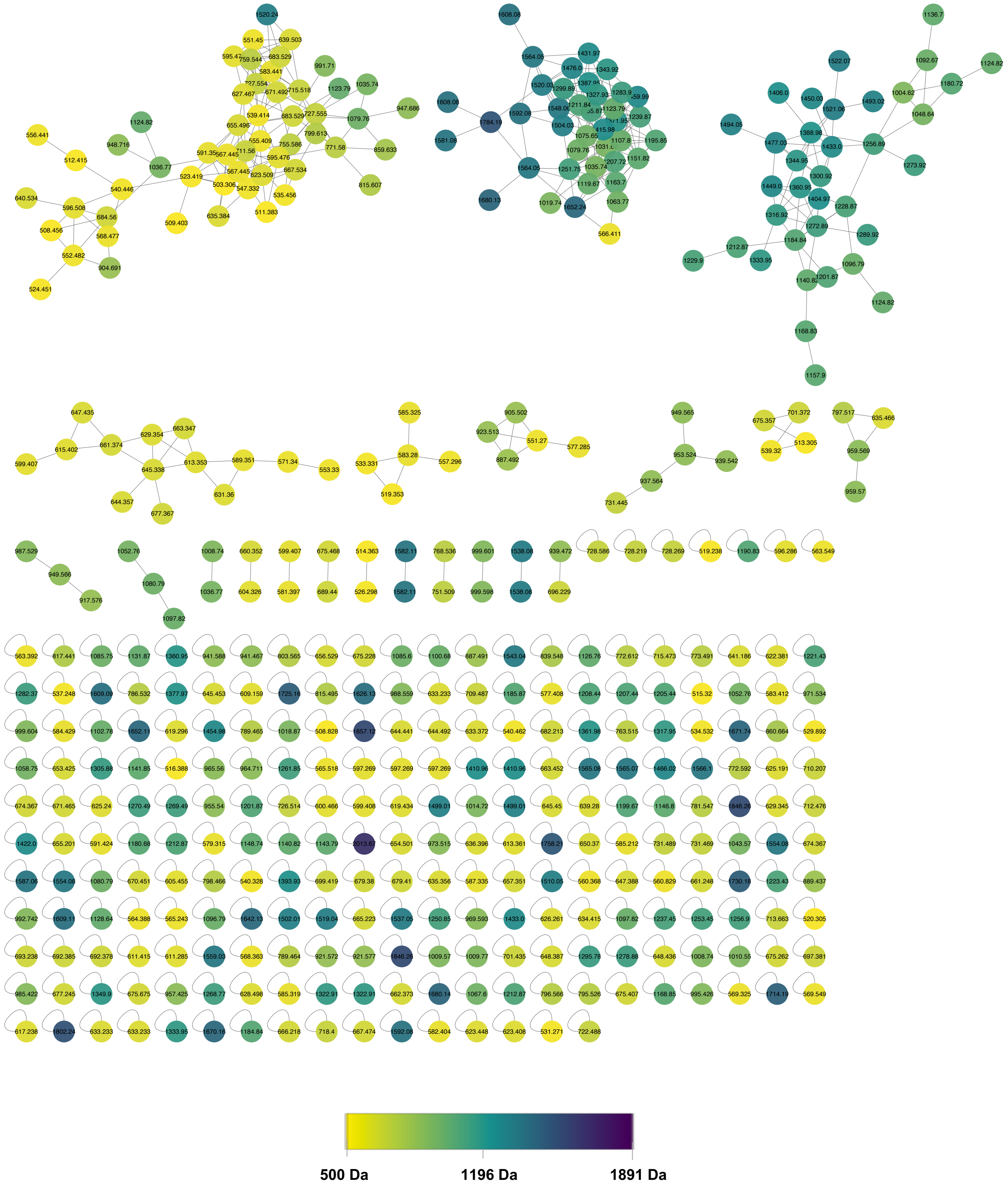

Figure S15. MN of cell-free medium extract of **ACRN03** in ESI+ mode.
Colored by parental mass value.

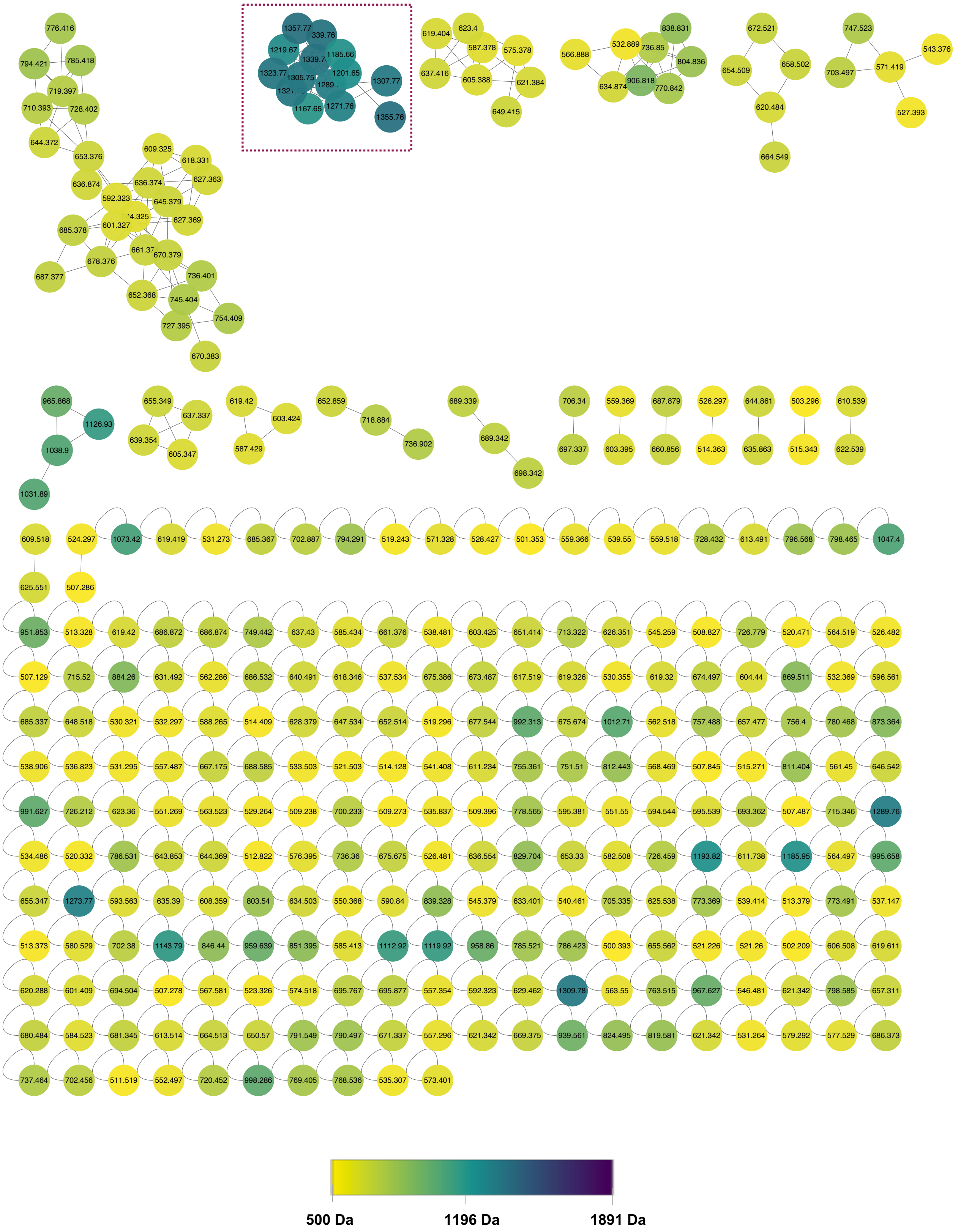

Figure S16. MN of cell-free medium extract of ACRN03 in ESI- mode. Colored by parental mass value.

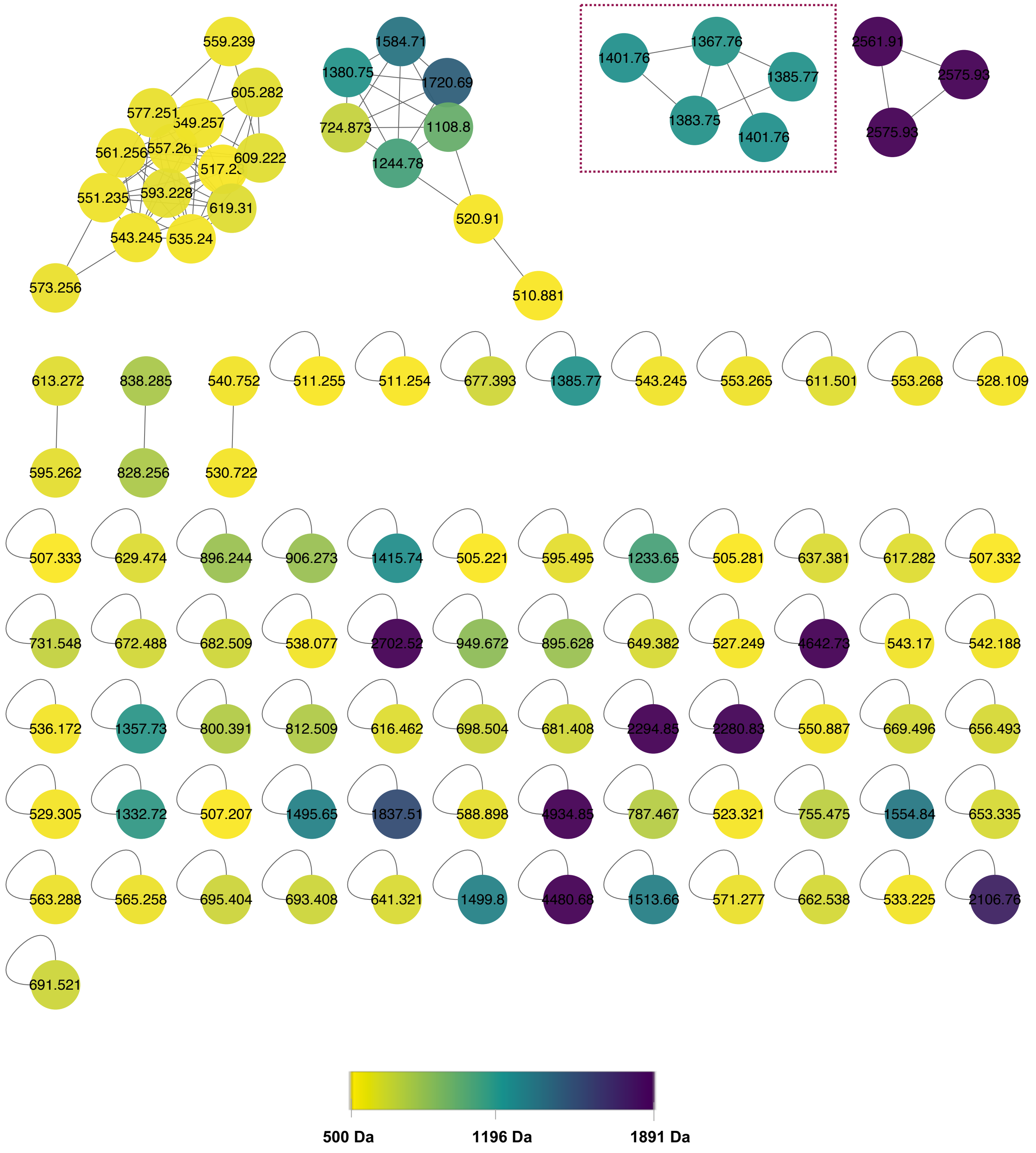

Figure S17. MN of biomass extract of **ACRN03** in ESI+ mode.
Colored by parental mass value.

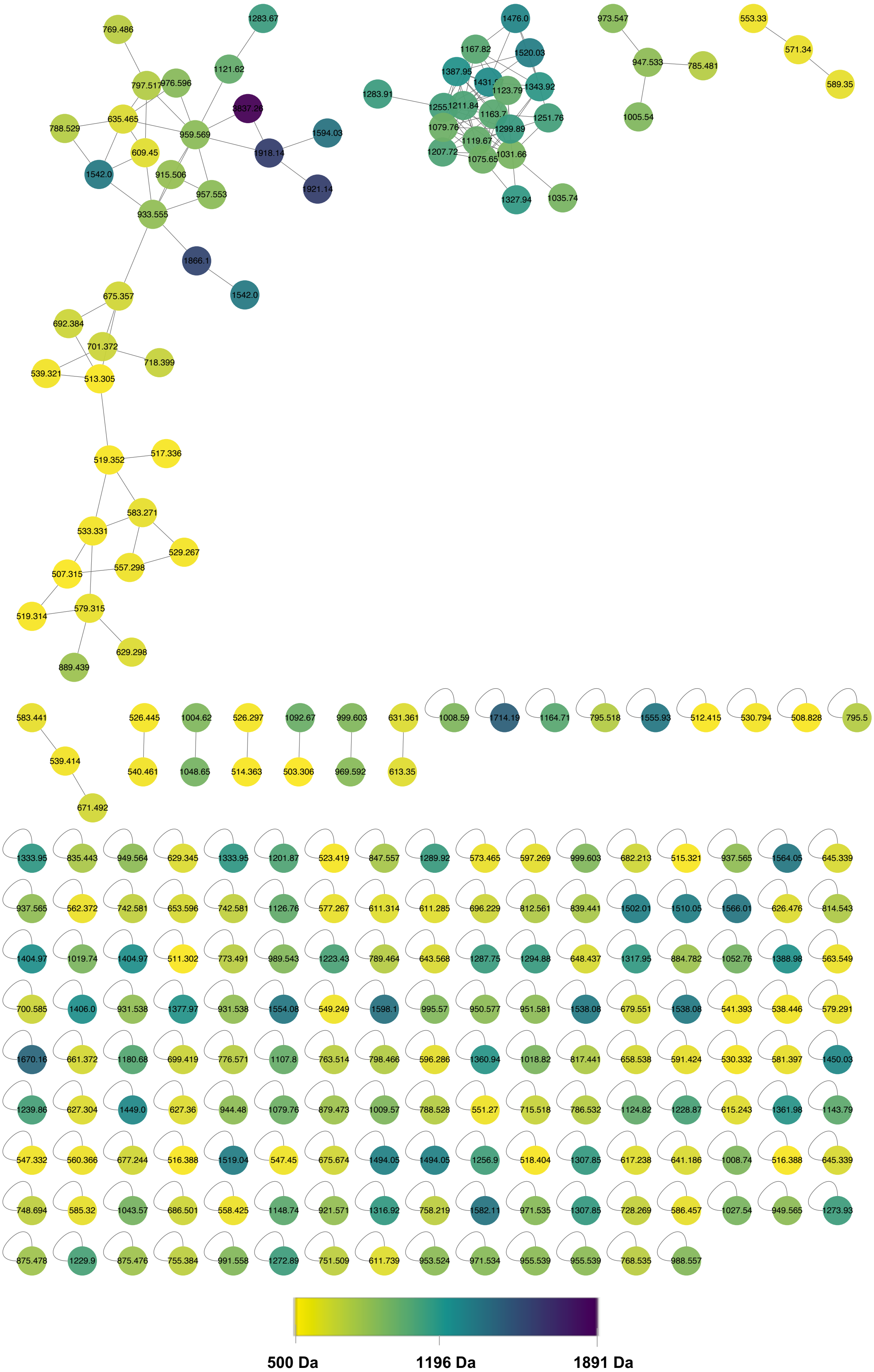

Figure S18. MN of cell-free medium extract of **ACMK03** in ESI+ mode.
Colored by parental mass value.

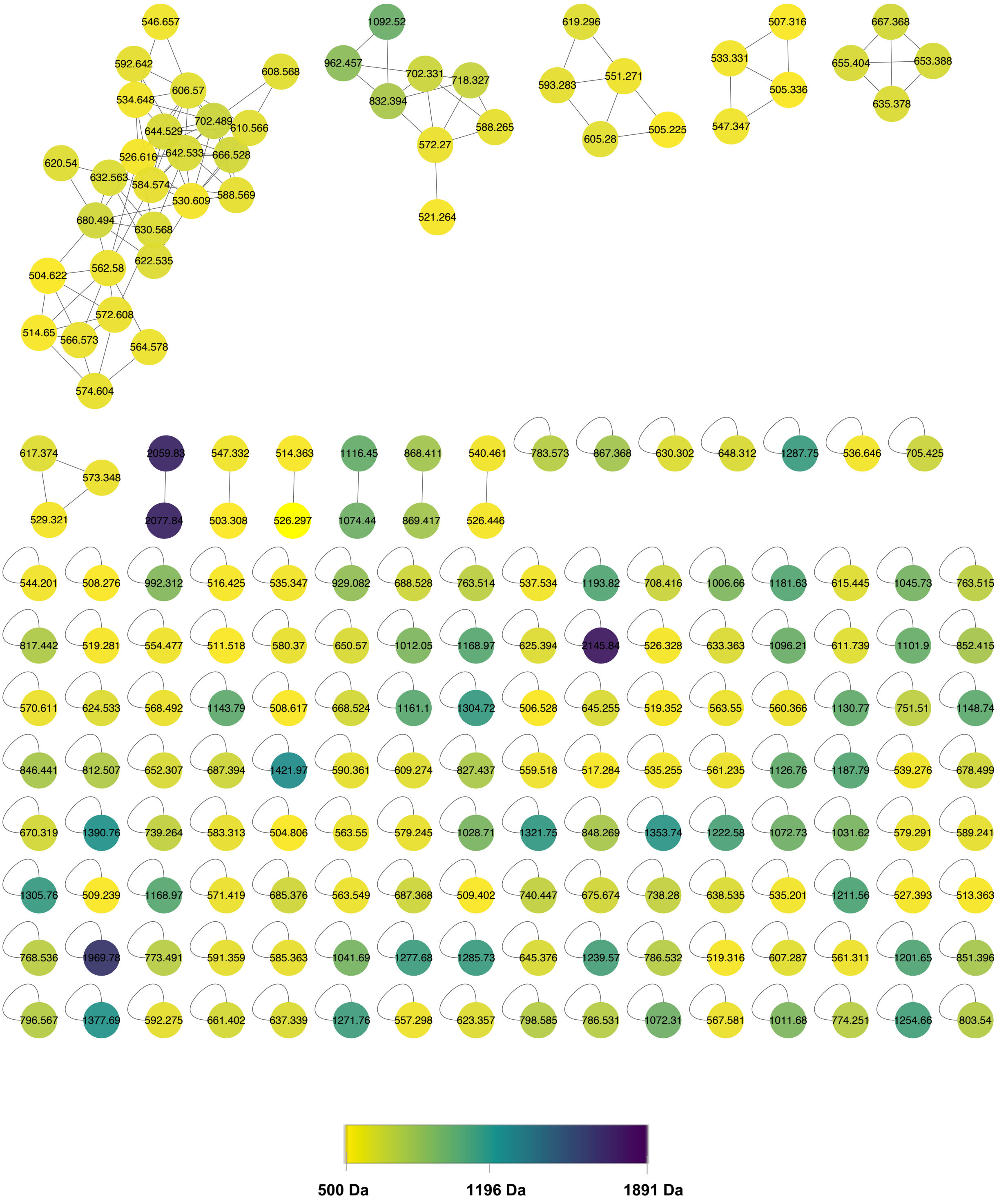

Figure S19. MN of cell-free medium extract of **ACMK03** in ESI- mode.
Colored by parental mass value.

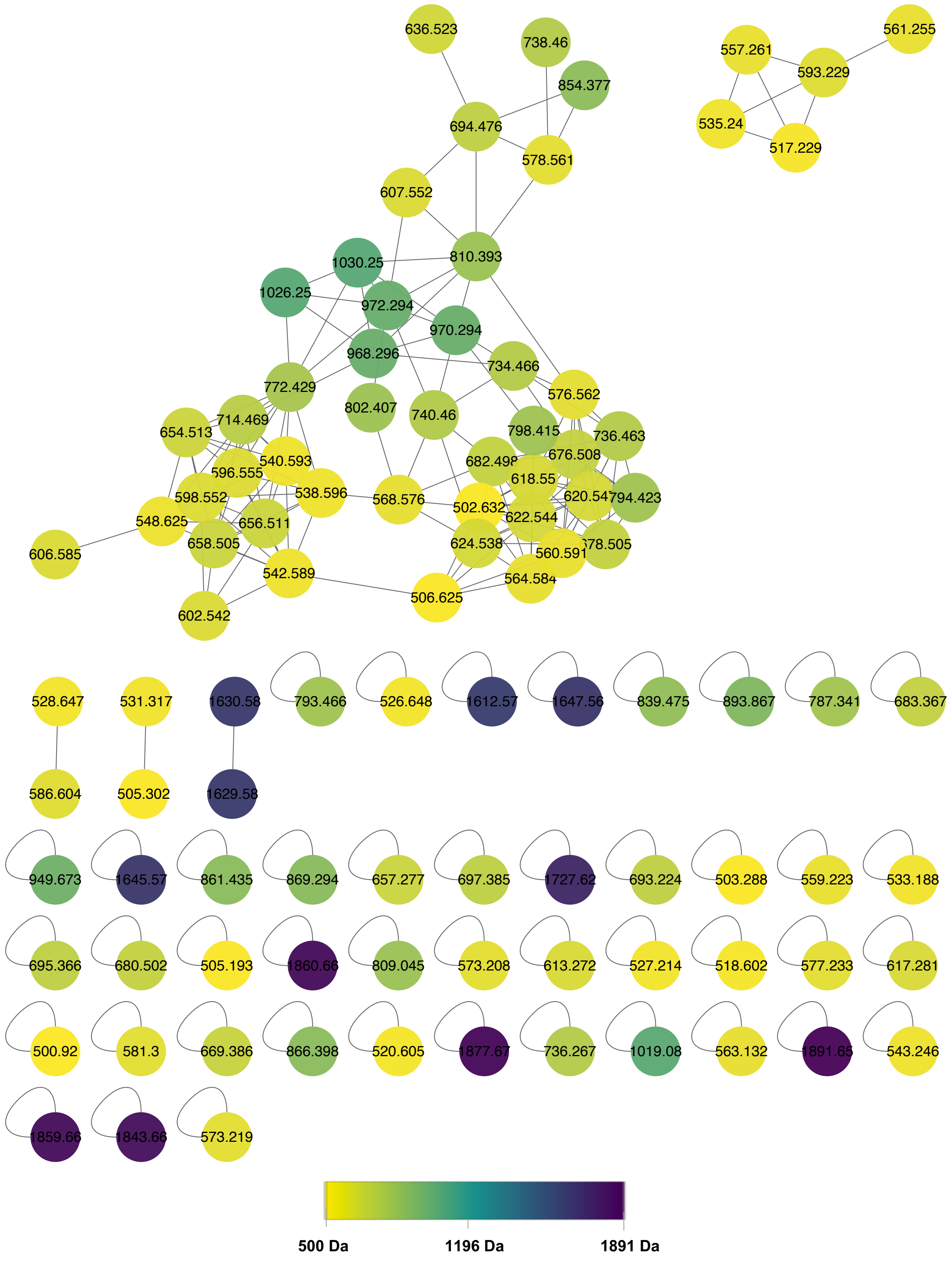

Figure S20. MN of biomass extract of **ACMK03** in ESI+ mode.
Colored by parental mass value.

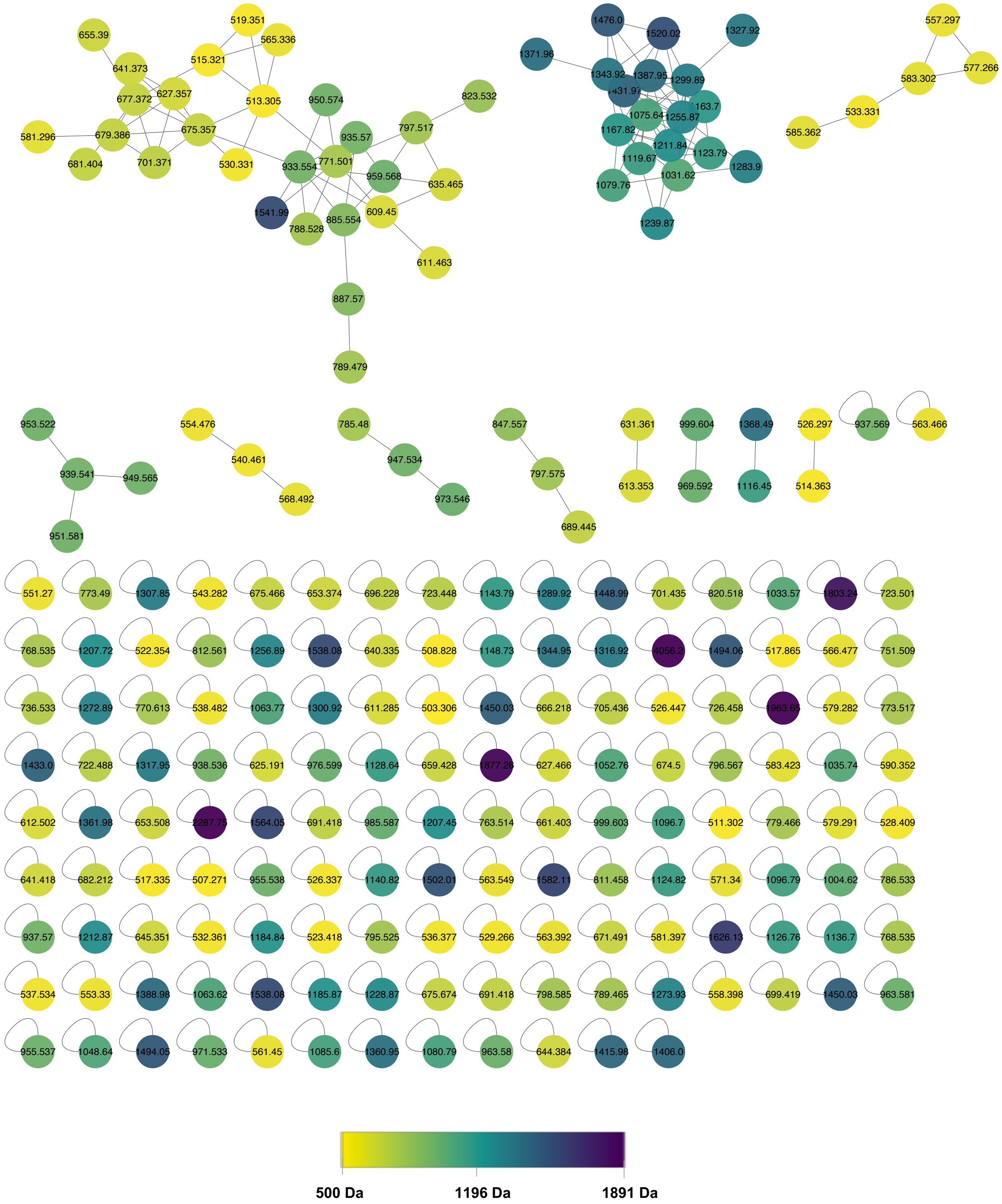

Figure S21. MN of all cell-free medium extracts combined in ESI+ mode. Nodes charted by strain of origin; borders colored by parental mass.

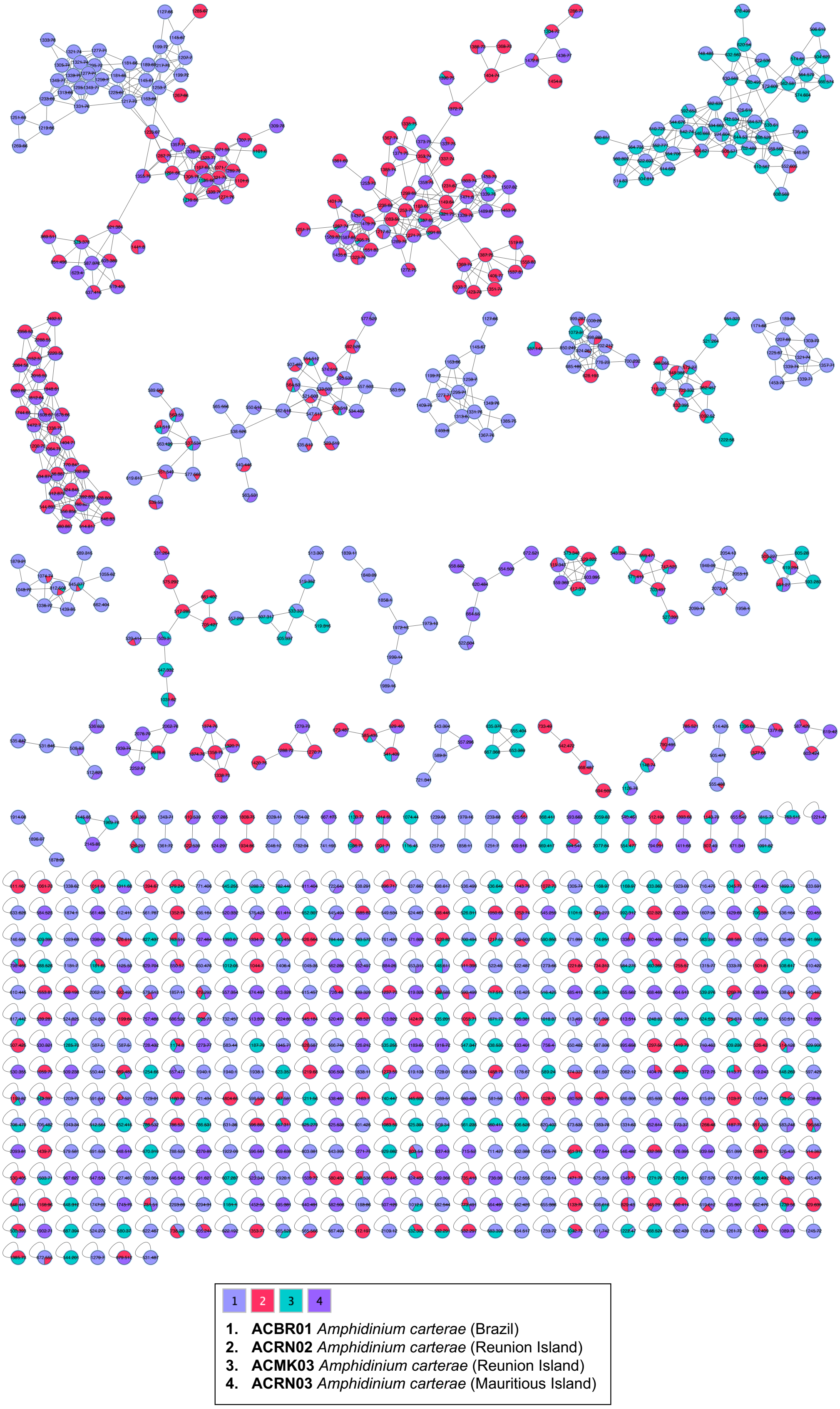

Figure S22. Common family of secreted amphidinols in ESI+ mode.
Nodes charted by strain of origin; borders colored by parental mass.

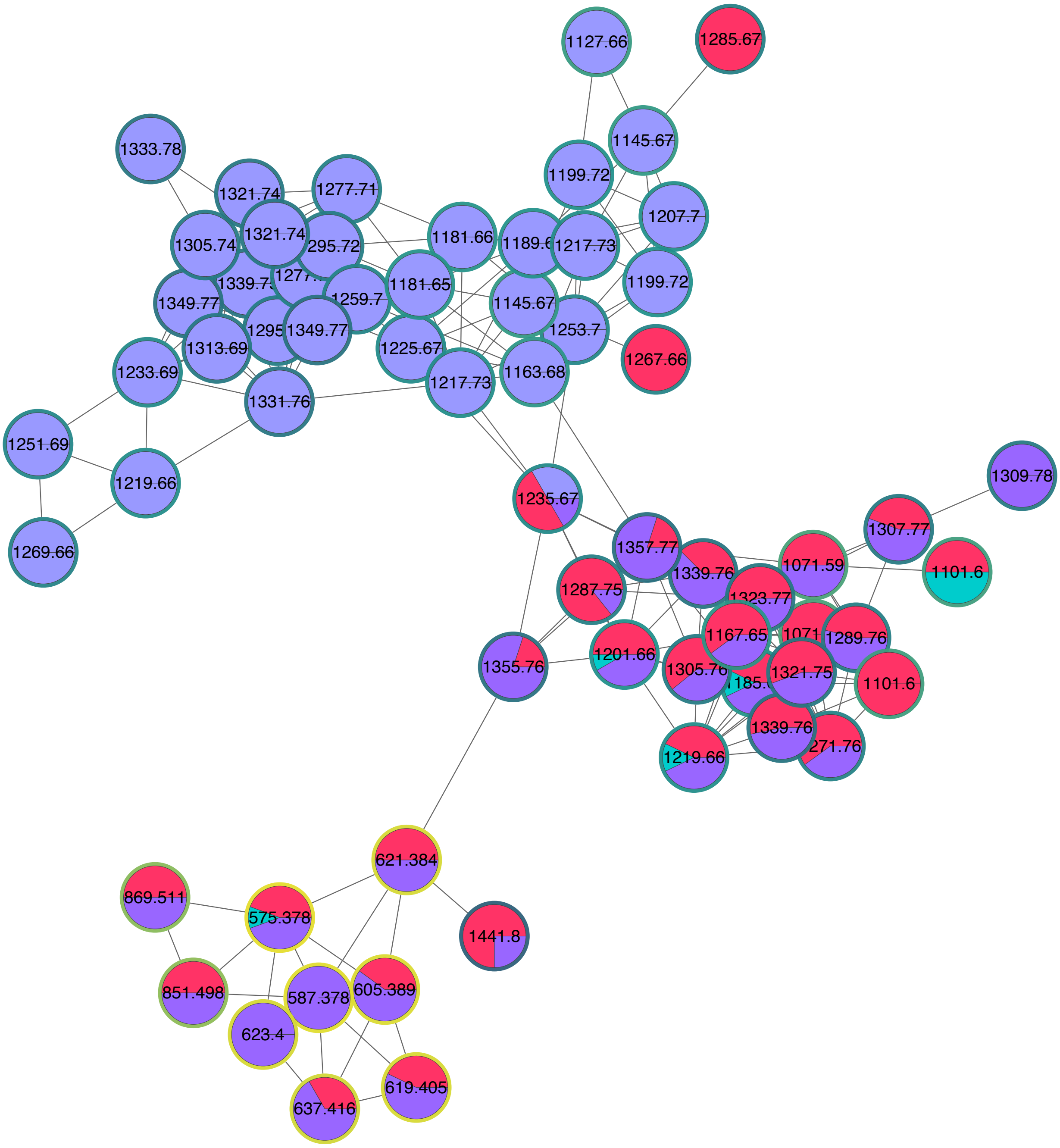

1 2 3 4

1. **ACBR01** *Amphidinium carterae* (Brazil)
2. **ACRN02** *Amphidinium carterae* (Reunion Island)
3. **ACMK03** *Amphidinium carterae* (Reunion Island)
4. **ACRN03** *Amphidinium carterae* (Mauritious Island)

Refined family:

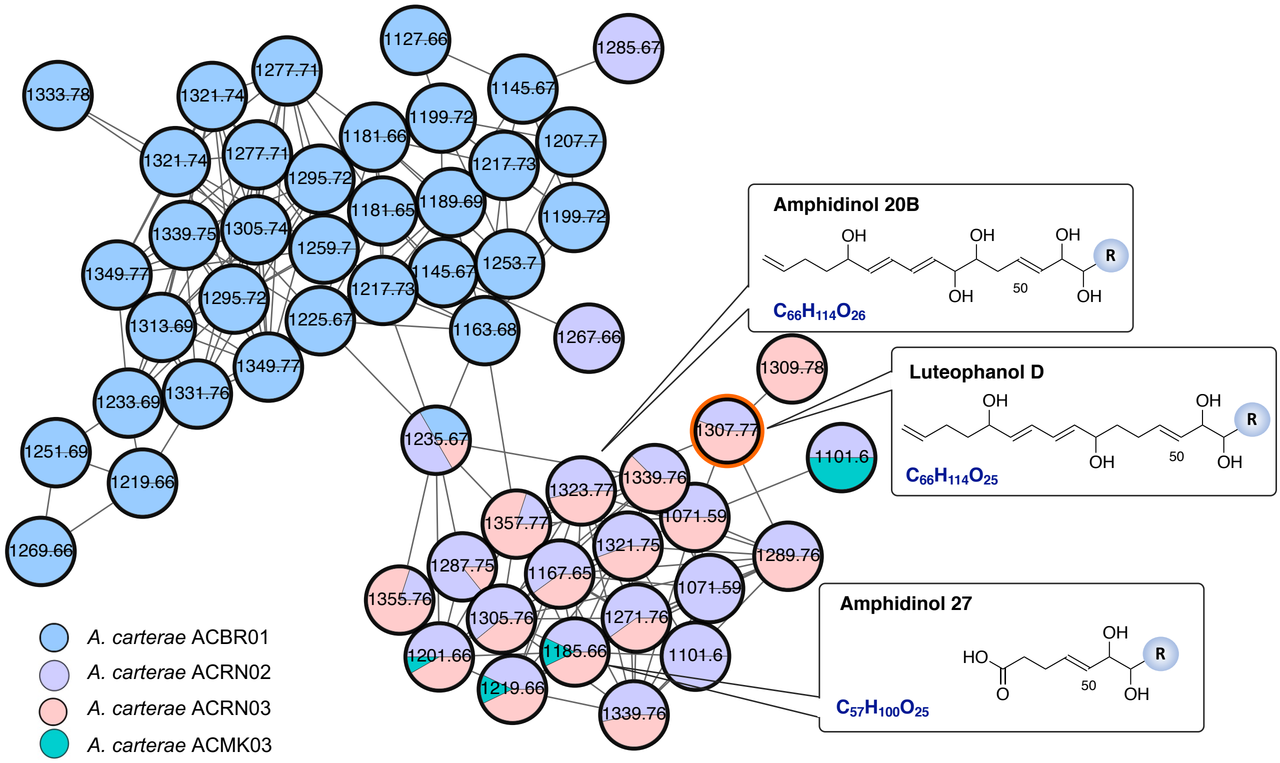

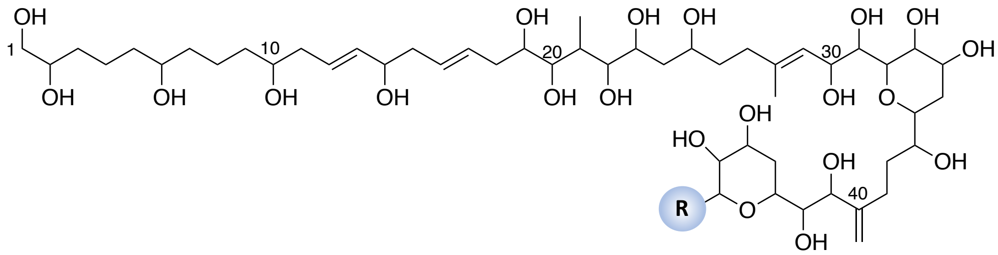

Scheme S3. General workflow for the identification and structural study of amphidinols in this study.

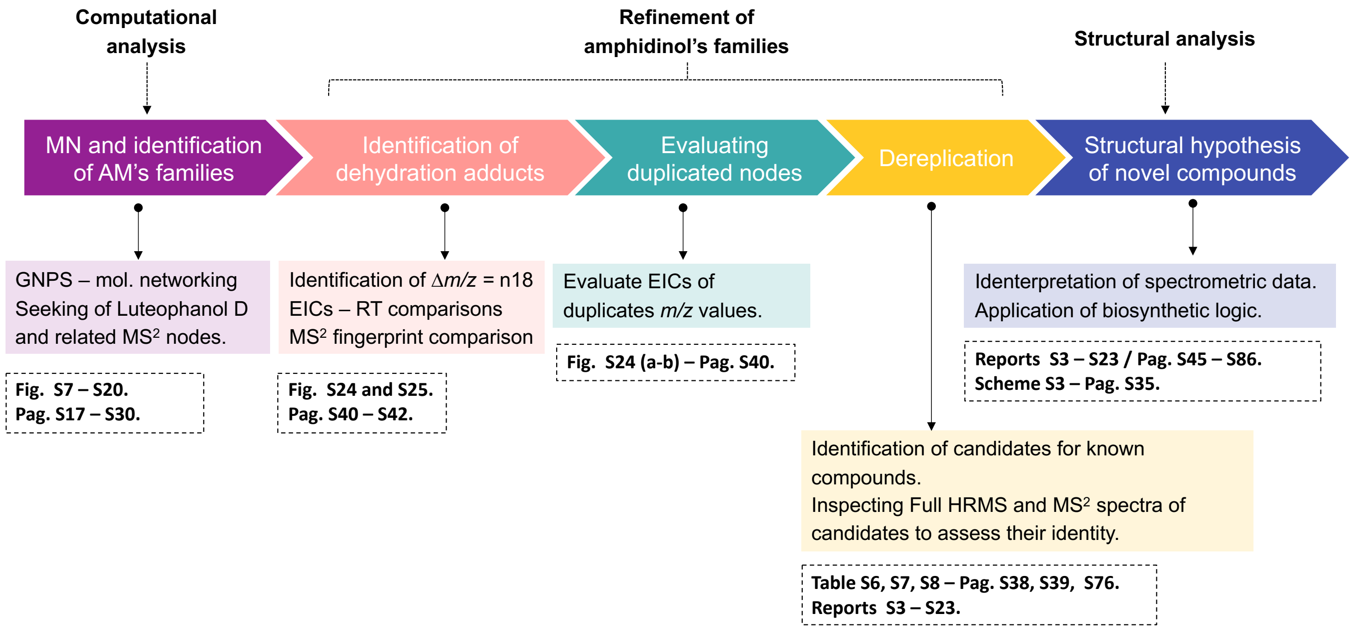

S5. Identification of amphidinol-analogs and structural study

Positive ion mode (ESI+) as [M+H]+ ions

Figure S23. Family of amphidinols in cell-free medium extract of **ACRB01**.
Colored by retention time.

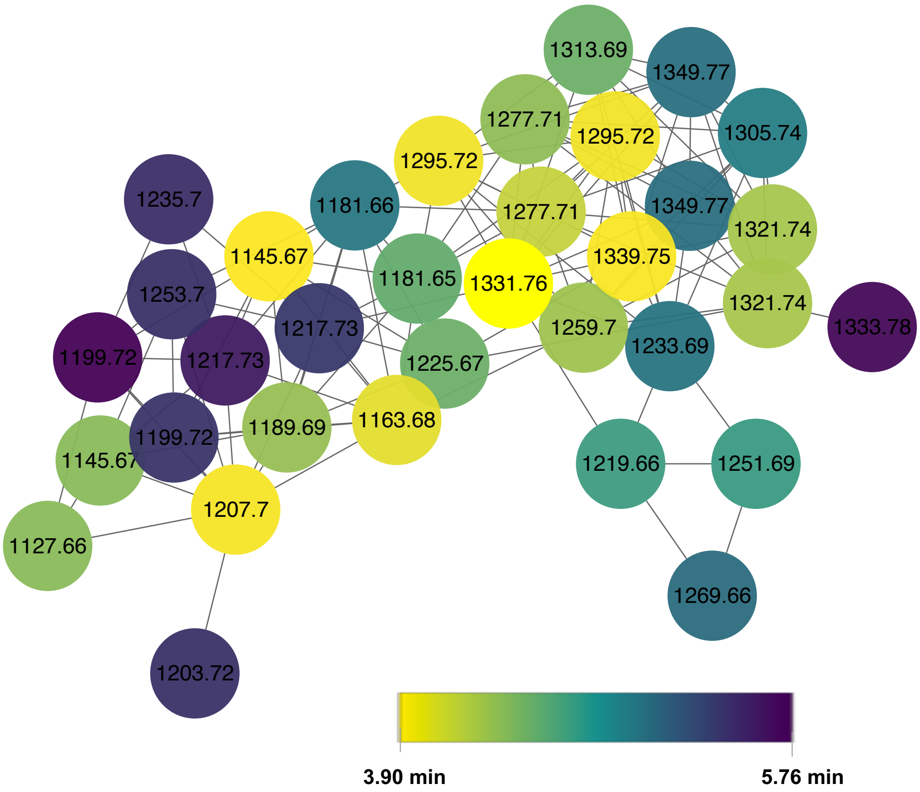

Table S8. Dereplication of potential amphidinols in **ACBR01**.

Precursor Mass	*m/z*	[M+H]+ -1.01 Da	[M+Na]+ -22.99 Da	[M+K]+ -39.10 Da	[M+ACN]+ -41.05 Da	1st Candidate
**1349.77**	+1	1348.76	**1326.78**	1310.67	1308.72	**Amphidinol 9**
**1349.77**	+1	1348.76	**1326.78**	1310.67	1308.72	**Amphidinol 3**
**1339.75**	+1	1338.74	1316.76	**1300.65**	1298.70	
**1333.78**	+1	1332.77	1310.79	1294.68	1292.73	
**1331.76**	+1	1330.75	1308.77	1292.66	1290.71	
**1321.74**	+1	**1320.73**	1298.75	**1282.64**	1280.69	**Luteophanol-B/C**
**1321.74**	+1	**1320.73**	1298.75	**1282.64**	1280.69	**Luteophanol-B/C**
**1313.69**	+1	1312.68	1290.70	1274.59	**1272.64**	**Lingshuiol-A**
**1305.75**	+1	1304.74	**1282.76**	1266.65	1264.70	**Amphidinol 17**
**1295.72**	+1	1294.71	**1272.73**	1256.62	**1254.67**	
**1295.72**	+1	1294.71	**1272.73**	1256.62	**1254.67**	
**1277.71**	+1	1276.70	**1254.72**	1238.61	1236.66	**Luteophanol-A**
**1277.71**	+1	1276.70	**1254.72**	1238.61	1236.66	**Luteophanol-A**
**1269.66**	+1	1268.65	1246.67	1230.56	1228.61	
**1259.70**	+1	1258.69	1236.71	**1220.60**	1218.65	**Luteophanol-B/C**
**1253.70**	+1	1252.69	1230.71	1214.60	1212.65	
**1251.69**	+1	1250.68	1228.70	1212.59	1210.64	
**1235.70**	+1	1234.69	1212.71	1196.60	1194.65	
**1233.69**	+1	1232.68	1210.70	1194.59	1192.64	
**1225.67**	+1	1224.66	1202.68	1186.57	1184.62	
**1217.73**	+1	1216.72	1194.74	1178.63	1176.68	
**1217.73**	+1	1216.72	1194.74	1178.63	1176.68	
**1219.66**	+1	1218.65	1196.67	1180.56	1178.61	
**1207.70**	+1	1206.69	1184.71	1168.60	1166.65	
**1203.72**	+1	1202.71	1180.73	1164.62	**1162.67**	**Amphidinol 15**
**1199.72**	+1	1198.71	1176.73	1160.62	1158.67	
**1199.72**	+1	1198.71	1176.73	1160.62	1158.67	
**1189.69**	+1	1188.68	1166.70	1150.59	1148.64	
**1181.66**	+1	1180.65	1158.67	1142.56	1140.61	
**1181.65**	+1	1180.64	1158.66	1142.55	1140.60	
**1163.68**	+1	**1162.67**	1140.69	1124.58	1122.63	**Amphidinol 15**
**1145.67**	+1	1144.66	1122.68	1106.57	1104.62	
**1145.67**	+1	1144.66	1122.68	1106.57	1104.62	
**1127.66**	+1	1126.65	1104.67	1088.56	1086.61	

Figure S24. Family of amphidinols of cell-free medium extract of **ACRN02**.
Colored by retention time

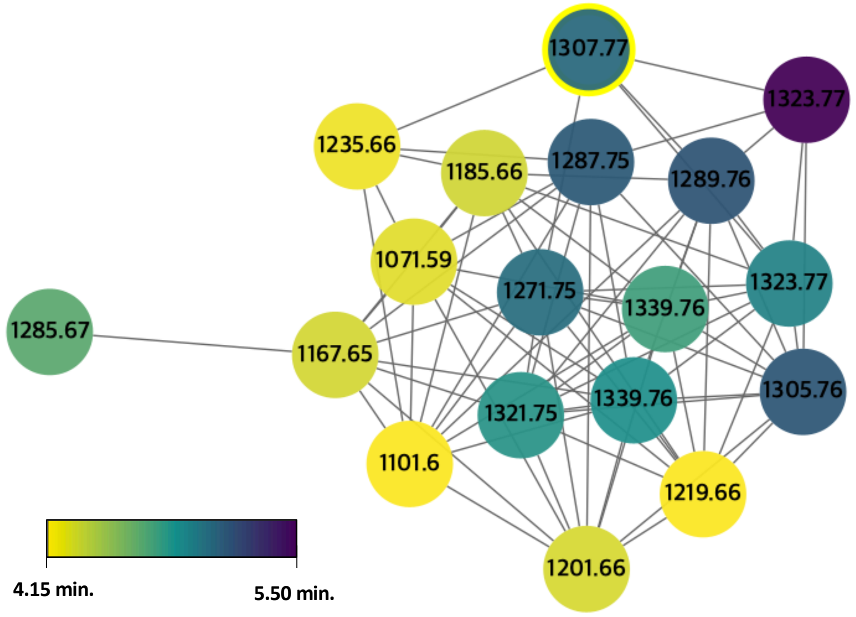

Table S9. Dereplication of potential amphidinols in **ACRN02**.

Parental Mass	*m/z*	[M+H]+ - 1.01 Da	[M+Na]+ - 22.99 Da	[M+K]+ - 39.10 Da	[M+ACN]+ - 41.05 Da	1st Candidate
1339.76	+1	1338.75	1316.77	**1300.66**	1298.71	**Amphidinol 4**
1339.76	+1	1338.75	1316.77	**1300.66**	1298.71	**Amphidinol 4**
1323.77	+1	**1322.76**	**1300.78**	1284.67	**1282.72**	**Amphidinol 20/4**
1323.77	+1	**1322.76**	**1300.78**	1284.67	1282.72	**Amphidinol 20/4**
1321.75*	+1	**1320.74**	1298.76	1282.65	1280.70	**Luteophanol B/C**
1307.77	+1	**1306.76**	1284.78	1268.67	1266.72	**Luteophanol D**
1305.76*	+1	1304.75	**1282.77**	1266.66	1264.71	**Amphidinol 17**
1289.76*	+1	1288.75	1266.77	1250.66	1248.71	
1287.75*	+1	1286.74	1264.76	1248.65	1246.70	
1285.67*	+1	1284.66	1262.68	1246.57	1244.62	
1271.75*	+1	1270.74	1248.76	1232.65	1230.70	
1235.66	+1	1234.65	1212.67	1196.56	1194.61	
1219.66	+1	1218.65	1196.67	1180.56	1178.61	
1201.66	+1	**1200.65**	1178.67	**1162.56**	1160.61	**Colopsinol-C**
1185.66	+1	1184.65	**1162.67**	1146.56	1144.61	**Amphidinol 15**
1167.65*	+1	1166.64	1144.66	1128.55	1126.60	
1101.60	+1	1100.59	1078.61	1062.50	1060.55	
1071.59	+1	1070.58	1048.60	1032.49	1030.54	

NOTE: Entries marked with the "*" symbol are dehydrated ion from other ion.

Figure S25. EIC comparison between potential dehydrated protonated ions.
Comparison of retention times (RT) and Δ*m/z*. The leaders of series are identified with diamond shape

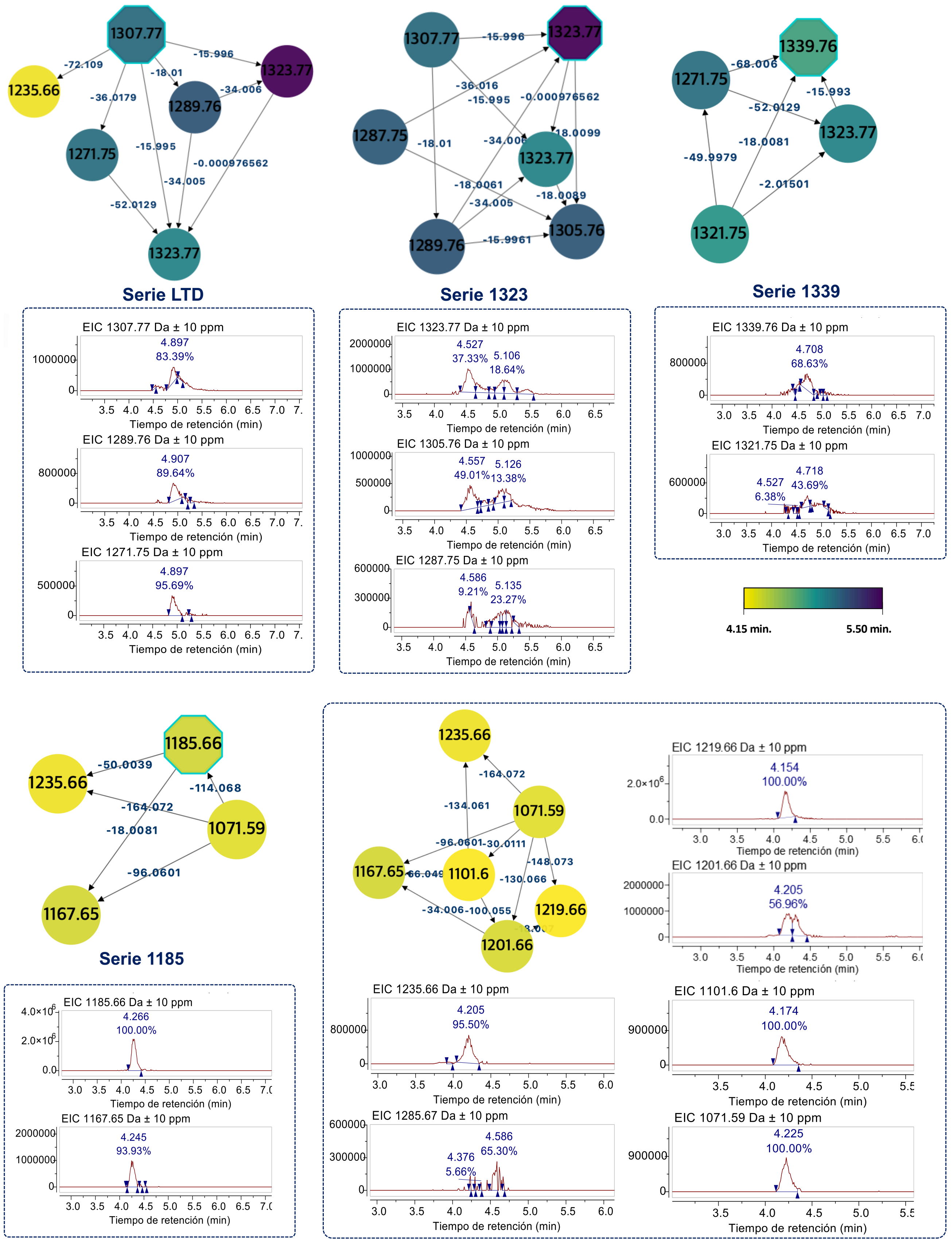

Figure S26 (a-d). MS^2 comparison between potential dehydrated protonated ions.

a. Luteophanol D serie:
[*m/z* 1307 vs. 1271)

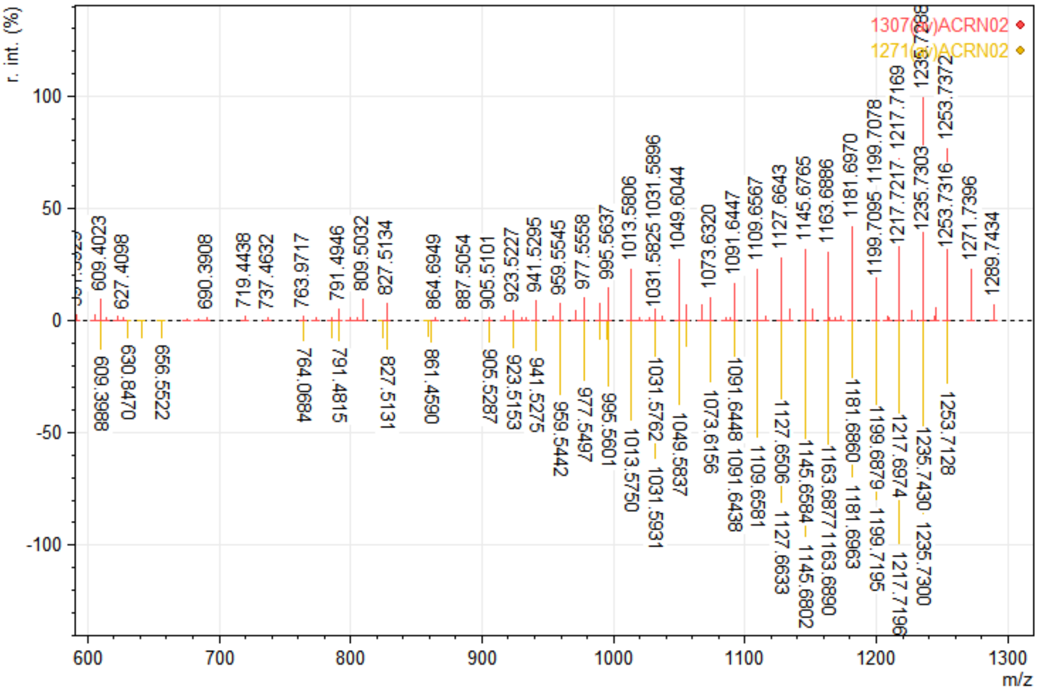

b. Amphidinol 20B serie:
[*m/z* 1323 vs 1305]

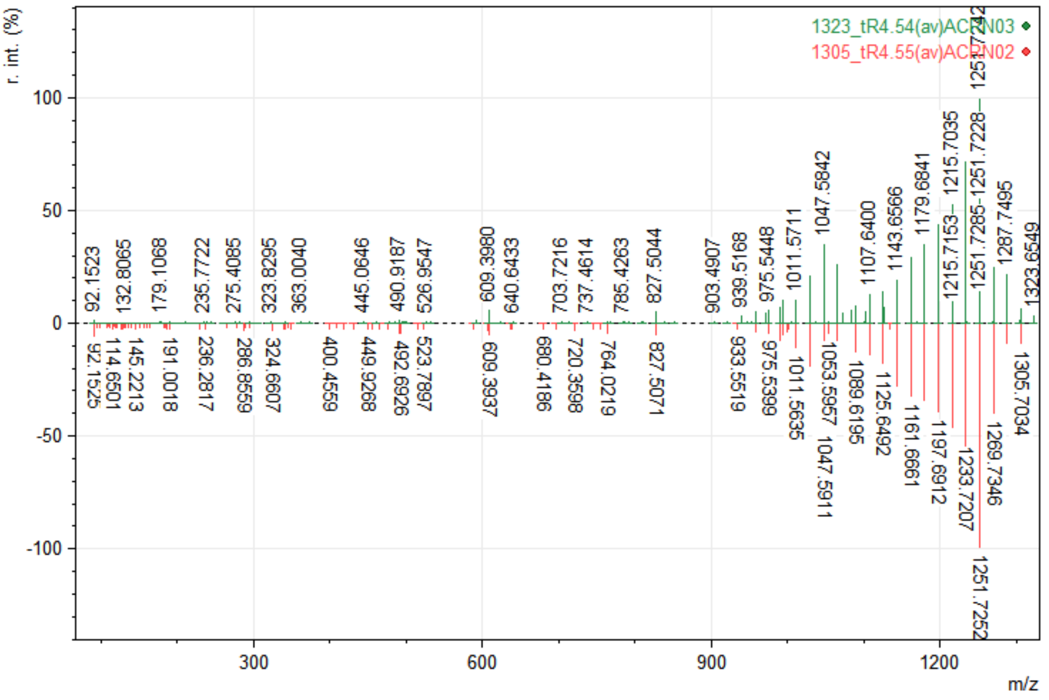

[*m/z* 1323 vs 1287]

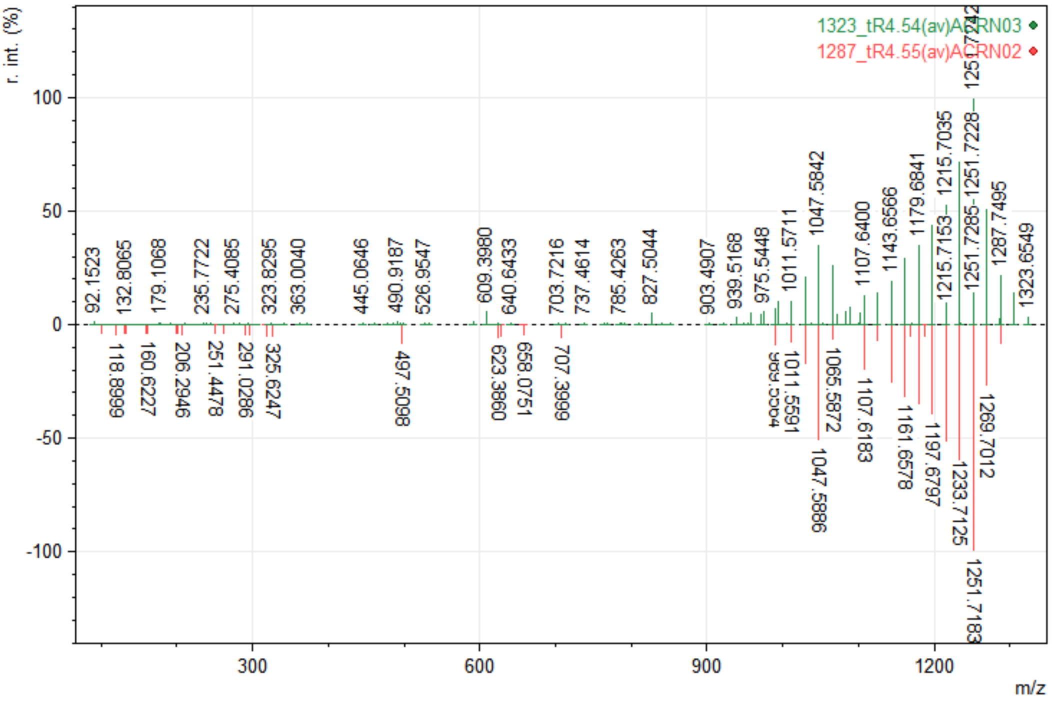

c. Amphidinol 29 serie:
[*m/z* 1339 vs 1321]

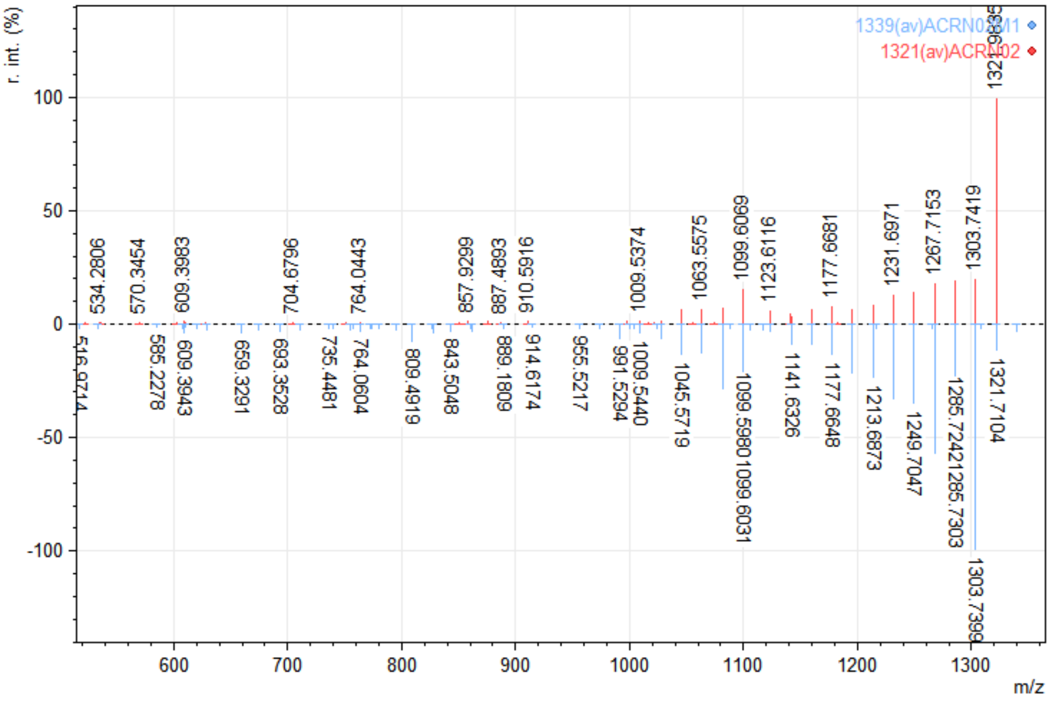

d. Amphidinol 27 serie:
[*m/z* 1185 vs 1167]:

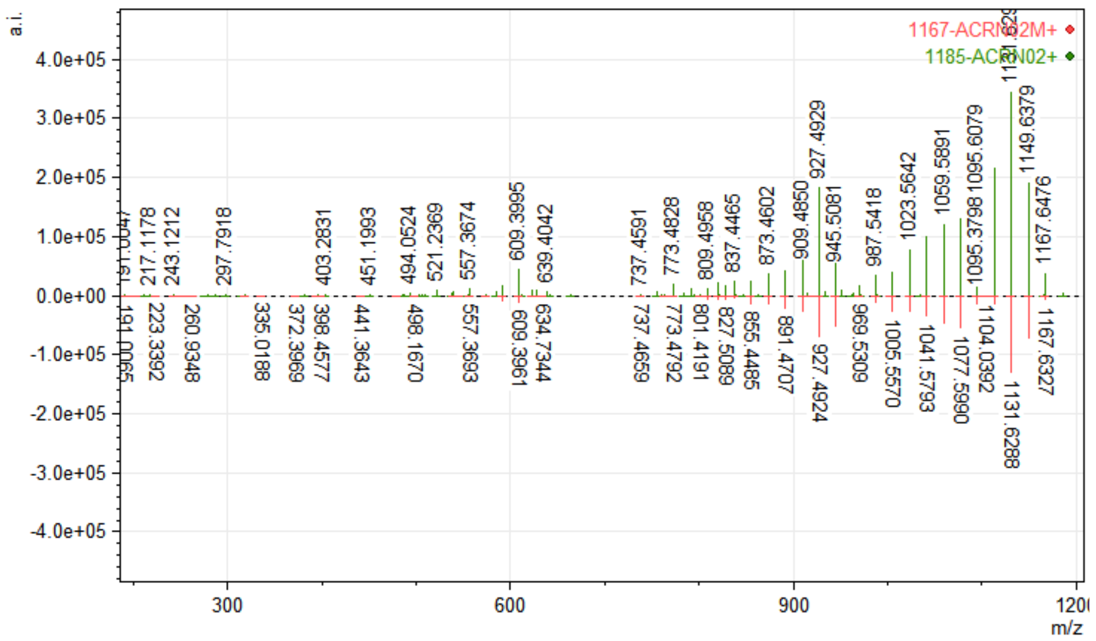

Report S5. Characterization of **luteophanol D** (*m/z* 1307.77) in **ACRN02**.

Properties

**Exact Mass**	1306.7649 g/mol
**Ion [M+H]⁺**	*m/z* 1307.7731
**Formula**	$C_{66}H_{114}O_{25}$
**RT**	4.90 min
**C LogP**	–8.1384

Full HRMS Characterization

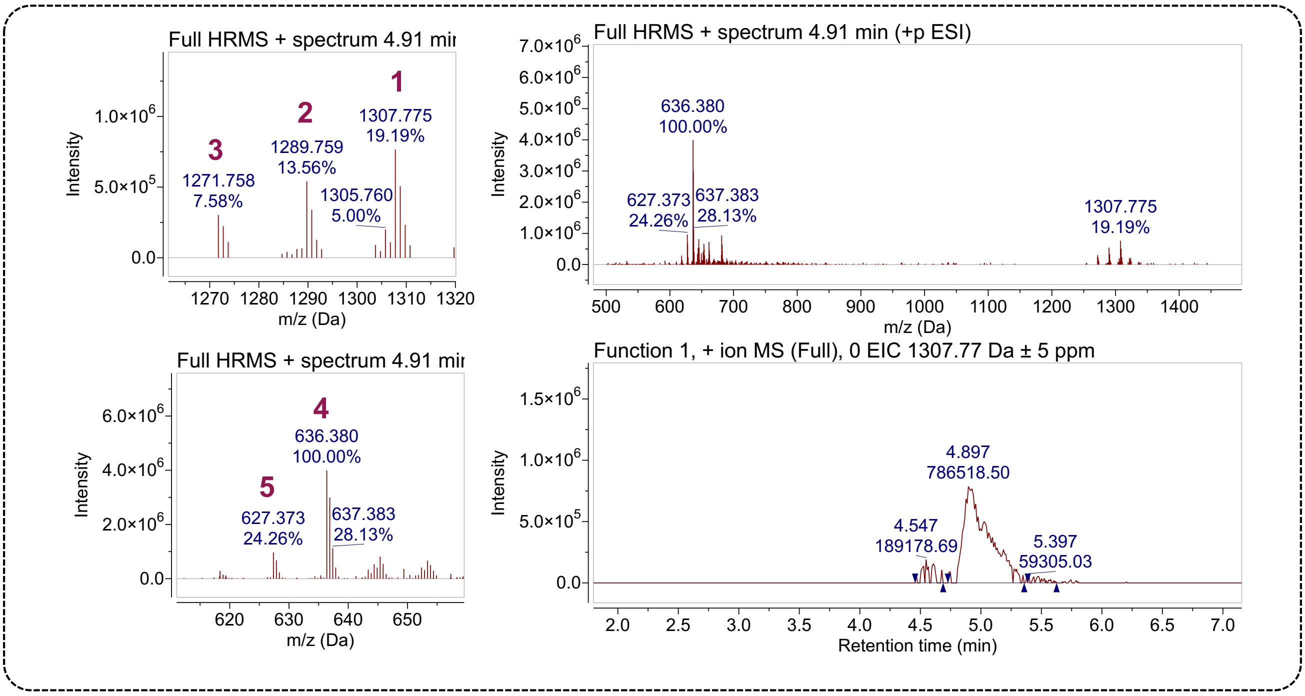

	Ion	Formula	*m/z* theo.	*m/z* exp.	RDB	Δ Da	Δ ppm
**1**	$[M+H]^+$	$C_{66}H_{115}O_{25}^+$	1307.7722	1307.7731	9.5	0.88	0.67
**2**	$[M+H–H_2O]^+$	$C_{66}H_{113}O_{24}^+$	1289.7616	1289.7618	10.5	0.21	0.16
**3**	$[M+H–2H_2O]^+$	$C_{66}H_{111}O_{23}^+$	1271.7511	1271.7516	11.5	0.52	0.41
**4**	$[M+2H–2H_2O]^{2+}$	$C_{66}H_{113}O_{23}^{2+}$	636.3792	636.3797	11.0	1.06	0.83
**5**	$[M+2H–3H_2O]^{2+}$	$C_{66}H_{111}O_{22}^{2+}$	627.3739	627.3734	12.0	–0.95	–0.76

MS^2 fragments annotation

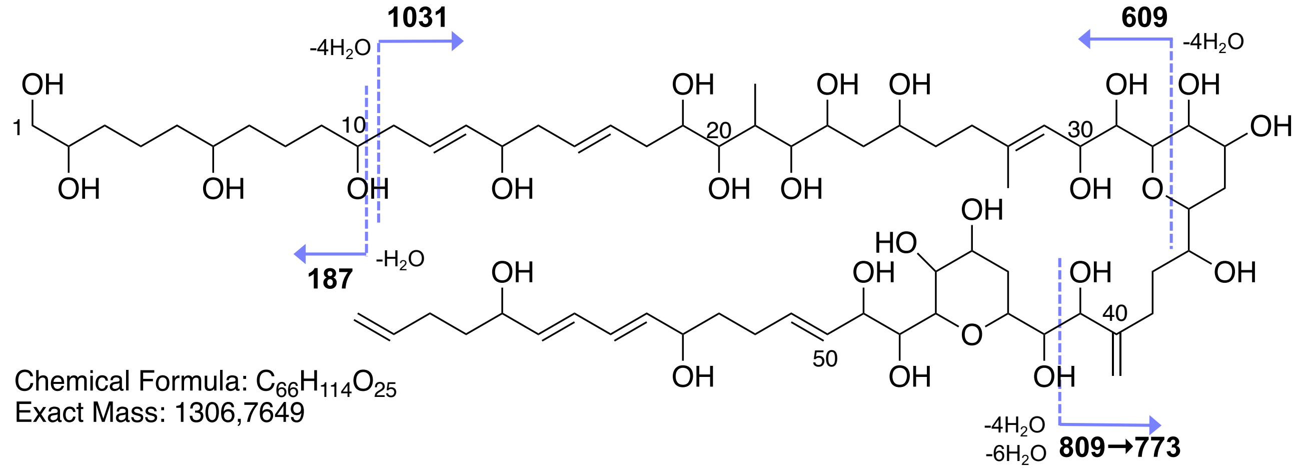

	clv	Ion	Formula	*m/z* theo.	*m/z* exp.	RDB	Δ ppm	Neutral loss (H_2O)
1		$[M+H–3H_2O]^+$	$C_{66}H_{107}O_{21}^+$	1235.7299	1235.7280	14.0	–1.55	12
2	a	$[X+H–4H_2O]^+$	$C_{56}H_{87}O_{17}^+$	1031.5938	1031.5889	14.0	–4.76	8
3	c	$[X+H–4H_2O]^+$	$C_{44}H_{73}O_{13}^+$	809.5046	809.5005	9.0	–5.05	5
4	c	$[X+H–6H_2O]^+$	$C_{44}H_{69}O_{11}^+$	773.4834	773.4816	11.0	–2.34	3
5	f	$[X+H–4H_2O]^+$	$C_{34}H_{57}O_9^+$	609.3997	609.3978	7.0	–3.19	2
6	k	$[X+H–H_2O]^+$	$C_{10}H_{19}O_3^+$	187.1329	187.1327	2.0	–0.89	3

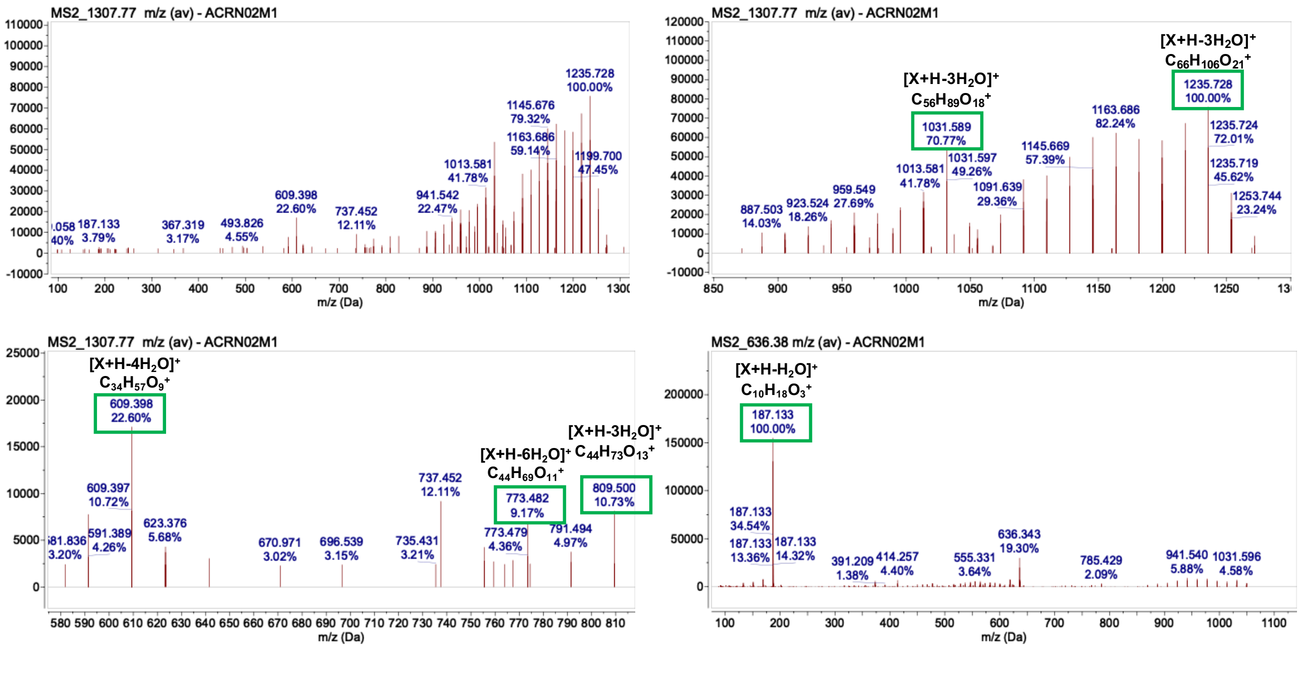

Report S6. Characterization of **amphidinol 28** (*m/z* 1323.77; RT 4.53) in **ACRN02**.

Properties

**Exact Mass**	1322.7598 g/mol
**Ion [M+H]⁺**	*m/z* 1323.7683
**Formula**	$C_{66}H_{114}O_{26}$
**RT**	4.53 min
**C LogP**	–9.4926

Full HRMS Characterization

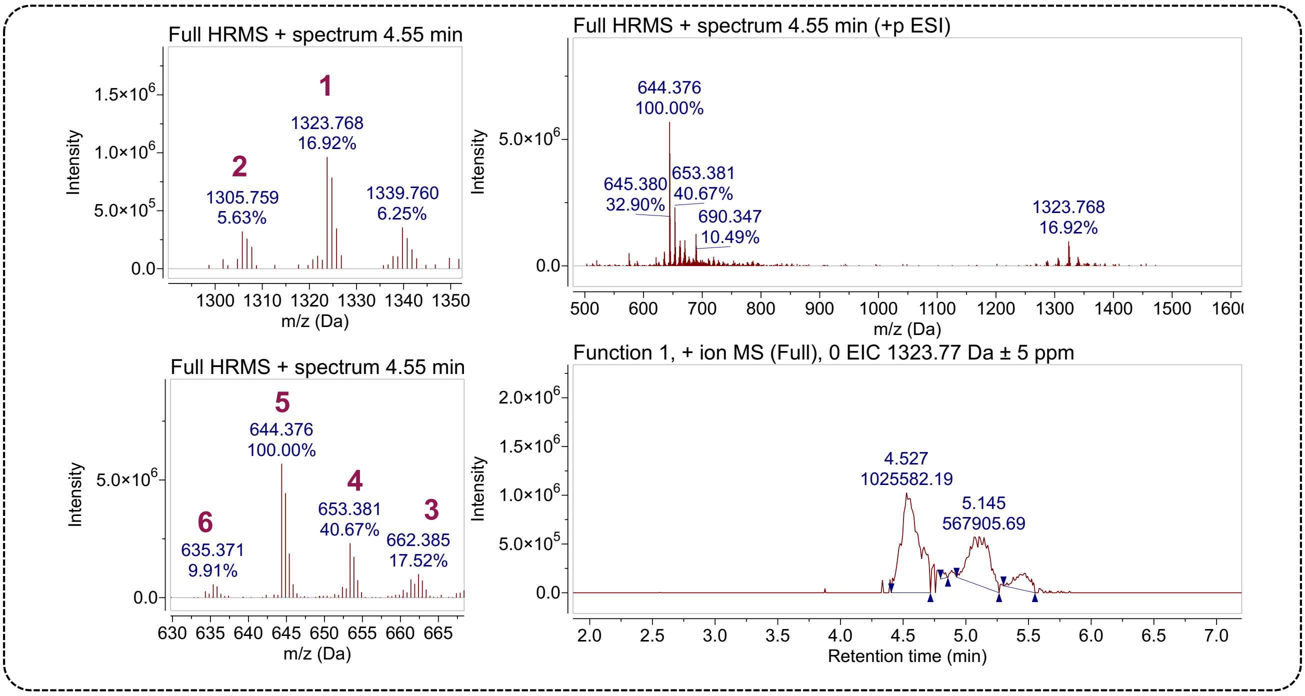

	Ion	Formula	*m/z* theo.	*m/z* exp.	RDB	Δ mDa	Δ ppm
**1**	$[M+H]^+$	$C_{66}H_{115}O_{26}^+$	1323.7671	1323.7683	9.5	1.20	0.91
**2**	$[M+H–H_2O]^+$	$C_{66}H_{113}O_{25}^+$	1305.7566	1305.7593	10.5	2.73	2.09
**3**	$[M+2H]^{2+}$	$C_{66}H_{116}O_{26}^{2+}$	662.3872	662.3854	9.0	–3.51	–2.65
**4**	$[M+2H–H_2O]^{2+}$	$C_{66}H_{114}O_{25}^{2+}$	653.3819	653.3808	10.0	–2.23	–1.70
**5**	$[M+2H–2H_2O]^{2+}$	$C_{66}H_{112}O_{24}^{2+}$	644.3767	644.3760	11.0	–1.18	–0.92
**6**	$[M+2H–3H_2O]^{2+}$	$C_{66}H_{110}O_{23}^{2+}$	635.3714	635.3712	12.0	–0.26	–0.21

MS² fragments annotation

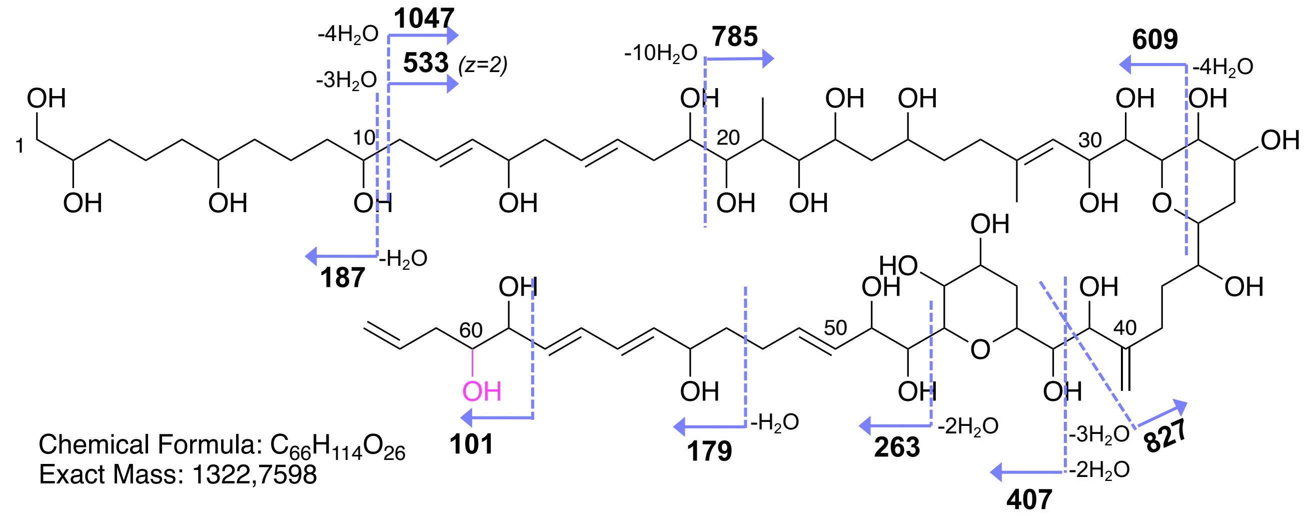

NOTE: The functional groups shown in red are located in the most likely position according to our approach and hypothesis described in the article.

	clv	Ion	Formula	*m/z* theo.	*m/z* exp.	RDB	Δ ppm	Neutral loss (H_2O)
1		$[M+H–4H_2O]^+$	$C_{66}H_{107}O_{22}^+$	1251.7249	1251.7222	13.5	–2.15	11
2	a	$[X+H–4H_2O]^+$	$C_{56}H_{87}O_{18}^+$	1047.5887	1047.5839	13.5	–4.62	7
3	c	$[X+H–3H_2O]^+$	$C_{44}H_{75}O_{14}^+$	827.5151	827.5081	7.5	–8.49	-
4		$[X+H–10H_2O]^+$	$C_{47}H_{61}O_{10}^+$	785.4259	785.4260	17.5	0.05	-
5		$[M+2H–4H_2O]^{2+}$	$C_{66}H_{108}O_{22}^{2+}$	626.3661	626.3660	12.5	–0.06	9
6	f	$[X+H–4H_2O]^+$	$C_{34}H_{57}O_9^+$	609.3997	609.3997	6.5	–0.08	-
7		$[X+2H–3H_2O]^{2+}$	$C_{56}H_{90}O_{19}^{2+}$	533.3033	533.3029	12.0	–0.65	7
8	s	$[X+H–H_2O]^+$	$C_{22}H_{33}O_8^+$	425.2170	425.2182	6.5	2.77	3
9	s	$[X+H–2H_2O]^+$	$C_{22}H_{31}O_7^+$	407.2064	407.2052	7.5	–2.95	2
11	r	$[X+H–2H_2O]^+$	$C_{16}H_{23}O_3^+$	263.1642	263.1631	5.5	–4.14	-
12	k	$[X+H–H_2O]^+$	$C_{10}H_{19}O_3^+$	187.1329	187.1327	1.5	–0.89	3
13	p	$[X+H–H_2O]^+$	$C_{11}H_{15}O_2^+$	179.1067	179.1065	4.5	–0.93	-
14	n	$[X]^{\cdot}$	$C_5H_9O_2^{\cdot}$	101.0603	101.0601	1.0	–1.98	-

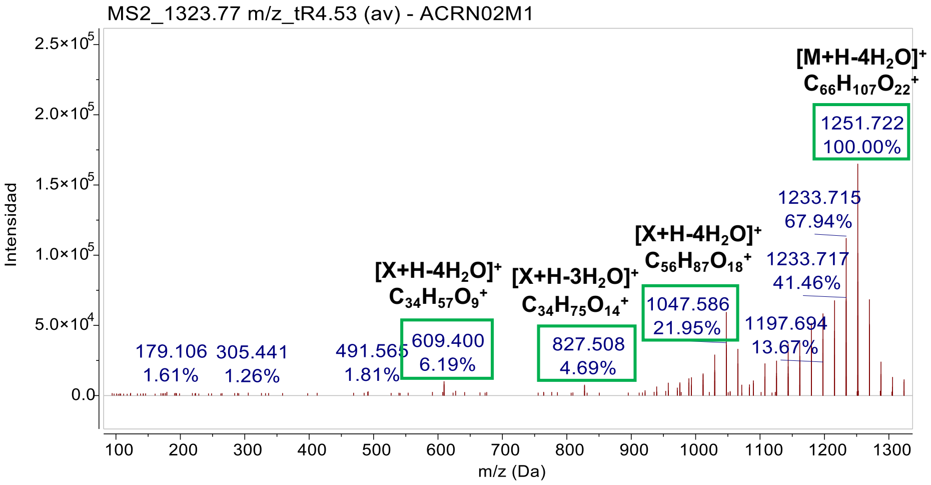
MS2_1323.77 m/z_tR4.53 (av) - ACRN02M1
Intensidad
m/z (Da)
[M+H-4H$_2$O]$^+$
C$_{66}$H$_{107}$O$_{22}$$^+$
1251.722
100.00%
1233.715
67.94%
1233.717
41.46%
[X+H-4H$_2$O]$^+$
C$_{56}$H$_{87}$O$_{18}$$^+$
1047.586
21.95%
1197.694
13.67%
[X+H-3H$_2$O]$^+$
C$_{34}$H$_{75}$O$_{14}$$^+$
827.508
4.69%
[X+H-4H$_2$O]$^+$
C$_{34}$H$_{57}$O$_9$$^+$
609.400
6.19%
179.106
1.61%
305.441
1.26%
491.565
1.81%

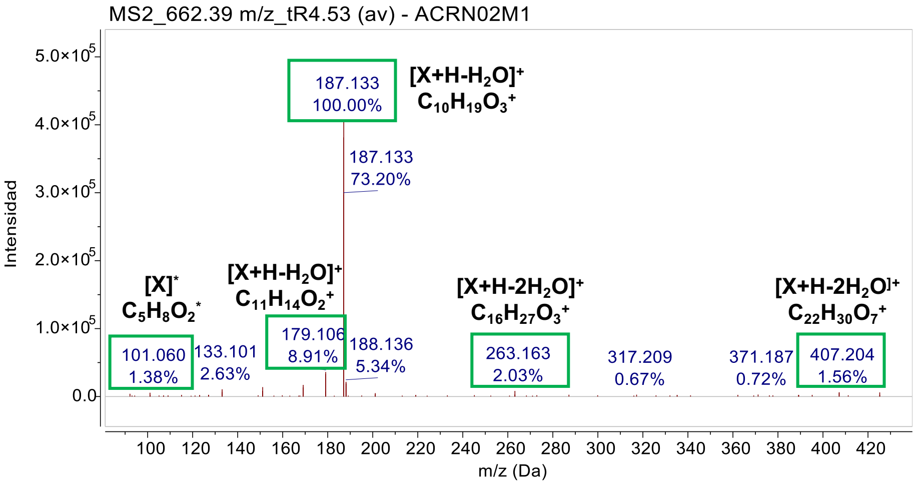
MS2_662.39 m/z_tR4.53 (av) - ACRN02M1
Intensidad
m/z (Da)
187.133
100.00%
[X+H-H$_2$O]$^+$
C$_{10}$H$_{19}$O$_3$$^+$
187.133
73.20%
[X]*
C$_5$H$_8$O$_2$*
101.060
1.38%
133.101
2.63%
[X+H-H$_2$O]$^+$
C$_{11}$H$_{14}$O$_2$$^+$
179.106
8.91%
188.136
5.34%
[X+H-2H$_2$O]$^+$
C$_{16}$H$_{27}$O$_3$$^+$
263.163
2.03%
317.209
0.67%
371.187
0.72%
[X+H-2H$_2$O]$^+$
C$_{22}$H$_{30}$O$_7$$^+$
407.204
1.56%

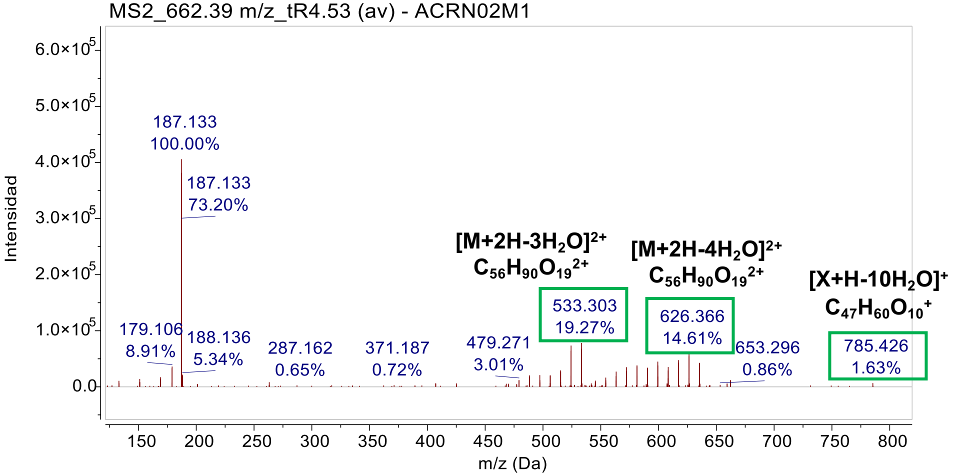
MS2_662.39 m/z_tR4.53 (av) - ACRN02M1
Intensidad
m/z (Da)
187.133
100.00%
187.133
73.20%
179.106
8.91%
188.136
5.34%
287.162
0.65%
371.187
0.72%
479.271
3.01%
[M+2H-3H$_2$O]$^{2+}$
C$_{56}$H$_{90}$O$_{19}$$^{2+}$
533.303
19.27%
[M+2H-4H$_2$O]$^{2+}$
C$_{56}$H$_{90}$O$_{19}$$^{2+}$
626.366
14.61%
653.296
0.86%
[X+H-10H$_2$O]$^+$
C$_{47}$H$_{60}$O$_{10}$$^+$
785.426
1.63%

Report S7. Characterization of **amphidinol 20B** (*m/z* 1323.77; RT 5.15) in **ACRN02**.

Properties

**Exact Mass**	1322.7598 g/mol
**Ion [M+H]+**	*m/z* 1323.7673
**Formula**	$C_{66}H_{114}O_{26}$
**RT**	5.15 min
**C LogP**	–9.4926

Full HRMS Characterization

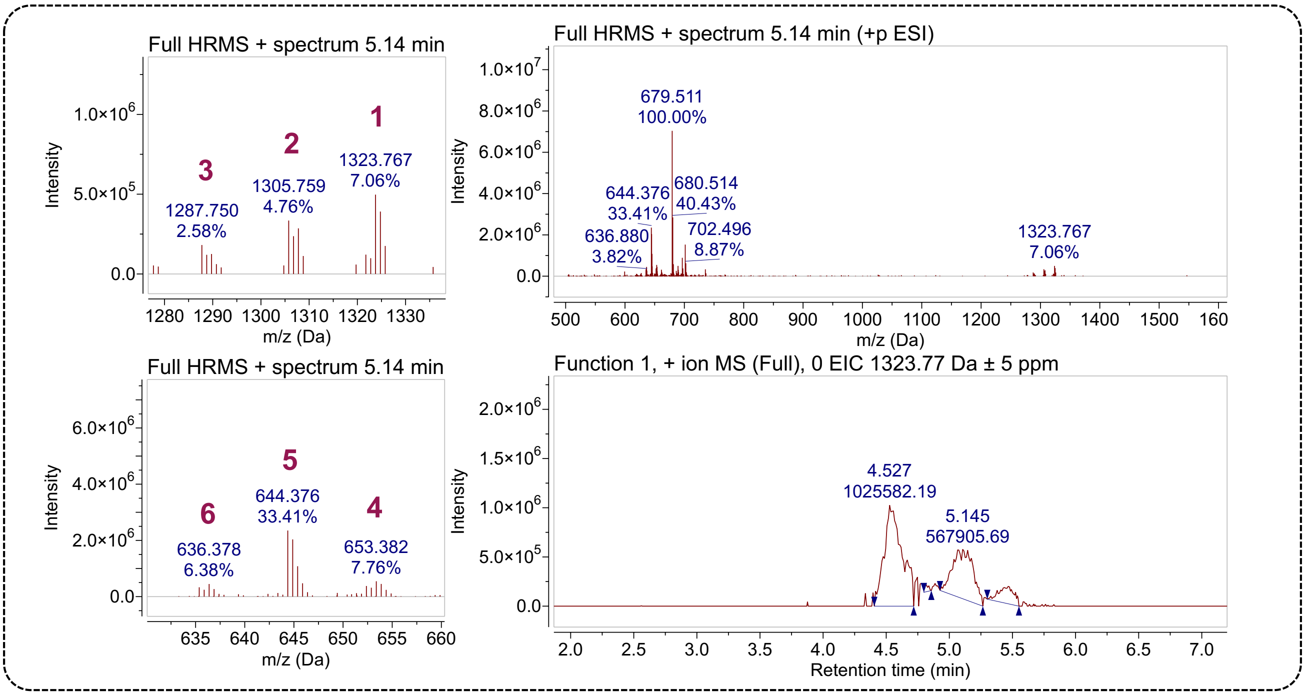

	Ion	Formula	*m/z* theo.	*m/z* exp.	RDB	Δ mDa	Δ ppm
**1**	$[M+H]^+$	$C_{66}H_{115}O_{26}^+$	1323.7671	1323.7673	9.5	0.22	0.17
**2**	$[M+H–H_2O]^+$	$C_{66}H_{113}O_{25}^+$	1305.7566	1305.7587	10.5	2.12	1.63
**3**	$[M+H–2H_2O]^+$	$C_{66}H_{111}O_{24}^+$	1287.7460	1287.7500	11.5	4.02	3.12
**4**	$[M+2H–H_2O]^{2+}$	$C_{66}H_{114}O_{25}^{2+}$	653.3819	653.3814	10.0	–1.13	–0.86
**5**	$[M+2H–2H_2O]^{2+}$	$C_{66}H_{112}O_{24}^{2+}$	644.3767	644.3762	11.0	–0.94	–0.73
**6**	$[M+2H–3H_2O]^{2+}$	$C_{66}H_{110}O_{23}^{2+}$	636.3792	636.3778	11.0	–2.85	–2.24

MS[2] fragments annotation

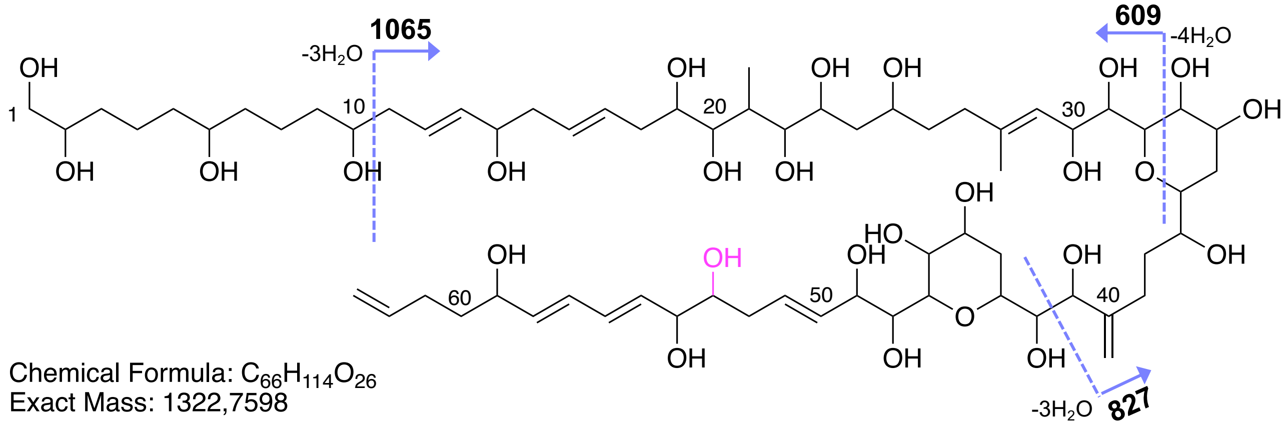

Chemical Formula: $C_{66}H_{114}O_{26}$
Exact Mass: 1322,7598

NOTE: The functional groups shown in red are located in the most likely position according to our approach and hypothesis described in the article.

	clv	Ion	Formula	*m/z* theo.	*m/z* exp.	RDB	Δ ppm	Neutral loss (H_2O)
1		$[M+H–3H_2O]^+$	$C_{66}H_{109}O_{23}^+$	1269.7354	1269.7341	12.5	–1.01	10
2	a	$[X+H–3H_2O]^+$	$C_{56}H_{89}O_{19}^+$	1065.5993	1065.6006	12.5	1.25	7
3	c	$[X+H–3H_2O]^+$	$C_{44}H_{75}O_{14}^+$	827.5151	827.5128	7.5	–2.88	4
4	f	$[X+H–4H_2O]^+$	$C_{34}H_{57}O_9^+$	609.3997	609.3961	6.5	–6.00	1

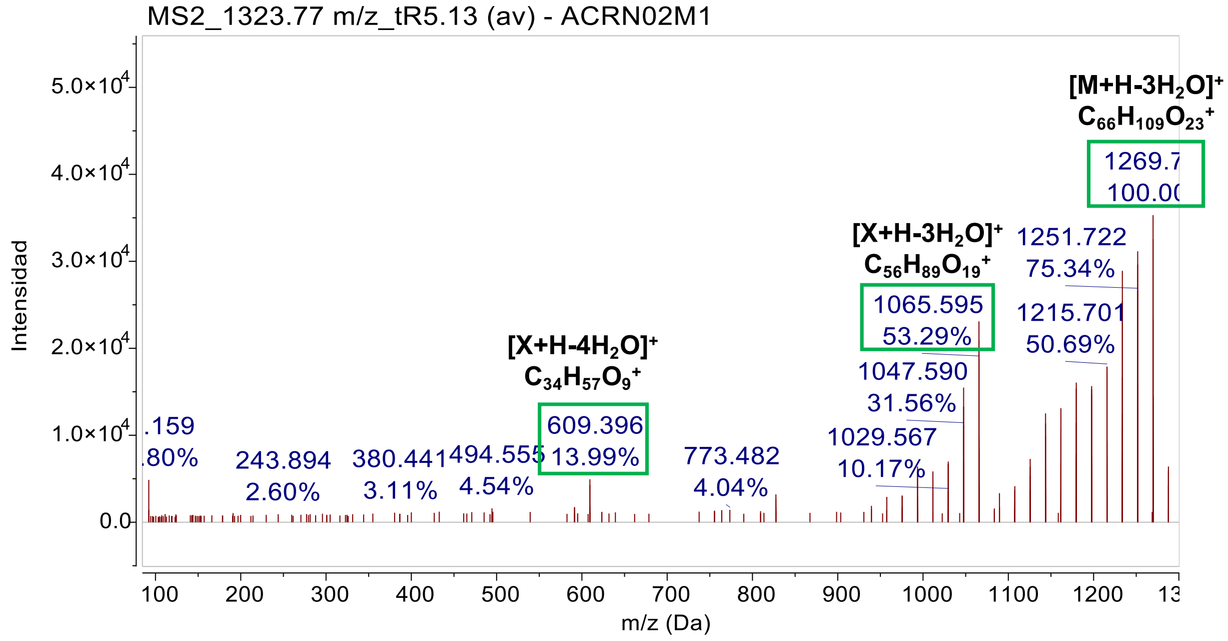

MS^2 comparison with 1323.77 RT 4.53 min

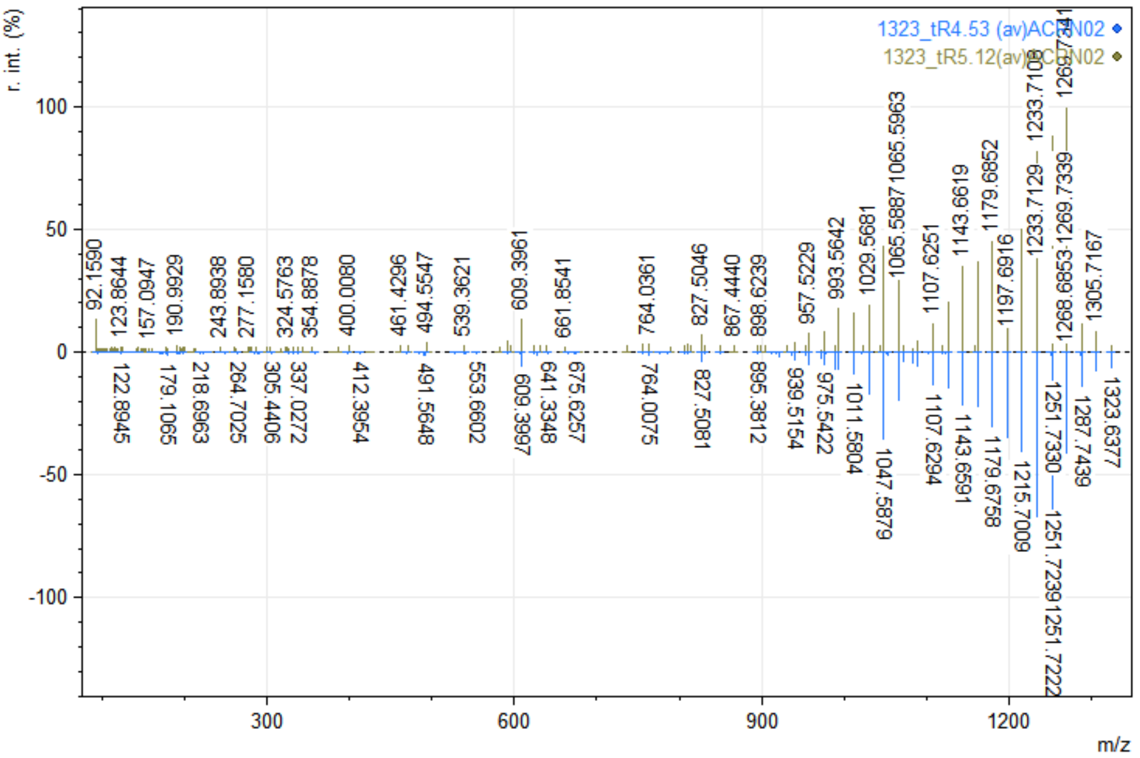

Report S8. Characterization of **amphidinol 29** (*m/z* 1339.76) in **ACRN02.**

Properties

**Exact Mass**	1338.7547 g/mol
**Ion $[M+H]^+$**	*m/z* 1339.7631
**Formula**	$C_{66}H_{114}O_{27}$
**RT**	4.70
**C LogP**	–10.8468

Full HRMS Characterization

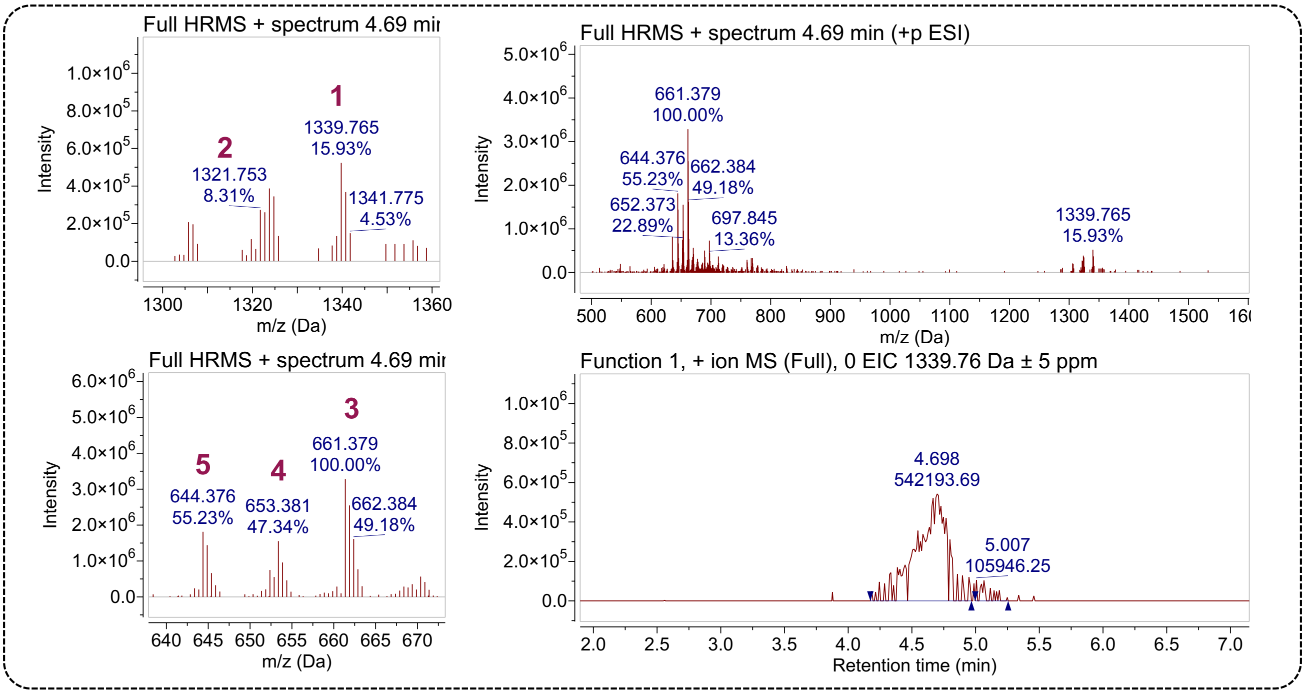

	Ion	Formula	*m/z* theo.	*m/z* exp.	RDB	Δ mDa	Δ ppm
**1**	$[M+H]^+$	$C_{66}H_{115}O_{27}^+$	1339.7620	1339.7631	9.5	1.04	0.77
**2**	$[M+H–H_2O]^+$	$C_{66}H_{113}O_{26}^+$	1321.7515	1321.7539	10.5	2.45	1.85
**3**	$[M+2H–H_2O]^{2+}$	$C_{66}H_{114}O_{26}^{2+}$	661.3793	661.3787	10.0	1.41	–1.07
**4**	$[M+2H–2H_2O]^{2+}$	$C_{66}H_{112}O_{25}^{2+}$	653.3819	653.3805	11.0	–2.84	–2.17
**5**	$[M+2H–3H_2O]^{2+}$	$C_{66}H_{110}O_{24}^{2+}$	644.3767	644.3764	12.0	–0.45	–0.35

MS^2 fragments annotation

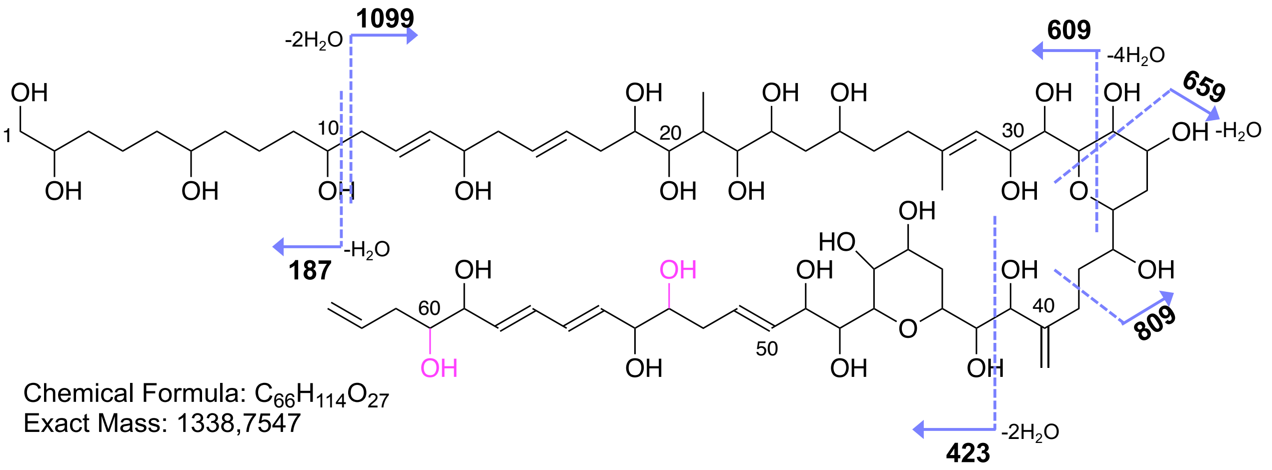

Chemical Formula: $C_{66}H_{114}O_{27}$
Exact Mass: 1338,7547

NOTE: The functional groups shown in red are located in the most likely position according to our approach and hypothesis described in the article.

	clv	Ion	Formula	*m/z* theo.	*m/z* exp.	RDB	Δ ppm	Neutral loss (H_2O)
1		$[M+H–H_2O]^+$	$C_{66}H_{111}O_{25}^+$	1303.7409	1303.7399	11.5	–0.79	12
2	a	$[X+H–2H_2O]^+$	$C_{56}H_{91}O_{21}^+$	1099.6047	1099.6032	11.5	–1.44	8
3	a	$[X+H–3H_2O]^+$	$C_{56}H_{89}O_{20}^+$	1081.5942	1081.5890	12.5	–4.80	7
4		$[X+H]^+$	$C_{40}H_{73}O_{16}^+$	809.4893	809.4919	4.5	3.18	-
5		$[X+H–H_2O]^+$	$C_{32}H_{51}O_{14}^+$	659.3273	659.3263	7.5	–1.58	-
6	f	$[X+H–4H_2O]^+$	$C_{34}H_{57}O_9^+$	609.3997	609.3944	6.5	–8.81	-
7	s	$[X+H–2H_2O]^+$	$C_{22}H_{31}O_8^+$	423.2013	423.1988	7.5	–6.05	-
8	k	$[X+H–H_2O]^+$	$C_{10}H_{19}O_3^+$	187.1329	187.1327	1.5	–1.13	3

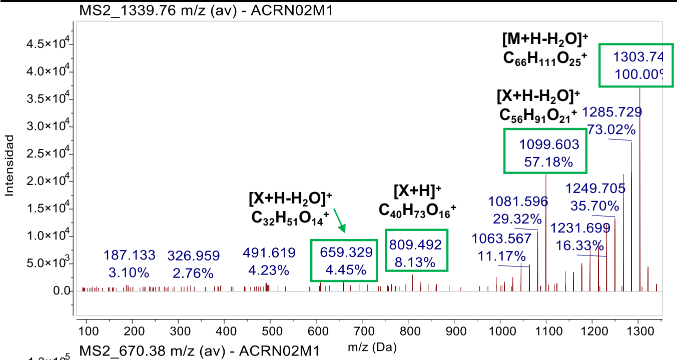

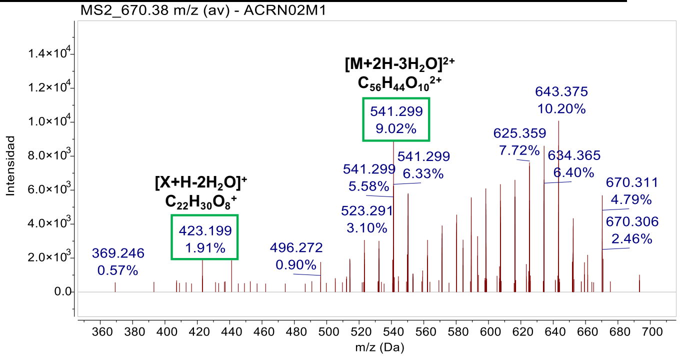

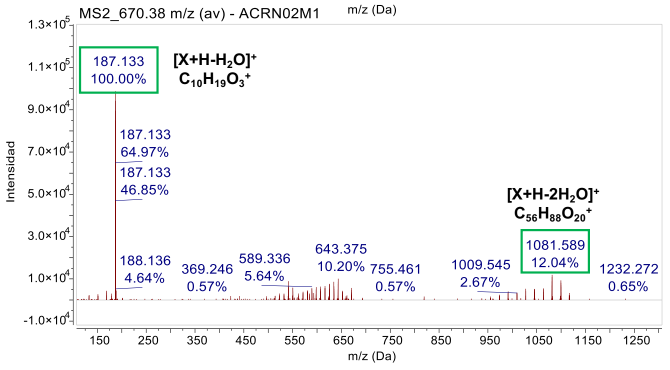

Report S9. Characterization of ion *m/z* 1321.76 (amphidinol 29) in **ACRN02**.

Properties

**Exact Mass**	1320.7442 g/mol
**Ion [M+H]+**	*m/z* 1321.7538
**Formula**	$C_{66}H_{112}O_{26}$
**RT**	4.72
**C LogP**	–9.7166

Full HRMS Characterization

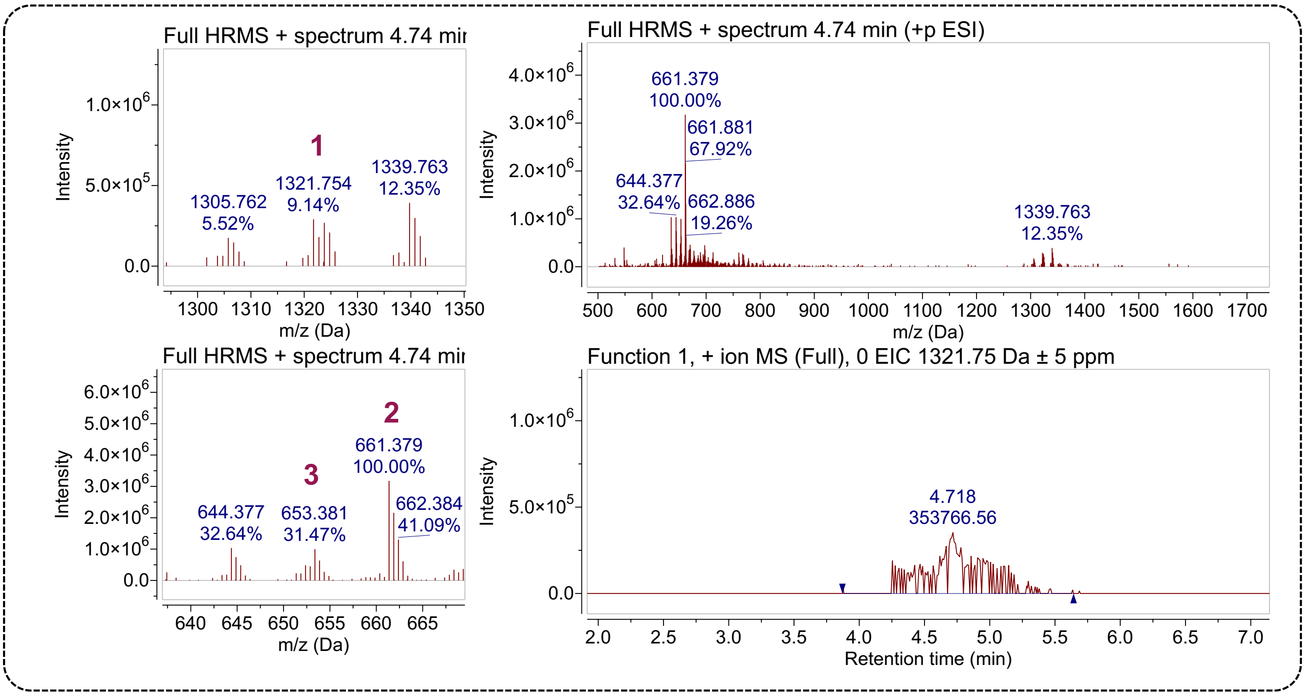

	Ion	Formula	*m/z* theo.	*m/z* exp.	RDB	Δ mDa	Δ ppm
**1**	$[M+H]^+$	$C_{66}H_{113}O_{26}^+$	1321.7515	1321.7538	10.5	2.32	1.76
**2**	$[M+2H]^{2+}$	$C_{66}H_{114}O_{26}^+$	661.3793	661.3789	10.0	–1.05	–0.79
**3**	$[M+2H–H_2O]^{2+}$	$C_{66}H_{112}O_{25}^{2+}$	653.3819	653.3812	11.0	–1.37	–1.05

MS[2] fragments annotation

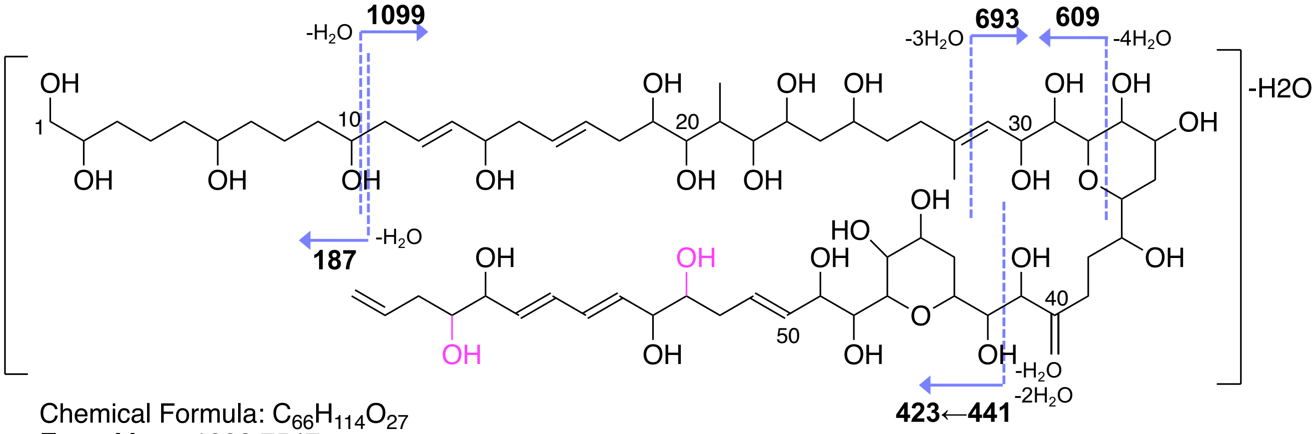

Chemical Formula: $C_{66}H_{114}O_{27}$
Exact Mass: 1338,7547

NOTE: The functional groups shown in red are located in the most likely position according to our approach and hypothesis described in the article.

	clv	Ion	Formula	*m/z* theo.	*m/z* exp.	RDB	Δ ppm	Neutral loss (H_2O)
1		$[M+H]^+$	$C_{66}H_{111}O_{26}^+$	1321.7515	1321.7509	11.5	0.80	10
2	a	$[X+H–H_2O]^+$	$C_{56}H_{91}O_{21}^+$	1099.6047	1099.6069	11.5	2.00	5
3		$[X+H–3H_2O]^+$	$C_{36}H_{53}O_{13}^+$	693.3481	693.3496	10.5	2.14	3
4	f	$[X+H–4H_2O]^+$	$C_{34}H_{57}O_9^+$	609.3997	609.3983	6.5	–2.39	-
5	a	$[X+2H–2H_2O]^{2+}$	$C_{56}H_{88}O_{20}^{2+}$	541.3007	541.2993	12.0	–8.03	3
6	s	$[X+H-H_2O]^+$	$C_{22}H_{31}O_9^+$	441.2119	441.2113	6.5	–1.44	4
7	s	$[X+H–2H_2O]^+$	$C_{22}H_{31}O_8^+$	423.2013	423.2003	7.5	–2.43	3
8	k	$[X+H–H_2O]^+$	$C_{10}H_{19}O_3^+$	187.1329	187.1326	1.5	–1.22	3

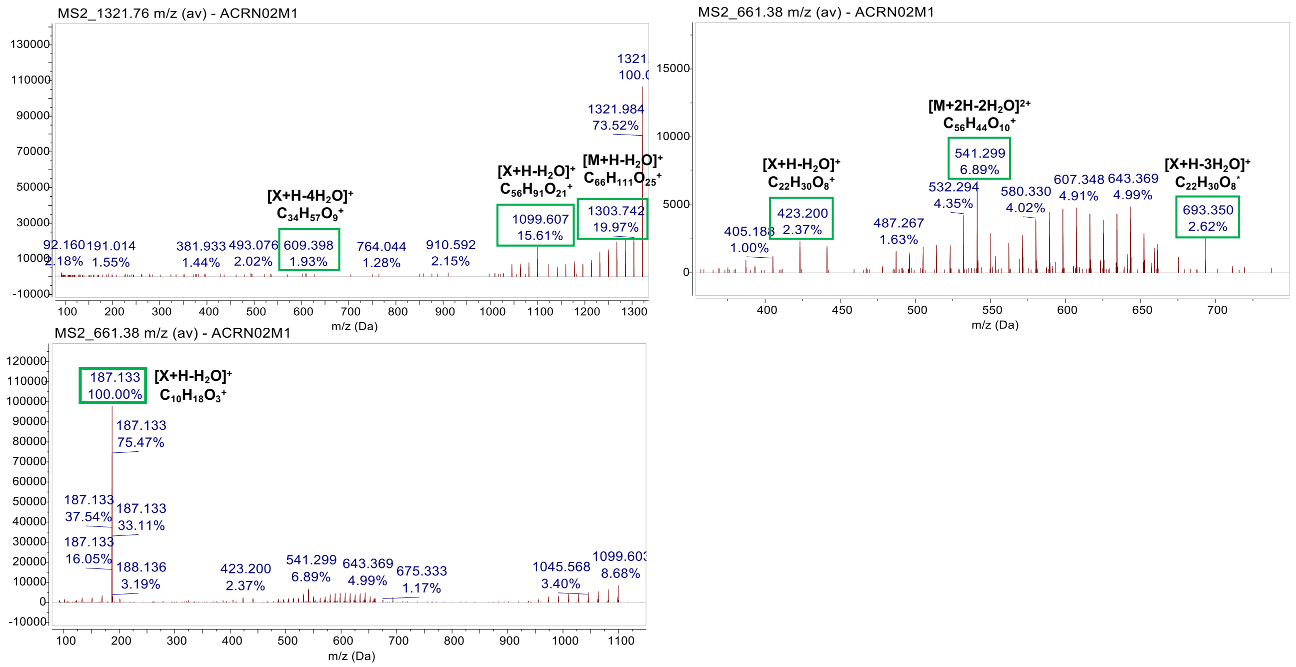

Report S10. Characterization of **amphidinol 27** (*m/z* 1185.66) in **ACRN02**.

Properties

**Exact Mass**	1184.6554 g/mol
**Ion [M+H]⁺**	*m/z* 1185.6602
**Formula**	$C_{57}H_{100}O_{25}$
**RT**	4.27
**C LogP**	–10.3056

Full HRMS Characterization

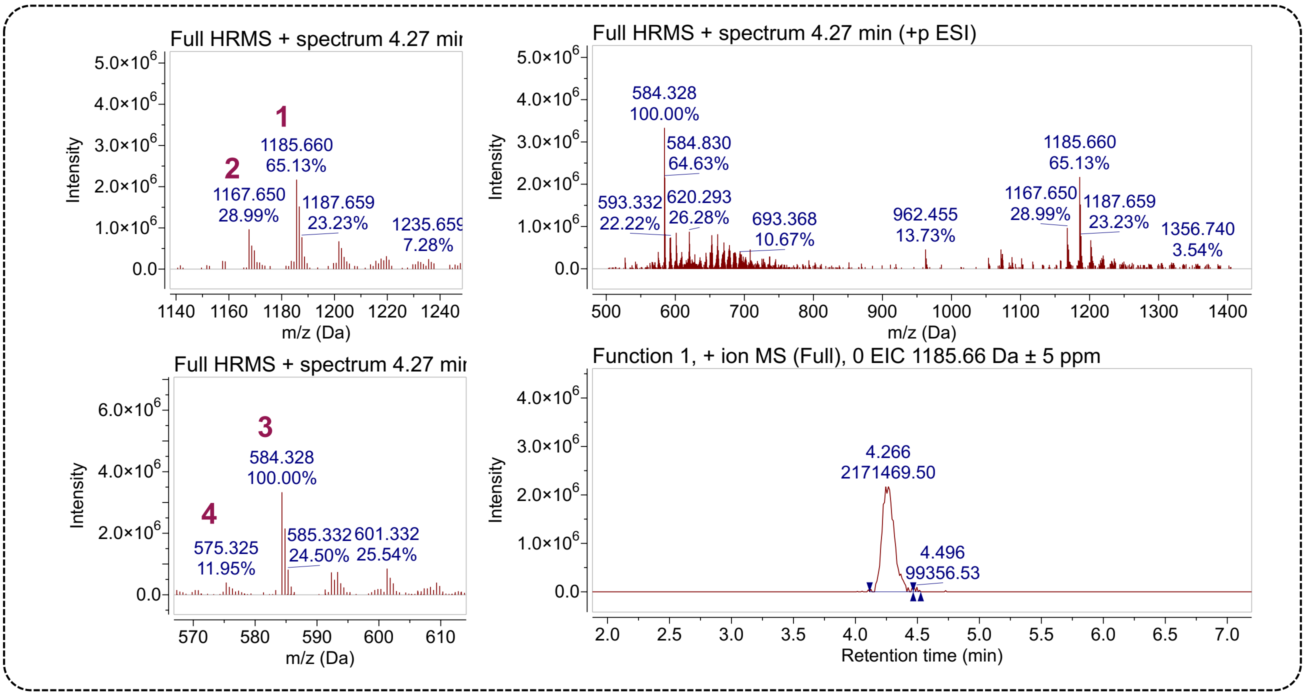

	**Ion**	**Formula**	***m/z* theo.**	***m/z* exp.**	**RDB**	**Δ mDa**	**Δ ppm**
**1**	$[M+H]^+$	$C_{57}H_{101}O_{25}^+$	1185.6627	1185.6602	7.5	–2.49	–2.10
**2**	$[M+H–H_2O]^+$	$C_{57}H_{99}O_{24}^+$	1167.6521	1167.6504	8.5	–1.69	–1.45
**3**	$[M+2H–H_2O]^{2+}$	$C_{57}H_{100}O_{24}^{2+}$	584.3297	584.3285	8.0	–2.37	–2.03
**4**	$[M+2H–2H_2O]^{2+}$	$C_{57}H_{98}O_{23}^{2+}$	575.3244	575.3247	9.0	0.50	0.43

MS[2] fragments annotation

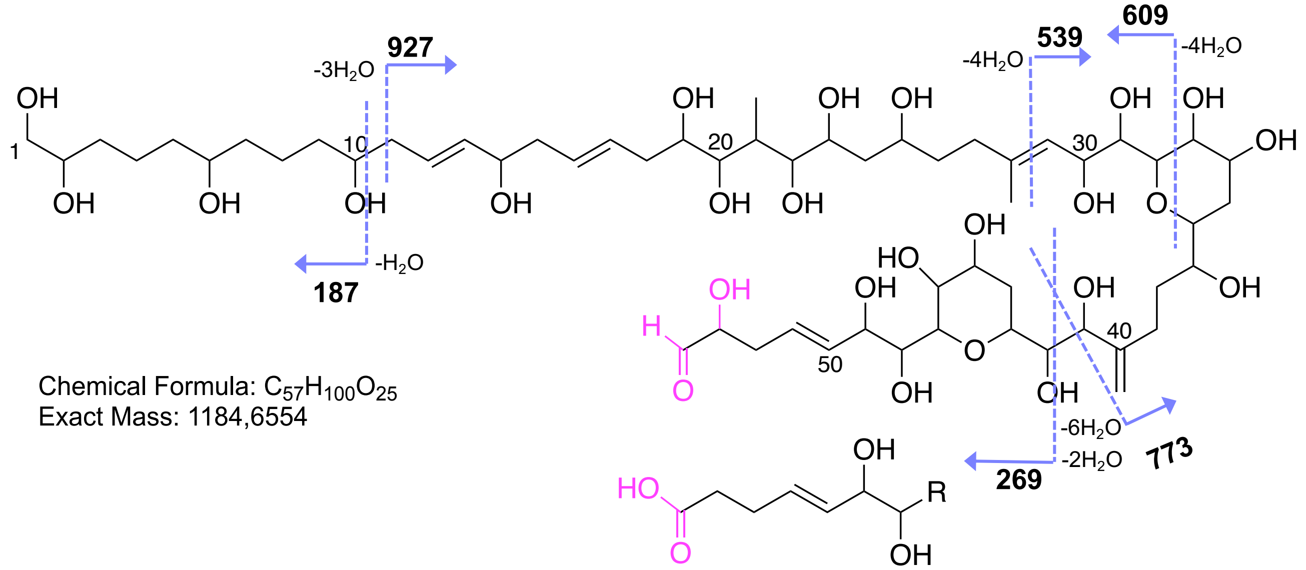

NOTE: The functional groups shown in red are located in the most likely position according to our approach and hypothesis described in the article.

	clv	Ion	Formula	*m/z* theo.	*m/z* exp.	RDB	Δ ppm	Neutral loss (H_2O)
1		$[M+H-3H_2O]^+$	$C_{57}H_{95}O_{22}^+$	1131.6310	1131.6294	10.5	–1.38	12
2	a	$[X+H-3H_2O]^+$	$C_{47}H_{75}O_{18}^+$	927.4948	927.4929	10.5	–2.02	8
3	c	$[X+H-6H_2O]^+$	$C_{44}H_{69}O_{11}^+$	773.4834	773.4829	10.5	–0.76	2
4	f	$[X+H-4H_2O]^+$	$C_{34}H_{57}O_9^+$	609.3997	609.3995	6.5	–0.39	3
5		$[X+H-4H_2O]^+$	$C_{27}H_{39}O_{11}^+$	539.2487	539.2488	8.5	0.28	3
6		$[X+H-5H_2O]^+$	$C_{27}H_{37}O_{10}^+$	521.2381	521.2369	9.5	–2.40	2
7	s	$[X+H-2H_2O]^+$	$C_{13}H_{17}O_6^+$	269.1020	269.1014	5.5	–2.07	-
8	k	$[X+H-H_2O]^+$	$C_{10}H_{19}O_3^+$	187.1329	187.1327	1.5	–0.17	3

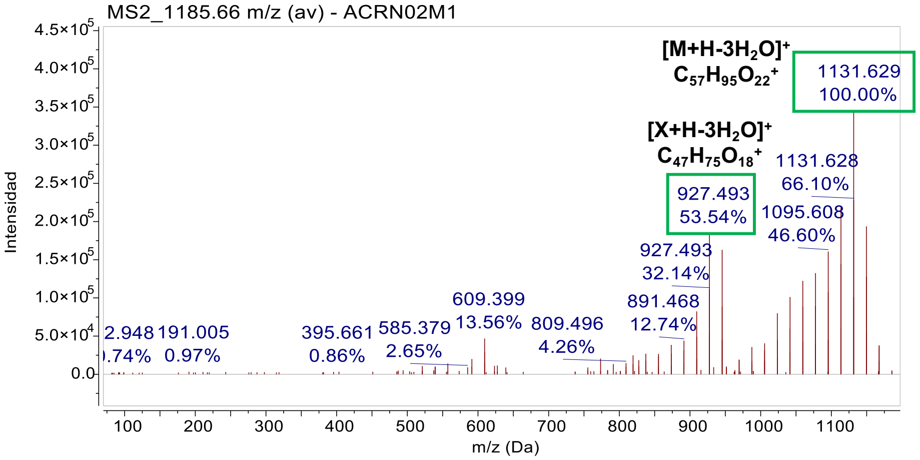

MS2_1185.66 m/z (av) - ACRN02M1
[M+H-3H2O]+
C57H95O22+
1131.629
100.00%
[X+H-3H2O]+
C47H75O18+
927.493
53.54%
1131.628
66.10%
1095.608
46.60%
927.493
32.14%
891.468
12.74%
609.399
13.56%
809.496
4.26%
585.379
2.65%
395.661
0.86%
191.005
0.97%
Intensidad
m/z (Da)

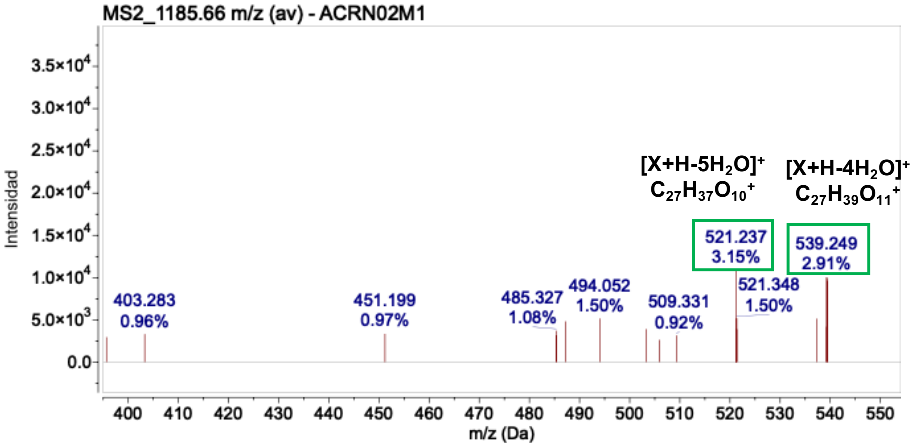

MS2_1185.66 m/z (av) - ACRN02M1
[X+H-5H2O]+
C27H37O10+
521.237
3.15%
[X+H-4H2O]+
C27H39O11+
539.249
2.91%
521.348
1.50%
509.331
0.92%
494.052
1.50%
485.327
1.08%
451.199
0.97%
403.283
0.96%
Intensidad
m/z (Da)

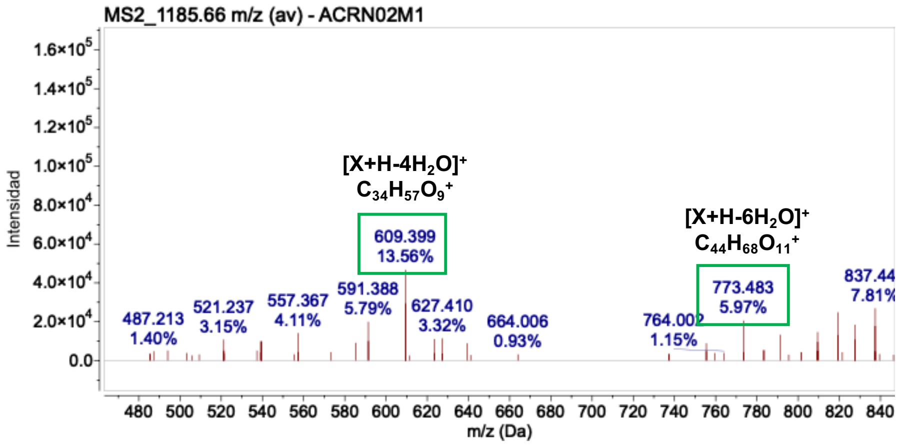

MS2_1185.66 m/z (av) - ACRN02M1
[X+H-4H2O]+
C34H57O9+
609.399
13.56%
[X+H-6H2O]+
C44H68O11+
773.483
5.97%
487.213
1.40%
521.237
3.15%
557.367
4.11%
591.388
5.79%
627.410
3.32%
664.006
0.93%
1.15%
Intensidad
m/z (Da)

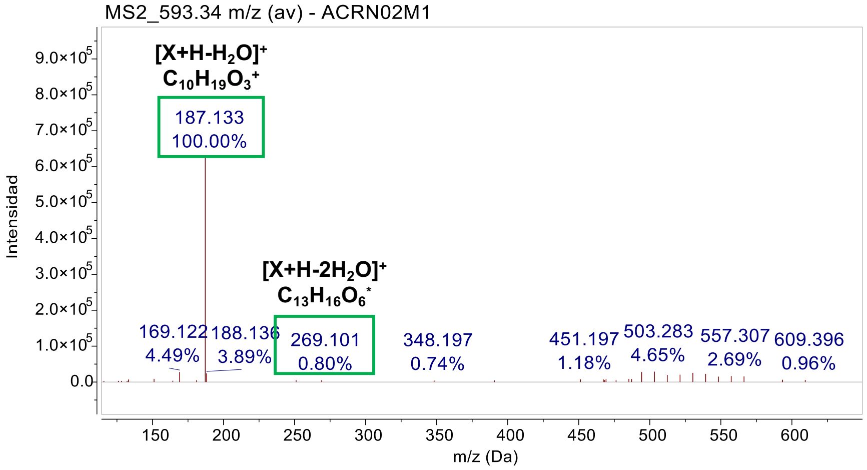

MS2_593.34 m/z (av) - ACRN02M1
[X+H-H2O]+
C10H19O3+
187.133
100.00%
[X+H-2H2O]+
C13H16O6+
269.101
0.80%
169.122
4.49%
188.136
3.89%
348.197
0.74%
451.197
1.18%
503.283
4.65%
557.307
2.69%
609.396
0.96%
Intensidad
m/z (Da)

Report S11. Characterization of ion *m/z* 1167.65 (amphidinol 27) in **ACRN02**.

Properties

**Exact Mass**	1166.6448 g/mol
**Ion [M+H]+**	*m/z* 1167.6480
**Formula**	$C_{57}H_{98}O_{24}$
**RT**	4.25
**C LogP**	–9.7234

Full HRMS Characterization

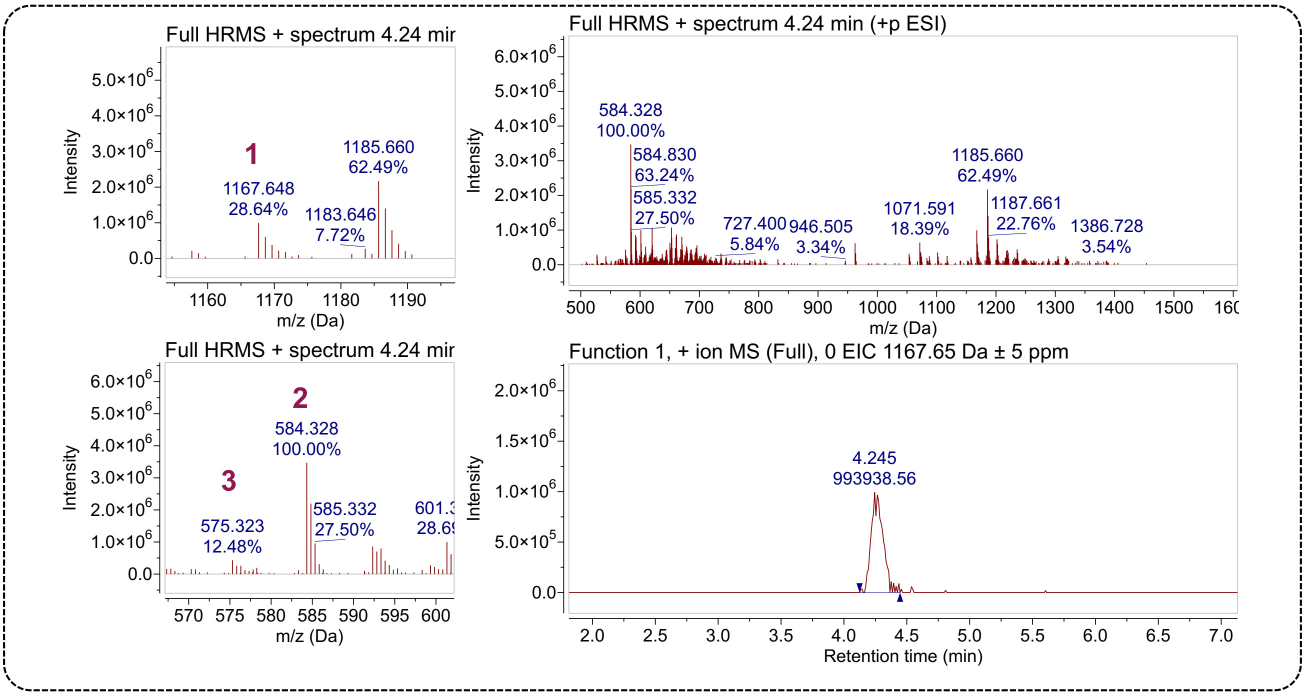

	**Ion**	**Formula**	***m/z* theo.**	***m/z* exp.**	**RDB**	**Δ mDa**	**Δ ppm**
**1**	$[M+H]^+$	$C_{57}H_{99}O_{24}^+$	1167.6521	1167.6480	9.0	–4.13	–3.54
**2**	$[M+2H]^{2+}$	$C_{57}H_{100}O_{24}^{2+}$	584.3297	584.3285	8.0	–2.37	–2.03
**3**	$[M+2H–H_2O]^{2+}$	$C_{57}H_{98}O_{23}^{2+}$	575.3244	575.3232	9.0	–2.31	–2.01

MS² fragments annotation

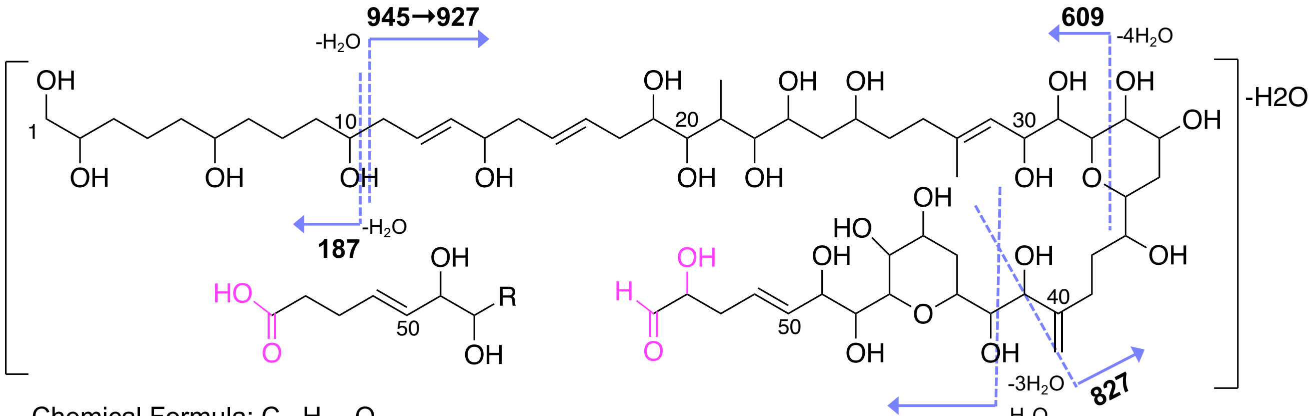

Chemical Formula: $C_{57}H_{100}O_{25}$
Exact Mass: 1184,6554

NOTE: The functional groups shown in red are located in the most likely position according to our approach and hypothesis described in the article.

	clv	Ion	Formula	*m/z* theo.	*m/z* exp.	RDB	Δ ppm	Neutral loss (H_2O)
1		$[M+H–2H_2O]^+$	$C_{57}H_{95}O_{22}^+$	1131.6310	1131.6288	11.0	–1.92	9
2	a	$[X+H–H_2O]^+$	$C_{47}H_{77}O_{19}^+$	945.5054	945.5054	10.0	0.08	8
3	a	$[X+H–2H_2O]^+$	$C_{47}H_{75}O_{18}^+$	927.4948	927.4924	11.0	–2.55	7
4	c	$[X+H–3H_2O]^+$	$C_{44}H_{75}O_{14}^+$	827.5151	827.5089	8.0	–7.53	1
5	f	$[X+H–4H_2O]^+$	$C_{34}H_{57}O_9^+$	609.3997	609.3961	7.0	–6.00	2
6	s	$[X+H–H_2O]^+$	$C_{13}H_{17}O_6^+$	269.1020	269.1016	6.0	–1.27	1
7	s	$[X+H–2H_2O]^+$	$C_{13}H_{15}O_5^+$	251.0914	251.0906	7.0	–3.05	-
8	k	$[X+H–H_2O]^+$	$C_{10}H_{19}O_3^+$	187.1329	187.1327	2.0	–1.05	3

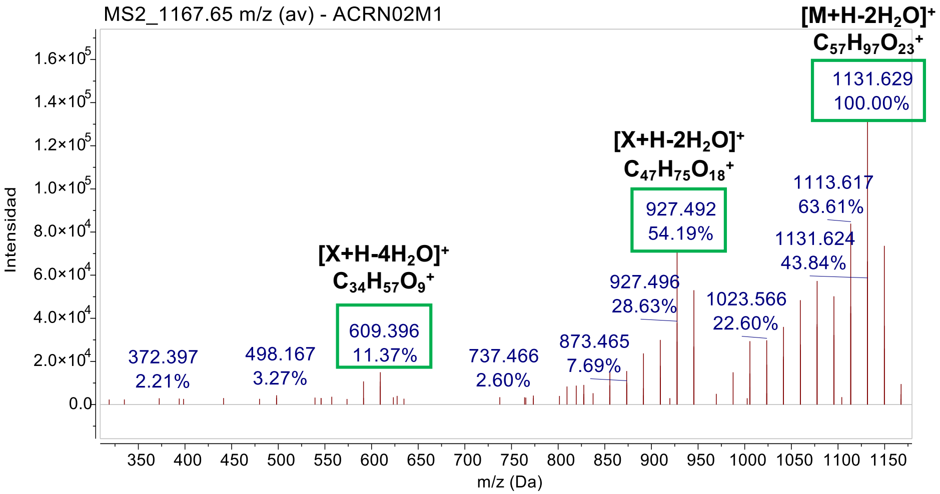
MS2_1167.65 m/z (av) - ACRN02M1
Intensidad
m/z (Da)
[M+H-2H$_2$O]$^+$
$C_{57}H_{97}O_{23}^+$
1131.629
100.00%
[X+H-2H$_2$O]$^+$
$C_{47}H_{75}O_{18}^+$
927.492
54.19%
1113.617
63.61%
1131.624
43.84%
927.496
28.63%
1023.566
22.60%
[X+H-4H$_2$O]$^+$
$C_{34}H_{57}O_9^+$
609.396
11.37%
372.397
2.21%
498.167
3.27%
737.466
2.60%
873.465
7.69%

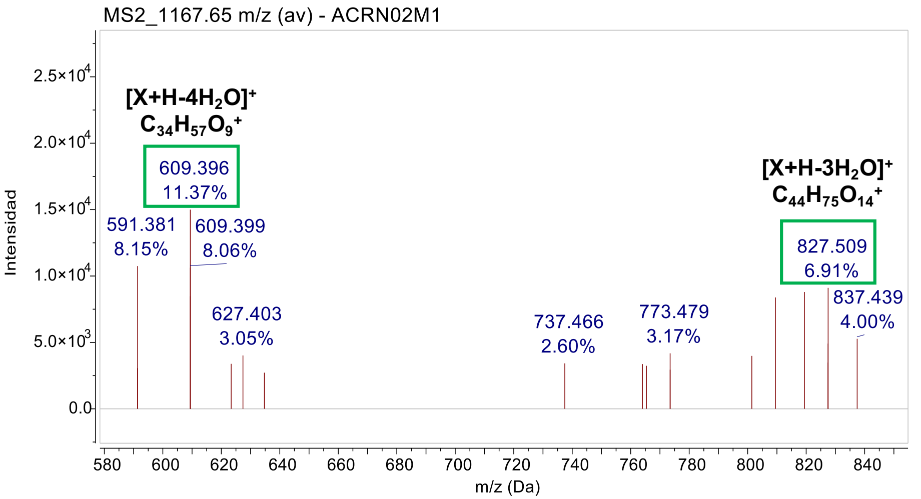
MS2_1167.65 m/z (av) - ACRN02M1
Intensidad
m/z (Da)
[X+H-4H$_2$O]$^+$
$C_{34}H_{57}O_9^+$
609.396
11.37%
591.381
8.15%
609.399
8.06%
627.403
3.05%
737.466
2.60%
773.479
3.17%
[X+H-3H$_2$O]$^+$
$C_{44}H_{75}O_{14}^+$
827.509
6.91%
837.439
4.00%

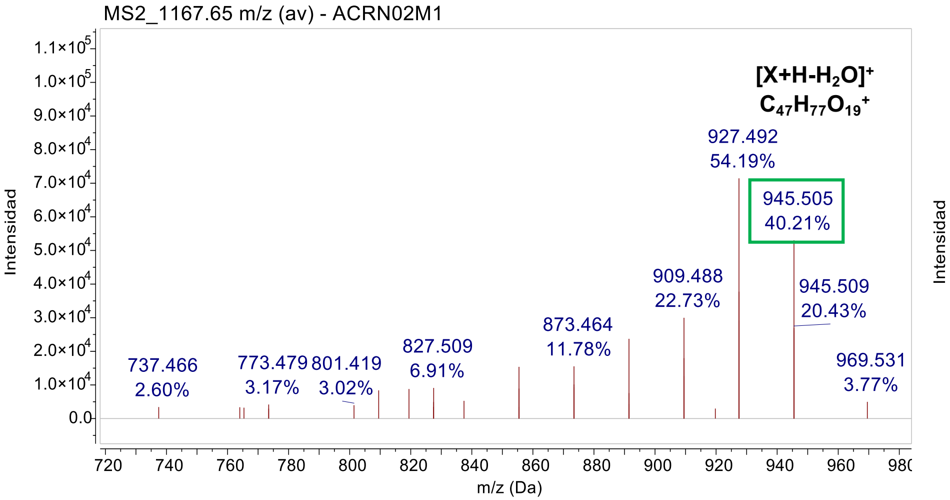
MS2_1167.65 m/z (av) - ACRN02M1
Intensidad
m/z (Da)
[X+H-H$_2$O]$^+$
$C_{47}H_{77}O_{19}^+$
927.492
54.19%
945.505
40.21%
909.488
22.73%
945.509
20.43%
873.464
11.78%
827.509
6.91%
737.466
2.60%
773.479
3.17%
801.419
3.02%
969.531
3.77%

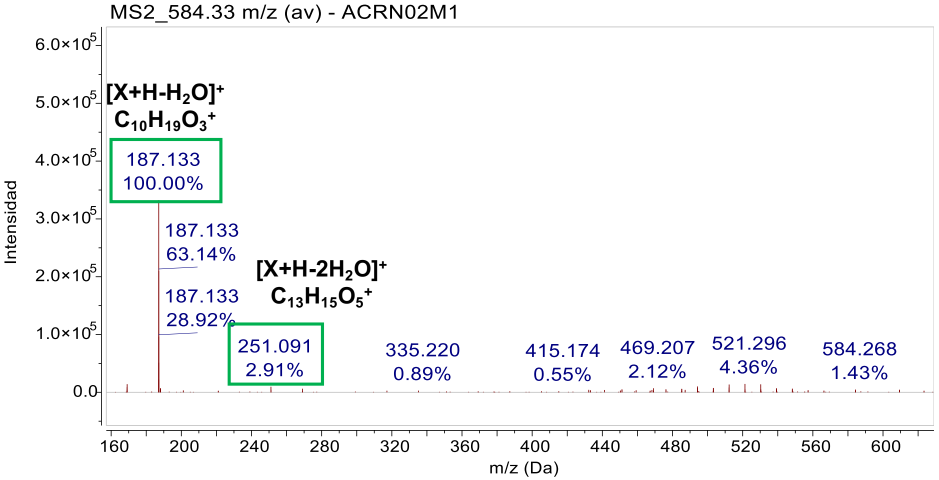
MS2_584.33 m/z (av) - ACRN02M1
Intensidad
m/z (Da)
[X+H-H$_2$O]$^+$
$C_{10}H_{19}O_3^+$
187.133
100.00%
187.133
63.14%
187.133
28.92%
[X+H-2H$_2$O]$^+$
$C_{13}H_{15}O_5^+$
251.091
2.91%
335.220
0.89%
415.174
0.55%
469.207
2.12%
521.296
4.36%
584.268
1.43%

Report S12. Characterization of **amphidinol 30** (*m/z* 1201.67) in **ACRN02**.

Properties

**Exact Mass**	1200.6503 g/mol
**Ion [M+H]+**	*m/z* 1201.6545
**Formula**	$C_{57}H_{100}O_{26}$
**RT**	4.21 min
**C LogP**	–10.5936

Full HRMS Characterization

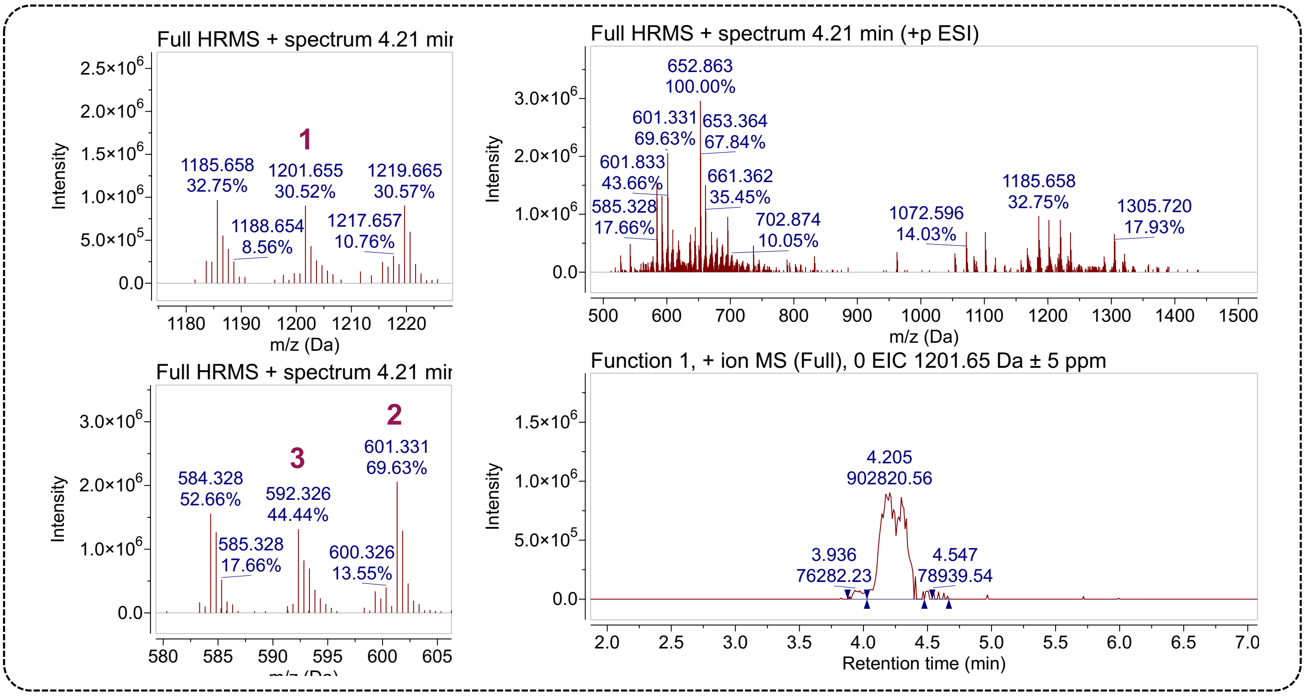

	Ion	Formula	*m/z* theo.	*m/z* exp.	RDB	Δ mDa	Δ ppm
**1**	$[M+H]^+$	$C_{57}H_{101}O_{26}^+$	1201.6576	1201.6554	7.5	–2.16	–1.80
**2**	$[M+2H]^{2+}$	$C_{57}H_{102}O_{26}^{2+}$	601.3324	601.3309	7.0	–3.09	–2.57
**3**	$[M+2H–H_2O]^{2+}$	$C_{57}H_{100}O_{25}^{2+}$	592.3272	592.3256	8.0	–3.03	–2.55

MS² fragments annotation

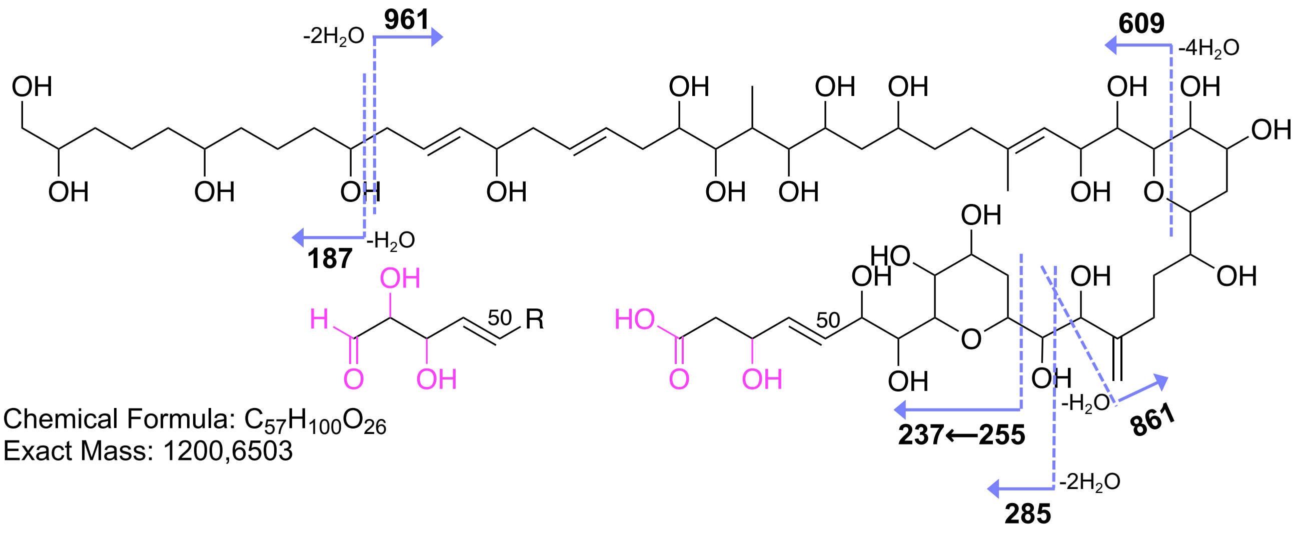

NOTE: The functional groups shown in red are located in the most likely position according to our approach and hypothesis described in the article.

	clv	Ion	Formula	*m/z* theo.	*m/z* exp.	RDB	Δ ppm	Neutral loss (H_2O)
1		$[M+H–2H_2O]^+$	$C_{57}H_{97}O_{24}^+$	1165.6364	1165.6350	9.5	–1.22	10
2	a	$[X+H–2H_2O]^+$	$C_{47}H_{77}O_{20}^+$	961.5003	961.4984	9.5	–2.00	7
3	c	$[X+H–H_2O]^+$	$C_{44}H_{77}O_{16}^+$	861.5206	861.5144	6.5	–7.21	1
4	f	$[X+H–4H_2O]^+$	$C_{34}H_{57}O_9^+$	609.3997	609.3963	6.5	–5.60	2
5	s	$[X+H–2H_2O]^+$	$C_{13}H_{17}O_7^+$	285.0969	285.0968	5.5	–0.17	-
6	w	$[X+H–2H_2O]^+$	$C_{12}H_{15}O_6^+$	255.0863	255.0855	5.5	–3.05	1
7	w	$[X+H–3H_2O]^+$	$C_{12}H_{13}O_5^+$	237.0758	237.0753	6.5	–2.09	-
8	k	$[X+H–H_2O]^+$	$C_{10}H_{19}O_3^+$	187.1329	187.1327	1.5	–0.97	3

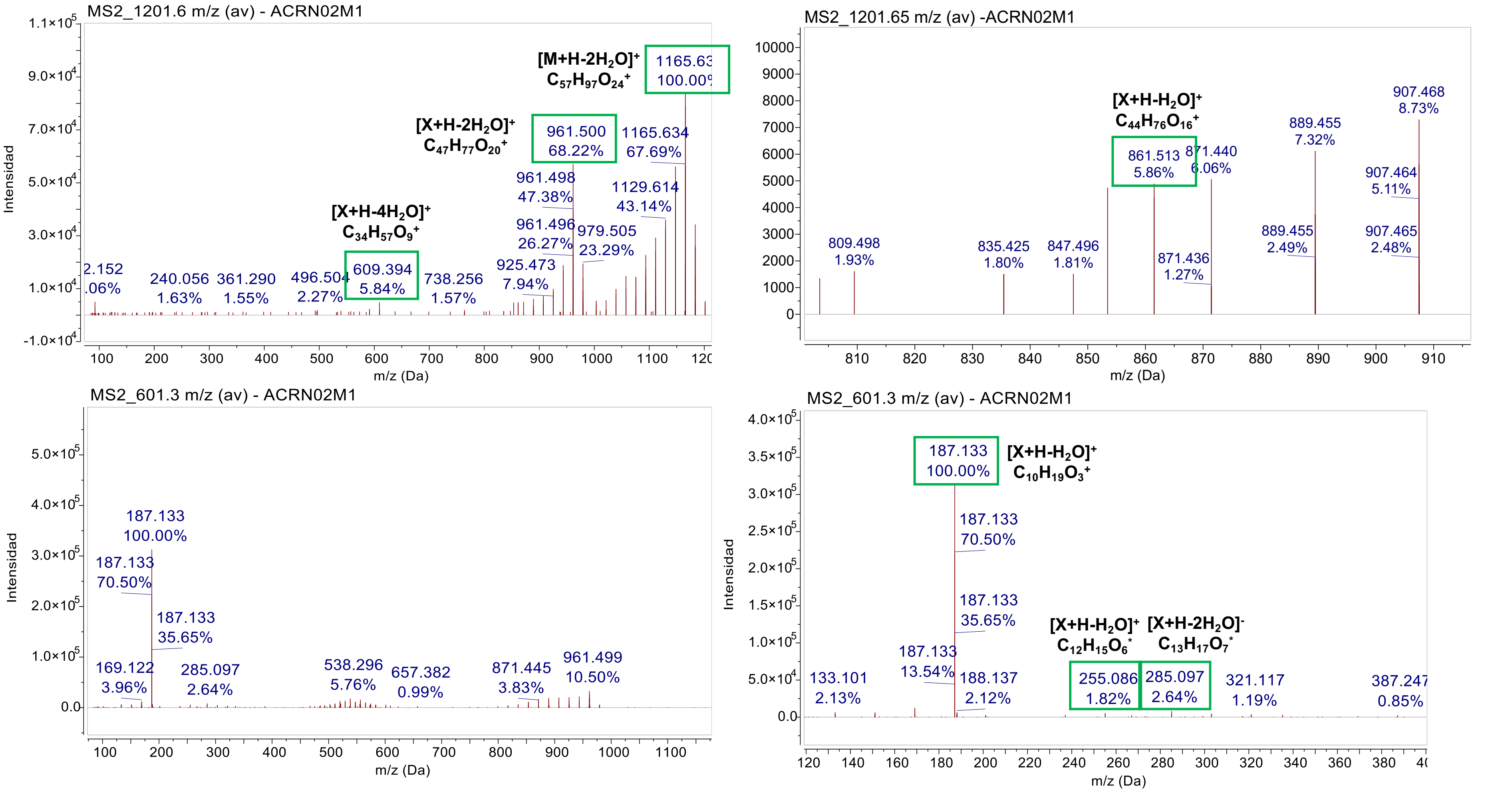
MS2_1201.6 m/z (av) - ACRN02M1
Intensidad
m/z (Da)
[M+H-2H2O]+ C57H97O24+
1165.63
100.00
[X+H-2H2O]+ C47H77O20+
961.500
68.22%
1165.634
67.69%
961.498
47.38%
1129.614
43.14%
961.496
26.27%
979.505
23.29%
925.473
7.94%
[X+H-4H2O]+ C34H57O9+
609.394
5.84%
738.256
1.57%
496.504
2.27%
361.290
1.55%
240.056
1.63%
2.152
.06%
MS2_1201.65 m/z (av) -ACRN02M1
m/z (Da)
[X+H-H2O]+ C44H76O16+
861.513
5.86%
871.440
6.06%
889.455
7.32%
907.468
8.73%
907.464
5.11%
889.455
2.49%
907.465
2.48%
871.436
1.27%
809.498
1.93%
835.425
1.80%
847.496
1.81%
MS2_601.3 m/z (av) - ACRN02M1
Intensidad
m/z (Da)
187.133
100.00%
187.133
70.50%
187.133
35.65%
169.122
3.96%
285.097
2.64%
538.296
5.76%
657.382
0.99%
871.445
3.83%
961.499
10.50%
MS2_601.3 m/z (av) - ACRN02M1
Intensidad
m/z (Da)
187.133
100.00%
[X+H-H2O]+ C10H19O3+
187.133
70.50%
187.133
35.65%
187.133
13.54%
188.137
2.12%
133.101
2.13%
[X+H-H2O]+ C12H15O6+
255.086
1.82%
[X+H-2H2O]- C13H17O7+
285.097
2.64%
321.117
1.19%
387.247
0.85%

Report S13. Characterization of **amphidinol 31** (*m/z* 1219.67) in **ACRN02**.

Properties

**Exact Mass**	1218.6608 g/mol
**Ion [M+H]⁺**	*m/z* 1219.6648
**Formula**	$C_{57}H_{102}O_{27}$
**RT**	4.15 min
**C LogP**	–11.8638

Full HRMS Characterization

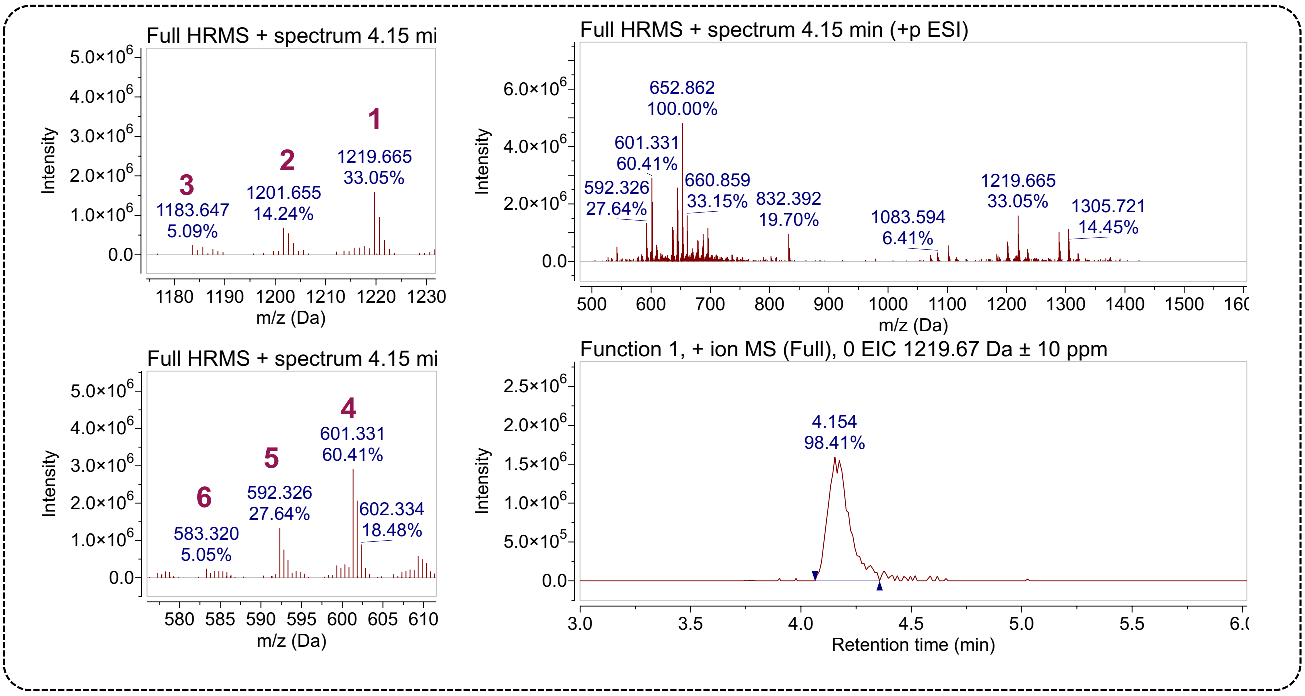

	Ion	Formula	*m/z* theo.	*m/z* exp.	RDB	Δ mDa	Δ ppm
**1**	[M+H]⁺	$C_{57}H_{103}O_{27}^+$	1219.6681	1219.6648	6.5	–3.33	–2.73
**2**	[M+H–H₂O]⁺	$C_{57}H_{101}O_{26}^+$	1201.6576	1201.6547	7.5	–2.90	–2.41
**3**	[M+H–2H₂O]⁺	$C_{57}H_{99}O_{25}^+$	1183.6470	1183.6467	8.5	–0.27	–0.23
**4**	[M+2H–H₂O]²⁺	$C_{57}H_{102}O_{26}^{2+}$	601.3324	601.3308	7.0	–3.34	–2.77
**5**	[M+2H–2H₂O]²⁺	$C_{57}H_{100}O_{25}^{2+}$	592.3271	592.3255	8.0	–3.27	–2.76
**6**	[M+2H–3H₂O]²⁺	$C_{57}H_{98}O_{24}^{2+}$	583.3218	583.3196	9.0	–4.55	–3.90

MS[2] fragments annotation

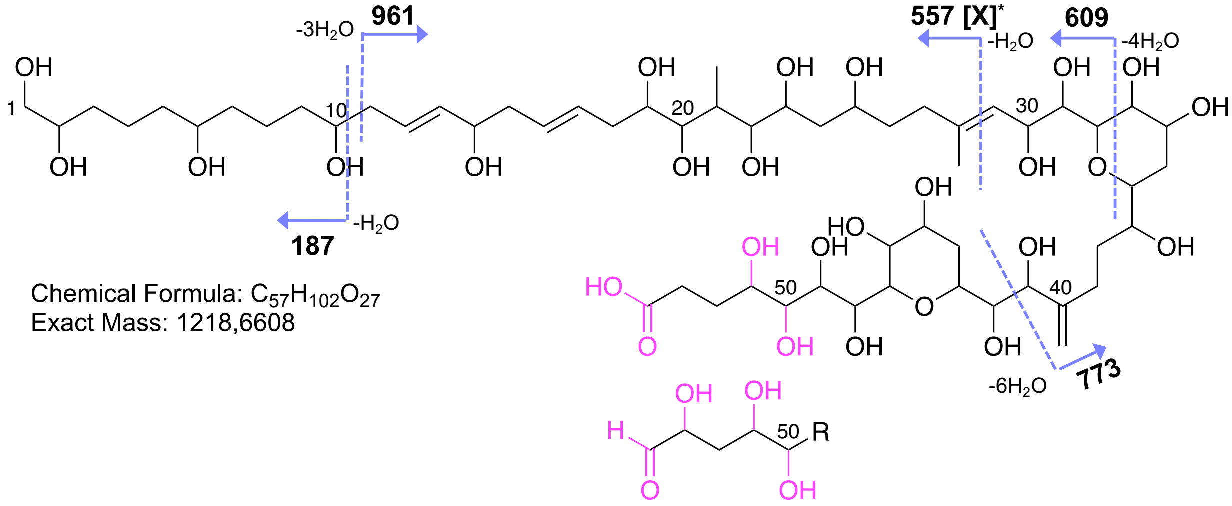

Chemical Formula: $C_{57}H_{102}O_{27}$
Exact Mass: 1218,6608

NOTE: The functional groups shown in red are located in the most likely position according to our approach and hypothesis described in the article.

	clv	Ion/radical	Formula	*m/z* theo.	*m/z* exp.	RDB	Δ ppm	Neutral loss (H_2O)
1		$[M+H–3H_2O]^+$	$C_{57}H_{97}O_{24}^+$	1165.6364	1165.6348	9.5	–1.43	10
2	a	$[X+H–3H_2O]^+$	$C_{47}H_{77}O_{20}^+$	961.5003	961.4974	9.5	–2.95	8
3	c	$[X+H–6H_2O]^+$	$C_{44}H_{69}O_{11}^+$	773.4834	773.4795	10.5	–5.11	2
4	f	$[X+H–4H_2O]^+$	$C_{34}H_{57}O_9^+$	609.3997	609.3999	6.5	0.32	3
5	g	$[X–H_2O]^+$	$C_{30}H_{53}O_9^+$	557.3684	557.3690	4.5	1.09	3
6	k	$[X+H–H_2O]^+$	$C_{10}H_{19}O_3^+$	187.1329	187.1326	1.5	–1.30	-

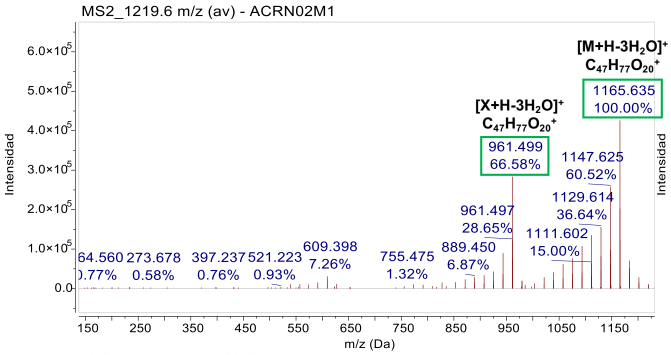

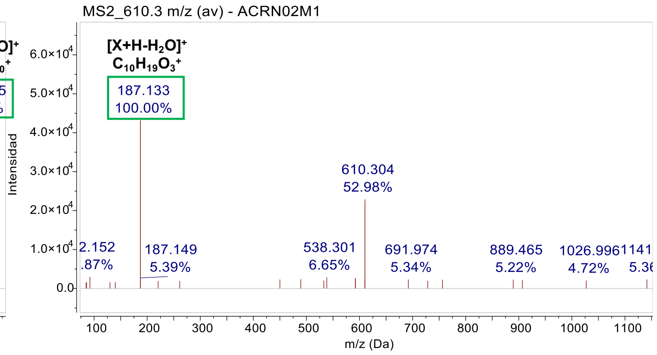

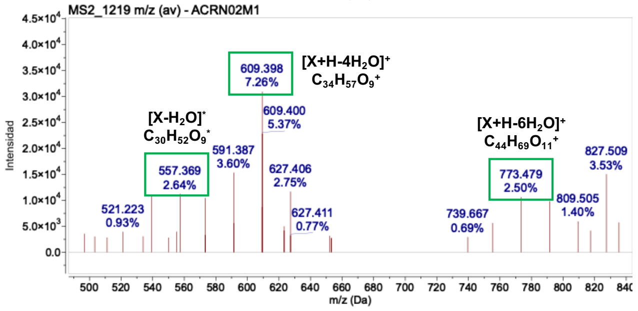

Report S14. Characterization of **amphidinol 32** (*m/z* 1235.66) in **ACRN02**.

Properties

**Exact Mass**	1234.6558 g/mol
**Ion [M+H]⁺**	*m/z* 1235.6648
**Formula**	$C_{57}H_{102}O_{28}$
**RT**	4.21
**C LogP**	–12.5134

Full HRMS Characterization

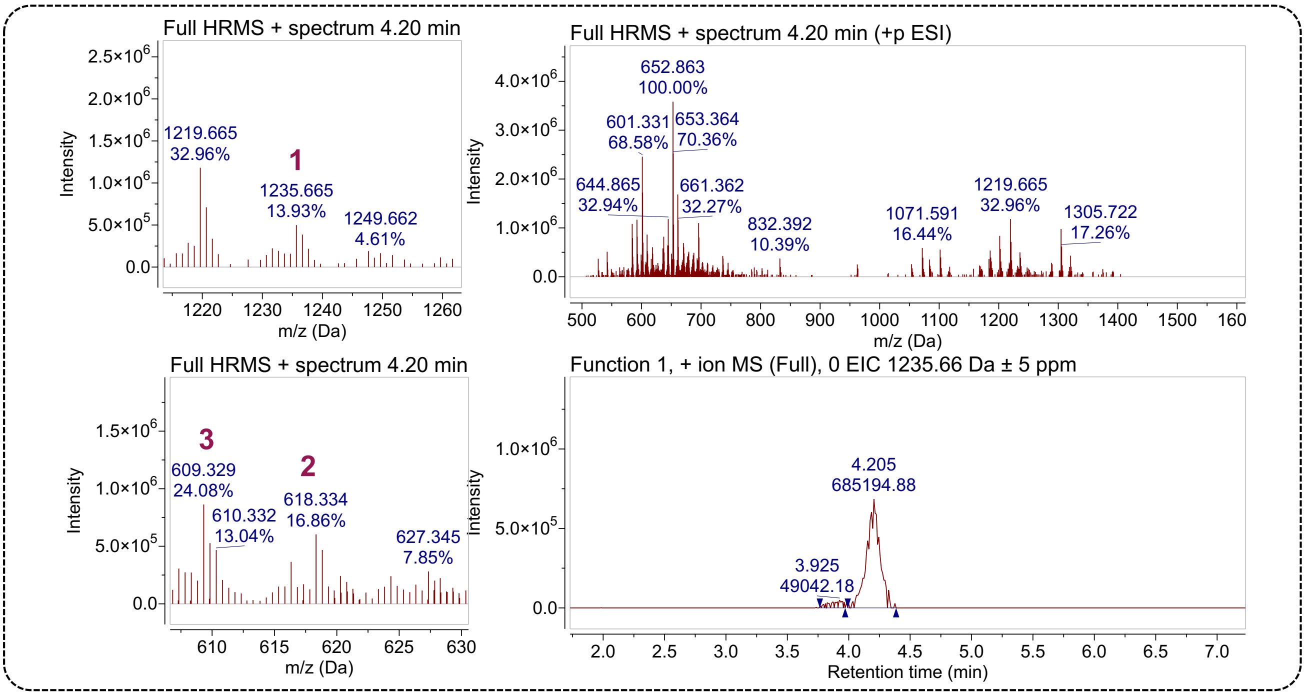

	Ion	Formula	*m/z* theo.	*m/z* exp.	RDB	Δ mDa	Δ ppm
**1**	[M+H]⁺	$C_{57}H_{103}O_{28}^{+}$	1235.6630	1235.6648	6.5	1.76	1.42
**2**	[M+2H]²⁺	$C_{57}H_{104}O_{28}^{2+}$	618.3355	618.3344	6.0	–1.61	–1.31
**3**	[M+2H–H₂O]²⁺	$C_{57}H_{102}O_{27}^{2+}$	609.3299	609.3278	7.0	–4.11	–3.37

MS² fragments annotation

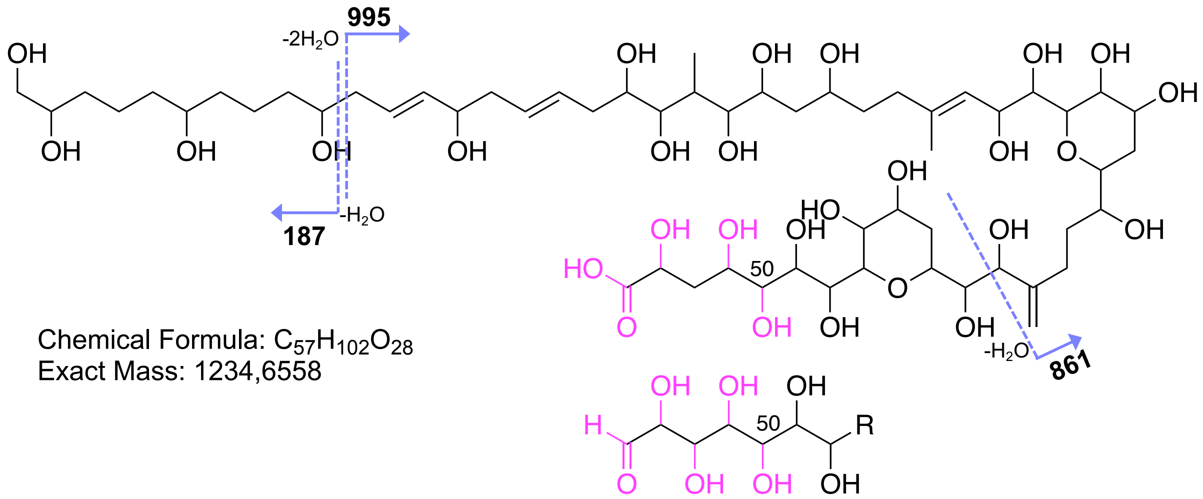

NOTE: The functional groups shown in red are located in the most likely position according to our approach and hypothesis described in the article.

	clv	Ion	Formula	*m/z* theo.	*m/z* exp.	RDB	Δ ppm	Neutral loss (H_2O)
1		$[M+H–2H_2O]^+$	$C_{57}H_{99}O_{26}^+$	1199.6419	1199.6418	8.5	–0.05	9
2	a	$[X+H–2H_2O]^+$	$C_{47}H_{81}O_{23}^+$	995.5057	995.5057	8.5	–0.75	4
3	c	$[X+H–H_2O]^+$	$C_{44}H_{77}O_{16}^+$	861.5206	861.5190	6.5	–1.82	1
4	s	$[X+H–3H_2O]^+$	$C_{13}H_{19}O_8^+$	303.1074	303.1065	4.5	–3.10	-
5	k	$[X+H–H_2O]^+$	$C_{10}H_{19}O_3^+$	187.1329	187.1327	1.5	–0.89	-

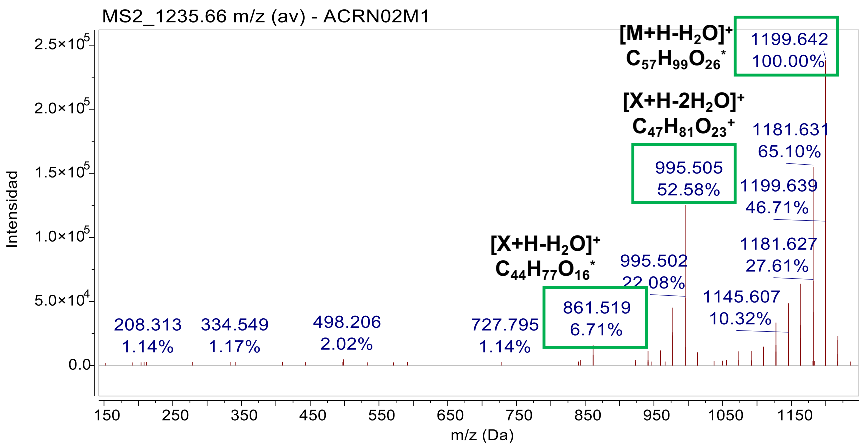

Report S15. Characterization of **amphidinol 33** (*m/z* 1101.60) in **ACRN02**.

Properties

**Exact Mass**	1100.5979 g/mol
**Ion [M+H]+**	*m/z* 1101.6027
**Formula**	$C_{57}H_{92}O_{24}$
**RT**	4.17 min
**C LogP**	–10.0295

Full HRMS Characterization

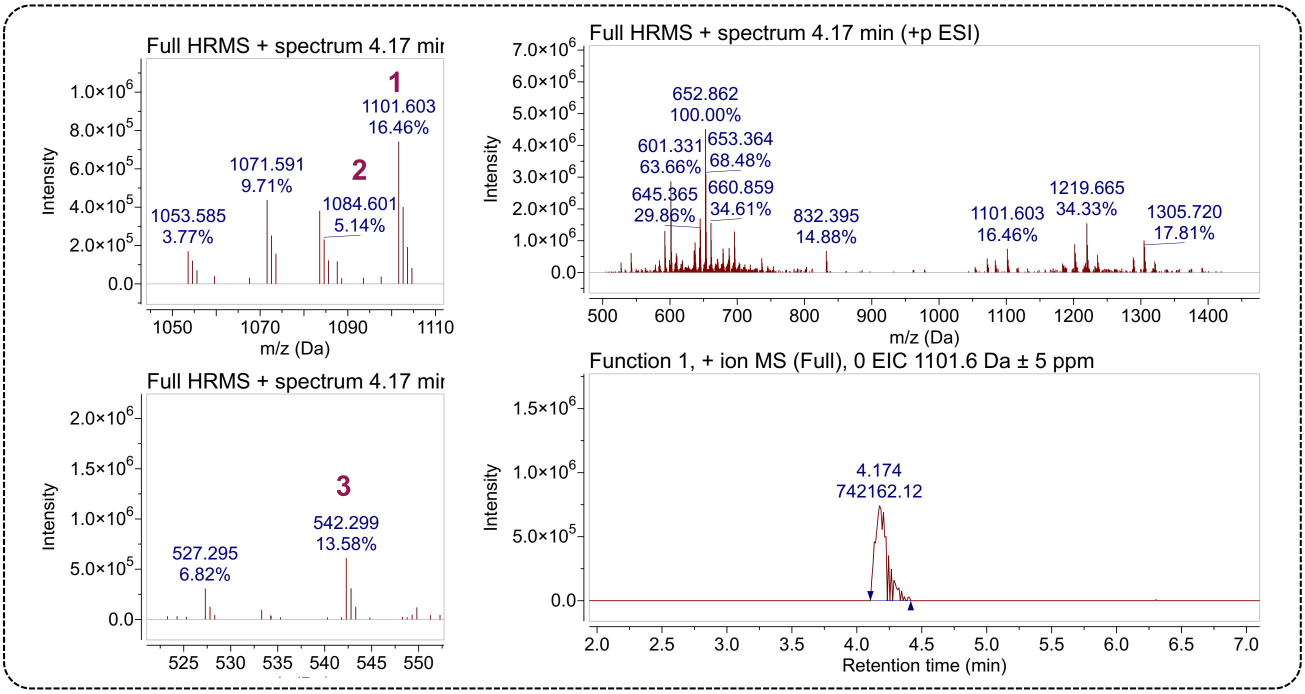

	Ion	Formula	*m/z* theo.	*m/z* exp.	RDB	Δ mDa	Δ ppm
**1**	$[M+H]^+$	$C_{52}H_{93}O_{24}^+$	1101.6051	1101.6027	6.5	–2.47	–2.24
**2**	$[M+H–H_2O]^+$	$C_{52}H_{91}O_{23}^+$	1083.5946	1083.5908	7.5	–3.75	–3.46
**3**	$[M+2H–H_2O]^{2+}$	$C_{52}H_{93}O_{23}^{2+}$	542.3009	542.2993	7.0	–3.21	–2.96

MS^2 fragments annotation

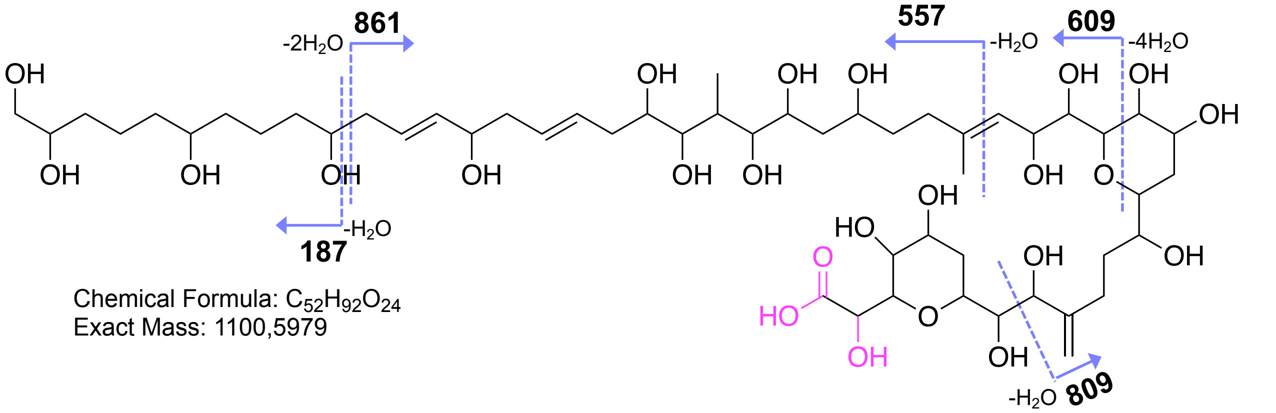

Chemical Formula: $C_{52}H_{92}O_{24}$
Exact Mass: 1100,5979

NOTE: The functional groups shown in red are located in the most likely position according to our approach and hypothesis described in the article.

	clv	Ion	Formula	*m/z* theo.	*m/z* exp.	RDB	Δ ppm	Neutral loss (H_2O)
1		$[M+H–2H_2O]^+$	$C_{52}H_{89}O_{22}^+$	1065.5840	1065.5823	8.5	–1.62	8
2	a	$[X+H–2H_2O]^+$	$C_{42}H_{69}O_{18}^+$	861.4478	861.4466	8.5	–1.45	7
3	c	$[X+H–4H_2O]^+$	$C_{44}H_{73}O_{13}^+$	809.5046	809.5004	8.5	–5.20	1
4	f	$[X+H–4H_2O]^+$	$C_{34}H_{57}O_9^+$	609.3997	609.3993	6.5	–0.69	-
5	g	$[X+H–H_2O]^+$	$C_{30}H_{53}O_9^+$	557.3684	557.3690	4.5	0.98	1
6	k	$[X+H–H_2O]^+$	$C_{10}H_{19}O_3^+$	187.1329	187.1327	1.5	–1.05	3

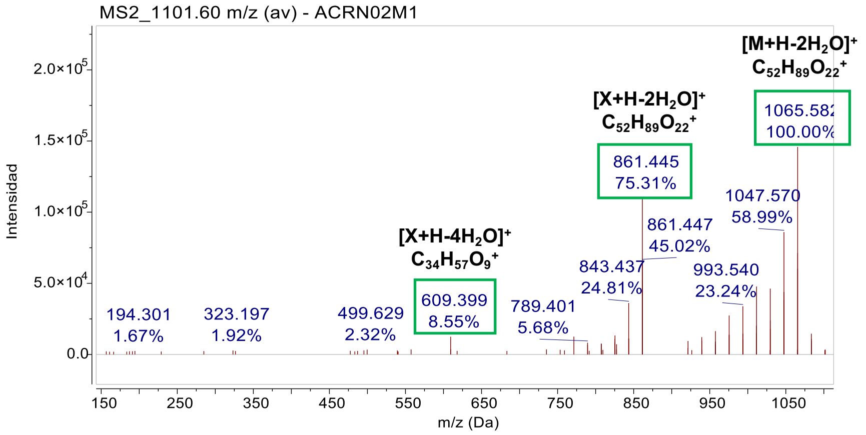

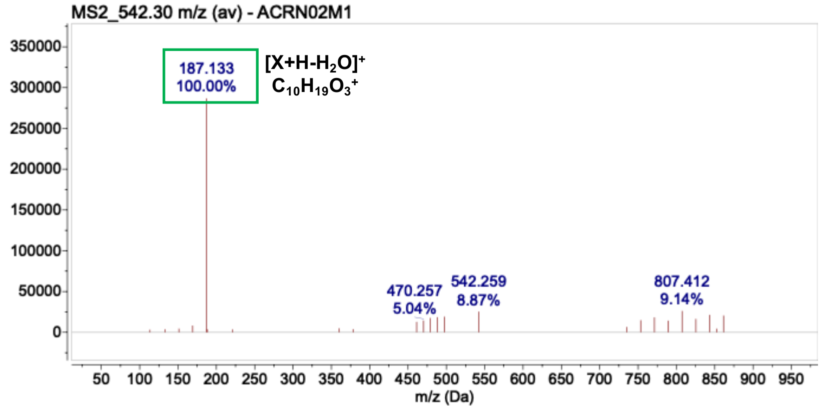

Report S16. Characterization of **amphidinol 34** (*m/z* 1071.59) in **ACRN02.**

Properties

**Exact Mass**	1070.5873 g/mol
**Ion [M+H]+**	*m/z* 1071.5917
**Formula**	$C_{51}H_{90}O_{23}$
**RT**	4.23 min
**C LogP**	–9.2880

Full HRMS Characterization

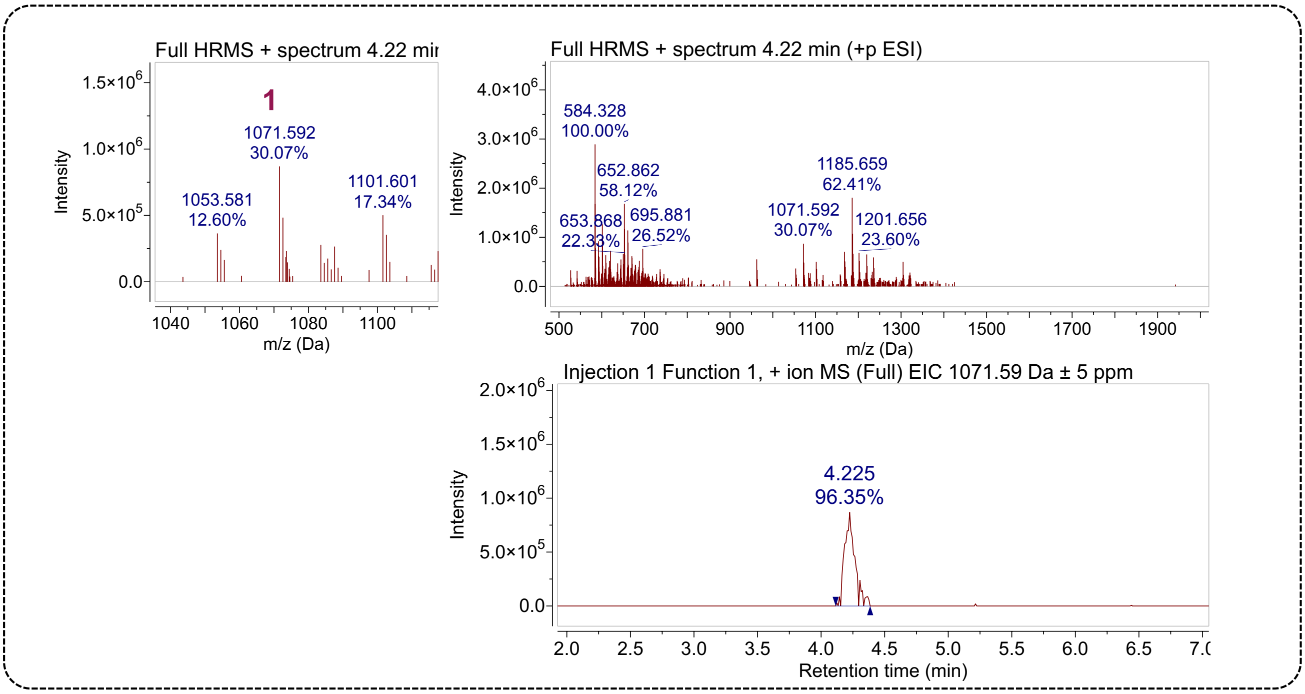

	Ion	Formula	*m/z* theo.	*m/z* exp.	RDB	Δ mDa	Δ ppm
**1**	[M+H]+	$C_{51}H_{91}O_{23}^+$	1071.5946	1071.5917	6.5	–2.89	–2.70

MS^2 fragments annotation

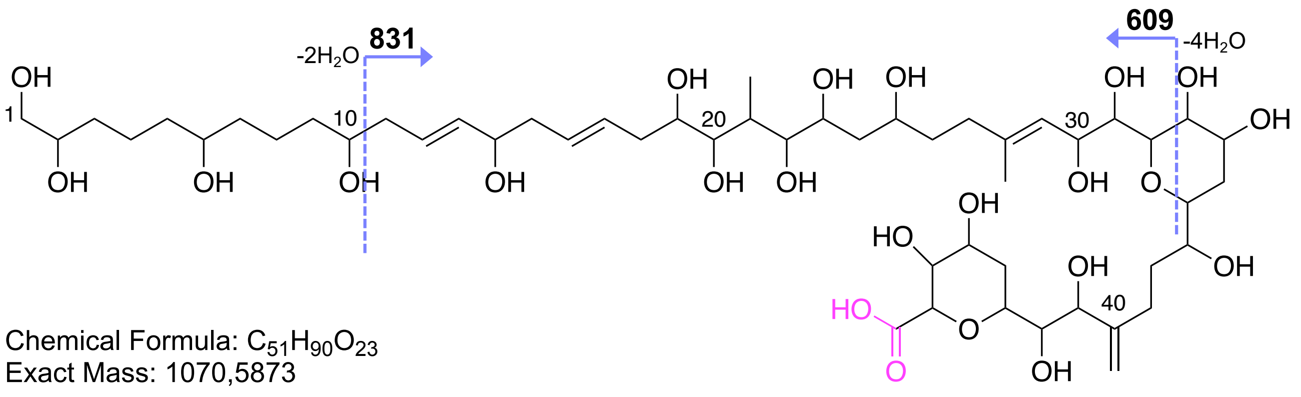

Chemical Formula: $C_{51}H_{90}O_{23}$
Exact Mass: 1070,5873

NOTE: The functional groups shown in red are located in the most likely position according to our approach and hypothesis described in the article.

	clv	Ion	Formula	*m/z* theo.	*m/z* exp.	RDB	Δ ppm	Neutral loss (H_2O)
1	a	$[X+H–2H_2O]^+$	$C_{41}H_{67}O_{17}^+$	831.4373	831.4365	8.5	–0.98	6
2	f	$[X+H–4H_2O]^+$	$C_{34}H_{57}O_9^+$	609.3997	609.3968	6.5	–4.70	-

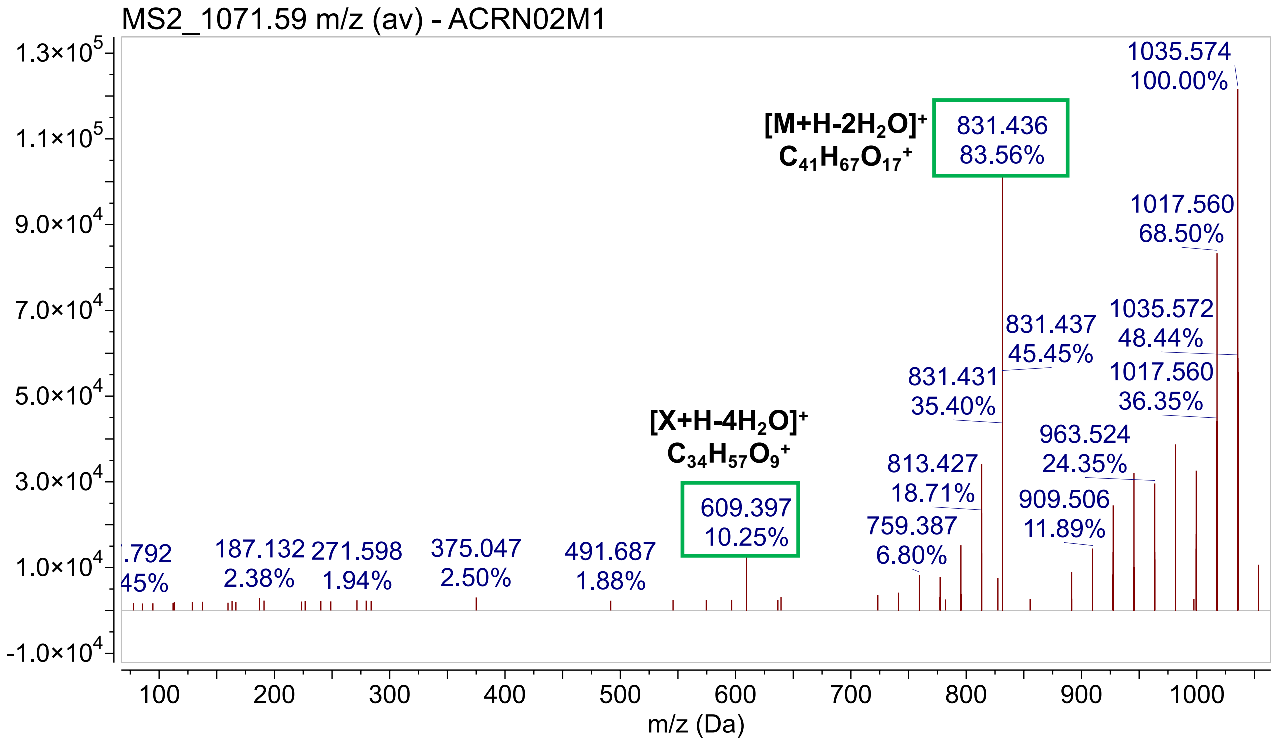

Report S17. Characterization of ion *m/z* 1285.67 in **ACRN02.**

Full HRMS

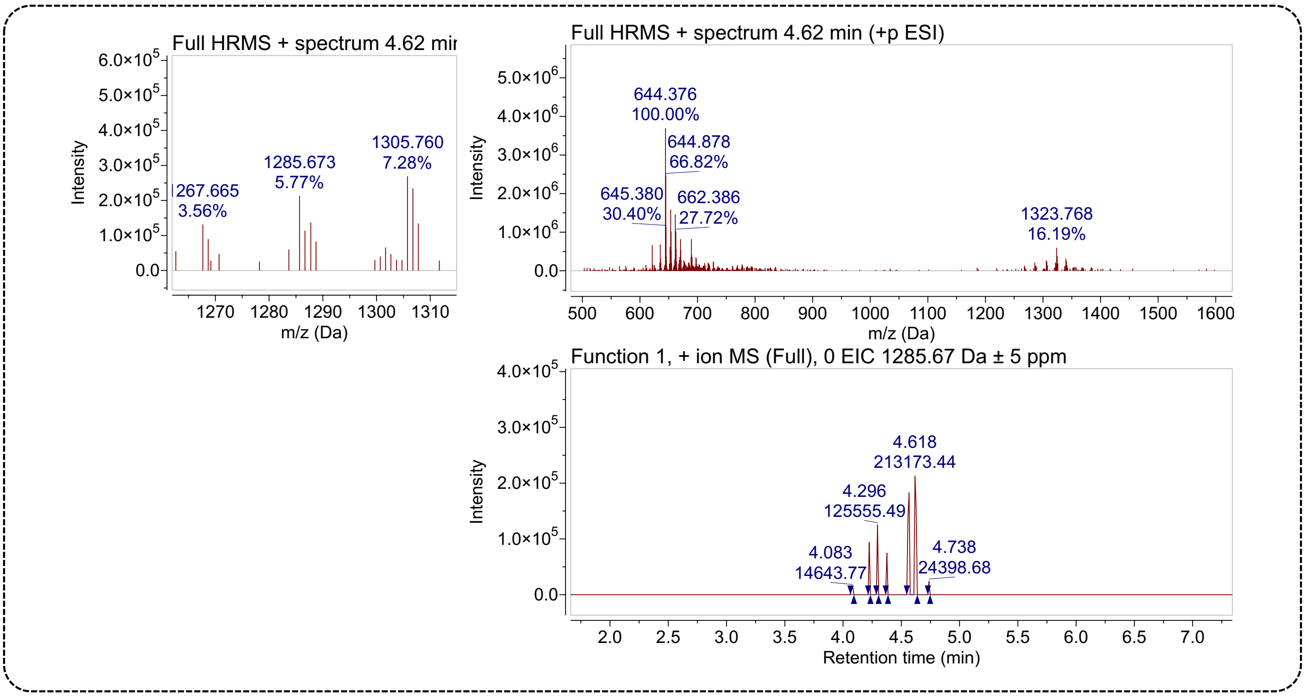

MS^2 – comparison with Luteophanol D

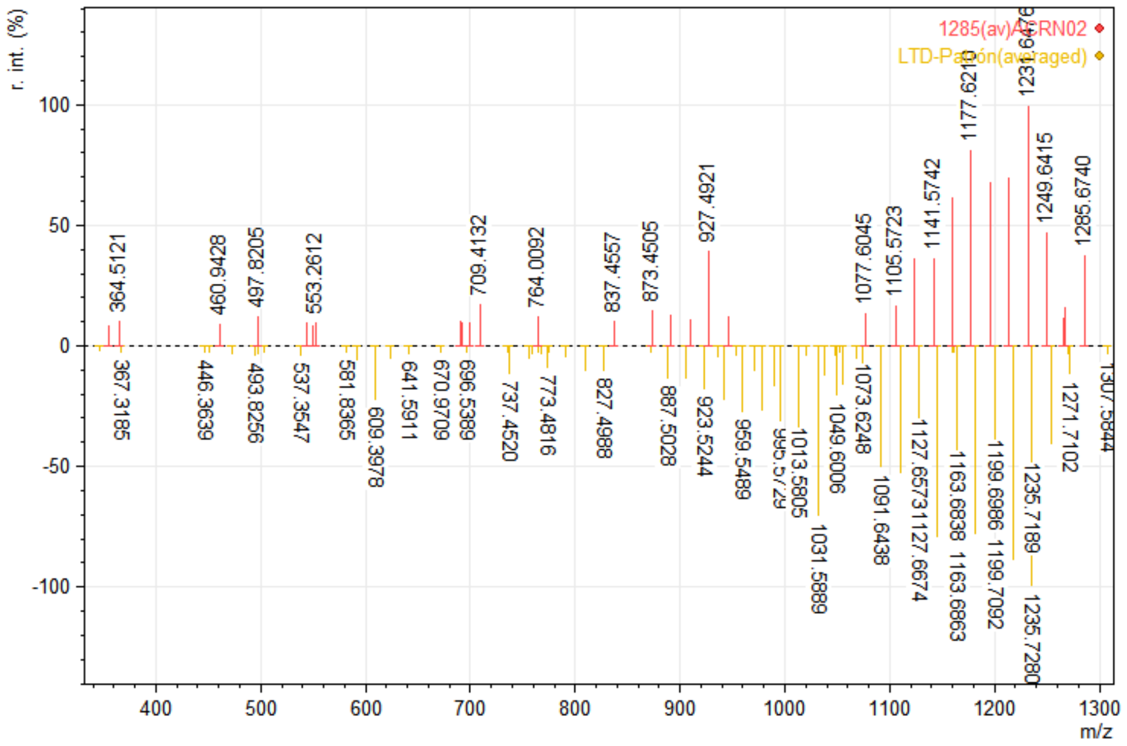

Figure S27. Family of AMs in cell-free medium extract of **ACRN03.**
Colored by retention time.

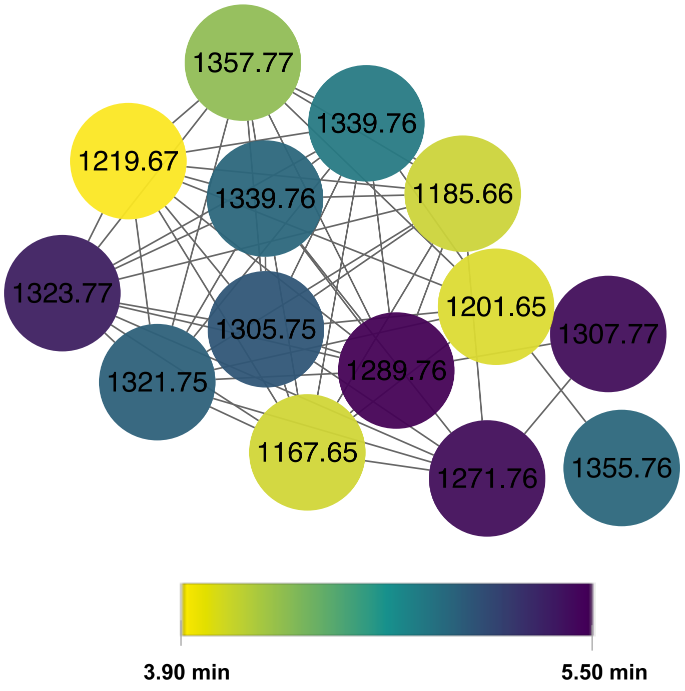

Table S10. Dereplication of potential amphidinols in ACRN03.

Precursor Mass	*m/z*	[M+H]+ 1.01 Da	[M+Na]+ 22.99 Da	[M+K]+ 39.10 Da	[M+ACN]+ 41.05 Da	1st Candidate
1357.77	+1	1356.76	1334.78	1318.67	1316.72	
1355.76	+1	1354.75	1332.77	1316.66	1314.71	
1339.76	+1	1338.75	1316.77	**1300.66**	1298.71	**Amphidinol 4**
1339.76	+1	1338.75	1316.77	**1300.66**	1298.71	**Amphidinol 4**
1323.77	+1	**1322.76**	**1300.78**	1284.67	**1282.72**	**Amphidinol 20B/4**
1321.75*	+1	**1320.74**	1298.76	**1282.65**	1280.70	**Luteophanol-B/C**
1307.77	+1	**1306.76**	1284.78	1268.67	1266.72	**Luteophanol D**
1305.75*	+1	1304.74	**1282.76**	1266.65	1264.70	**Amphidinol 17**
1289.76*	+1	1288.75	1266.77	1250.66	1248.71	
1271.76*	+1	1270.75	1248.77	1232.66	1230.71	
1219.67	+1	1218.66	1196.68	1180.57	1178.62	
1201.65	+1	**1200.64**	1178.66	**1162.55**	1160.60	**Colopsinol-C**
1185.66	+1	1184.65	**1162.67**	1146.56	1144.61	**Amphidinol 15**
1167.65*	+1	1166.64	1144.66	1128.55	1126.66	

(*] Dehydrated protonated ions from other protonated ions

Report S18. Identification of **luteophanol D** (*m/z* 1307.77) in **ACRN03**.

RT 4.89 min

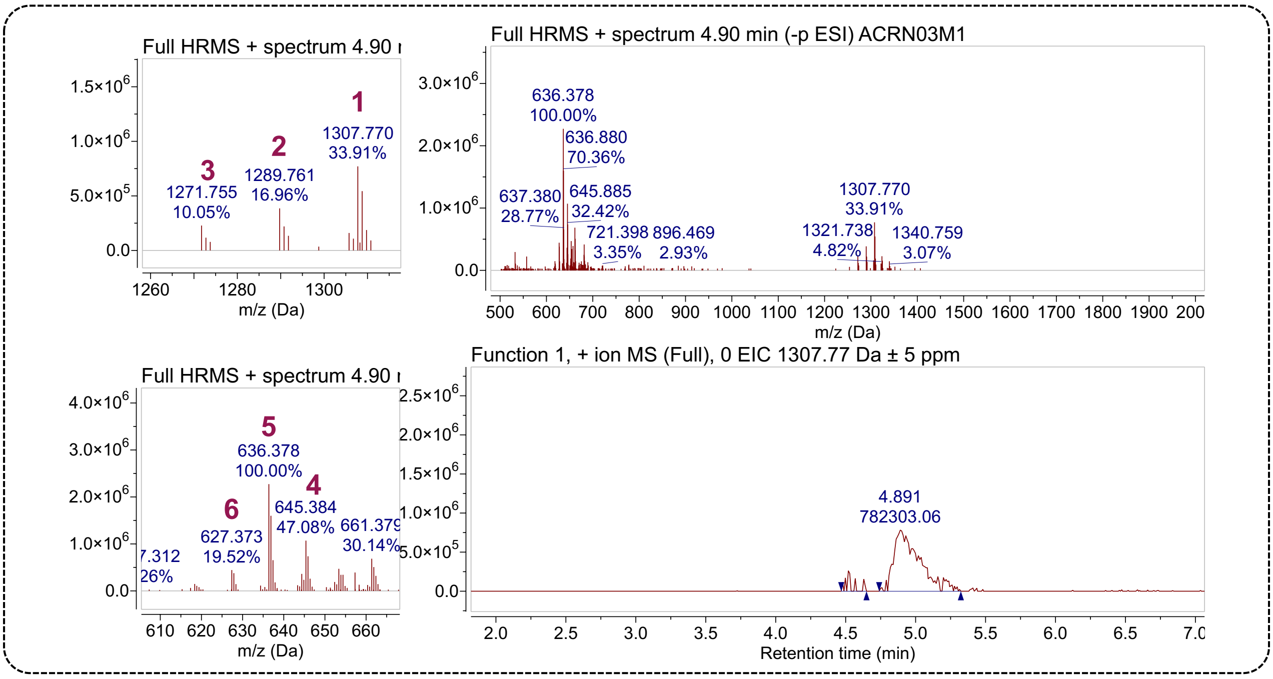

	Ion	Formula	*m/z* theo.	*m/z* exp.	RDB	Δ mDa	Δ ppm
1	$[M+H]^+$	$C_{66}H_{115}O_{25}^+$	1307.7722	1307.7703	9.5	–1.93	–1.48
2	$[M+H–H_2O]^+$	$C_{66}H_{113}O_{24}^+$	1289.7616	1289.7606	10.5	–1.01	–0.78
3	$[M+H–2H_2O]^+$	$C_{66}H_{111}O_{23}^+$	1271.7511	1271.7551	11.5	4.06	3.20
4	$[M+2H–H_2O]^{2+}$	$C_{66}H_{114}O_{24}^{2+}$	645.3845	645.3835	10.0	–1.82	–1.41
5	$[M+2H–2H_2O]^{2+}$	$C_{66}H_{112}O_{23}^{2+}$	636.3792	636.3785	11.0	–1.38	–1.09
6	$[M+2H–3H_2O]^{2+}$	$C_{66}H_{110}O_{22}^{2+}$	627.3739	627.3728	12.0	–2.17	–1.73

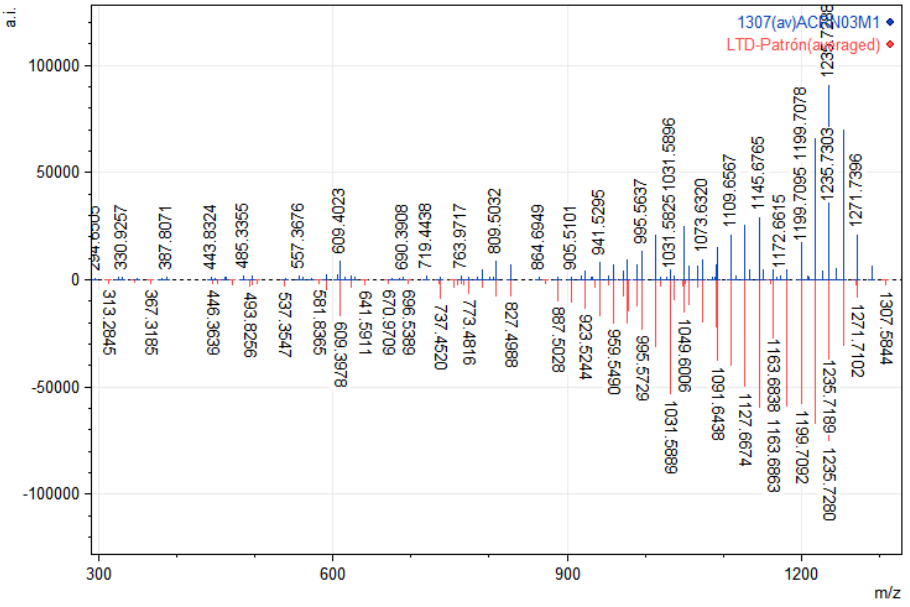

Report S19. Identification of **amphidinol 28** (*m/z* 1323.77) in **ACRN03**.

RT 4.52 min

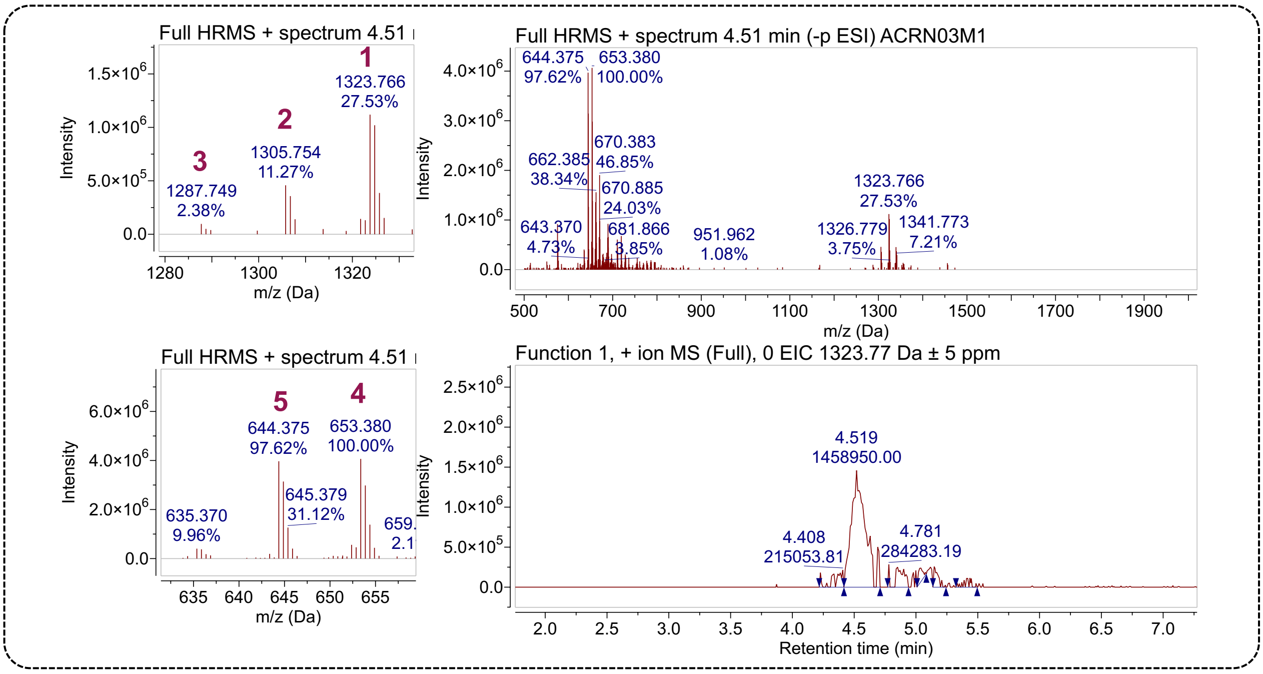

	Ion	Formula	*m/z* theo.	*m/z* exp.	RDB	Δ mDa	Δ ppm
1	$[M+H]^+$	$C_{66}H_{115}O_{26}^+$	1323.7671	1323.7655	9.5	–1.49	–1.12
2	$[M+H–H_2O]^+$	$C_{66}H_{113}O_{25}^+$	1305.7566	1305.7549	10.5	–1.66	–1.27
3	$[M+H–2H_2O]^+$	$C_{66}H_{111}O_{24}^+$	1287.7519	1287.7461	11.5	0.11	0.09
4	$[M+2H–H_2O]^{2+}$	$C_{66}H_{114}O_{25}^{2+}$	653.3819	653.3801	10.0	–3.69	–2.82
5	$[M+2H–2H_2O]^{2+}$	$C_{66}H_{112}O_{24}^{2+}$	644.3767	644.3753	11.0	–2.65	–2.05

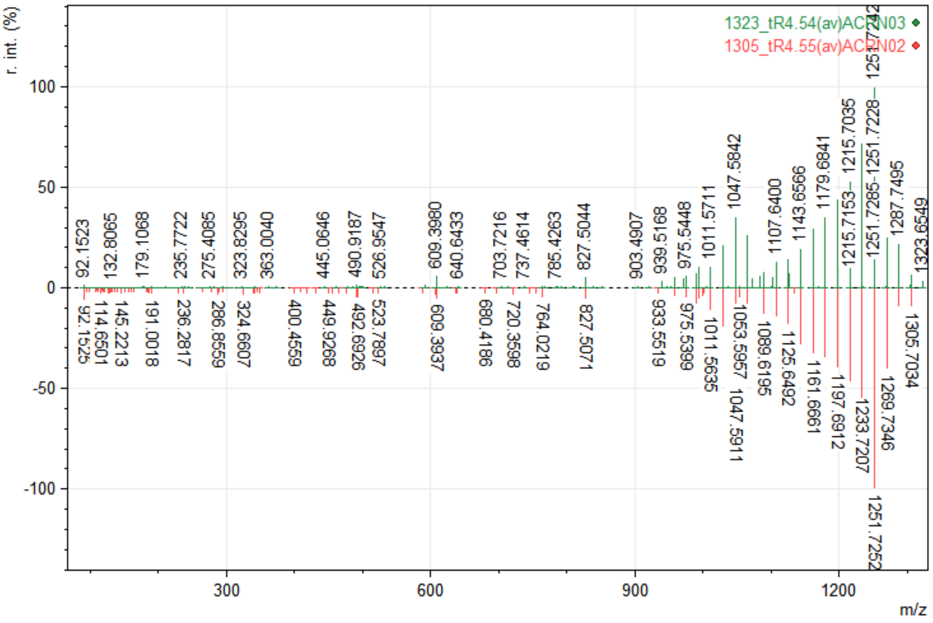

Report S20. Identification of **amphidinol 29** (*m/z* 1339.76) in **ACRN03**.

RT 4.69 min

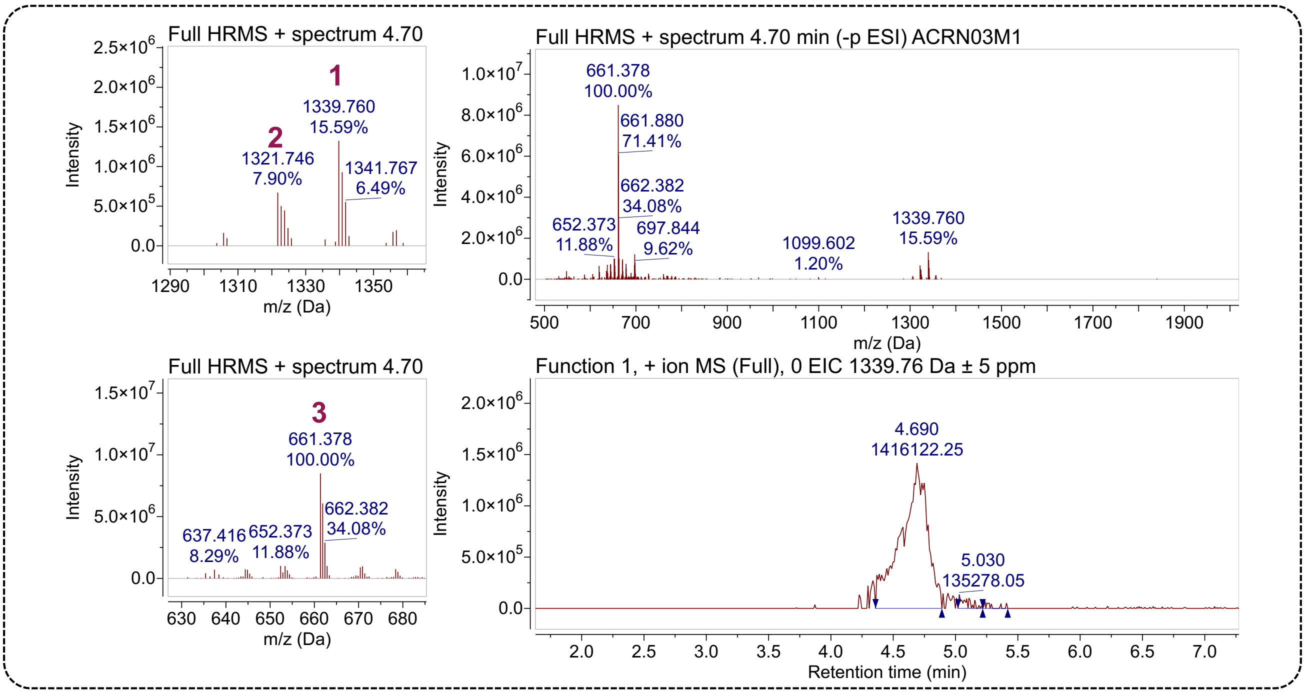

	Ion	Formula	*m/z* theo.	*m/z* exp.	RDB	Δ mDa	Δ ppm
**1**	$[M+H]^+$	$C_{66}H_{115}O_{27}^+$	1339.7620	1339.7600	9.5	–2.01	–1.51
**2**	$[M+H–H_2O]^+$	$C_{66}H_{113}O_{26}^+$	1321.7515	1321.7465	10.5	–5.00	–3.79
**3**	$[M+2H–H_2O]^{2+}$	$C_{66}H_{114}O_{26}^{2+}$	661.3794	661.3782	10.0	–2.27	–1.71

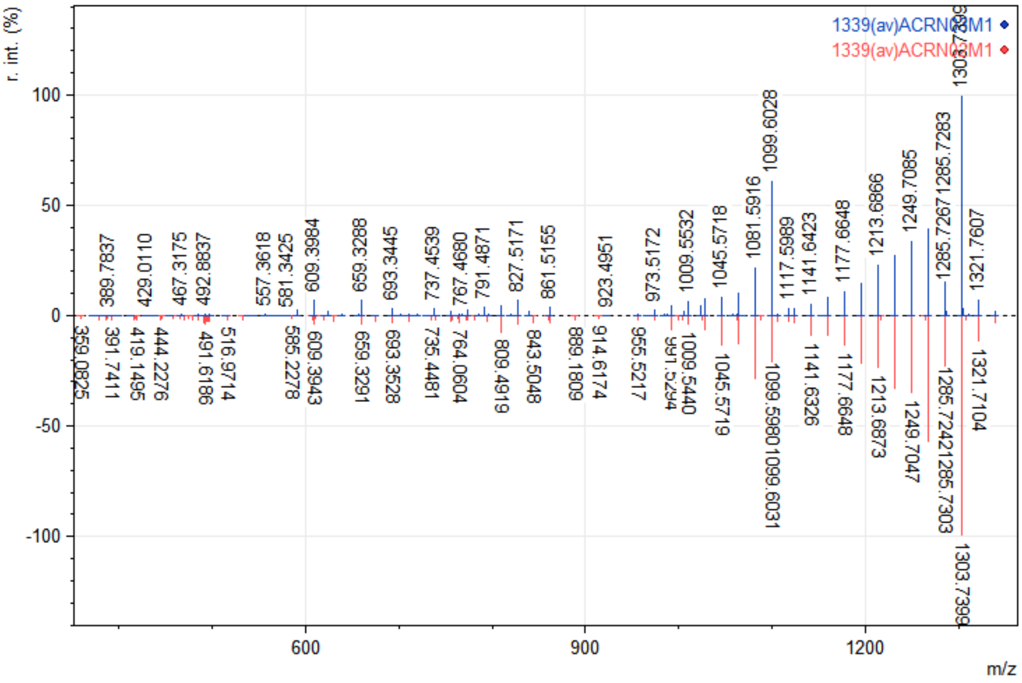

Report S21. Identification of **amphidinol 27** (*m/z* 1185.66) in **ACRN03**.

**RT**	4.26 min

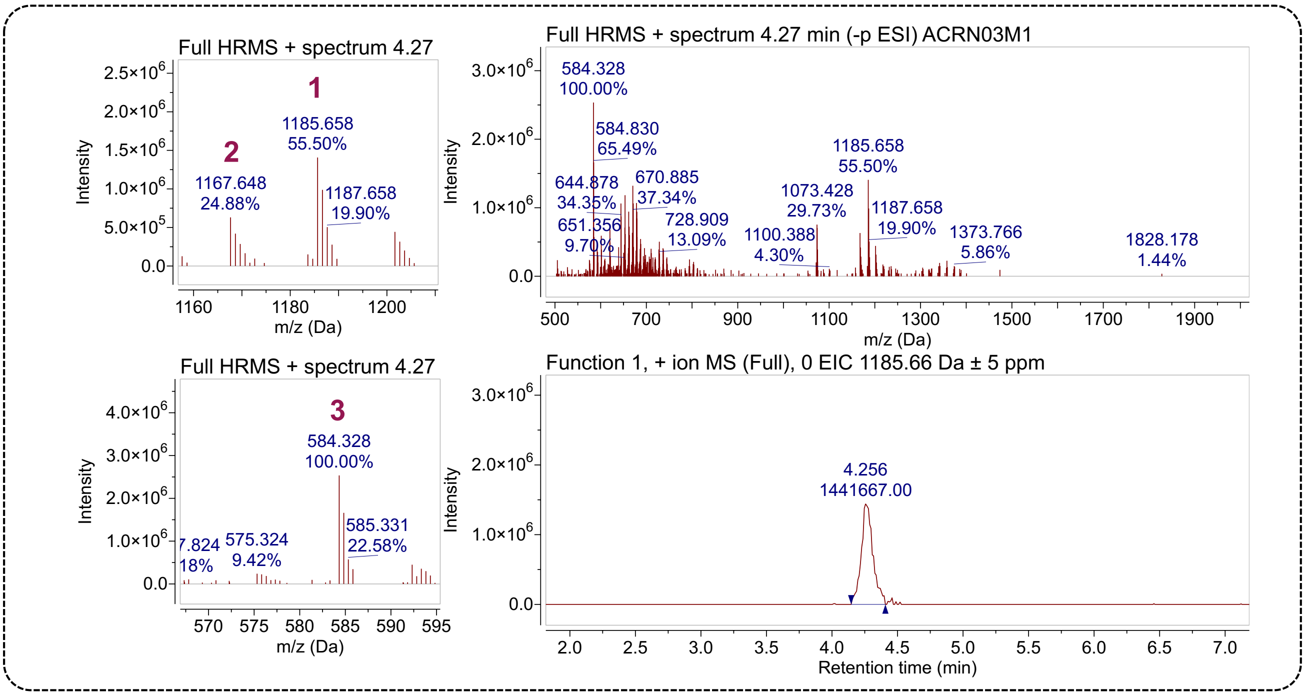

	Ion	Formula	*m/z* theo.	*m/z* exp.	RDB	Δ mDa	Δ ppm
**1**	$[M+H]^+$	$C_{57}H_{101}O_{25}^+$	1185.6626	1185.6580	7.5	–4.69	–3.87
**2**	$[M+H–H_2O]^+$	$C_{57}H_{99}O_{24}^+$	1167.6521	1167.6476	8.5	–4.50	–3.86
**3**	$[M+2H–H_2O]^{2+}$	$C_{57}H_{100}O_{24}^{2+}$	584.3297	584.3280	8.0	–3.35	–2.87

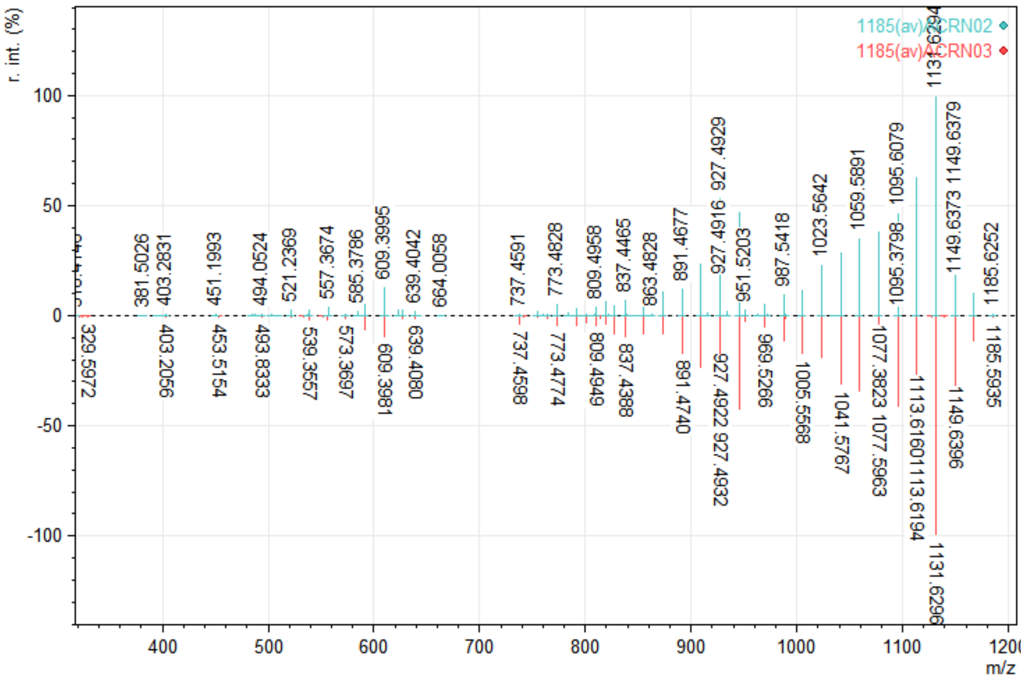

Report S22. Identification of **amphidinol 30** (*m/z* 1201.65) in **ACRN03**.

**RT**	4.23 min

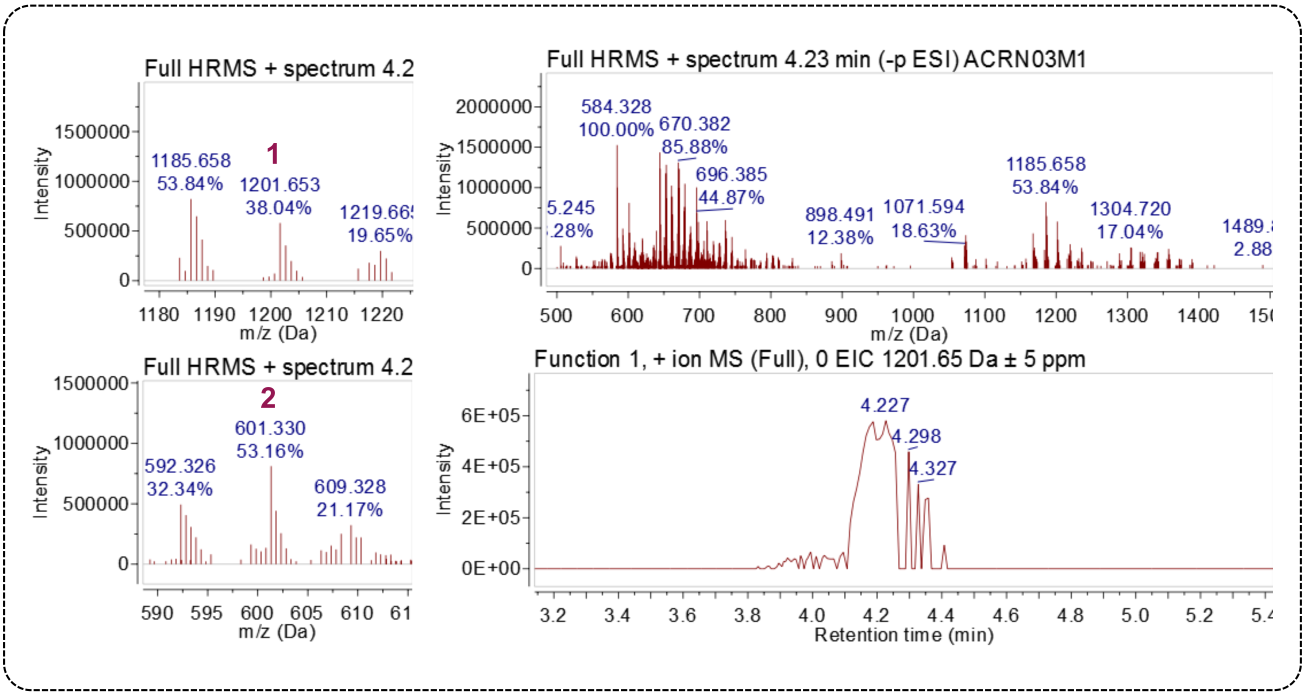

	Ion	Formula	*m/z* theo.	*m/z* exp.	RDB	Δ mDa	Δ ppm
1	$[M+H]^+$	$C_{57}H_{101}O_{26}^+$	1201.6576	1201.6528	7.5	–4.73	–3.94
2	$[M+2H]^{2+}$	$C_{57}H_{102}O_{26}^{2+}$	601.3332	601.3306	9.0	–2.10	–1.75

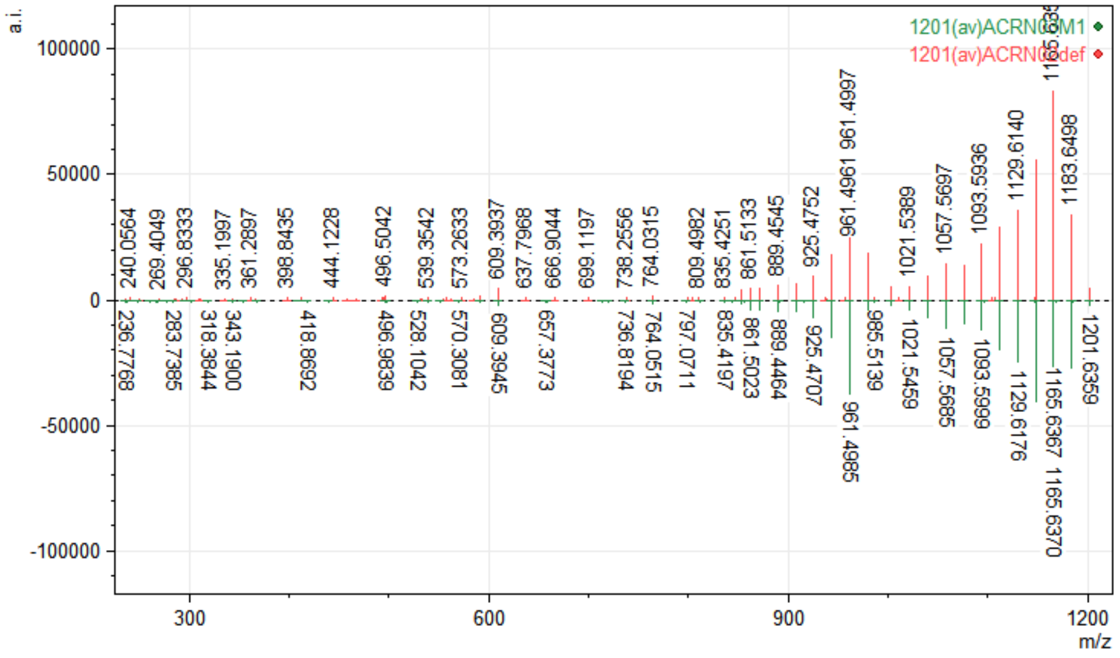

Report S23. Identification of **amphidinol 31** (*m/z* 1219.66) in **ACRN03**.

**RT** 4.17 min

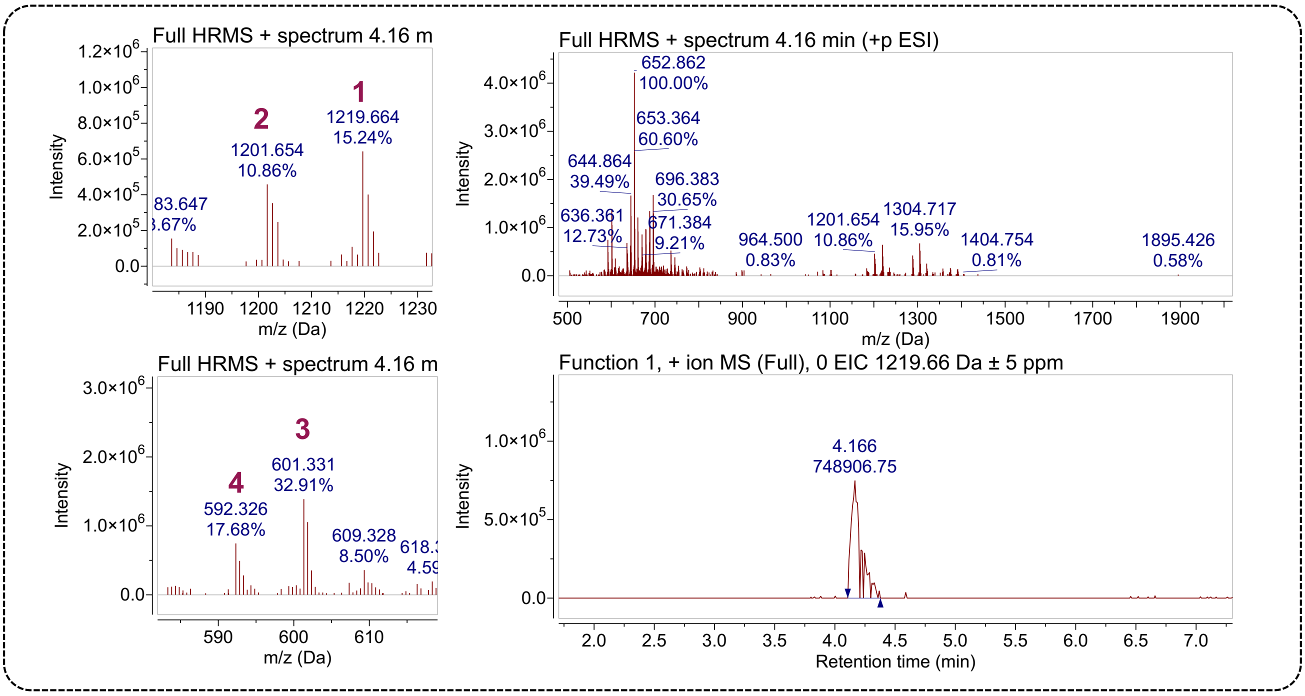

	Ion	Formula	*m/z* theo.	*m/z* exp.	RDB	Δ mDa	Δ ppm
**1**	$[M+H]^+$	$C_{57}H_{103}O_{27}^+$	1219.6681	1219.6644	6.5	–3.70	–3.03
**2**	$[M+H–H_2O]^+$	$C_{57}H_{101}O_{26}^+$	1201.6576	1201.6537	7.5	–3.87	–3.23
**3**	$[M+2H–H_2O]^{2+}$	$C_{57}H_{102}O_{26}^{2+}$	601.3332	601.3306	7.0	–3.70	–3.08
**4**	$[M+2H–2H_2O]^{2+}$	$C_{57}H_{100}O_{25}^{2+}$	592.3272	592.3258	8.0	–2.66	–2.25

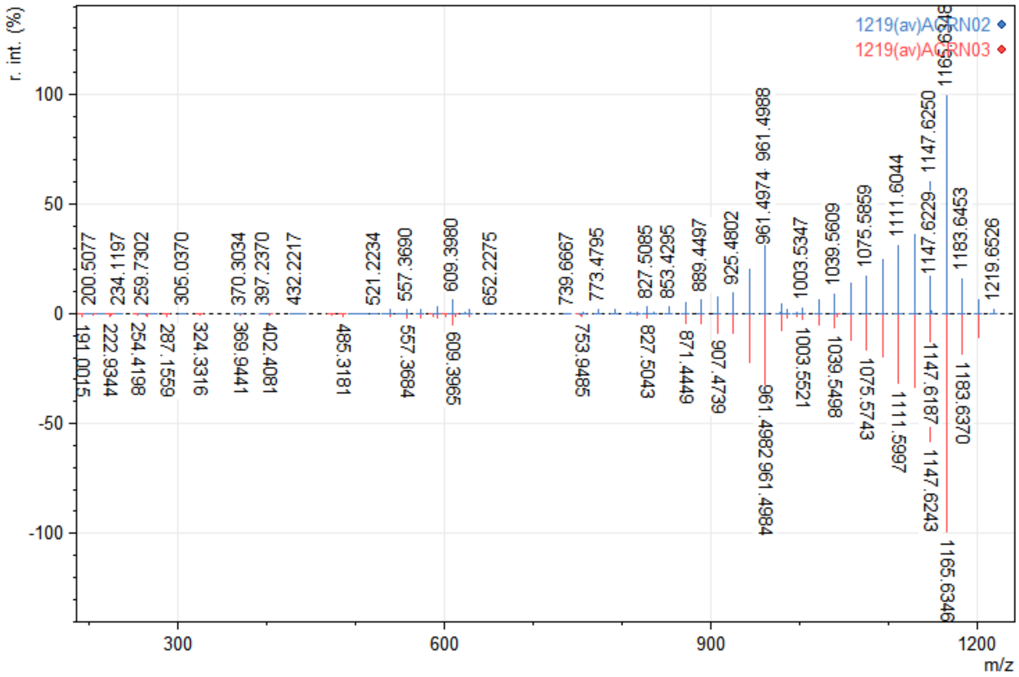

Report S24. Characterization of **amphidinol 35** (*m/z* 1355.76) in **ACRN03**.

Properties

**Exact Mass**	1354.7497 g/mol
**Ion [M+H]+**	*m/z* 1355.7548
**Formula**	$C_{66}H_{114}O_{28}$
**RT**	4.68 min
**C LogP**	–11.4030

Full HRMS Characterization

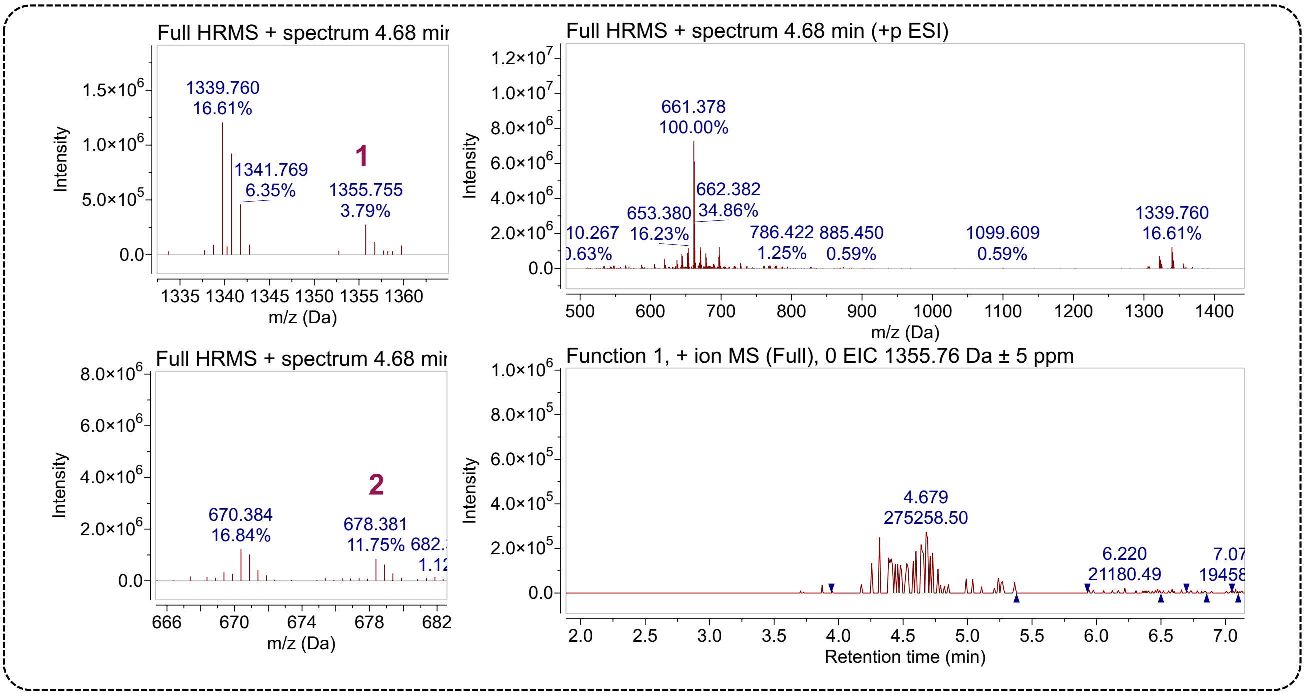

	Ion	Formula	*m/z* theo.	*m/z* exp.	RDB	Δ mDa	Δ ppm
**1**	$[M+H]^+$	$C_{66}H_{115}O_{28}^+$	1355.7569	1355.7548	9.5	–2.18	–1.61
**2**	$[M+2H]^{2+}$	$C_{66}H_{116}O_{28}^{2+}$	678.3821	678.3812	9.0	–1.89	–1.39

MS^2 fragments annotation

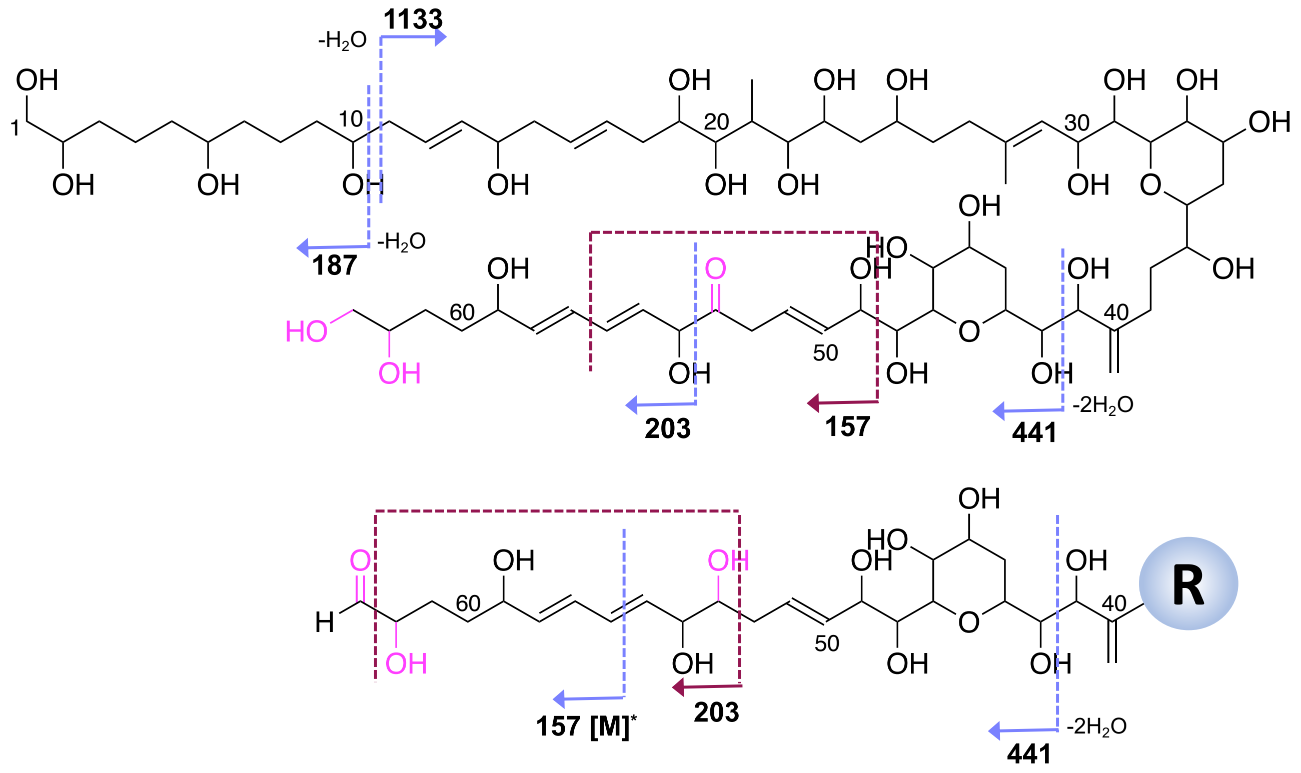

Chemical Formula: $C_{66}H_{114}O_{28}$
Exact Mass: 1354,7497

NOTE: The functional groups shown in red are located in the most likely position according to our approach and hypothesis described in the article.

	clv	Ion	Formula	*m/z* theo.	*m/z* exp.	RDB	Δ ppm	Neutral loss (H_2O)
1		$[M+H–H_2O]^+$	$C_{66}H_{113}O_{27}^+$	1337.7464	1331.7457	10.5	–0.48	10
2	a	$[X+H–H_2O]^+$	$C_{56}H_{93}O_{23}^+$	1133.6102	1133.6032	10.5	–6.24	3
3		$[M+2H–3H_2O]^{2+}$	$C_{66}H_{110}O_{25}^+$	651.3663	651.3662	12.0	–0.17	8
4		$[M+2H–2H_2O]^{2+}$	$C_{56}H_{90}O_{21}^+$	549.2882	549.2980	12.0	–0.28	3
5	s	$[X+H–2H_2O]^+$	$C_{22}H_{33}O_9^+$	441.2119	441.2132	6.5	2.92	2
6	v	$[X+H]^+$	$C_{10}H_{19}O_4^+$	203.1278	203.1283	1.5	2.37	-
7	k	$[X+H–H_2O]^+$	$C_{10}H_{19}O_4^+$	187.1329	187.1326	1.5	–1.22	4
8	w	$[X+H]^+$	$C_8H_{13}O_3^+$	157.0859	157.0858	2.5	–0.97	-

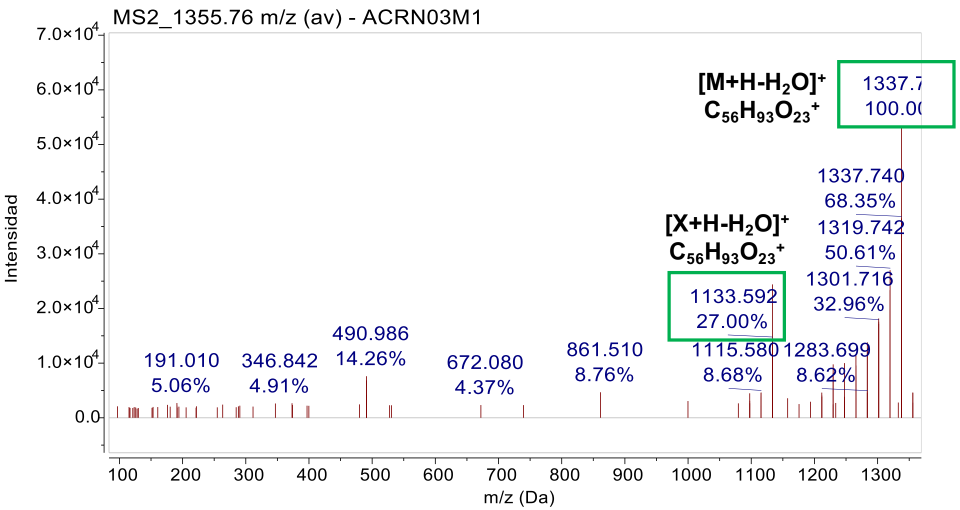
MS2_1355.76 m/z (av) - ACRN03M1
[M+H-H2O]+
C56H93O23+
[X+H-H2O]+
C56H93O23+
Intensidad
m/z (Da)

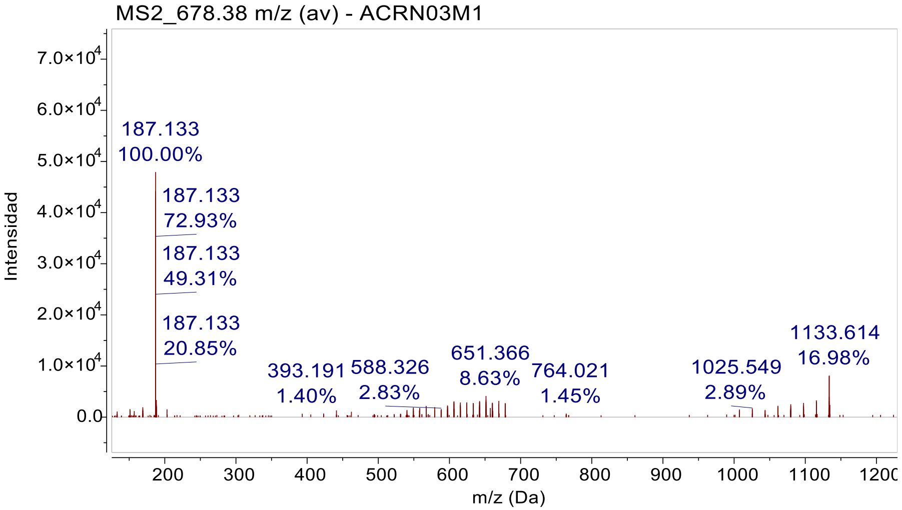
MS2_678.38 m/z (av) - ACRN03M1
Intensidad
m/z (Da)

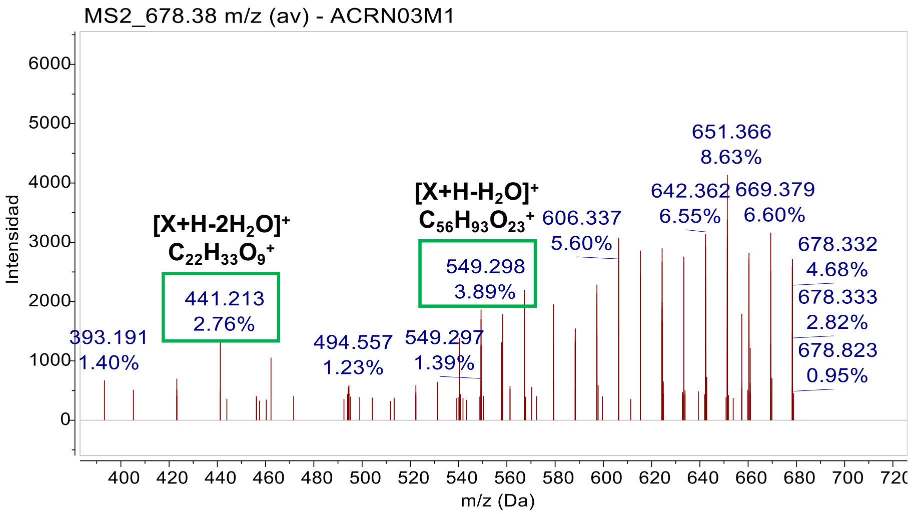
MS2_678.38 m/z (av) - ACRN03M1
[X+H-2H2O]+
C22H33O9+
[X+H-H2O]+
C56H93O23+
Intensidad
m/z (Da)

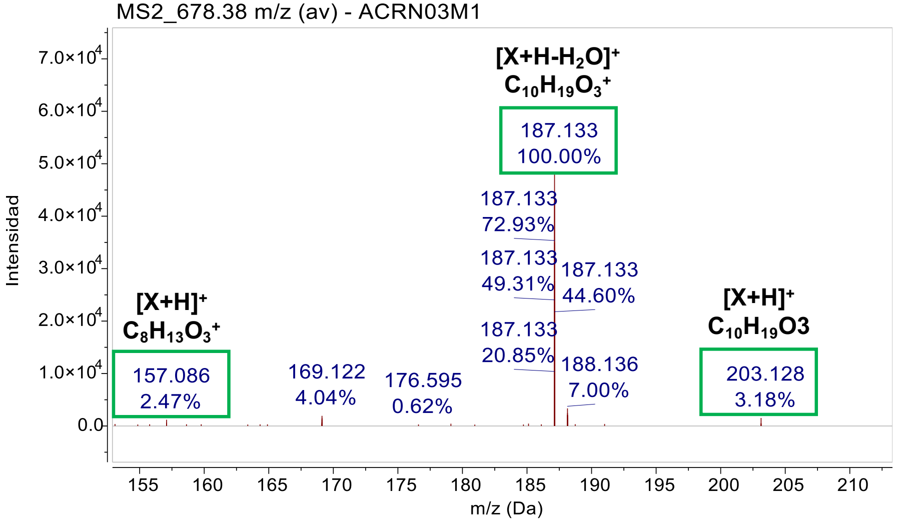
MS2_678.38 m/z (av) - ACRN03M1
[X+H-H2O]+
C10H19O3+
[X+H]+
C8H13O3+
[X+H]+
C10H19O3
Intensidad
m/z (Da)

Report S25. Characterization of **amphidinol 36** (*m/z* 1357.76) in **ACRN03**.

Properties

**Exact Mass**	1356.7653 g/mol
**Ion [M+H]⁺**	*m/z* 1357.7732
**Formula**	$C_{66}H_{116}O_{28}$
**RT**	4.21 min
**C LogP**	–12.3498

Full HRMS Characterization

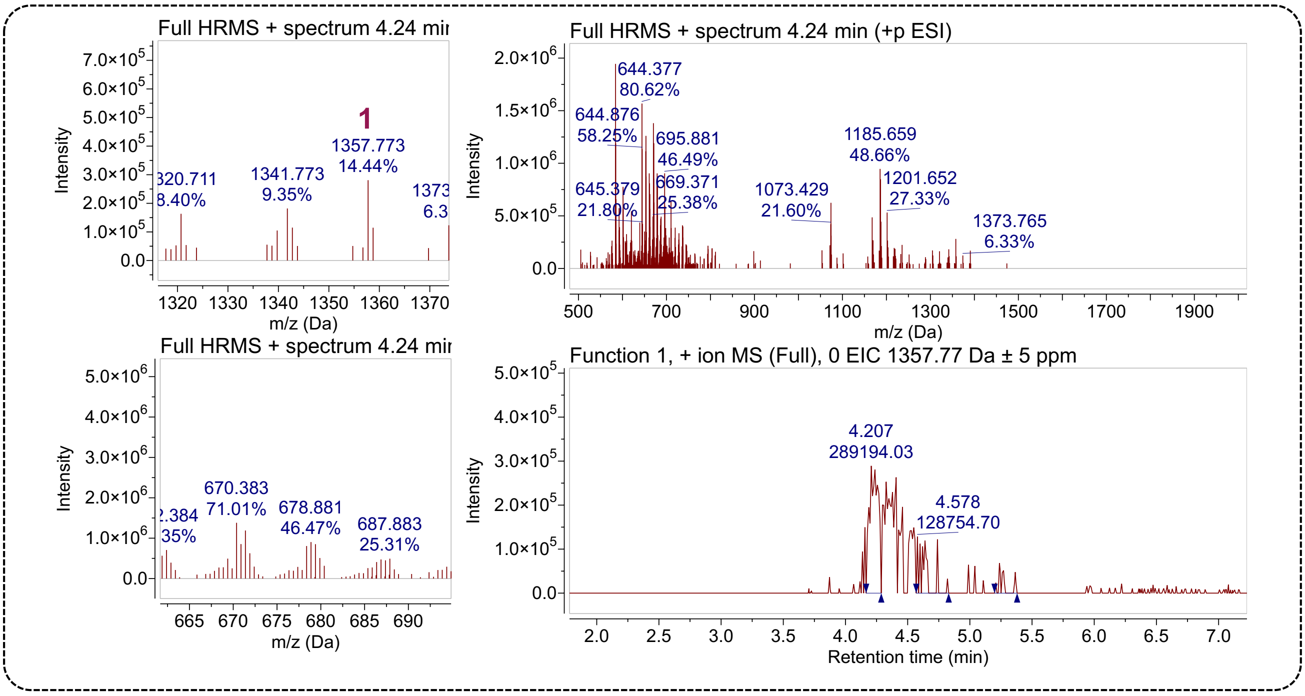

	Ion	Formula	*m/z* theo.	*m/z* exp.	RDB	Δ mDa	Δ ppm
**1**	[M+H]⁺	$C_{66}H_{117}O_{28}^{+}$	1357.7726	1357.7732	8.5	0.60	0.45

MS2 fragments annotation

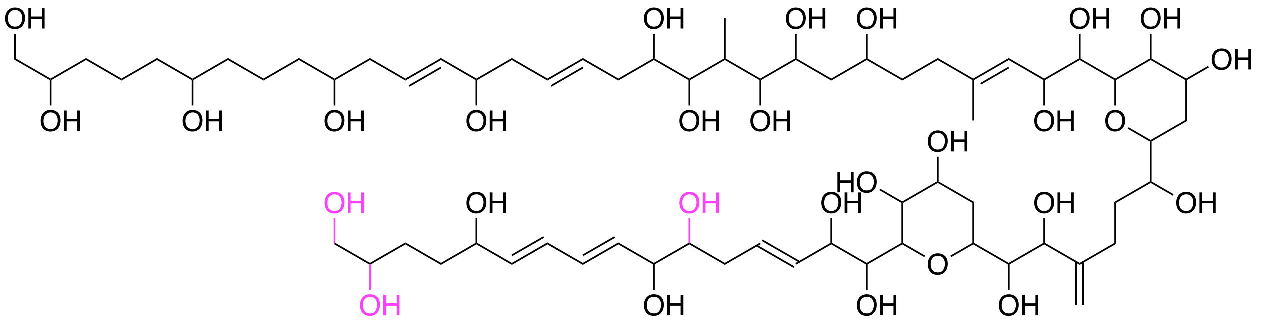

Chemical Formula: $C_{66}H_{116}O_{28}$
Exact Mass: 1356,7653

NOTE: The functional groups shown in red are located in the most likely position according to our approach and hypothesis described in the article.

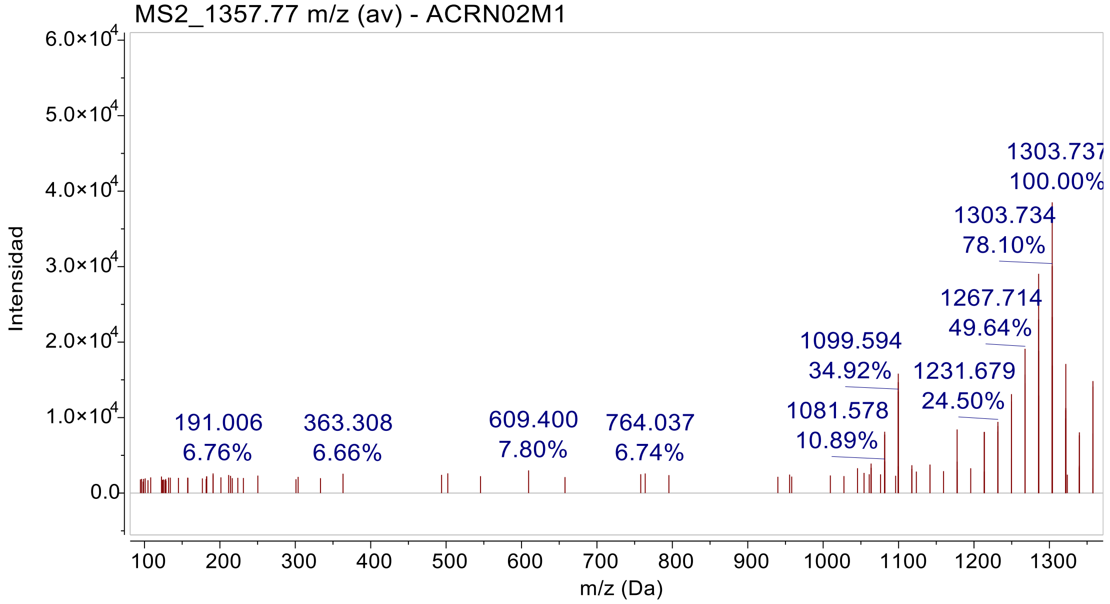

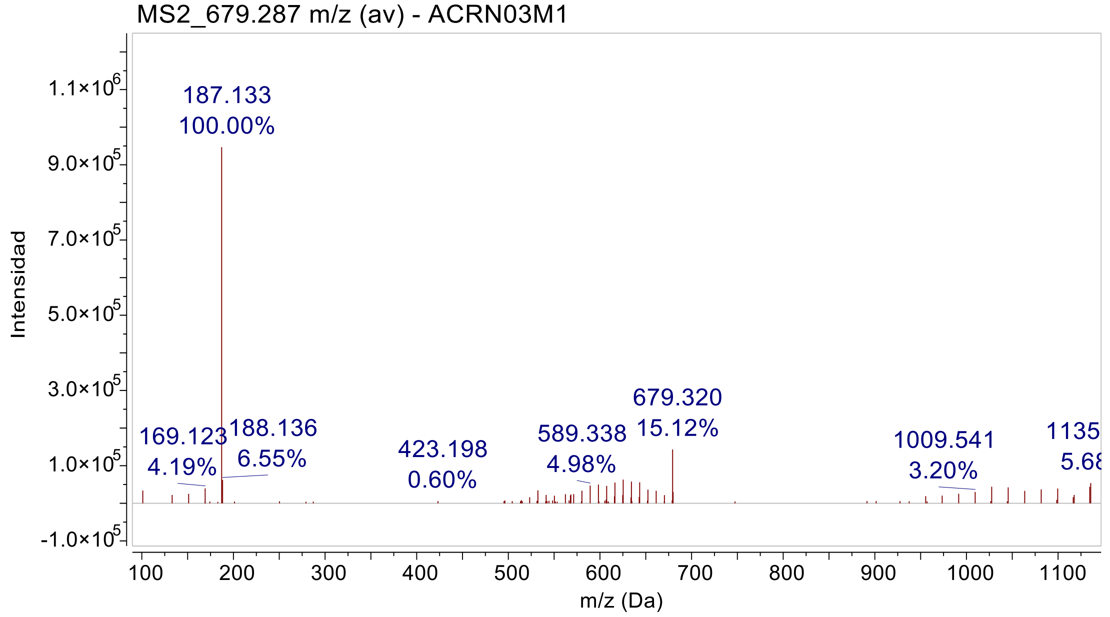

Table S11. Assignment of the internal fragments contained in MS^2 spectra of AMs 28-35 to relevant cleavages (clv).
Elemental formulae of the monoisotopic ion peaks are reported, rings and double bond equivalents (RDB) and error (Δppm) of the corresponding ion fragments obtained from each cleavage.

	Compound	clv	Adduct/Formula	RDB	*m/z* exp.	Δppm
AM 28	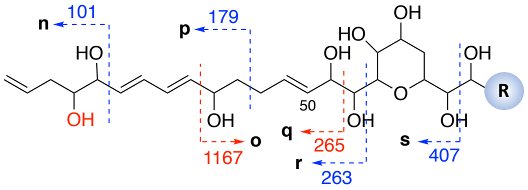	n	$[X]^{\cdot}$; $C_5H_9O_2^{\cdot}$	1.0	101.0601	–1.98
		o	$[X–H]^-$; $C_{57}H_{99}O_{24}^-$	8.5	1167.6473	–5.00
		p	$[X+H–H_2O]^+$; $C_{11}H_{15}O_2^+$	4.5	179.1065	–0.93
		q	$[X–H]^-$; $C_{15}H_{21}O_4^-$	5.5	265.1451	2.30
		r	$[X+H–2H_2O]^+$; $C_{16}H_{23}O_3^+$	5.5	263.1631	–4.14
		s	$[X+H–2H_2O]^+$; $C_{22}H_{31}O_7^+$	7.5	407.2052	–2.95
AM 29	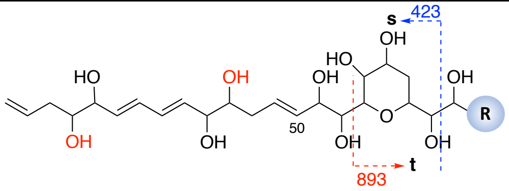	s	$[X+H–2H_2O]^+$; $C_{22}H_{31}O_8^+$	7.5	423.2003	–2.43
		t	$[X–H]^-$; $C_{44}H_{77}O_{18}^-$	6.5	893.5120	0.54
AM 35	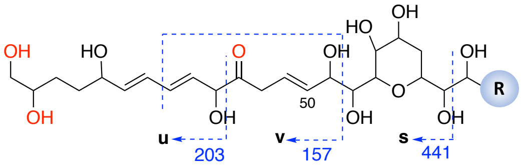	s	$[X+H–2H_2O]^+$; $C_{22}H_{33}O_9^+$	6.5	441.2132	2.92
		u	$[X+H]^+$; $C_{10}H_{19}O_4^+$	1.5	203.1283	2.37
		v	$[X+H]^+$; $C_8H_{13}O_3^+$	2.5	157.0858	–0.97
AM 30	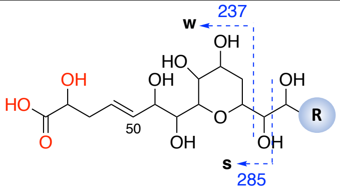	s	$[X+H–2H_2O]^+$; $C_{13}H_{17}O_7^+$	5.5	285.0968	–0.17
		w	$[X+H–3H_2O]^+$; $C_{12}H_{13}O_5^+$	7.0	237.0753	–2.09

Negative ion mode (ESI–, HCD 22%) as [M+HCOO]⁻ ions

Figure S28. Family of amphidinols in cell-free medium extract of **ACRN02** Colored by retention time.

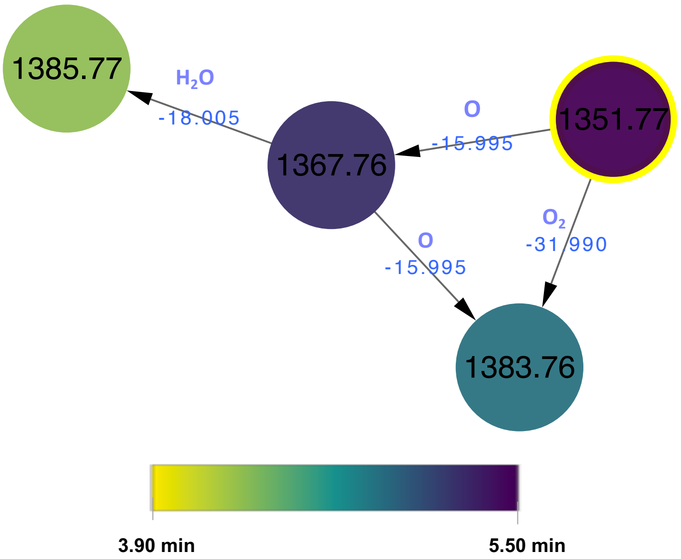

Report S26. Characterization of **luteophanol D** (*m/z* 1351.77) in **ACRN02**

Properties

**Exact Mass**	1306.7649 g/mol
**Ion [M+HCOO]⁻**	*m/z* 1351.7664
**Formula**	$C_{66}H_{114}O_{25}$
**RT**	4.91 min
**C LogP**	–8.1384

Full HRMS Identification

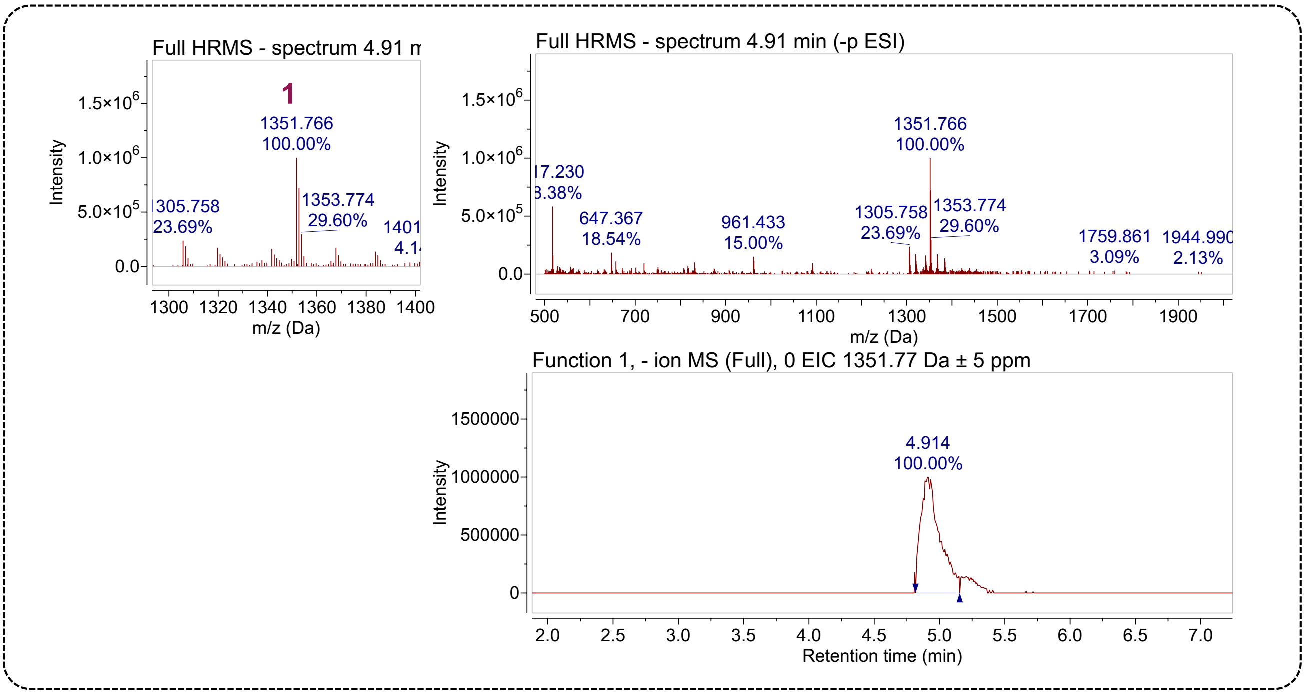

	Ion	Formula	*m/z* theo.	*m/z* exp.	RDB	Δ mDa	Δ ppm
**1**	[M+HCOO]⁻	$C_{67}H_{115}O_{27}^{-}$	1351.7631	1351.7664	10.5	3.30	2.44

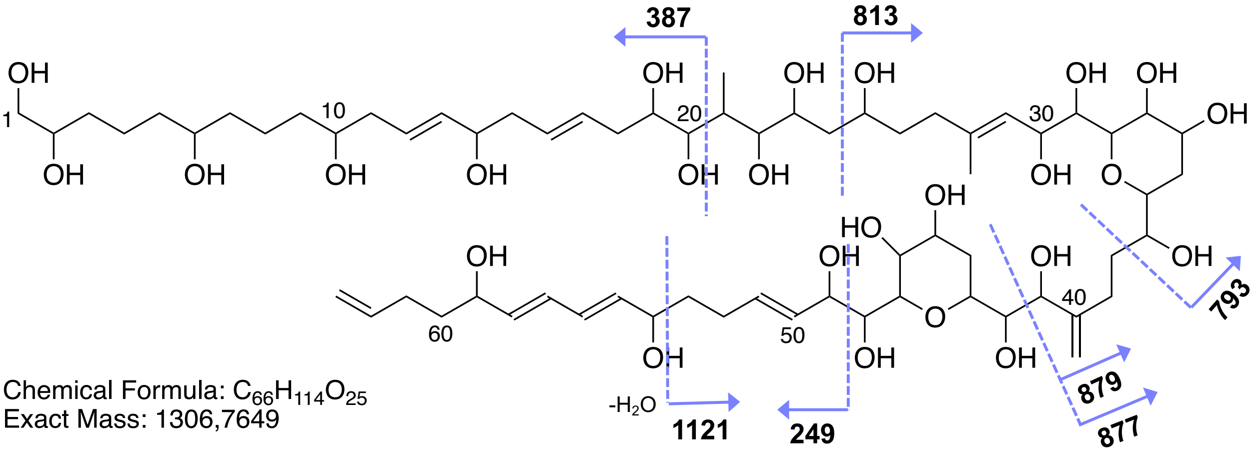

Chemical Formula: $C_{66}H_{114}O_{25}$
Exact Mass: 1306,7649

MS^2 fragments annotation

	clv	Ion	Formula	*m/z* theo.	*m/z* exp.	RDB	Δ ppm
1		$[M{-}H]^-$	$C_{66}H_{113}O_{25}^-$	1305.7576	1305.7584	10.5	0.60
2		$[M{-}H{-}H_2O]^-$	$C_{66}H_{111}O_{24}^-$	1287.7471	1287.7488	11.5	1.32
3		$[X{-}H{-}H_2O]^-$	$C_{56}H_{97}O_{22}^-$	1121.6477	1121.6414	8.5	–5.65
4	b	$[X{-}H]^-$	$C_{44}H_{79}O_{17}^-$	879.5323	879.5329	5.5	0.71
5	b	$[X{-}H]^-$	$C_{44}H_{77}O_{17}^-$	877.5166	877.5169	6.5	0.25
6	d	$[X{-}H]^-$	$C_{41}H_{65}O_{16}^-$	813.4278	813.4294	9.5	1.93
7		$[X{-}H]^-$	$C_{39}H_{69}O_{16}^-$	793.4591	793.4584	5.5	–0.85
8	h	$[X{-}H]^-$	$C_{20}H_{35}O_7^-$	387.2388	387.2391	3.5	0.72
9	q	$[X{-}H]^-$	$C_{15}H_{21}O_3^-$	249.1496	249.1500	5.5	1.45

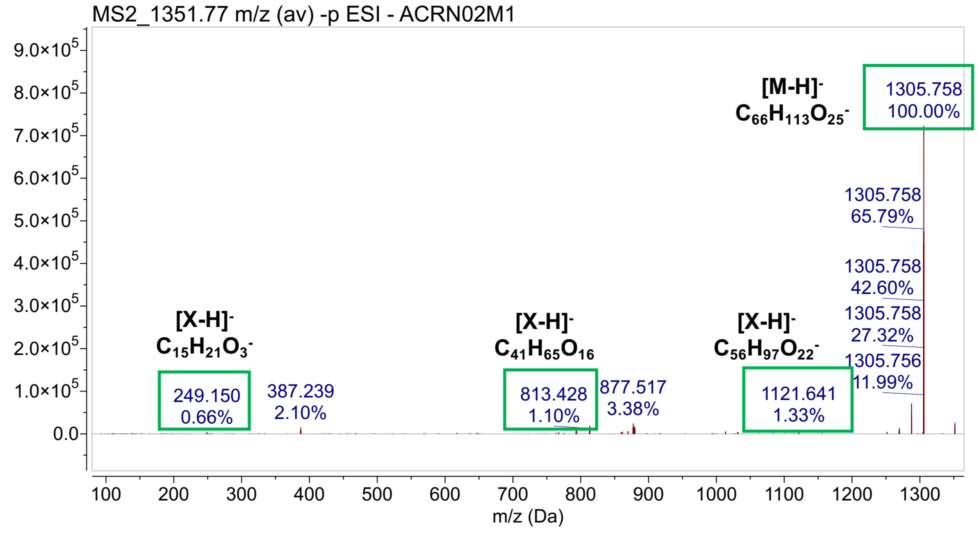

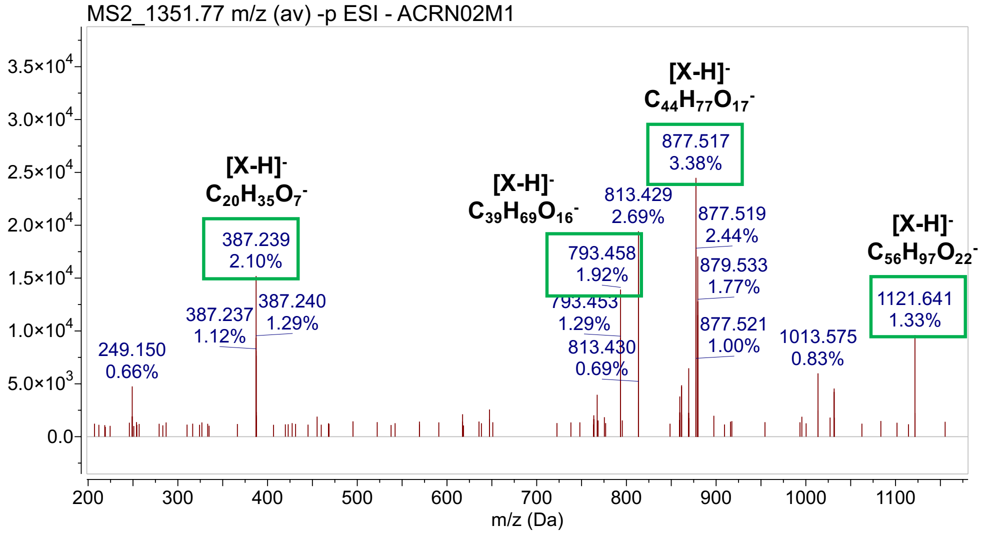

Report S27. Characterization of **amphidinol 28** (*m/z* 1367.76; RT 4.54) in **ACRN02**

Properties

**Exact Mass**	1322.7598 g/mol
**Ion [M+HCOO]⁻**	*m/z* 1367.7612
**Formula**	$C_{66}H_{114}O_{26}$
**RT**	4.54 min
**C LogP**	–9.4926

Full HRMS Identification

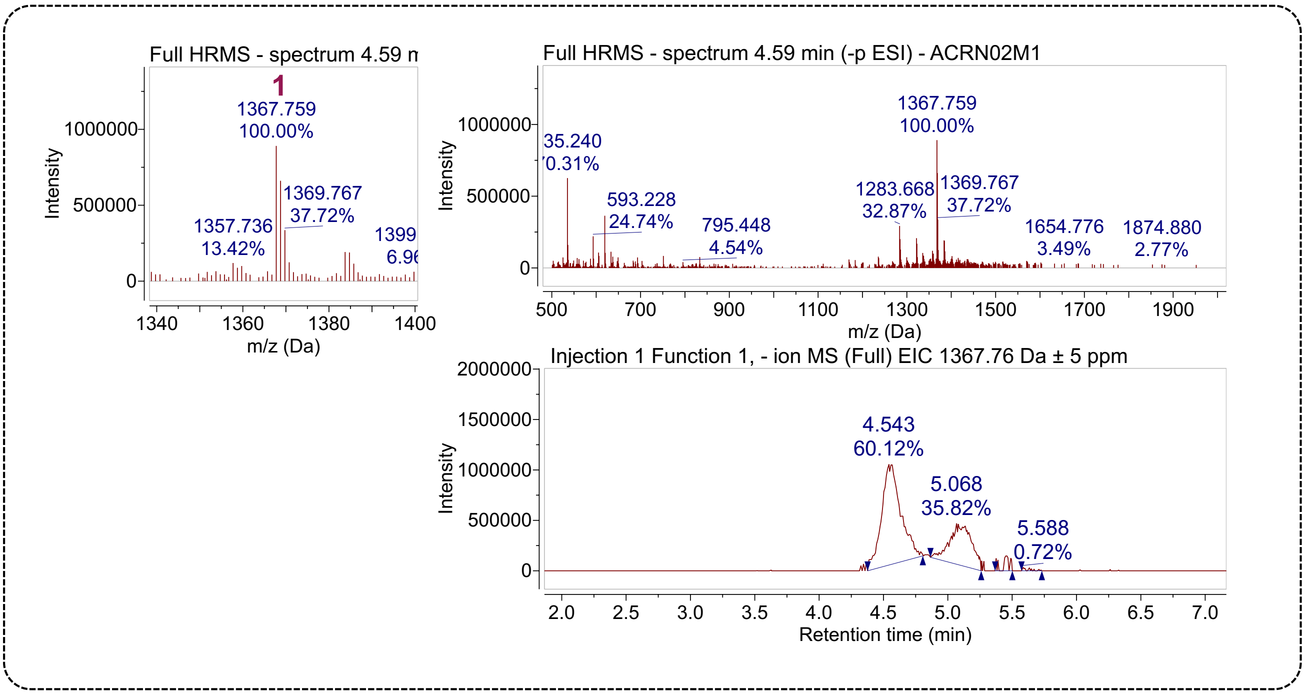

	Ion	Formula	*m/z* theo.	*m/z* exp.	RDB	Δ mDa	Δ ppm
**1**	[M+HCOO]⁻	$C_{67}H_{115}O_{28}^{-}$	1367.7580	1367.7589	10.5	0.90	0.65

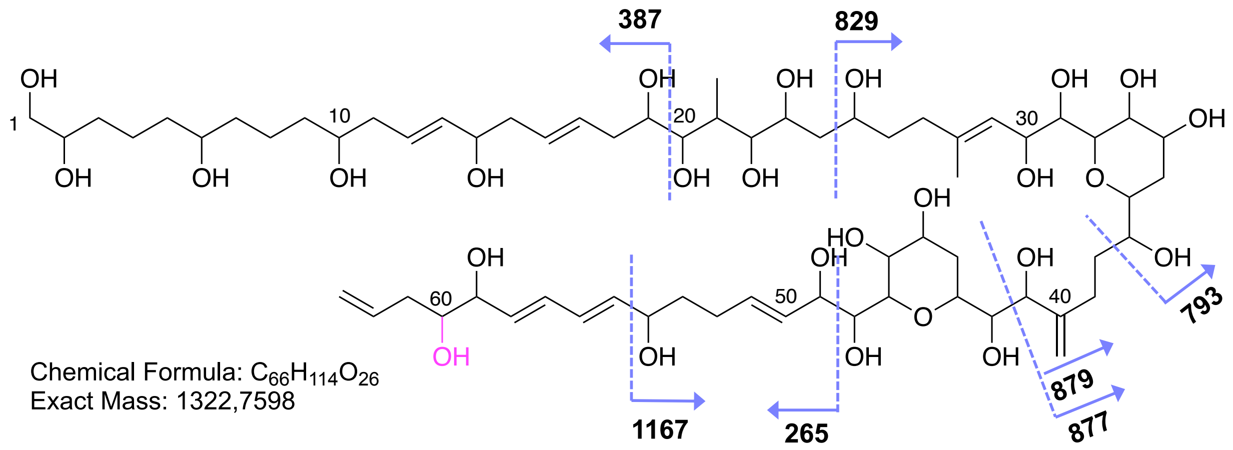

Chemical Formula: $C_{66}H_{114}O_{26}$
Exact Mass: 1322,7598

NOTE: The functional groups shown in red are located in the most likely position according to our approach and hypothesis described in the article.

MS^2 fragments annotation

	clv	Ion	Formula	*m/z* theo.	*m/z* exp.	RDB	Δ ppm
1		[M–H]⁻	$C_{66}H_{113}O_{26}^-$	1321.7526	1321.7539	10.5	1.02
2		[M–H]⁻	$C_{66}H_{111}O_{25}^-$	1303.7420	1303.7421	11.5	0.06
3	o	[X–H]⁻	$C_{57}H_{99}O_{24}^-$	1167.6532	1167.6473	8.5	–5.00
5	b	[X–H]⁻	$C_{44}H_{79}O_{17}^-$	879.5323	879.5331	5.5	0.92
6	b	[X–H]⁻	$C_{44}H_{77}O_{17}^-$	877.5166	877.5206	6.5	4.49
7	d	[X–H]⁻	$C_{41}H_{65}O_{17}^-$	829.4227	829.4264	9.5	4.42
8		[X–H]⁻	$C_{39}H_{69}O_{16}^-$	793.4591	793.4620	5.5	3.69
9	h	[X–H]⁻	$C_{20}H_{35}O_{7}^-$	387.2388	387.2404	3.5	4.03
10	q	[X–H]⁻	$C_{15}H_{21}O_{4}^-$	265.1445	265.1451	5.5	2.30

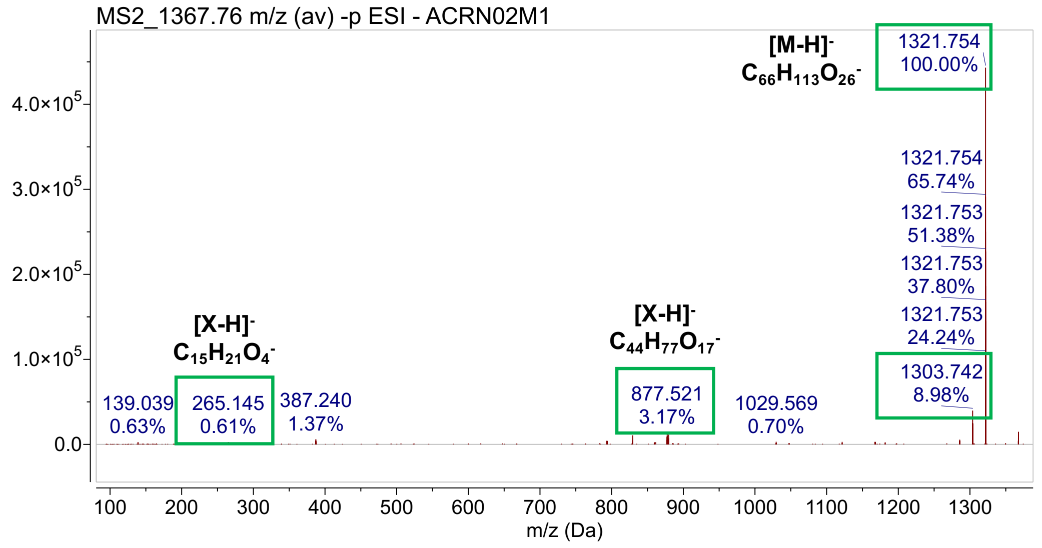

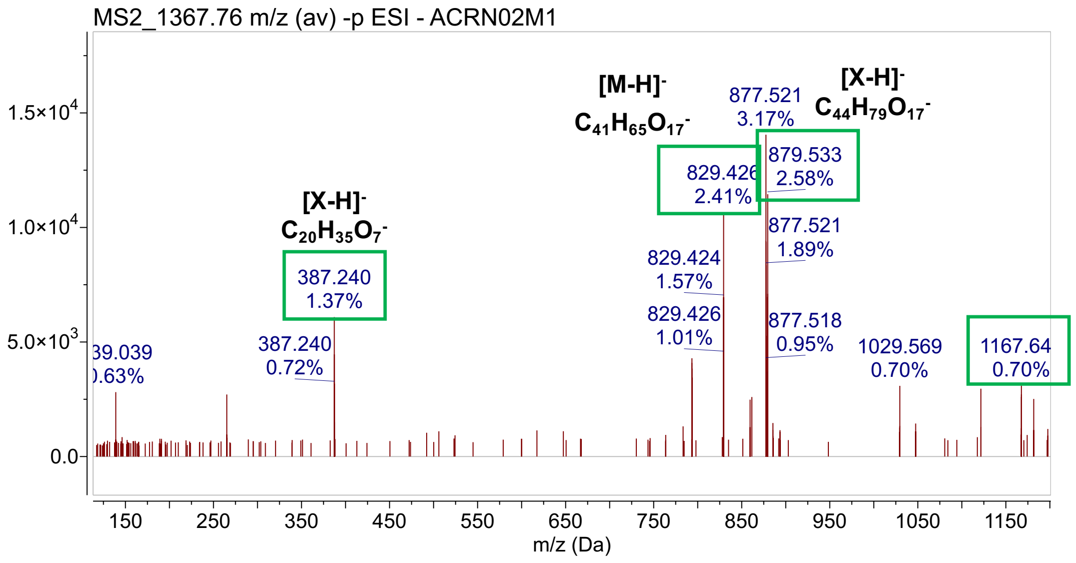

Report S28. Characterization of **amphidinol 20B** (*m/z* 1367.76, RT 5.07) in **ACRN02**

Properties

**Exact Mass**	1322.7598 g/mol
**Ion [M+HCOO]⁻**	*m/z* 1367.7612
**Formula**	$C_{66}H_{114}O_{26}$
**RT**	5.07 min
**C LogP**	–9.4926

Full HRMS Identification

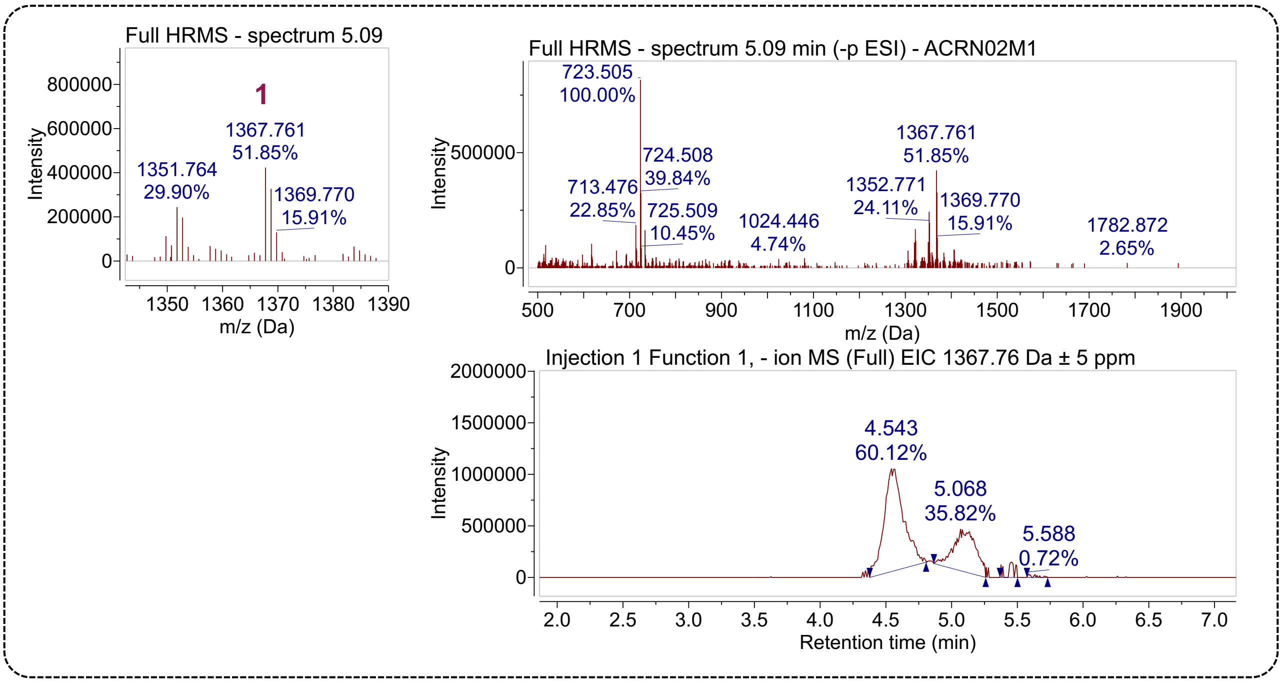

	Ion	Formula	*m/z* theo.	*m/z* exp.	RDB	Δ mDa	Δ ppm
**1**	[M+HCOO]⁻	$C_{67}H_{115}O_{28}^{-}$	1367.7580	1367.7612	10.5	3.20	2.34

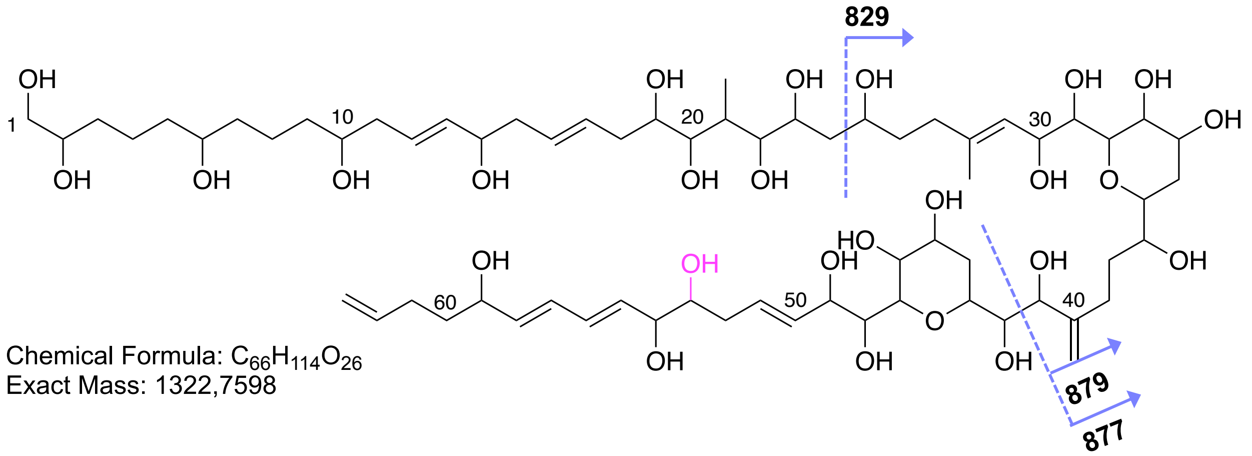

Chemical Formula: $C_{66}H_{114}O_{26}$
Exact Mass: 1322,7598

NOTE: The functional groups shown in red are located in the most likely position according to our approach and hypothesis described in the article.

MS² fragments annotation

	clv	Ion	Formula	*m/z* theo.	*m/z* exp.	RDB	Δ ppm
1		$[M–H]^–$	$C_{66}H_{113}O_{26}^–$	1321.7526	1321.7534	10.5	0.65
2		$[X–H]^–$	$C_{66}H_{111}O_{25}^–$	1303.7420	1303.7421	11.5	1.93
3	b	$[X–H]^–$	$C_{44}H_{79}O_{17}^–$	879.5323	879.5355	5.5	3.62
4	b	$[X–H]^–$	$C_{44}H_{77}O_{17}^–$	877.5166	877.5206	6.5	3.62
5	d	$[X–H]^–$	$C_{41}H_{65}O_{17}^–$	829.4227	829.4264	9.5	3.62

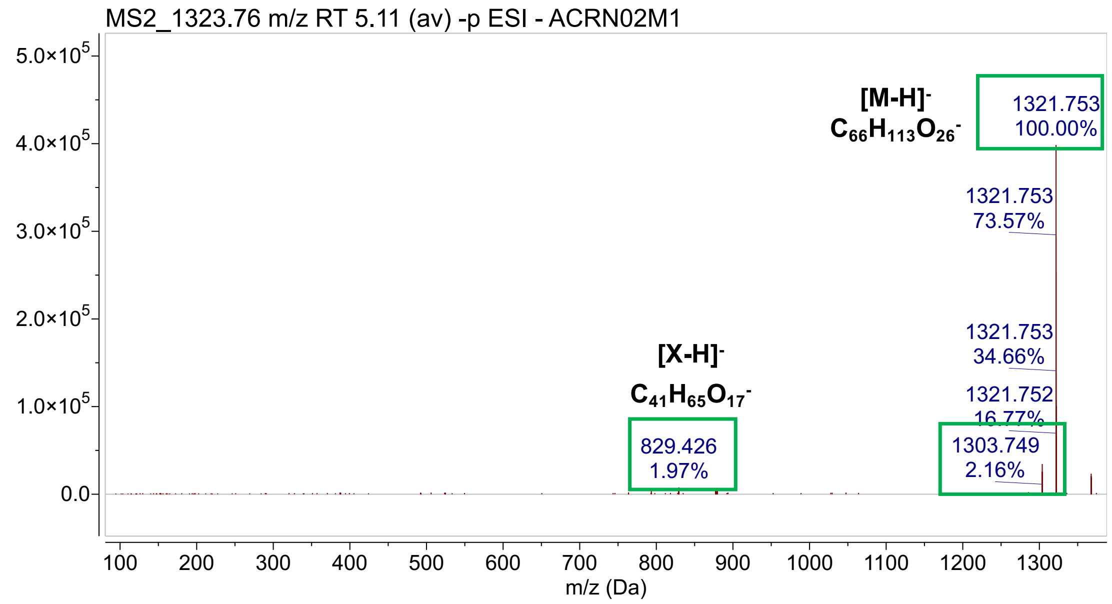

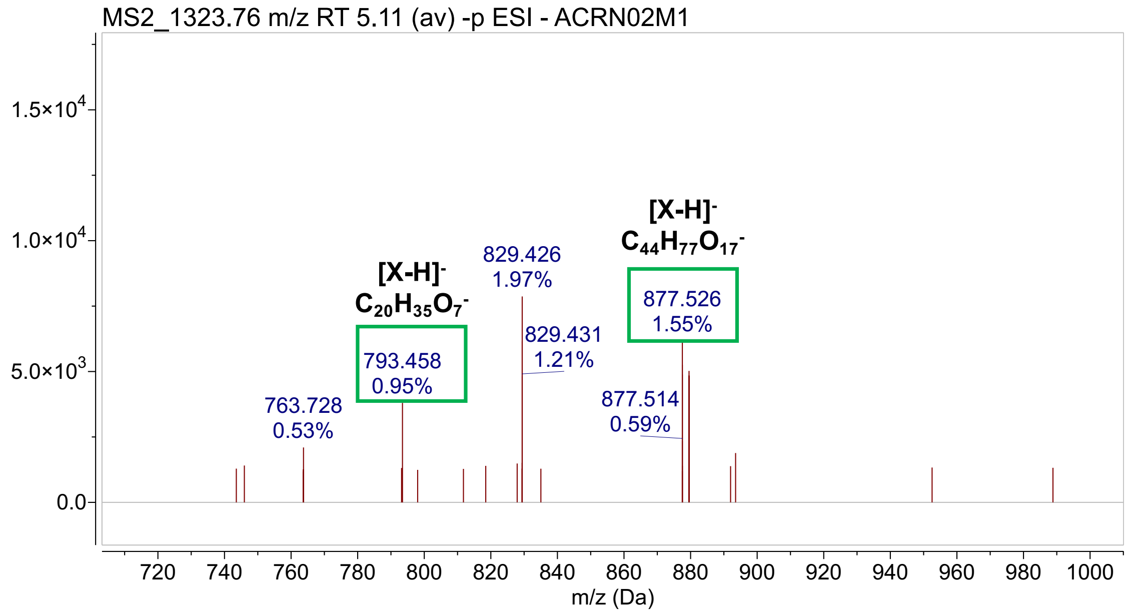

NOTE: Signal 793 describes fragment C1–37, but it is rejected because its intensity is low.

Report S29. Characterization of **amphidinol 29** (*m/z* 1383.75) in **ACRN02**

Properties

**Exact Mass**	1338.7547g/mol
**Ion [M+HCOO]⁻**	*m/z* 1383.7557
**Formula**	$C_{66}H_{114}O_{27}$
**RT**	4.70 min
**C LogP**	–10.8568

Full HRMS Identification

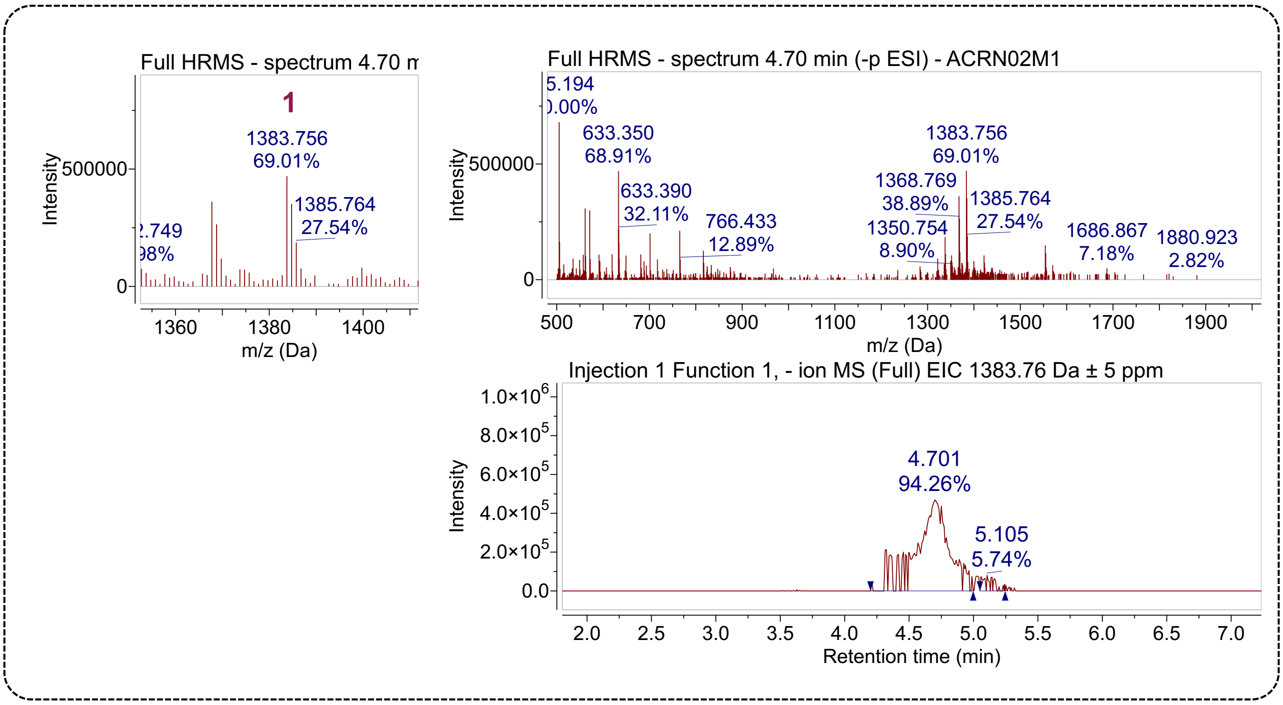

	Ion	Formula	*m/z* theo.	*m/z* exp.	RDB	Δ mDa	Δ ppm
**1**	[M+HCOO]⁻	$C_{67}H_{115}O_{29}^{-}$	1383.7529	1383.7557	10.5	2.80	2.02

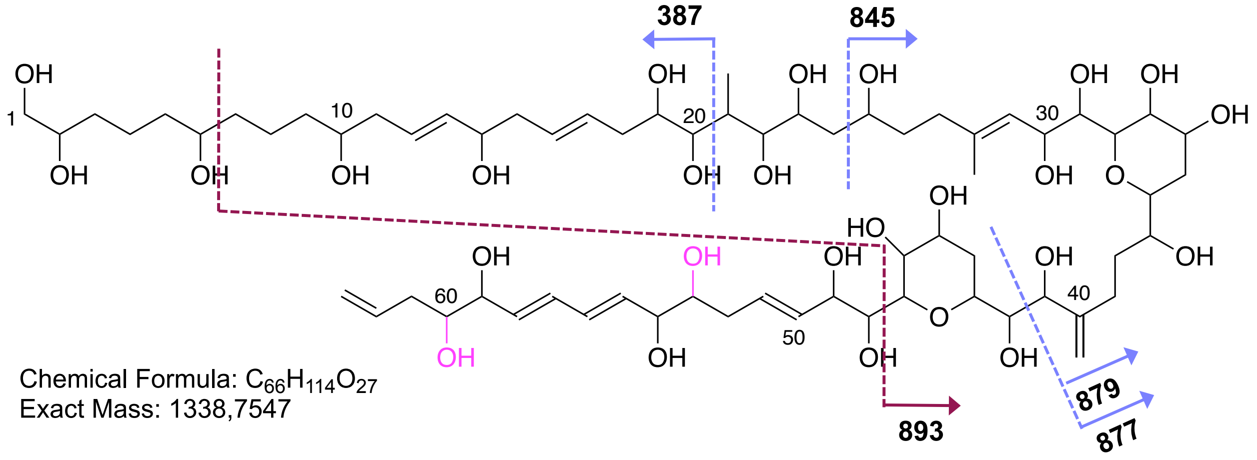

Chemical Formula: $C_{66}H_{114}O_{27}$
Exact Mass: 1338,7547

NOTE: The functional groups shown in red are located in the most likely position according to our approach and hypothesis described in the article.

MS^2 fragment annotation

	clv	Ion	Formula	*m/z* theo.	*m/z* exp.	RDB	Δ ppm
1		$[M–H]^–$	$C_{66}H_{113}O_{27}^–$	1337.7475	1337.7483	10.5	0.61
2		$[M–H–H_2O]^–$	$C_{66}H_{111}O_{26}^–$	1319.7369	1319.7401	11.5	2.43
3		$[X–H]^–$	$C_{44}H_{77}O_{18}^–$	893.5115	893.5120	6.5	0.54
4	b	$[X–H]^–$	$C_{44}H_{79}O_{17}^–$	879.5323	879.5314	5.5	–0.96
5	b	$[X–H]^–$	$C_{44}H_{77}O_{17}^–$	877.5166	877.5190	6.5	2.76
6	d	$[X–H]^–$	$C_{41}H_{65}O_{18}^–$	845.4176	845.4191	9.5	1.76
7	h	$[X–H]^–$	$C_{20}H_{35}O_7^–$	387.2388	387.2401	3.5	3.32

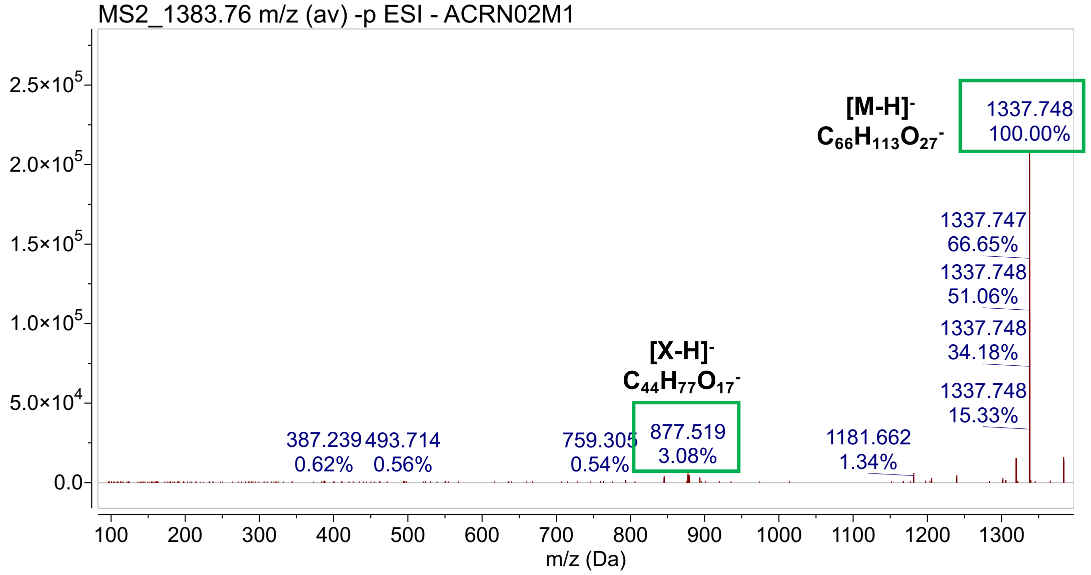

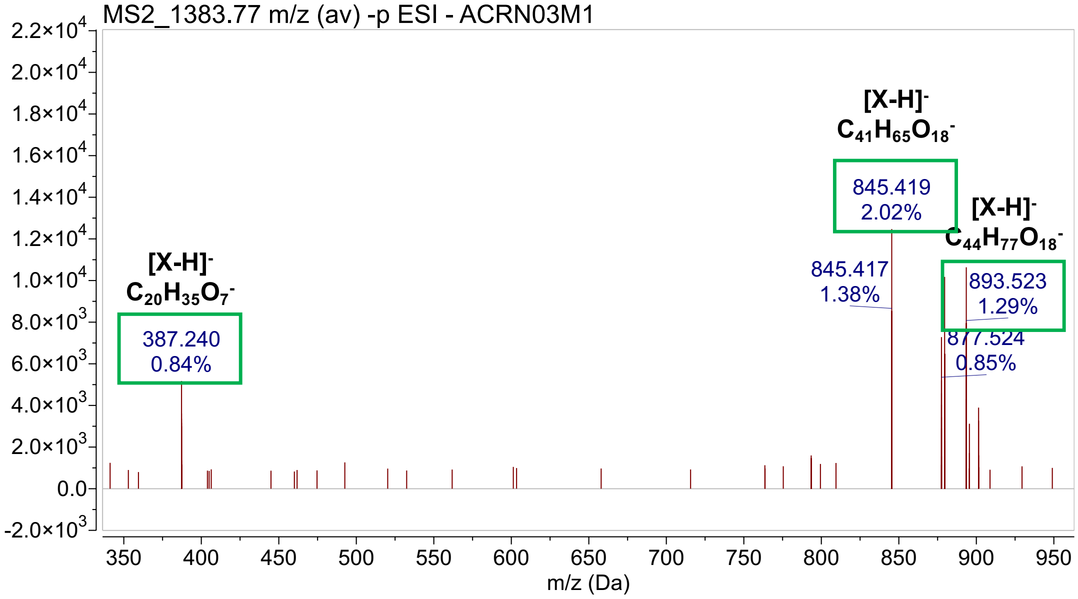

Report S30. Characterization of **amphidinol 24** (*m/z* 1385.77) in **ACRN02.**

Properties

**Exact Mass**	1340.7704 g/mol
**Ion [M+HCOO]⁻**	*m/z* 1385.7694
**Formula**	$C_{66}H_{116}O_{27}$
**RT**	4.45 min
**C LogP**	–10.9956

Full HRMS Identification

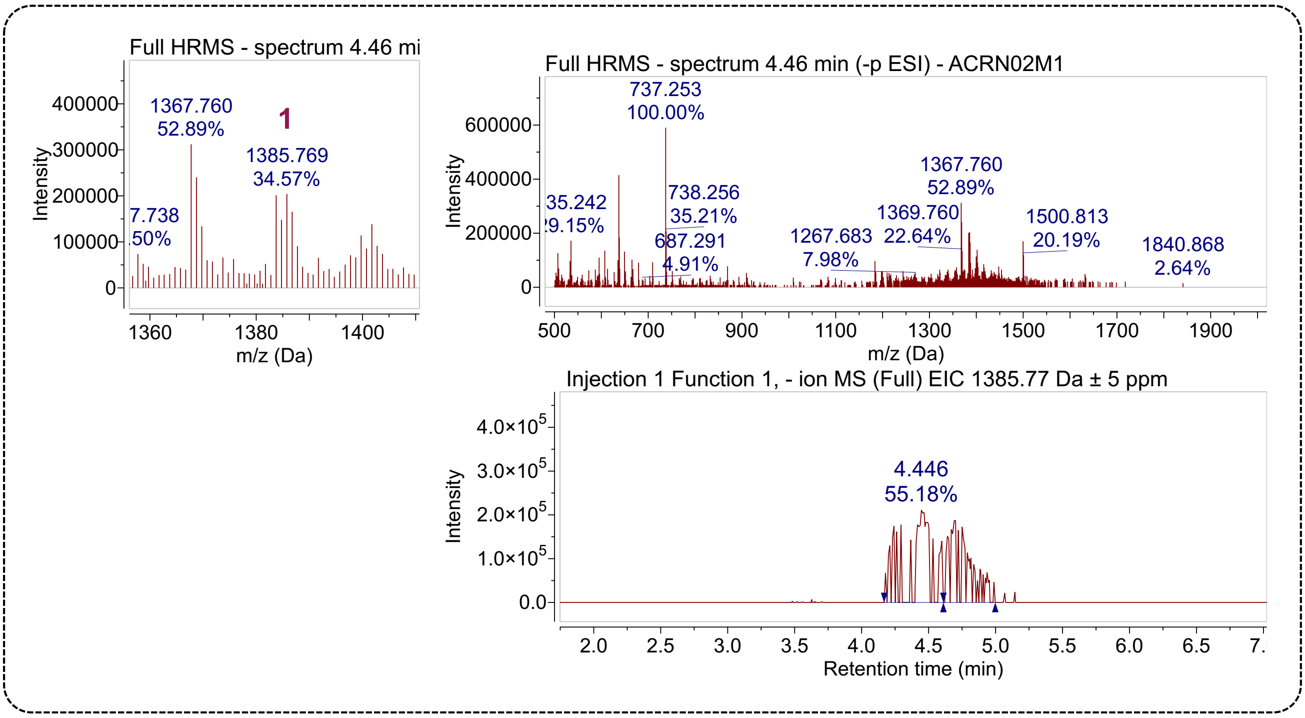

	Ion	Formula	*m/z* theo.	*m/z* exp.	RDB	Δ mDa	Δ ppm
**1**	[M+HCOO]⁻	$C_{67}H_{117}O_{29}^-$	1385.7686	1385.7694	9.5	0.80	0.57

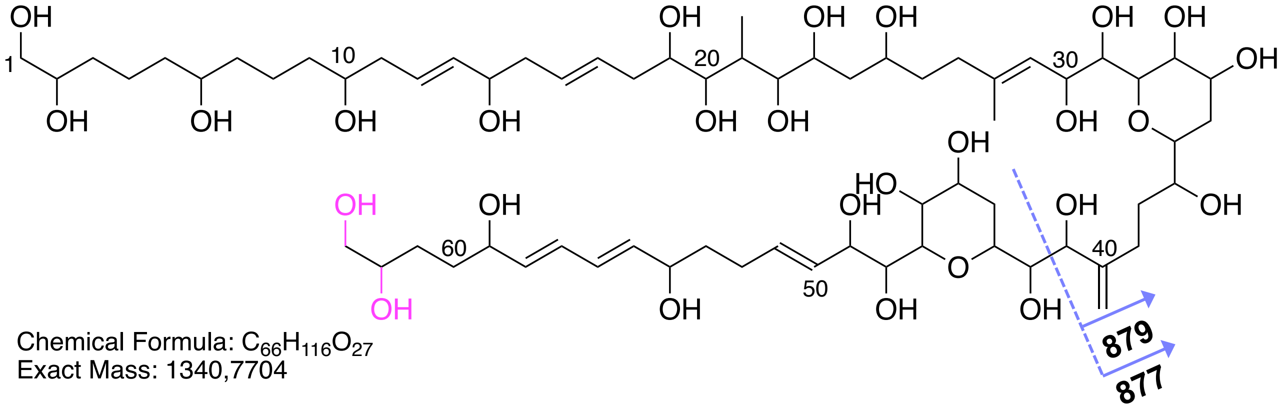

Chemical Formula: $C_{66}H_{116}O_{27}$
Exact Mass: 1340,7704

NOTE: The functional groups shown in red are located in the most likely position according to our approach and hypothesis described in the article.

MS^2 fragments annotation

	clv	Ion	Formula	*m/z* theo.	*m/z* exp.	RDB	Δ ppm
1		$[M–H]^–$	$C_{66}H_{115}O_{27}^–$	1339.7631	1339.7615	9.5	–1.23
2		$[M–H–H_2O]^–$	$C_{66}H_{113}O_{26}^–$	1321.7526	1321.7545	10.5	1.48
3	b	$[X–H]^–$	$C_{44}H_{79}O_{17}^–$	879.5323	879.5289	5.5	–3.80
4	b	$[X–H]^–$	$C_{44}H_{77}O_{17}^–$	877.5166	877.5173	6.5	0.74

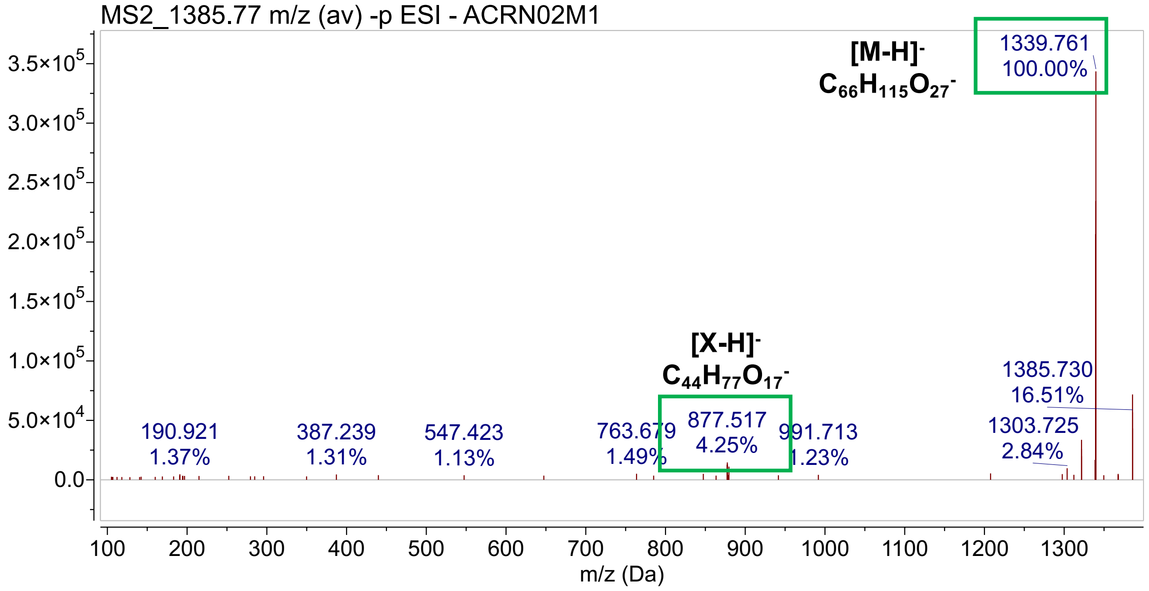

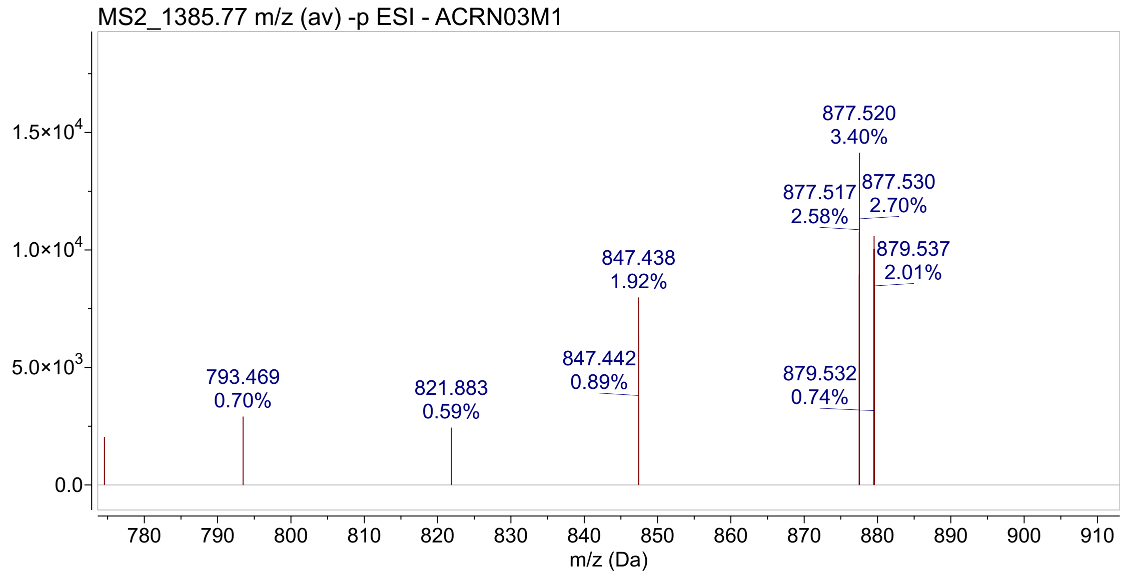

NOTE: Signal 847 defines fragment C25-C66 being a typical signal for these structures in negative ionization fragmentation, however it was rejected because of its intensity.

Figure S29. Family of amphidinols in cell-free medium extract of **ACRN03.** Colored by retention time.

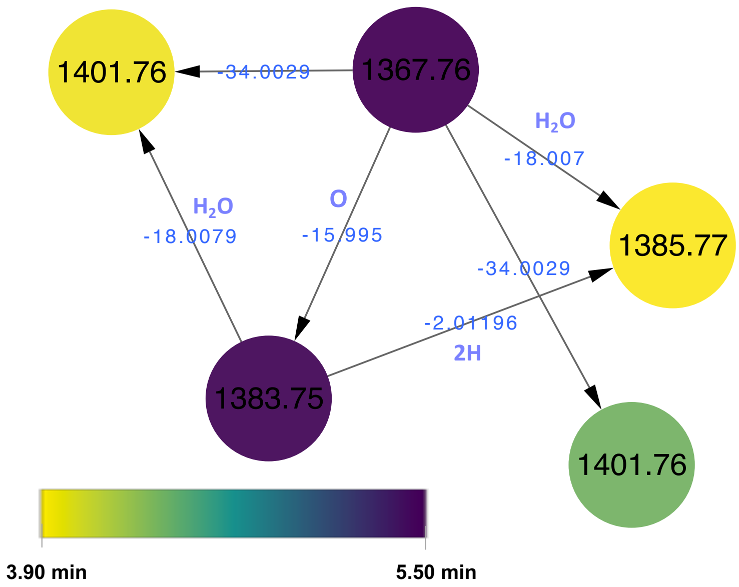

Report S31. Identification of **amphidinol 28** (*m/z* 1367.76) in **ACRN03**.

**RT**	4.53 min

Full HRMS Identification

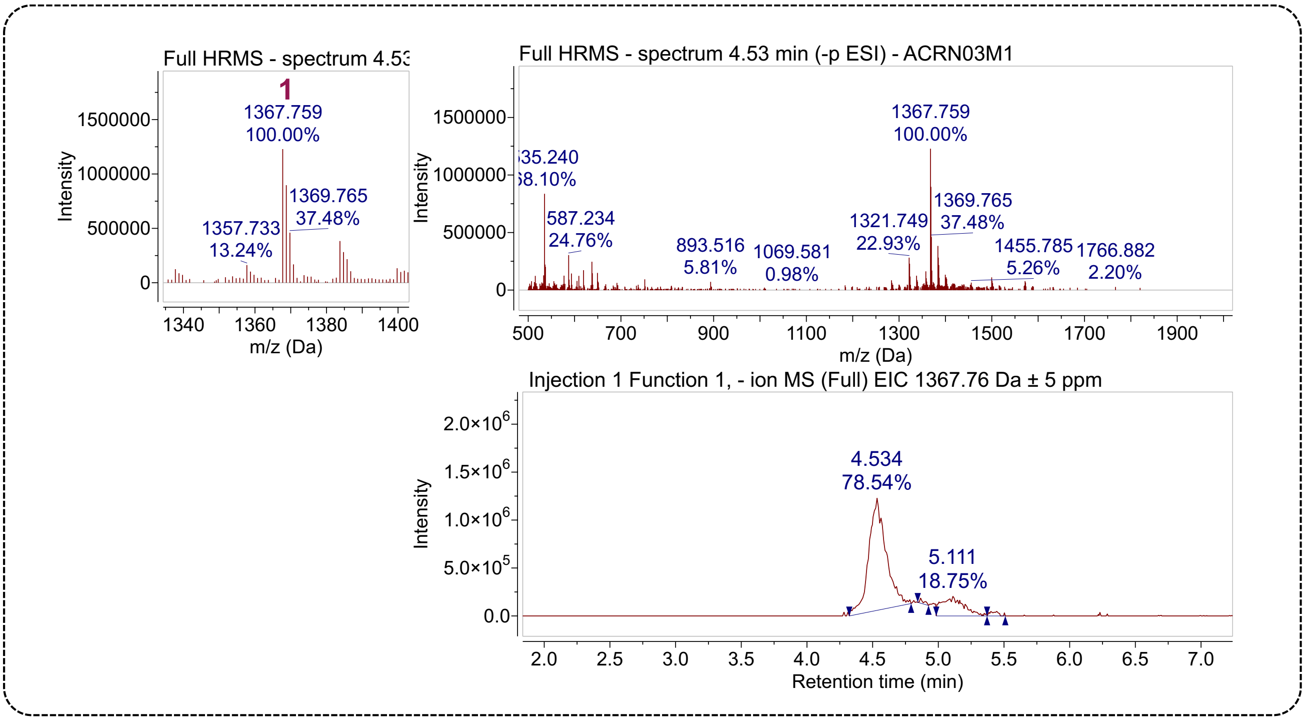

	Ion	Formula	*m/z* theo.	*m/z* exp.	RDB	Δ mDa	Δ ppm
**1**	$[M+HCOO]^-$	$C_{67}H_{115}O_{28}^-$	1367.7580	1367.7588	10.5	0.80	0.58

MS² comparison

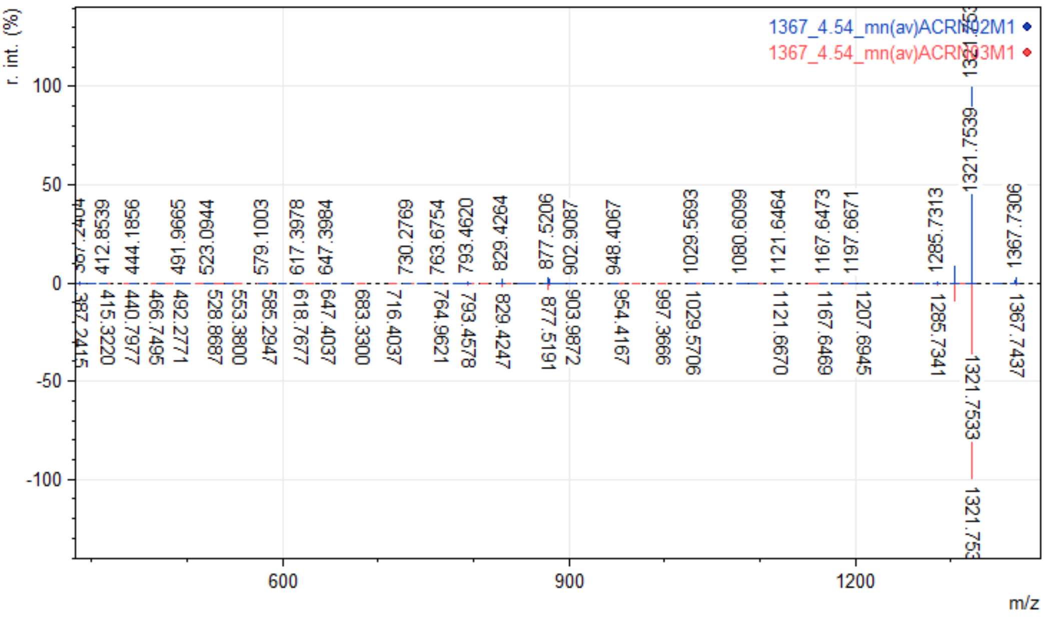

Report S32. Identification of **amphidinol 29** (*m/z* 1383.76) in **ACRN03**.

**RT**	4.72 min

Full HRMS Identification

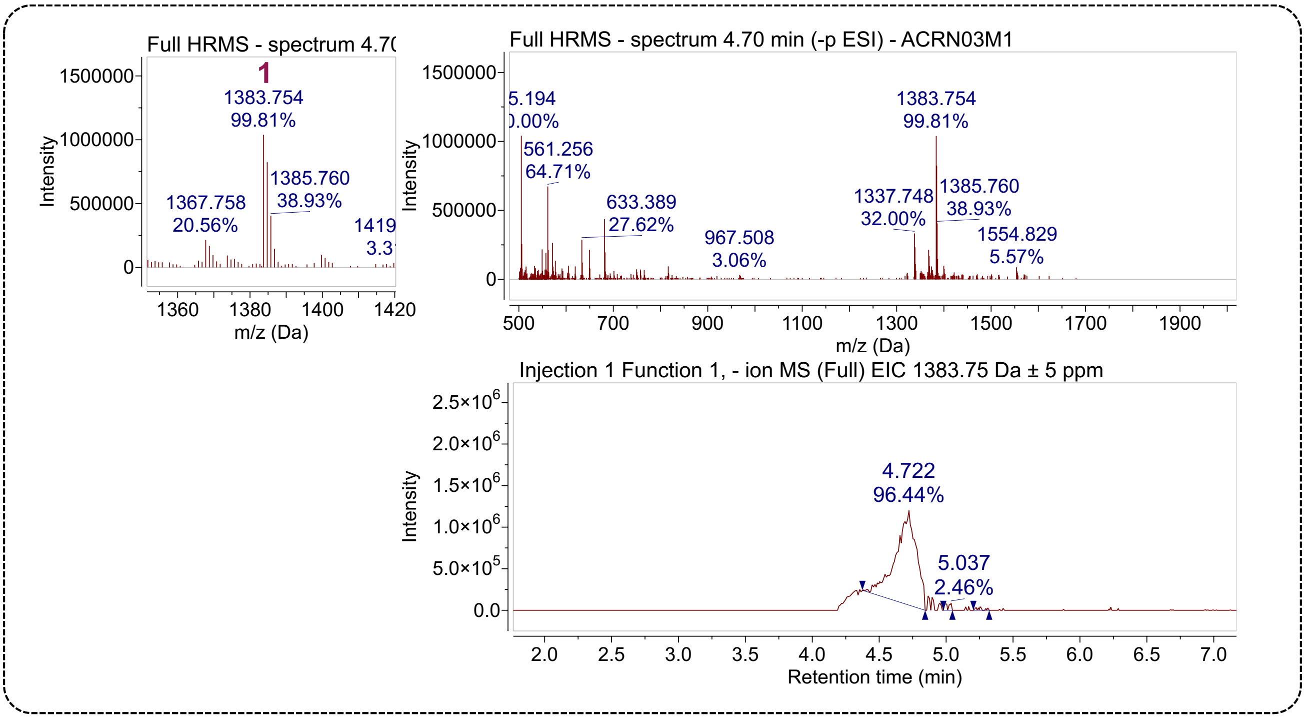

	Ion	Formula	*m/z* theo.	*m/z* exp.	RDB	Δ mDa	Δ ppm
**1**	[M+HCOO]⁻	$C_{66}H_{114}O_{27}^{-}$	1383.7529	1383.7544	10.5	1.54	1.08

MS² comparison

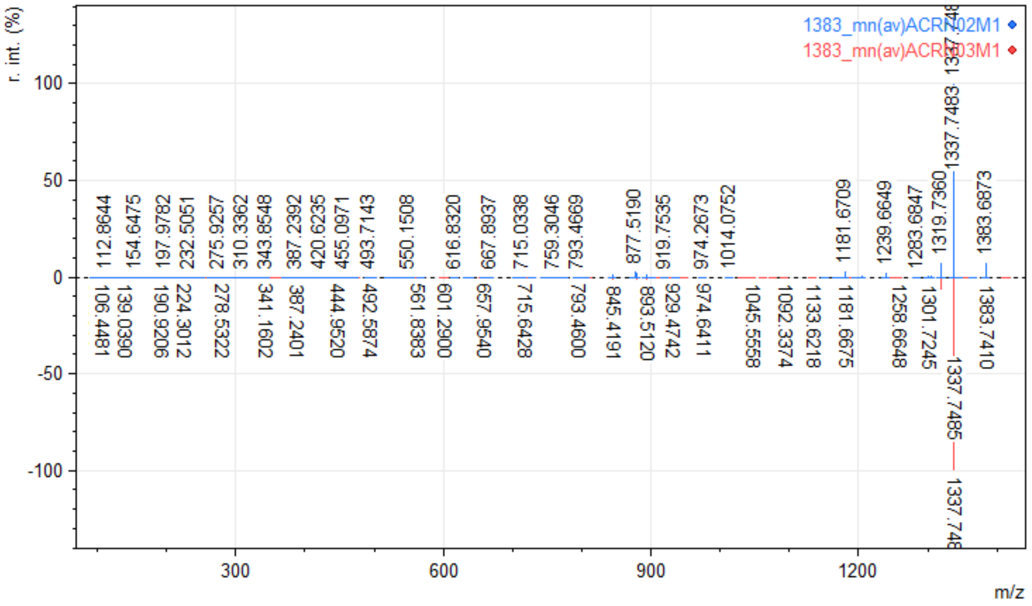

Report S33. Identification of **amphidinol 24** (*m/z* 1385.77) in **ACRN03**.

**RT**	4.25 min

Full HRMS Identification

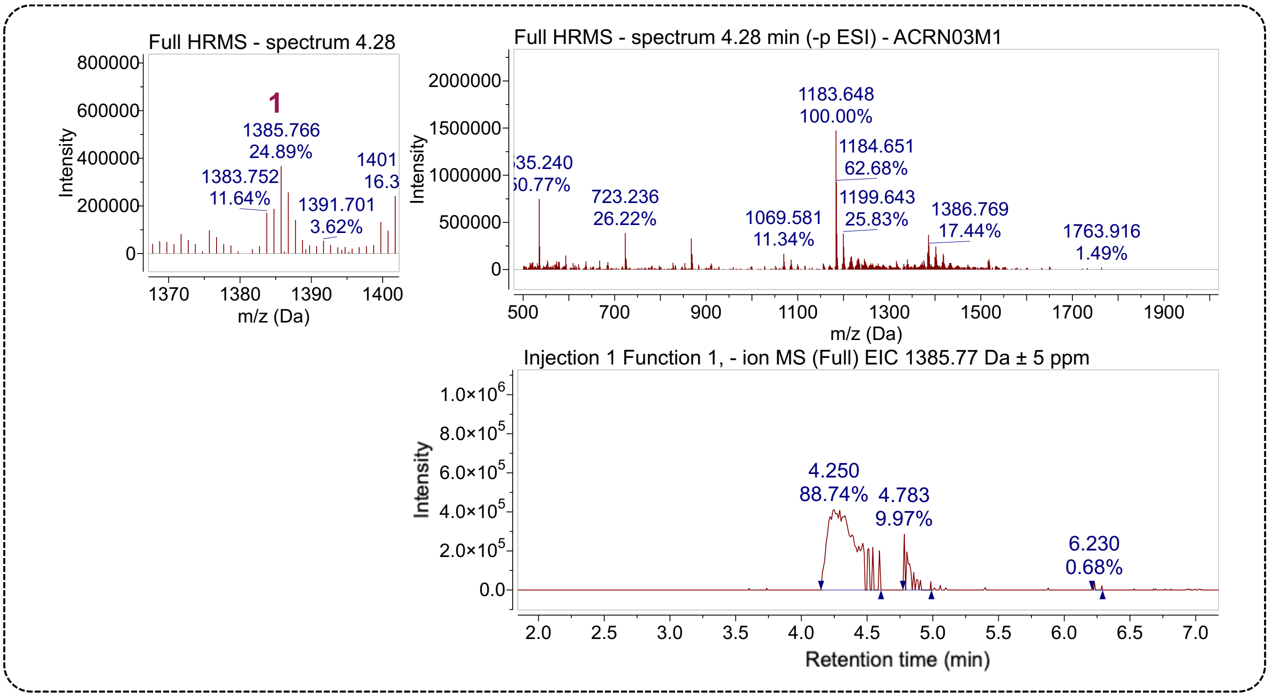

	Ion	Formula	*m/z* theo.	*m/z* exp.	RDB	Δ mDa	Δ ppm
**1**	[M+HCOO]⁻	$C_{67}H_{117}O_{29}^{-}$	1385.7686	1385.7661	9.5	2.50	1.80

MS^2 comparison

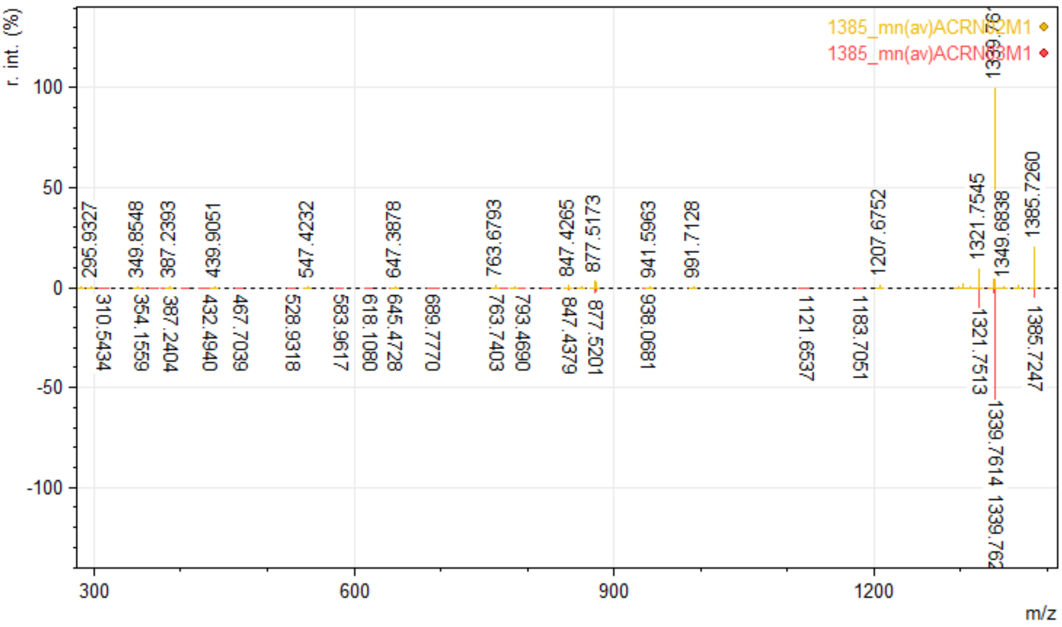

Report S34. Characterization of **amphidinol 36** (*m/z* 1401.76) in **ACRN03**.

Properties

**Exact Mass**	1356.7653 g/mol
**Ion [M+HCOO]⁻**	*m/z* 1401.7622
**Formula**	$C_{66}H_{116}O_{28}$
**RT**	4.24 min
**C logP**	–12.3498

Full HRMS Identification

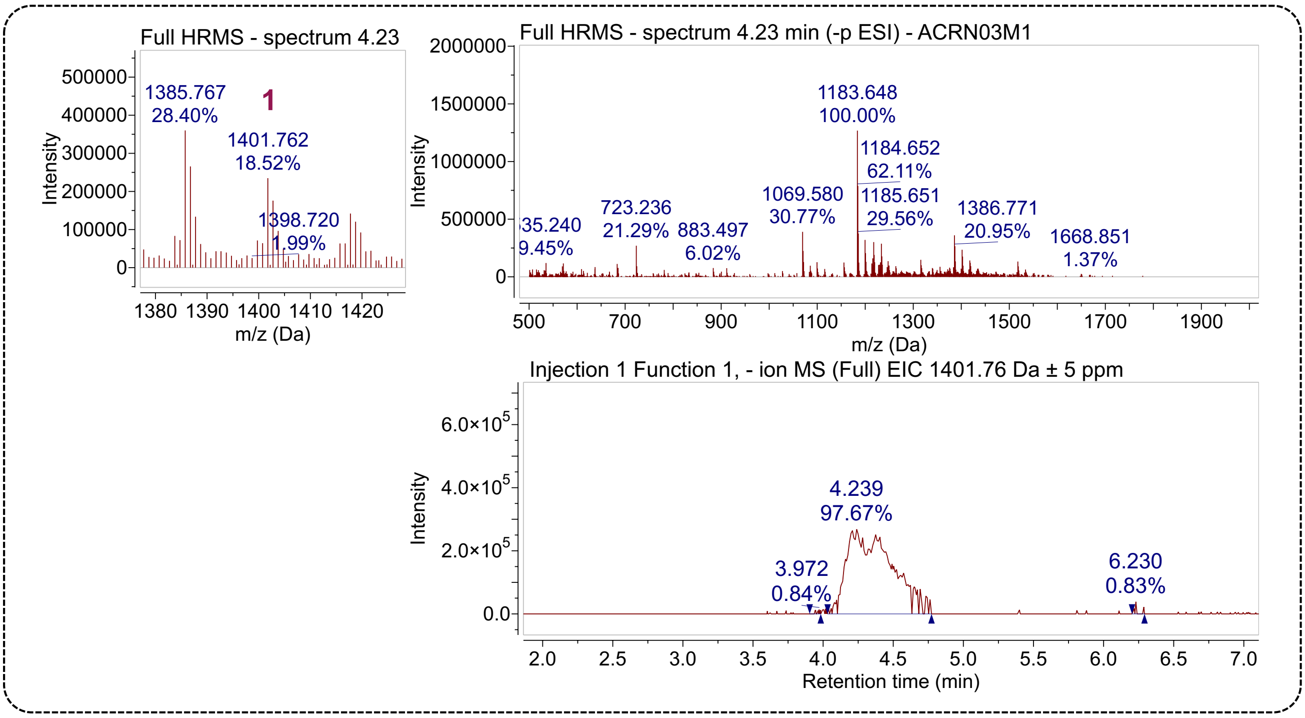

	Ion	Formula	*m/z* theo.	*m/z* exp.	RDB	Δ mDa	Δ ppm
**1**	[M+HCOO]⁻	$C_{67}H_{117}O_{30}^{-}$	1401.7635	1401.7622	9.5	-1.30	-0.93

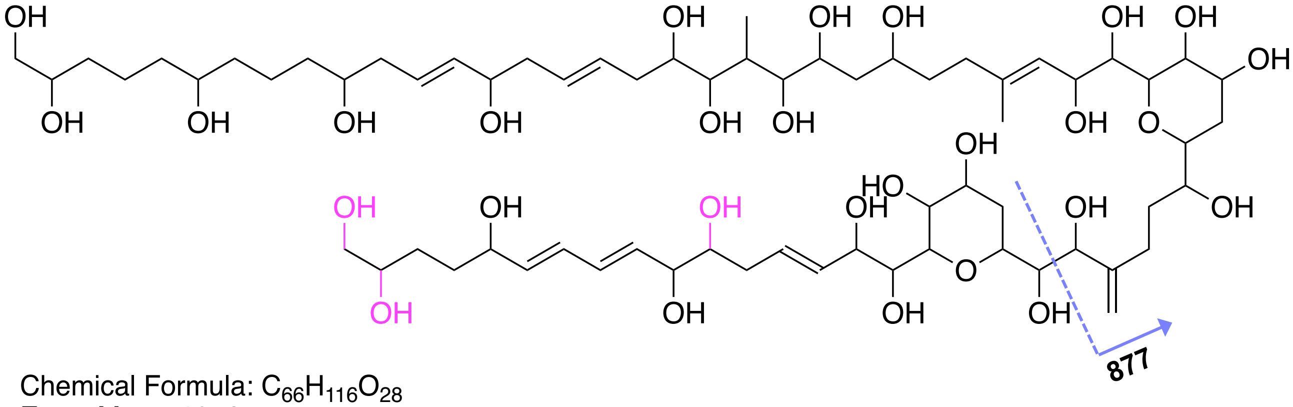

Chemical Formula: $C_{66}H_{116}O_{28}$
Exact Mass: 1356,77

MS^2 fragments annotation

	clv	Ion	Formula	*m/z* theo.	*m/z* exp.	RDB	Δ ppm
1		$[M–H]^–$	$C_{66}H_{115}O_{28}^–$	1355.7580	1355.7570	9.5	–0.80
2	b	$[X–H]^–$	$C_{44}H_{79}O_{17}^–$	877.51662	877.51477	6.5	–2.11

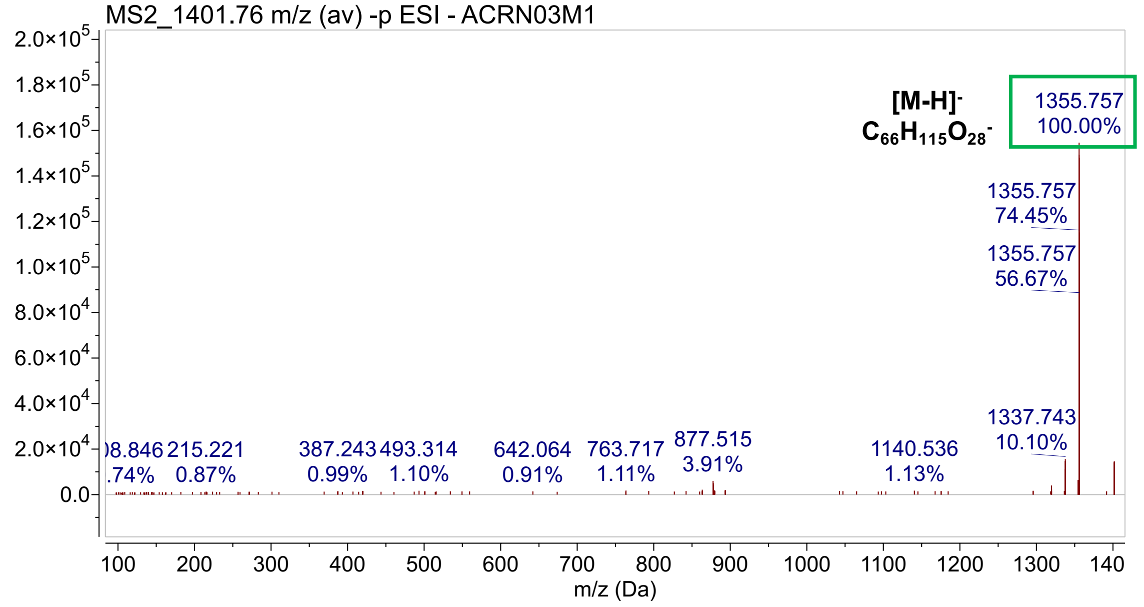

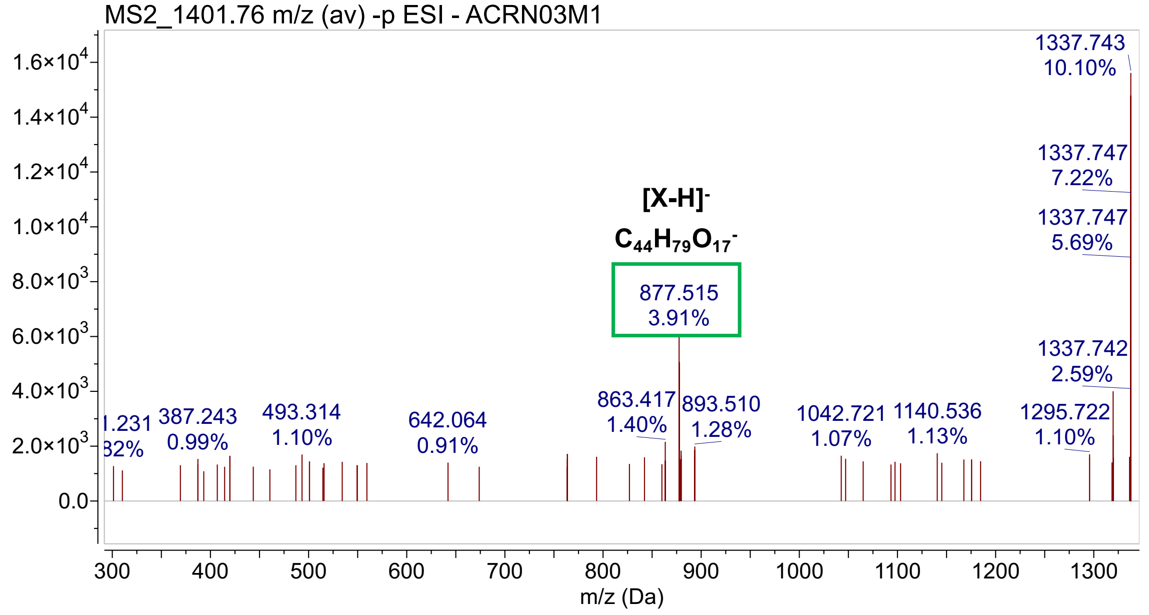

Figure S30. Common fragment ions found in luteophanol D, amphidinol 24 and amphidinols 27-36.

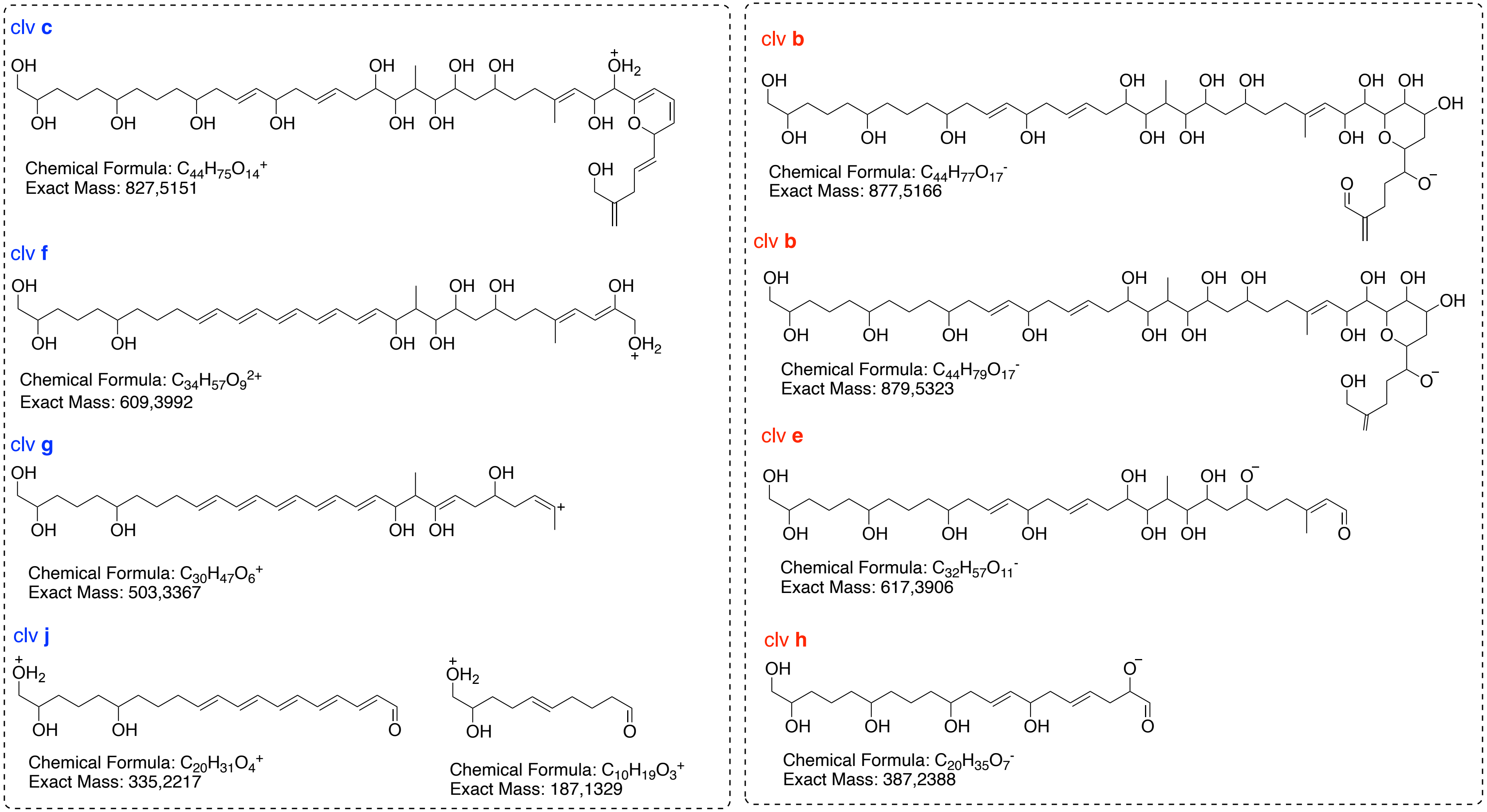

(Water loses and charges are drawn in arbitrary positions).

Figure S31. Fragment ions of lutophanol D.

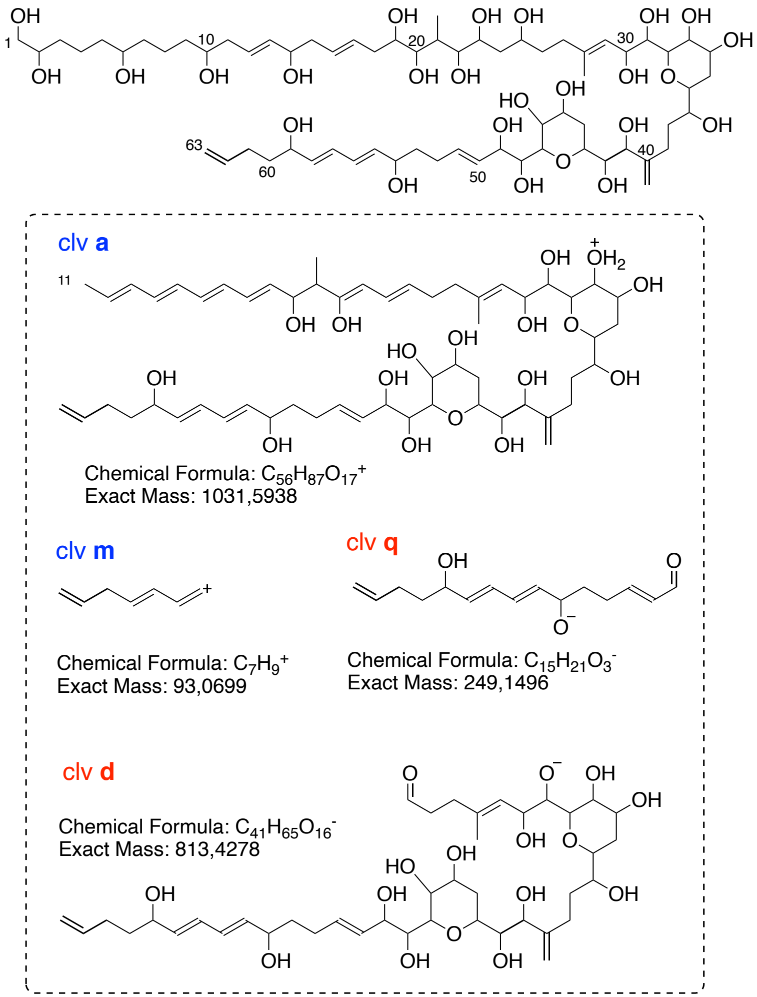

Figure S32. Fragment ions of amphidinol 28.

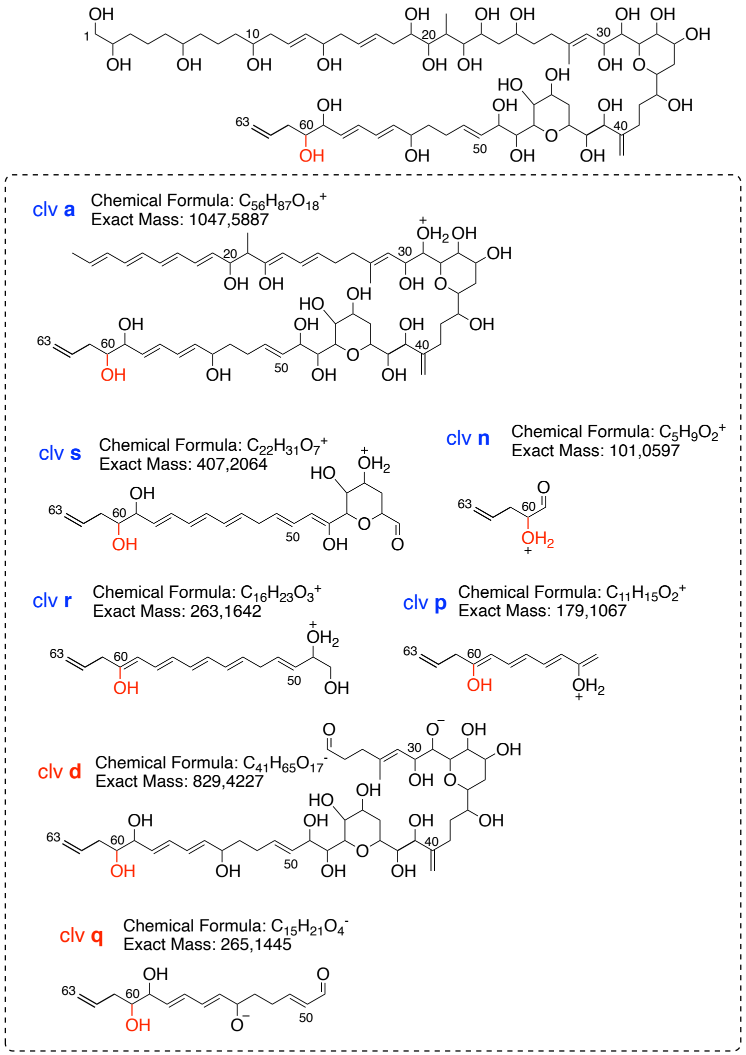

(Water loses and charges are drawn in arbitrary positions).

Figure S33. Fragment ions of amphidinol 20B.

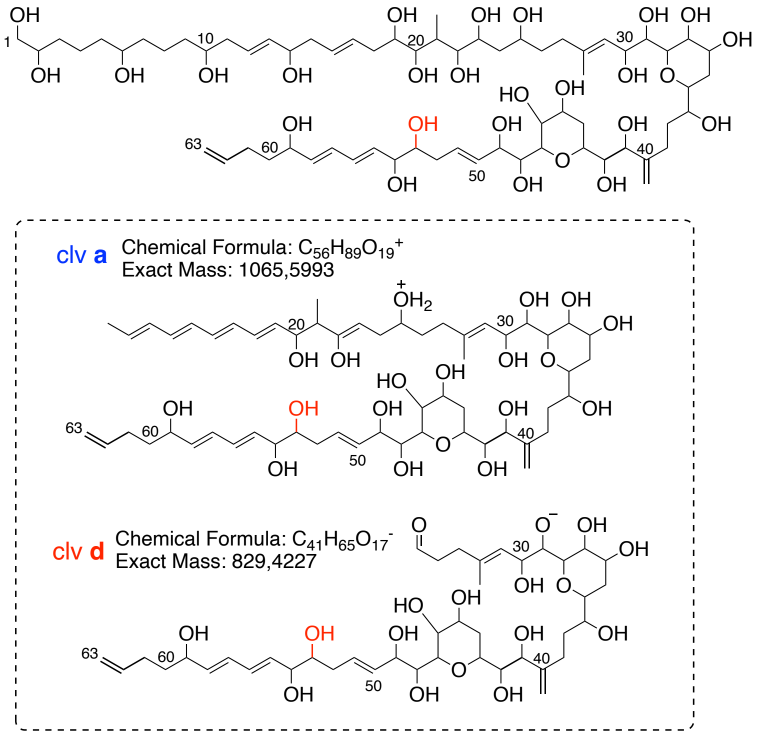

Figure S34. Fragment ions of amphidinol 29.

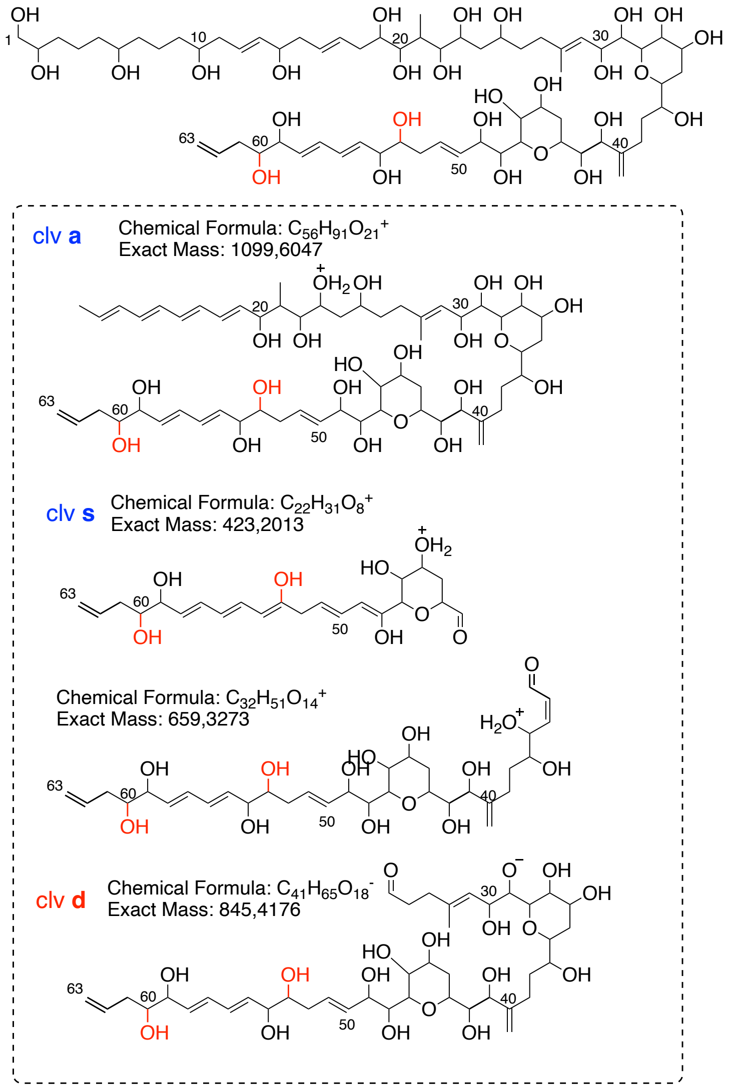

(Water loses and charges are drawn in arbitrary positions).

Figure S35. Fragment ions of amphidinol 27.

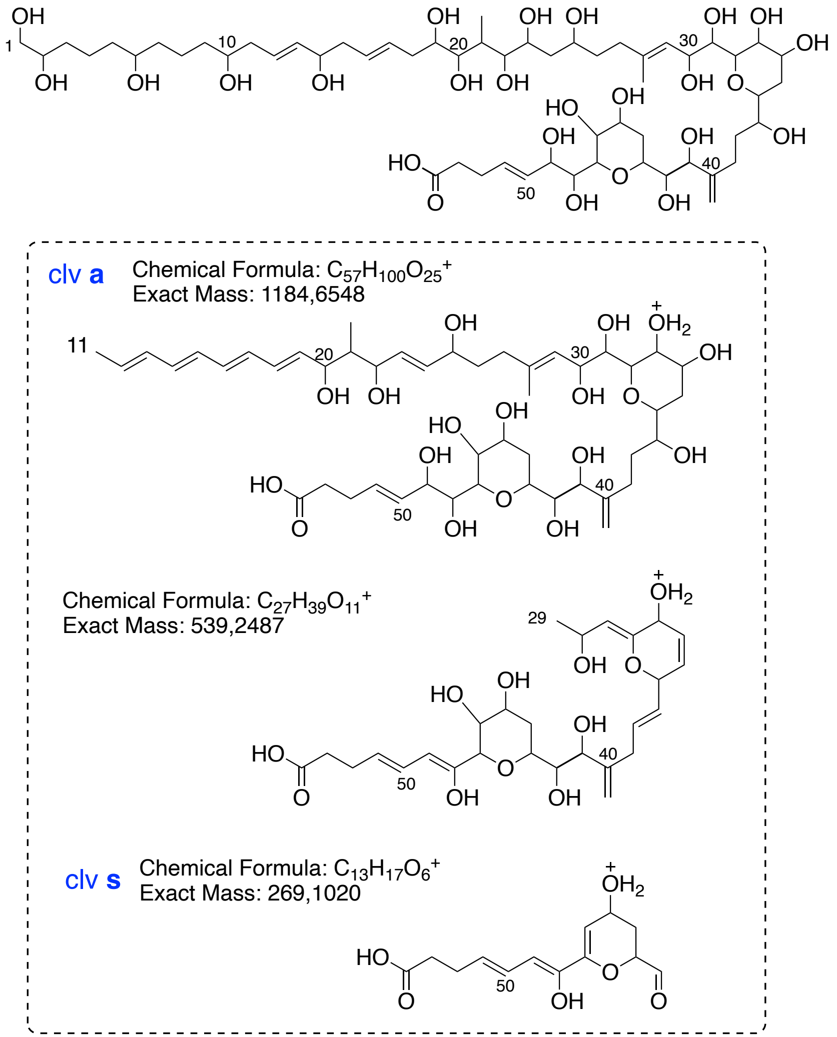

Figure S36. Fragment ions of amphidinol 30.

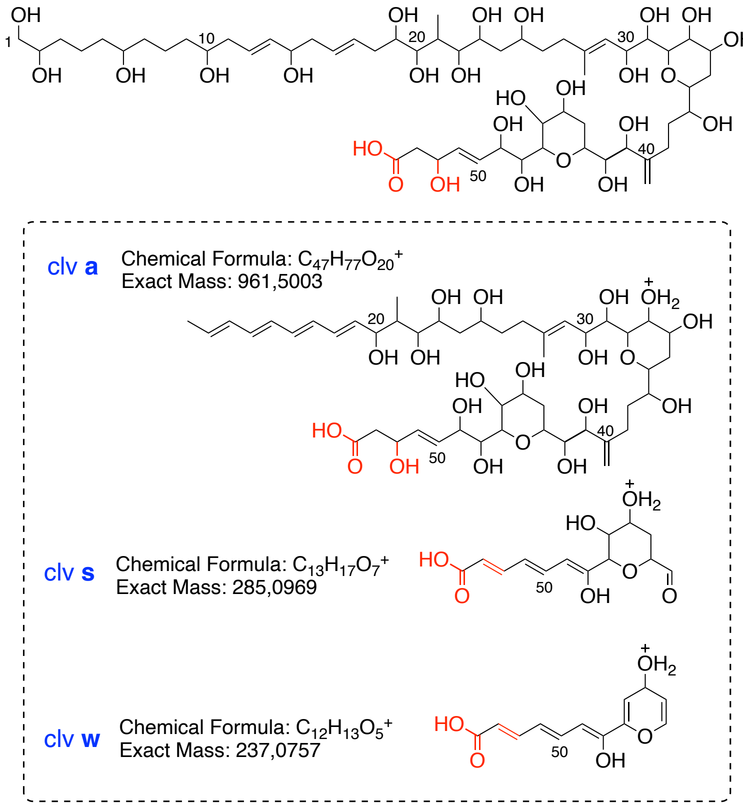

(Water loses and charges are drawn in arbitrary positions).

Figure S37. Fragment ions of amphidinol 31.

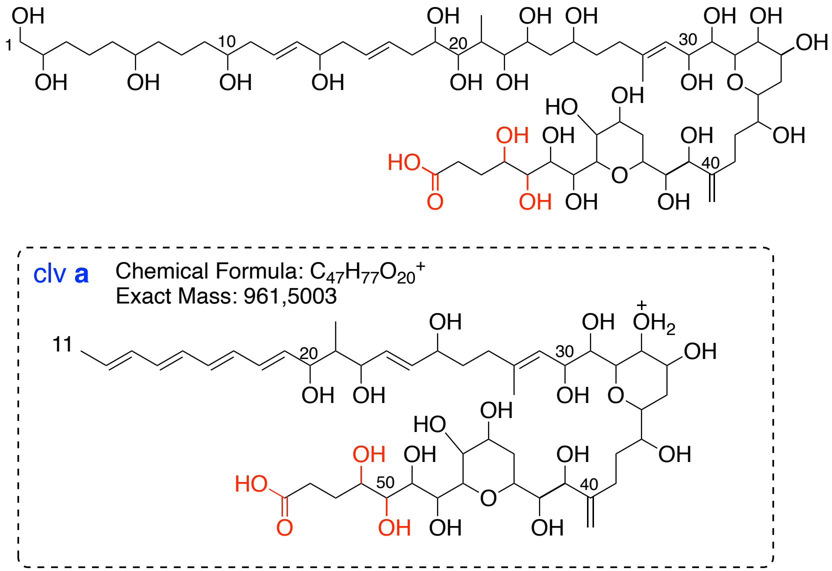

Figure S38. Fragment ions of amphidinol 32.

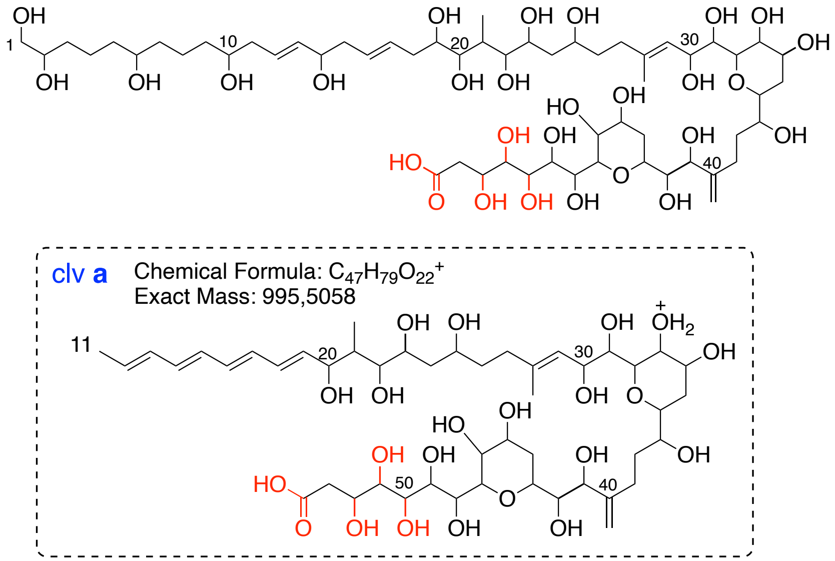

(Water loses and charges are drawn in arbitrary positions).

Figure S39. Fragment ions of amphidinol 33.

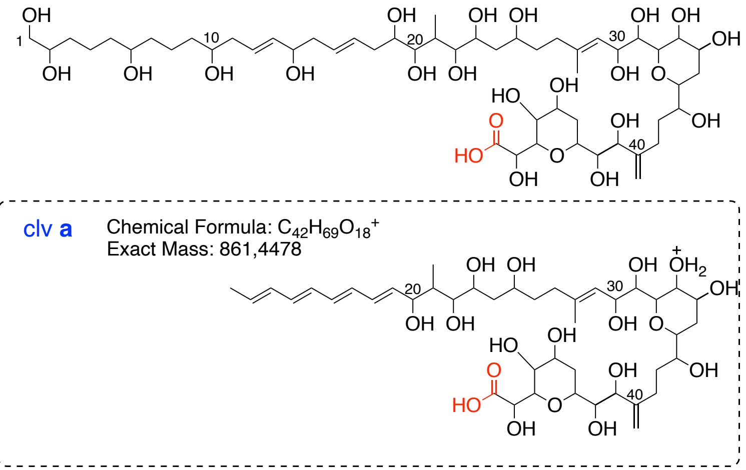

Figure S40. Fragment ions of amphidinol 34.

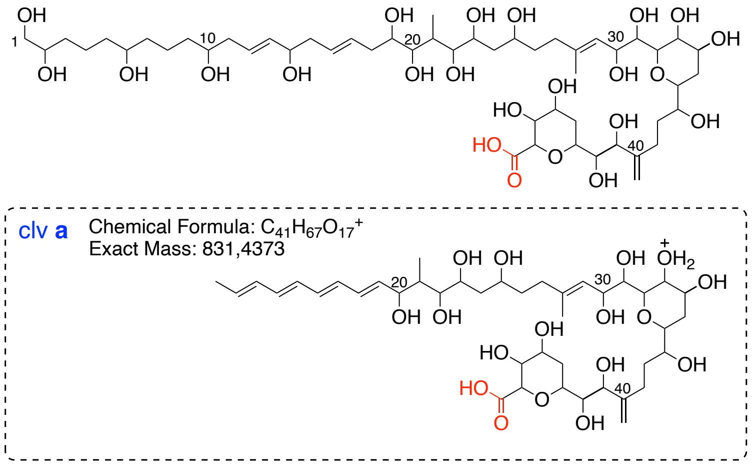

(Water loses and charges are drawn in arbitrary positions).

Figure S41. Fragment ions of amphidinol 35.

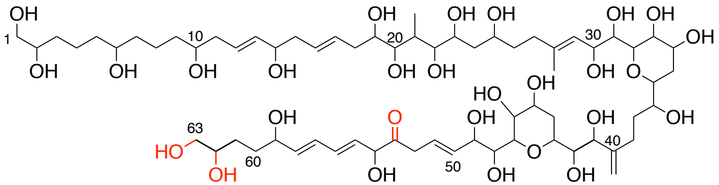

clv **a** Chemical Formula: $C_{56}H_{93}O_{23}^{+}$
Exact Mass: 1133,6102

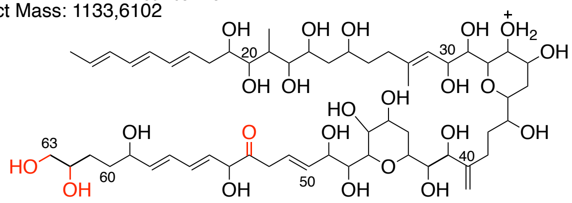

clv **s** Chemical Formula: $C_{22}H_{33}O_{9}^{+}$
Exact Mass: 441,2119

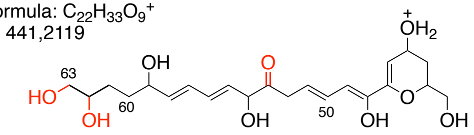

clv **v** Chemical Formula: $C_{10}H_{19}O_{4}^{+}$
Exact Mass: 203,1278

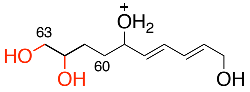

clv **w** Chemical Formula: $C_{8}H_{13}O_{3}^{+}$
Exact Mass: 157,0859

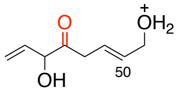

(To draw the fragment ions with the same formula and *m/z* than those reported in Reports S4-S34 we drawn the water loses and charges in arbitrary positions).
